# Food-Borne Chemical Carcinogens and the Evidence for Human Cancer Risk

**DOI:** 10.3390/foods11182828

**Published:** 2022-09-13

**Authors:** Tetyana Kobets, Benjamin P. C. Smith, Gary M. Williams

**Affiliations:** 1 Department of Pathology, Microbiology and Immunology, New York Medical College, Valhalla, NY 10595, USA; 2 Future Ready Food Safety Hub, Nanyang Technological University, Singapore 639798, Singapore

**Keywords:** carcinogens, food, DNA-reactants, epigenetic, risk assessment

## Abstract

Commonly consumed foods and beverages can contain chemicals with reported carcinogenic activity in rodent models. Moreover, exposures to some of these substances have been associated with increased cancer risks in humans. Food-borne carcinogens span a range of chemical classes and can arise from natural or anthropogenic sources, as well as form endogenously. Important considerations include the mechanism(s) of action (MoA), their relevance to human biology, and the level of exposure in diet. The MoAs of carcinogens have been classified as either DNA-reactive (genotoxic), involving covalent reaction with nuclear DNA, or epigenetic, involving molecular and cellular effects other than DNA reactivity. Carcinogens are generally present in food at low levels, resulting in low daily intakes, although there are some exceptions. Carcinogens of the DNA-reactive type produce effects at lower dosages than epigenetic carcinogens. Several food-related DNA-reactive carcinogens, including aflatoxins, aristolochic acid, benzene, benzo[a]pyrene and ethylene oxide, are recognized by the International Agency for Research on Cancer (IARC) as causes of human cancer. Of the epigenetic type, the only carcinogen considered to be associated with increased cancer in humans, although not from low-level food exposure, is dioxin (TCDD). Thus, DNA-reactive carcinogens in food represent a much greater risk than epigenetic carcinogens.

## 1. Introduction

Foods and beverages are essentially complex mixtures of chemicals consumed for either sustenance or pleasure. The diversity of chemicals found in food is vast, as are their varying properties. It has long been known that chemicals with carcinogenic activity in rodent models can be found in many commonly consumed foods [1,2,3,4,5] from a variety of sources including plants, microorganisms, contaminations, additive uses and reactions which occur during storage, processing and cooking [2] (Table 1). In addition, carcinogens can be formed endogenously, from food materials [6,7,8]. This review focuses mainly on carcinogens, both rodent and human, present in foods and beverages at low concentrations which are imperceptible, and a few components present at levels associated with adverse effects. It does not address drinking water contaminants, such as arsenic, or the contributions of caloric content and macro components such as fat content, or the excess consumption of alcoholic beverages, all of which, nevertheless, have been implicated in increased cancer risks in humans [9,10,11,12,13,14,15,16].

Chemical carcinogens exert their effects through two distinct types of mechanism of action (MoA), which have been characterized as DNA-reactive (genotoxic) and epigenetic (non-genotoxic) [17,18,19,20], as discussed below. Chemical structure determines the carcinogenic MoAs; DNA-reactive carcinogens have structures that form reactive electrophiles, either directly or following bioactivation, whereas epigenetic carcinogens lack such properties, but have structures that exert other molecular and cellular effects leading to cancer [17,18]. These differences in MoA underly the nature of human cancer risks from exposures [21,22].

### 1.1. Mechanisms of Carcinogenicity of DNA-Reactive Carcinogens

DNA-reactive carcinogens have structures that permit formation of electrophilic reactants that covalently bind (adduct) to nucleophilic sites in nuclear DNA, as well as in other macromolecules, including RNA and proteins, in the target tissue(s) of carcinogenicity [23,24,25]. In target tissue(s), a single DNA reactant can form different DNA adducts on various nucleophilic sites either on a single base or on different bases. Each adduct can undergo different rates of repair depending upon its location in the genome. For example, adducts in transcriptionally active regions are repaired by a transcription-coupled repair system whereas adducts in transcriptionally silent regions are repaired by a global repair system [26]. The levels of DNA adducts resulting from exposures are a function of several metrics including dose levels, the frequency of exposure, and rates of DNA repair for specific adducts. Each adduct has a characteristic efficiency with which it gives rise to mutations, with those at sites of base pairing being more mutagenic.

Pro-mutagenic DNA alterations are converted to mutations during cell replication [27,28,29]. Mutations in critical growth control genes lead to neoplastic conversion, and subsequent neoplastic development [28,30]. DNA-reactive carcinogens can also exert other cellular effects, such as cytotoxicity, leading to enhanced cell proliferation, which can contribute to their carcinogenic activity [31,32]. DNA-reactive carcinogens can have additive effects with one another in their target organ(s).

Some DNA adducts evidently do not lead to carcinogenicity, since some adducts can be found in tissues where no tumors are induced following administration of a carcinogen [33,34,35,36]. For example, acrylamide, which is discussed below, forms adducts in target and non-target tissues [37]. It could also be the case that epigenetic effects are required to enable neoplastic conversion resulting from some adducts [38,39].

As a result of DNA interactions, DNA-reactive carcinogens are typically genotoxic in assay systems in which appropriate bioactivation is represented [17,18,24,40,41]. Moreover, DNA-reactive carcinogens often produce tumors at multiple sites and with a short duration of exposure, even after administration of a single dose for some. This property underlies their activity in limited short-term bioassays [18].

Some DNA-reactive carcinogens have been demonstrated to exhibit no-observed-adverse-effect-levels (NOAELs) for carcinogenic effects in animal models [25,31,42,43,44,45,46], although conflicting data have been reported. Based on the steps for tumorigenesis, it is evident that biological thresholds that may influence the likelihood of cancer progression for genotoxic carcinogens exist. Nevertheless, currently, thresholds are not generally accepted for DNA-reactive carcinogens from a risk assessment and management perspective [47]. It is acknowledged that the derivation of NOAELs can be dependent on the study design, and more research is needed in this space. It is outside the scope of this paper to discuss thresholds for carcinogens in detail; however, this topic is reviewed elsewhere [25,31,42,43,44,45,46].

### 1.2. Mechanisms of Carcinogenicity of Epigenetic Carcinogens

Epigenetic carcinogens do not chemically react with DNA [17,20,48,49,50,51,52]. In the target tissue(s) of carcinogenicity, MoAs of these types of carcinogens involve molecular or cellular effects, which through secondary mechanisms, can either indirectly result in modification of DNA function or cell behavior [17,48]. For example, epigenetic carcinogens can induce oxidative stress, resulting in oxidative DNA damage [53,54,55], leading to either neoplastic conversion or stimulation of cell proliferation, thereby facilitating neoplastic development, often from cryptogenic pre-neoplastic cells. Epigenetic carcinogens can also affect gene expression [56,57], leading to neoplastic conversion. Such effects are often specific for rodents (e.g., d-limonene). Epigenetic carcinogens can enhance carcinogenicity of DNA-reactive carcinogens through interactive effects such as neoplasm promotion (e.g., butylated hydroxyanisole).

Due to their lack of direct DNA reactivity, epigenetic carcinogens, in contrast to DNA-reactive agents, are typically negative in genotoxicity assays, even in the presence of bioactivation, unless some artifact, such as extreme cytotoxicity, mediates mutagenicity. To exert their carcinogenicity, epigenetic agents often require prolonged high-level exposures. Their MoA underlies the fact that in limited bioassays they are negative for initiating activity, but may be positive for promoting activity [18].

Epigenetic carcinogens are well established to exhibit NOAELs for the cellular effect underlying their carcinogenicity in animal models [17,19], as discussed for several of the food-borne carcinogens reviewed herein. Accordingly, thresholds are generally accepted for DNA-reactive carcinogens from a risk assessment perspective [47].

## 2. Risk Assessment of Food-Derived Carcinogens

### 2.1. Application of Carcinogenicity Data to Human Risk

Two types of carcinogenicity data are used in the assessment of risk: human epidemiologic data and tumor data obtained in testing in rodent models [58]. The former is considered more relevant for a variety of reasons [59,60,61,62], although such data are often limited in human exposure information and can be poorly controlled [63].

Animal data are usually more robust, but frequently involve findings whose relevance to humans is uncertain [18,64,65], because the tumorigenic effect involves MoAs operational only in rodents. In addition, rodent studies do not mimic real life human exposures with respect to both the concentration and frequency of exposure. The human diet is also composed of mixture of components, which can both enhance and inhibit carcinogenicity.

Thus, in assessing human risk, two considerations are critical, i.e., the MoA of carcinogenicity and human exposure dose [21,25].

Once a chemical has been identified in a food product and its structure determined, it is possible to undertake an in silico analysis to determine, based on structure-activity relationships, the potential for DNA reactivity [66]. While this works well for relatively simple compounds, with the complexity of many natural products, the subtleties of metabolic activation become increasingly difficult to predict. If sufficient material is available, direct testing for DNA reactivity is the preferred approach [18].

This review focuses primarily on chemicals present in food that have sufficient evidence of carcinogenicity in either humans or experimental animals and which were classified by the International Agency for Research on Cancer (IARC) as either carcinogenic to humans (Group 1), probably (Group 2A) or possibly (Group 2B) carcinogenic to humans [58,67]. IARC also recognizes a third group of substances (Group 3) which lack sufficient evidence to be classified as carcinogenic to humans but nonetheless can have the potential to cause carcinogenicity in animals. Moreover, a variety of chemicals has not yet been characterized as to their carcinogenic risk to humans. Where available, evaluations by other expert groups are cited. Data on classification of carcinogens by government agencies and their carcinogenic potencies (TD_50_) calculated based on the tumorigenicity findings in rodents are provided in Table 2.

In this review, the evidence for human cancer risk from intake of food borne carcinogens of both the DNA-reactive and epigenetic types is discussed. In the assessment of risk from experimental studies, the greatest weight is given to studies with oral administration since that route of intake is most relevant to human consumption. The demonstration of human carcinogenicity is made in epidemiologic studies, although, the absence of an effect can be due to inadequacy of the studies.

### 2.2. Risk Assessment of DNA-Reactive Rodent Carcinogens

In order to evaluate possible safety concerns arising from presence of carcinogens with DNA-reactive MoA in the diet, many regulatory and advisory agencies, including the European Food Safety Authority Panel on Contaminants in the Food Chain (EFSA CONTAM) and the Joint Food and Agriculture Organization of the United Nations (FAO)/World Health Organization (WHO) the Expert Committee on Food Additives (JECFA) use a margin of exposure (MoE) approach [69,70]. MoE is calculated as a ratio between an appropriate Point of Departure for a tumor response, such as NOAELs obtained from animal studies, and a predicted or estimated human exposure level. A number of considerations should be taken into account when a MoE is derived, including the biological relevance of carcinogenic MoAs to humans [65].

Among DNA-reactive rodent carcinogens, only aflatoxins, aristolochic acid I, benzene, benzo[a]pyrene and ethylene oxide, have been found to be associated with cancer causation in humans (Table 2). Nevertheless, all materials in this class are genotoxic, indicating an MoA that represents human risk [22].

### 2.3. Risk Assessment of Epigenetic Carcinogens

The contribution and relevance of epigenetic mechanisms produced by dietary factors leading to the development of cancer in humans is uncertain [71], and the best approach to risk assessment of such carcinogens remains a topic of a debate [72]. Nevertheless, at low intermittent exposures (less than 1 mg/day) epigenetic carcinogens are not considered to pose cancer risks to humans [21]. This may reflect the absence in humans of the processes involved in the MoAs in rodents, e.g., d-limonene alpha 2μ(α_2μ_)-globulin nephropathy in male rats leading to kidney tumors [73], or the much lower exposures of humans, e.g., forestomach irritation in rats caused by butylated hydroxyanisole leading to squamous cell carcinoma [74]. Additionally, the fact that epigenetic changes can be reversible could contribute to lack of human risk. Hence, for epigenetic carcinogens NOAELs are used to derive safety values, such as tolerable daily intake (TDI) [21].

## 3. DNA-Reactive Carcinogens and Related Chemicals Present in Food

**Figure 1:**
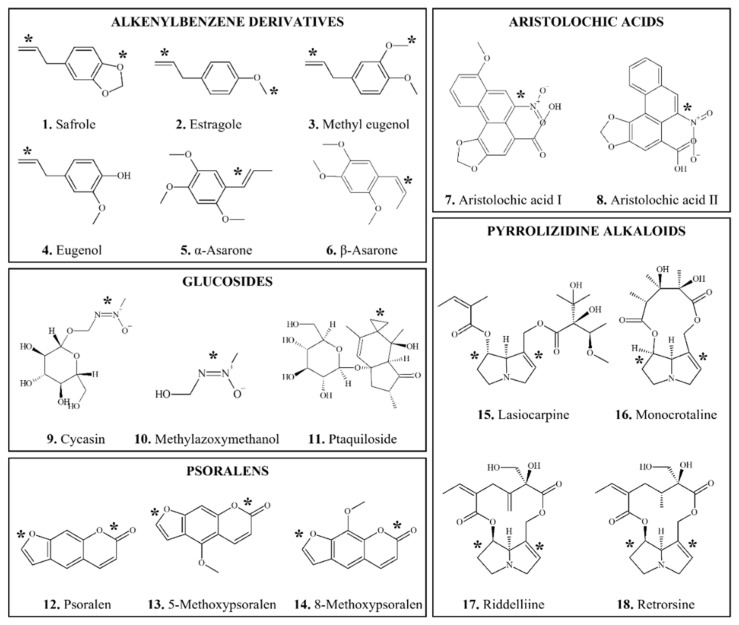
Chemical structures of DNA-reactive carcinogenic phytochemicals and related chemicals present in foods. Asterisks indicate sites of activation.

**Figure 2:**
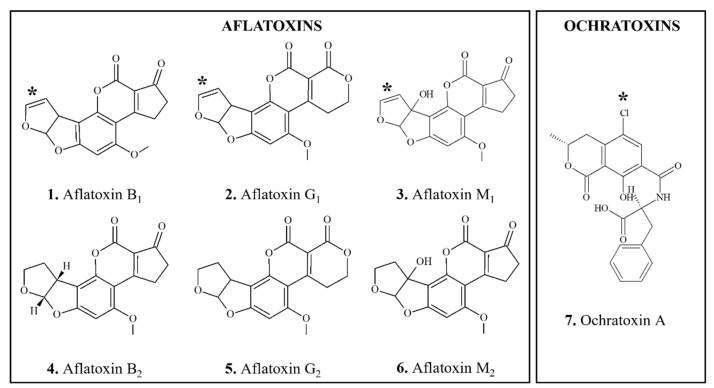
Chemical structures of DNA-reactive carcinogenic mycotoxins and related chemicals present in foods. Asterisks indicate sites of activation.

**Figure 3:**
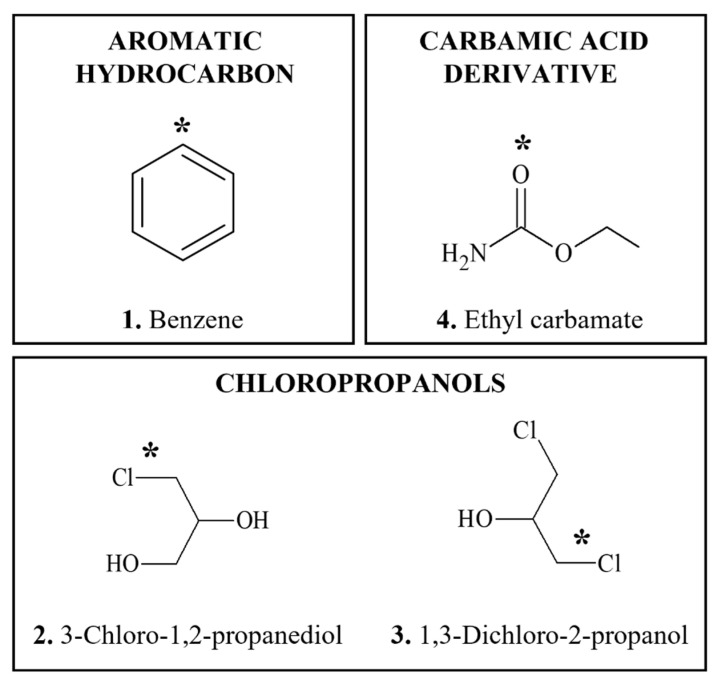
Chemical structures of DNA-reactive carcinogens formed during food processing and related chemicals present in foods. Asterisks indicate sites of activation.

**Figure 4:**
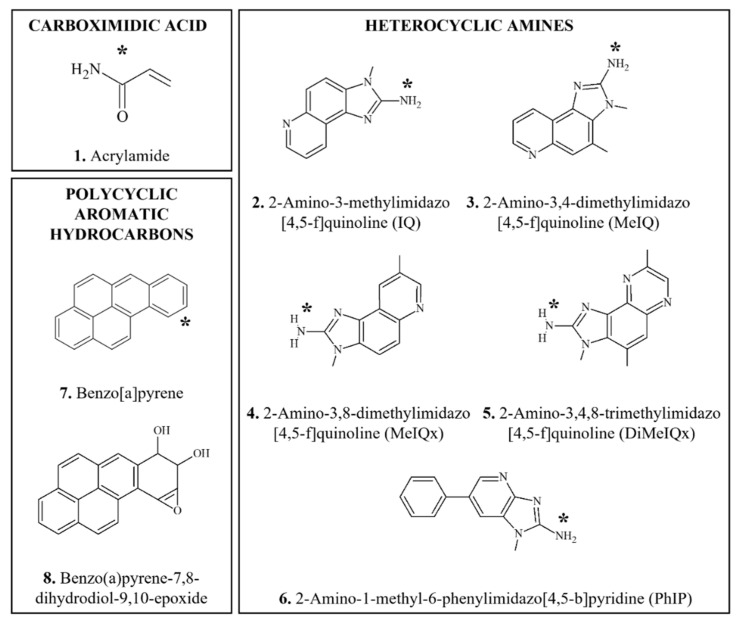
Chemical structures of DNA-reactive heat-generated carcinogens and related chemicals present in foods. Asterisks indicate sites of activation.

**Figure 5:**
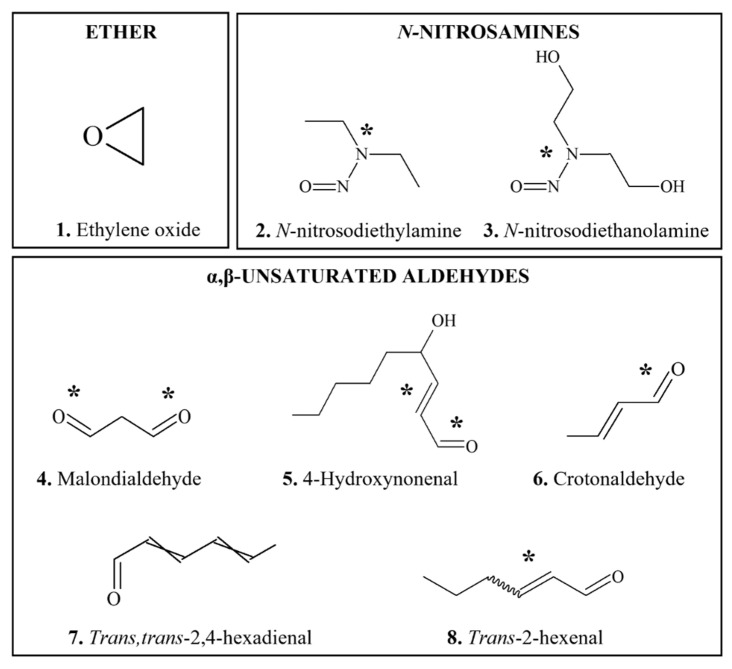
Chemical structures of DNA-reactive carcinogens and related chemicals formed endogenously and exogenously present in foods. Asterisks indicate sites of activation.

This section provides an overview of food-derived carcinogens that typically produce genotoxic and mutagenic effects in vitro and/or in vivo, in particular with appropriate bioactivation. Chemical structures of DNA-reactive carcinogens and related chemicals discussed in this section are provided in Figure 1, Figure 2, Figure 3, Figure 4 and Figure 5.

### 3.1. Phytotoxins

A recent inventory of botanical ingredients that are of possible concern for human health because of their genotoxic and carcinogenic properties revealed that the majority of the compounds identified belong to the group of alkenylbenzenes or the group of unsaturated pyrrolizidine alkaloids [75].

#### 3.1.1. Alkenylbenzene Derivatives

*Alkenylbenzene* (AB) compounds (Figure 1(1–6)) are important constituents of herbs and spices such as nutmeg (*Myristica fragrans*), cinnamon (*Cinnamomum verum*), anise star (*Illicium verum*), tarragon (*Artemisia dracunculus*), sweet basil (*Ocimum basilicum*), and sweet fennel (*Foeniculum vulgare*) which are present in the modern food chain mainly as a result of use of these herbs and spices and the use of their essential oils as flavorings [76]. There are two general types of ABs, methylenedioxyallylbenzenes and methoxyallylbenzenes [77,78], with different potential for bioactivation.

ABs are well absorbed following oral intake [79]. Biotransformation pathways are influenced by dose; at lower doses, ring oxidation occurs, whereas, at higher doses, the allyl side chain is also oxidized ultimately through sulfate ester formation to chemically reactive intermediates [78,79,80,81,82]. Polymorphisms in metabolism and lifestyle differences are likely to influence metabolism of these compounds [83].

Some ABs are DNA-reactive, as shown in a study in mice in which several cola beverages were administered in place of drinking water leading to formation of significant levels of DNA adducts in the livers [84]. These adducts were detected in mice treated with extracts of nutmeg or mace, or myristicin (1-allyl-5-methoxy-3,4-methylenedioxybenzene), the major spice constituent of nutmeg and smaller amounts of adducts derived from safrole, a minor constituent of nutmeg.

The ABs discussed in this section have exhibited carcinogenic activity in rodents. Other ABs, such as eugenol (Figure 1(4)) and methyl isoeugenol, with structures not conducive to formation of an electrophile have not been found to be carcinogenic under conditions in which related ABs with structures that form electrophiles were [78,82,85,86].

##### 3.1.1.1. Safrole

*Occurrence: Safrole* (SAF) (4-allyl-1,2-methylenedioxy-benzene) (Figure 1(1)), the prototype compound of the AB group, is present in *Sassafras*, nutmeg, cinnamon, sweet basil and star anise [77,82,87]. Until 1960, the beverage root beer contained approximately 30 ppm safrole it being made from the root of *Sassafras albidum* which contains about 85% safrole in the essential oil from its root bark [88].

*Carcinogenicity*: Dietary administration of SAF at up to 5000 mg/kg body weight (bw) to mice and rats caused increases in the incidences of hepatocellular carcinoma or cholangiocarcinoma [78,89,90]. SAF carcinogenicity in mice was strain specific [91]. The hepatocarcinogenicity of 1′-hydroxy SAF metabolites has been also demonstrated [91,92].

*Genotoxicity/DNA Binding (Adducts)*: Genotoxicity tests yielded inconclusive results, being generally negative in vitro, although, some genotoxicity was observed in vivo [78,93,94]. SAF also induced in vitro chromosomal aberrations sister chromatid exchange (SCE), unscheduled DNA synthesis (UDS) and DNA damage [90]. Nevertheless, guanine derivative SAF-DNA adducts were isolated from the livers of multiple species, including rats, mice [82,90,95,96], chicken and turkey [85,86] and humans [97,98].

*Biotransformation:* SAF undergoes bioactivation primarily on its side chain (Figure 1(1)) to form a hydroxy metabolite which is subsequently sulfated [99,100]. These reactions involve several cytochrome P450 (CYP) enzymes, especially CYP1A2 [83] and sulfotransferase [101]. Genotoxic effects of SAF are likely mediated by metabolites, 1′-hydroxysafrole and 1′-sulfoxysafrole [77]. A number of hydroxylated metabolites have been isolated from human urine [102].

*MoA*: SAF was considered to be a genotoxic carcinogen, based on its ability to induce formation of DNA adducts [90].

*Human Exposure*: Humans may ingest SAF with edible spices, such as sassafras, cinnamon, nutmeg, mace, star anise, ginger, black and white pepper, and from chewing betel quid [68]. An Estimated Daily Intake (EDI) for SAF was reported to be 300 μg/person/day [82,90]. JECFA [78] estimated the intake of safrole to be around 879 μg/person/day.

*Human Effects:* Most of the evidence that SAF may be carcinogenic to humans comes from studies of individuals who chew betel quid, which is known to contain up to 15 mg/g SAF. Thus, SAF-like DNA adducts have been reported in oral squamous cell cancers [97,103] and hepatocellular carcinoma [104] isolated from users of betel quid. Betel quid users are known to have an increased risk for oral cancer development [103].

*Risk*: SAF was classified by the IARC [87] as possibly carcinogenic to humans (Group 2B) (Table 2). Reflecting these concerns, JECFA did not allocate an Acceptable Daily Intake (ADI) [78]. The direct addition of SAF to food is prohibited in the USA (21 CFR § 189.180) [105] and Europe (Regulation EC No. 1334/2008) [106]. Nevertheless, exposure to SAF continues to occur [107].

##### 3.1.1.2. Estragole

*Occurrence: Estragole* (1-methoxy-4-(2-propenyl)-benzene) (Figure 1(2)) is a natural constituent of a number of aromatic plants and their essential oil fractions including among others tarragon, sweet basil, sweet fennel and anise star [77,78,82,108]. As a flavoring agent it is used at maximum levels of 50 ppm [79].

*Carcinogenicity:* Estragole and its 1′-hydroxy metabolite were hepatocarcinogenic in mice when administered in diet at doses up to 600 mg/kg bw for 12 months [91,92]. In mice susceptibility to estragole carcinogenicity was strain specific [91]. In rats, estragole administered by gavage up to 600 mg/kg bw, 5 days/week for 3 months showed evidence of carcinogenic activity, increasing incidences of cholagiocarcinomas and hepatocellular adenomas [109].

*Genotoxicity/DNA Binding (Adducts):* Genotoxicity and DNA binding of estragole has been reported [78,79,85,96,98,110,111,112]. However, it was primarily negative in in vitro tests [109], likely due to inadequate bioactivation [25].

*Biotransformation*: With regard to metabolism, studies in rats indicate that the proximate carcinogen, the 1′-hydroxy metabolite, was produced in minimal amounts at doses in the range of 1–10 mg/kg bw/day [79]. In humans, this metabolite appears to be produced at an even lower rate [113]. These considerations would argue for the existence of a practical threshold for carcinogenic risk in human population [114].

*MoA:* Formation of DNA adducts and genotoxicity are considered to underly carcinogenicity of estragole [79,108].

*Human Exposure*: Based on the annual production volume for flavoring substances, the per capita intake of estragole in the US is 5 μg/day [78], while other sources estimated average baseline exposures to estragole from food intake to range from 500 to 5000 μg/day, with an average exposure of 1000 μg/person/day [108].

*Human Effects*: No evidence for human carcinogenicity of estragole is available [108].

*Risk*: The Expert Panel of the Flavor and Extract Manufacturers’ Association (FEMA), concluded that based on the fact that genotoxic and carcinogenic effects of estragole are dose dependent, present dietary exposures to estragole do not pose a significant cancer risk to humans [79]. However, JECFA indicated that further research is required to assess potential human risk from low-level exposures [78]. Analyses of cancer responses in rodents demonstrated that thresholds for estragole carcinogenicity were well above the levels normally associated with human consumption [114]. Based on the carcinogenic potency, the European Medical Agency (EMA) [108] calculated an ADI for adults of 52 μg/person/day.

##### 3.1.1.3. Methyl Eugenol

*Occurrence*: *Methyl eugenol* (ME) (1,2-dimethoxy-4-(2-propenyl)benzene) (Figure 1(3)) occurs in a variety of plants, including nutmeg, sweet basil, tarragon, allspice and pimento [77,78,79,82,115]. Both ME and eugenol (Figure 1(3,4)) were found in juice from oranges treated on the tree with rind-injuring abscission agents used to loosen the fruit for mechanical harvesting [116]. As a flavoring agent, ME was used in the past at a maximum level of 50 ppm [79]; however, since 2008, ME has been banned for direct addition to foods in Europe (Regulation EC No. 1334/2008) [106].

*Carcinogenicity*: In a 2-year study, with ME administered to rats and mice of both sexes at doses up to 150 mg/kg bw by gavage, 5 days/week for 105 weeks, chemical-related increases in liver neoplasms occurred in all dosed groups of rats [79,115,117]. In the glandular stomach, mucosal atrophy, an early indication of potential neoplasia, was increased at all doses in rats and malignant gastric neuroendocrine tumors were observed in high dose group in male mice. In rats, gastric neuroendocrine cell hyperplasia was evident at 6 months and neuroendocrine tumors occurred in the high dose group. Other neoplasms with increased incidence included forestomach squamous cell papilloma or carcinoma, renal tubule adenomas, malignant mesotheliomas, mammary gland fibroadenomas and fibromas of the subcutaneous tissue [115,117].

*Genotoxicity/DNA Binding (Adducts)*: ME tested generally negative in genotoxicity tests in vitro and in vivo [115,117]. However, it induced chromosomal aberrations and UDS in vitro [82,115] and formed DNA adducts in human hepatocytes [98] and the livers of rats [118], turkey and chicken fetuses [85,86]. Moreover, correlation between formation of DNA adducts and tumor formation has been shown for ME, and a threshold for tumors was calculated at 10^20.1^ molecules/kg/day [119]. Results of PBPK modelling for rats and humans support validity of linear extrapolation of ME tumor data from rodents to humans [120]. However, the application of this log/linear plot for extrapolation is not uniformly accepted [121].

*Biotransformation:* Similar to other ABs discussed above, ME is bioactivated by CYP1A2 through hydroxylation at the 1′ position (Figure 1(3)) to produce reactive 1′-hydroxymethyleugenol, followed by sulfation. Other metabolic pathways include oxidation of the 2′,3′-double bond to form ME-2,3-oxide and O-demethylation followed by spontaneous rearrangement to form eugenol quinone methide [68,79,115].

*MoA:* DNA-binding of 1′-hydroxy ME metabolite most likely underlies MoA for the several types of ME-induced neoplasms [115,122]. In rat liver, ME rapidly induced preneoplastic lesions indicating tumor initiating activity [118]. In addition, based on mechanistic studies of other chemicals that have induced gastric neuroendocrine tumors [123], the mucosal atrophy may have produced decreased hydrochloric acid production which stimulates gastrin production leading to neuroendocrine cell proliferation, and eventually to neuroendocrine neoplasia.

*Human Exposure*: The overall EDI of ME in US from dietary sources was estimated to be 0.77 μg/kg bw/day, with basil, nutmeg and allspice being primary sources of exposure [79]. JECFA calculated mean per-capita dietary exposure to ME of 80.5 μg/day in US and 9.6 μg/day in Europe [78]. The total dietary intake of food containing ME was calculated to be 66 μg/kg bw/day for regular consumers [122].

*Human Effects*: No epidemiological studies evaluating evidence of human carcinogenicity from ME are available [68,115].

*Risk:* ME has been classified by IARC [115] as possibly carcinogenic to humans (Group 2B) (Table 2) based on sufficient evidence for carcinogenicity in animals. While FEMA concluded that present exposures to ME do not pose significant risk to human health [79], estimated MoE based on the dose–response modelling ranges from 100 to 800, suggesting that the dietary intake of ME is of high concern [122]. In 2018, the FEMA Expert Panel removed ME from the FEMA Generally Recognized as Safe (GRAS) list, citing the need for additional data to clarify the relevance of DNA adducts formed by ME in humans [124].

##### 3.1.1.4. α- and β-asarone

*Occurrence*: Propenylic phenylpropenes, *α-* and *β-asarone* ((E)-/(Z)-1,2,4-trimethoxy-5-prop-1-enylbenzene) (Figure 1(5,6)), are constituents of essential oils (e.g., calamus oil) which are present in certain plants such as *Acorus* spp. and *Aarum* spp. and are used as flavoring agents [125,126]. *β-asarone* content varies with the source of the plant; Indian plant oil is approximately 75–95% β-asarone, whereas European is 5–10% [127,128].

*Carcinogenicity*: When fed to rats for 2 years at doses up to 2000 mg/kg bw, *β-asarone* induced leiomyosarcomas of the small intestine of males but not females [126,127,128]. Feeding Indian calamus oil at 0.05% and greater produced intestinal tumors in male and female rats, while feeding European calamus oil induced leiomyosarcomas and additionally, liver neoplasms at 1% and greater. Hepatocarcinogenicity of *α-* and *β-asarone* was also reported following oral administration or intraperitoneal injections to mice [91,129].

*Genotoxicity/DNA Binding (Adducts)*: In the in vitro genotoxicity assays, *α-* and *β-asarone* produced conflicting results, while in vivo mutagenicity data is limited [128]. Nevertheless, positive results in the in vitro mutagenicity assays were obtained in the presence of bioactivating systems or in metabolically competent cell lines, including human Hepa-G2 cells [94,126,129,130,131]. Asarones also induced SCE, UDS and DNA breaks in vitro [126,132]. Both isomers produced DNA adducts in rat hepatocytes [133] and in avian embryos [86].

*Biotransformation*: In rat hepatocytes, the major metabolite of asarones was 2,4,5-trimethoxycinnamic acid, which was not genotoxic [131]. In rat and human liver microsomes epoxide-derived side-chain diols were the major metabolites, and the major bioactivation pathway for *α-asarone* was considered to be 3′-hydroxylation of propenylic side chain by CYP1A2, while for β-asarone, epoxidation by CYP3A4 prevails [126,134,135,136]. O-demethylation catalyzed by CYP1A1, 2A6, 2B6, and 2C19 was a minor reaction.

*MoA:* The mutagenicity and DNA binding of side chain epoxides formed during bioactivation of asarones suggests that this intermediate is responsible for carcinogenic effects, at least in the liver [129,136]. The MoA for induction of the intestinal tumors remains undetermined.

*Human Exposure*: The primary source of human exposure to asarones is through the consumption of alcoholic beverages such as bitters, liqueurs and vermouths, in which levels of calamus oil have been detected up to 0.35 mg/kg [128]. While no regulations for the use of *α-asarone* are currently in place, limits of 0.1 and 1 mg/kg are set for *β-asarone* in food and alcoholic beverages, respectively [126]. Nevertheless, some alcoholic drinks can contain up to 4.96 mg/kg of *β-asarone* [128]. Based on limited British data, maximum EDI for *β-asarone* is approximately 115 µg/day or 2 µg/kg bw/day [127,128].

*Human Effects*: No epidemiological studies investigating association of asarones with human cancer risk has been reported; however, some in vitro studies indicate anticarcinogenic properties of *β-asarone* [137,138].

*Risk:* JECFA and the Scientific Committee on Food (SCF) concluded that the existence of a threshold cannot be assumed for *β-asarone* due to its genotoxicity [127,128]. Accordingly, an ADI for nutritional exposure could not be derived. Committees recommended that calamus oil used in foods should have the lowest practicable levels of *β-asarone*. Calamus oil and its extracts are prohibited from use in the USA (21 CFR § 189.110) [139].

#### 3.1.2. Aristolochic Acids

*Occurrence: Aristolochic acid I* (AAI) (8-methoxy-6-nitrophenanthro[3,4-d]-l,3-dioxole-5-carboxylic acid) (Figure 1(7)) is one of a group of about 14 AAs known to be present in plants belonging to the family *Aristolochiaceae* (Birthwort family). Species known to contain AAs include *A. contorta*, *A. debilis*, *A. fangchi*, and *A. manshuriensis* [15,68,140,141,142].

*Carcinogenicity*: AAI, either purified or as a mixture with AAII (Figure 1(8)), was carcinogenic in rats and mice after oral exposure producing tumors predominantly in the forestomach and in the kidneys [15,68,140,143,144]. Other target organs of carcinogenicity include lung, uterus and lymphatic system in female mice and urinary bladder, thymus, small intestine and pancreas in rats. In addition, extracts from *Aristolochia* plants, *A. manshuriensis* and *A. fructus* induced forestomach and kidney tumors in rats when administered orally [15].

*Genotoxicity/DNA Binding* (Adducts): AAI and AAII, have been found to be genotoxic in vitro and in vivo [141,145,146] and to form DNA adducts in vitro and in rodent tissues [141,147,148,149,150,151], as well as in humans urothelial tissues of patients with Chinese herb nephropathy, Balkan endemic nephropathy or urothelial cancer [152,153]. The major AA-specific DNA adducts were 7-(deoxyadenosin-*N*6-yl)aristolactam and 7-(deoxyguanosin-*N*2-yl)aristolactam [141]. Adducts of deoxyguanosine and deoxyadenosine were found in animal studies in both target (forestomach) and non-target tissues (glandular stomach, liver, lung, and bladder). In addition, AAs can bind to codon 61 of the *ras* oncogene and to purines in the *p53* tumor suppressor gene [68,141,153].

*Biotransformation:* Bioactivation of AAI occurs by nitro reduction in the presence of NAD(P)H quinone oxidoreductase and CYP1A2 [154] leading to formation of a nitrenium ion which, by rearrangement reactions, forms adducts on both deoxyguanosine and deoxyadenosine, the latter being biologically more stable [155].

*MoA*: Covalent binding to DNA and resulting mutagenicity is the predominant MoA of AAI carcinogenicity [15,68]. The most frequently observed mutation is a single *TP53* mutation (A to T transversion), consistent with the presence of persistent AAI-adenine adducts in DNA of exposed patients [141,153,156].

*Human Exposure*: AAs are present in herbal products and several teas made from *Aristolochia* plants [68,157] and in wild ginger used by North American Indians [158]. A combined EDI for AAI and AAII was calculated to be 1.7 × 10^−3^–30 µg/kg bw/day [142].

*Human Effects*: Consumption of herbal supplements containing AAs has been linked to nephropathy [159] and cases of urothelial cancer [160,161]. Among patients with AA nephropathy, the rate of urothelial cancer is much higher compared to the prevalence of transitional-cell carcinoma of the urinary tract [68].

*Risk*: Based on the evidence that AA-specific DNA adducts and *TP53* mutations have been described in humans, IARC [15] upgraded classification of AAI from probable human carcinogen (Group 2A) to human carcinogen (Group 1) (Table 2). MoEs for kidney tumor formation calculated based on the rodent data were below 10,000 indicating risk to humans [142]. The US Food and Drug Administration (FDA) advised consumers in 2001 to discontinue use of botanical products that contain AA; however, exposure to AA continues despite its known hazards [162].

#### 3.1.3. Glucosides

##### 3.1.3.1. Cycasin

*Occurrence: Cycasin* (methylazoxymethanol-D-glucoside) (Figure 1(9)) is a glucoside produced by the cycad nut, which grows in most tropical climates [89,163]. The amount of cycasin ranges from 0.02% to 2.3% [89].

*Carcinogenicity*: With oral administration, cycasin induced neoplasia in mice, rats, hamsters, guinea pigs, and monkeys mainly in liver, kidney and colon [89,164]. A metabolite of cycasin, methylazoxymethanol (MAM) (Figure 1(10)), has also been shown to induce hepatocellular carcinomas and tumors in other organs, including kidneys and intestinal tract, in nonhuman primates [164], and colon carcinogenesis in rodents [165,166,167,168].

*Genotoxicity/DNA Binding (Adducts)*: Cycasin was genotoxic after removal of a sugar residue to yield the aglycone, MAM, which is an alkylating agent [89,169,170,171,172,173]. MAM produced DNA adducts, specifically O^6^-methylguanine and *N*7-methylguanine, in vitro and in vivo in rats and guinea pigs [174,175,176,177,178].

*Biotransformation*: Bioactivation of cycasin to MAM occurs by hydroxylation of the methyl group, a reaction which is catalyzed by CYP2E1 [179]. Interspecies differences in metabolic bioactivation of cycasin to MAM was suggested to underly different susceptibility to its carcinogenicity [178].

*MoA:* The genotoxic metabolite MAM was shown to target cellular processes involved in carcinogenesis [180].

*Human Exposure*: Human exposure to cycasin is limited since cycad nuts are no longer used as a source of starch. Cycasin can, however, contaminate improperly prepared flour, as has occurred in Guam, where concentrations of cycasin ranged from 0.004 to 75.93 μg/g [175].

*Human Effects*: Human ingestion of cycad plant toxins has been associated with neurodegenerative disorders in inhabitants of Guam [181,182], but no appreciable increase in cancer mortality was evident at 2 to 7 years after heavy intake [89]. Cases of acute toxicity from high exposures have been reported but all with complete initial recovery [183].

*Risk*: IARC [89] classified both, cycasin and MAM, as possibly carcinogenic to humans (Group 2B) (Table 2).

##### 3.1.3.2. Ptaquiloside and Bracken Fern

*Occurrence*: *Ptaquiloside* (Figure 1(11)) is an unstable norsesquiterpene glucoside of the illudane type [184,185]. It is present in bracken fem (*Pteridium aquilinum*), in wild species and in products made from fronds at concentration ranges of 6300 ± 520 and 44 ± 3 μg/g, respectively [186]. High quantities of ptaquiloside, in various studies ranging from 0.0006 to 0.0058 μg/mL, were found in the milk from farm animals that consume diet containing bracken fern [187,188,189,190].

*Carcinogenicity*: Ingestion of bracken fern by cattle and sheep has been reported to cause cancers of the esophagus and urinary bladder [191,192]. Feeding of bracken fern to rats and mice induced intestinal and bladder cancers [193], which was initially attributed to the content of quercetin [194], but ptaquiloside was subsequently demonstrated to be the carcinogenic constituent [190,195]. With oral administration, ptaquiloside induced tumors of mammary glands, ileum and urinary bladder in female rats [196,197] and oral squamous cell carcinomas in HPV16-transgenic mice [198].

*Genotoxicity/DNA Binding (Adducts)*: Ptaquiloside was genotoxic in bacterial mutagenicity assays and in the rat hepatocyte primary culture DNA-repair assay [199,200,201]. In addition, it produced chromosomal aberrations in Chinese hamster lung cells and human mononuclear blood cells [202,203], and DNA damage in human gastric epithelial cells [204]. Formation of DNA adducts was reported in upper gastrointestinal tract of mice that were fed bracken fern [205,206] and in target tissue of carcinogenicity, ileum, in rats injected with ptaquiloside intravenously [207]. DNA adducts formed after exposure to bracken fern were distinctly different from the adducts formed by ptaquiloside [206].

*Biotransformation*: Bioactivation of ptaquiloside is not enzyme mediated, and involves conversion to aglycone, ptaquilosin, which, under alkaline conditions undergoes aromatization resulting in a reactive metabolite, bracken dienone [185]. Dienone has an ability to alkylate DNA, forming adducts primarily on *N*3 position of adenine and *N*7 position of guanine [185].

*MoA:* DNA alkylation of adenine bases with subsequent DNA depurination and breakage leading to induction of mutations, in particular to activation of *H-ras* proto-oncogenes and frameshift mutations of *p53* gene [192,204,208,209] is thought to be the main mechanism underlying ptaquiloside-related carcinogenicity. Other potential MoAs, including clastogenicity and aneugenicity, as well as alteration of monocyte function, TNFα expression and cell proliferation, cannot be excluded [202,207,210].

*Human Exposure:* Estimation of human consumption of ptaquiloside with cow’s milk resulted in intake levels ranging from 1.75 to 13.4 mg/day [211]. Some populations in Japan, Brazil and Canada can also consume cooked or salted bracken crosiers [185,186].

*Human Effects*: No study on human carcinogenicity of ptaquiloside is available. However, in areas where bracken fern is consumed, there is a correlation between the consumption of ptaquiloside-contaminated milk and increased risk of esophageal or stomach cancer [184,212,213].

*Risk*: While IARC recognizes bracken fern as possibly carcinogenic to humans (Group 2B), it considers ptaquiloside to be unclassifiable as to its carcinogenicity (Group 3) (Table 2) based on limited evidence [193]. Nevertheless, genotoxicity and mutagenicity of ptaquiloside, as well as some epidemiological evidence of potential carcinogenicity raises concerns for human safety [184,190,202].

#### 3.1.4. Psoralens

*Occurrence*: *Psoralen* (7H-furo[3,2-g][1]benzopyran-7-one) (Figure 1(12)) is a furocoumarin which is naturally present in several plants, notably *Psoralea corylifolia*, celery, parsley and in all citrus fruits, including bergamot orange peel, whose oils are used as flavors [214,215,216,217]. In citrus-flavored beverages, the highest levels of psoralens, 29 and 24 mg/L, were found in bergamot juice and home-made limoncello, respectively [218]. Levels of psoralens in celery varied, depending on when it was harvested, from 26 to 84 μg/g fresh weight [216]. Psoralens are widely used in the photochemotherapy of various skin conditions in humans [219].

*Carcinogenicity*: Psoralen derivatives, 5- and 8-methoxypsoralen (methoxsalen) (Figure 1(13,14)), produced skin tumors in mice in the presence of UV A light, even with oral administration [15,68,193,220,221]. In male rats, tumors of Zymbal glands and kidneys were also reported after oral gavage with methoxsalen [222].

*Genotoxicity/DNA Binding (Adducts)*: Psoralen can be photoactivated to DNA cross linking reactant which exhibit genotoxicity and photomutagenicity [15,193,222,223,224]. Intercalation occurs predominantly on pyrimidine bases of DNA, mainly with thymine, which leads to inhibition of DNA synthesis, in addition, psoralens have high affinity for uridine bases on RNA [219].

*Biotransformation*: Metabolism of psoralen involves hydroxylation of phenyl ring, hydrogenation and hydrolysis of the unsaturated lactone ester, and oxidation of the furan ring to generate epoxide or/and γ-ketoenal intermediates [225,226]. These reactions are catalyzed by CYP3A4, CYP1A1, CYP1A2 and CYP2B6 [226,227,228,229,230]. Incubation of psoralen with liver microsomes from different species, including humans, dogs, non-human primates and rodents, demonstrated similarity of metabolites produced by humans and dogs, while metabolic capabilities of rat and monkey microsomes were the closest to those of human microsomes [225].

*MoA*: Photochemical genotoxicity and mutagenicity are most likely responsible mechanism of psoralens carcinogenicity [15,68,193]. Other potential MoAs may involve oxidative damage [219].

*Human Exposure:* Dietary exposure to psoralens occurs mainly from either limes, with estimated per capita exposure of 1300 μg/day [231], or grapefruit juice, with dietary exposure re-estimated to be in the range of 548 to 2237 μg/day [232].

*Human Effects:* Human exposures have so far been mainly associated with photodermatitis due to occupational contact [216,233,234] with only one report of phototoxicity following ingestion [235]. Human carcinogenicity studies relating to oral psoralens have only been made with patients receiving photodynamic therapy (PUVA) [15,68,236,237] and no attempt to extrapolate these positive results to normal populations has been undertaken. One study identified an association between high citrus consumption and melanoma [238]. This study, however, did not specifically assess risk from psoralen consumption.

*Risk:* IARC classifies methoxsalen with UV A radiation as a human carcinogen (Group 1) and 5-methoxypsoralen as probable human carcinogen (Group 2A) [15,193] (Table 2). Further investigation to establish potential health risks of dietary intake of psoralens in humans is warranted [219].

#### 3.1.5. Pyrrolizidine Alkaloids

*Occurrence:Pyrrolizidine alkaloids* (PAs) are heterocyclic compounds (Figure 1(15–18)), most of which derive from esters of basic alcohols known as necine bases [239,240,241,242,243]. Close to 500 PAs have been identified [244]. They occur widely in flowering plants, and consequently in honey, and are present in herbal teas from many countries [241,244,245,246].

*Carcinogenicity*: Over 20 PAs are established to be carcinogenic in experimental animals [89,240,247,248,249,250]. Oral administration of lasiocarpine (Figure 1(15)), monocrotaline (Figure 1(16)), riddelliine (Figure 1(17)), and retrorsine (Figure 1(18)) produced tumors primarily in the liver of rats [68,242,251,252]. Other target organs of PA carcinogenicity include lung, kidney, skin, bladder, brain and spinal cord, pancreatic islets and adrenal gland.

*Genotoxicity/DNA Binding (Adducts)*: Many PAs are genotoxic and mutagenic in vivo and in vitro following metabolic activation [240,242,245,248,250,252,253,254,255,256]. Several PAs, including retronecine-type PAs riddelliine [257] and monocrotaline [258], are known to form DNA crosslinking and DNA adducts in vivo [250,253,259]. Levels of DNA adducts was reported to closely correlate with the carcinogenic potency of some PAs [25,250,253,257].

*Biotransformation*: The bioactivation of PAs is mediated by CYPs, in particular, CYP3A4, which catalyze hydroxylation of the necine base, followed by dehydration to form the corresponding dehydropyrrolizidine derivatives [240,245,246,249,260]. The dehydropyrrolizidine derivatives (i.e., pyrrole metabolites) have been reported to be strong alkylating agents and have been linked to tumor initiation [261,262]. Similarities have been observed between metabolic activation of several PAs in vitro by human and rat microsomes [260,263]; however, certain differences in formed metabolites were reported [264,265].

*MoA*: Genotoxicity and acute toxicity of PAs are the most likely mechanisms involved in the carcinogenicity of these compounds [250,253].

*Human Exposure*: In the majority of developed countries, human exposure to PAs, which mainly occurs from consumption of contaminated foods of animal origin, grains and plant-derived foods, including herbs, spices and teas, is low, ranging from 0.035 to 0.214 μg/kg bw/day [240,249,266]. Mean total dietary intakes of PAs were estimated to be 0.019 μg/kg bw/day for children and to 0.026 μg/kg bw/day for adults [246], with the highest dietary exposure, ranging from 0.0013 to 0.26 µg/kg bw/day, resulting from herbal tea consumption, while consumption of honey has been calculated to result in chronic dietary exposure ranging between 0.0001 and 0.027 μg/kg bw/day [240,266]. In Europe, levels of PAs is various foods is limited up to 400 μg/kg for herbal infusions [249].

*Human Effects*: In humans, PAs are known to be teratogenic and to act as abortafacients, and exposure can be potentially lethal [267]. Hepatotoxicity of PAs in humans has been also reported [268]. There is a need for epidemiologic studies on acute and long-term effects of PAs.

*Risk*: IARC [89,247] classified lasiocarpine, monocrotaline and riddelliine as possibly carcinogenic to humans (Group 2B) (Table 2), even though there is no epidemiological evidence to indicate that intake of these substances, even at toxic levels, present a carcinogenic risk [246,269]. Other PAs, namely hydroxysenkirkine, isatidine, jacobine, retrorsine, seneciphylline, senkirkine and symphytine, were not classifiable as to their carcinogenicity to humans (Group 3) (Table 2) [89,247]. EFSA and JECFA concluded that based on calculated MoEs, there is a potential concern for human health, in particular for high-level long-term consumers [240,246,248,270]. Genotoxic and carcinogenic potentials of PAs indicates priority for risk management and warrants effort to continue reduction of PAs content in herbal products [271].

### 3.2. Mycotoxins

Mycotoxins are produced by fungi that can contaminate a variety of crops pre- and post-harvest, and which are associated with several diseases in animals and humans. Mycotoxins cannot be completely eliminated from food by food processing procedures, including thermal processing [272]. Of major concern are the mycotoxins aflatoxins, ochratoxin A and fumonisins [273,274].

#### 3.2.1. Aflatoxins

*Occurrence: Aflatoxins* (AFs) (Figure 2(1–6)) are mycotoxins formed by various strains of the fungus, *Aspergillus flavus*, and are present in contaminated foods, particularly corn and peanuts [68,275,276]. Food levels of AFs are often expressed as total AFs [276], which is useful for monitoring purposes. AFB_1_ has a tetrahydrocyclopenta[c]-furo [3′,2′:4,5]-furo [2,3-h]chromene skeleton with oxygen functionality at positions 1, 4 and 11 (Figure 2(1)).

*Carcinogenicity*: AFB_1_ is the most highly carcinogenic AF [277] and one of the most potent carcinogens [278,279] (Table 2). Oral administration of AFB_1_, including as AFs mixtures, produced sufficient evidence for carcinogenicity in multiple species [275]. Specifically, AFB_1_-induced increases in the incidences of hepatocellular or cholangiocellular carcinomas were observed in rats, hamsters, marmosets, tree shrews, and monkeys; in addition, increase were observed in renal cell carcinomas and colon tumors in rats, lung adenomas in mice as well as osteogenic sarcoma, gallbladder tumors and adenocarcinoma of the pancreas in monkeys [68,275]. AFB_2_ (Figure 2(4)), AFG_1_ (Figure 2(2)), and AFM_1_ (Figure 2(3)) also produced liver tumors in experimental animals, but their potency was significantly lower compared to that of AFB_1_ [68,280]. No evidence for carcinogenicity of AFG_2_ have been reported [275].

*Genotoxicity/DNA Binding (Adducts)*: AFB_1_ is genotoxic in vitro and in vivo, producing mutagenic, aneugenic and clastogenic effects [275,281,282], as well as DNA adducts in multiple species [275,283,284,285,286], with the AFB_1_-*N*7-guanine adduct being assumed to be pro-mutagenic and pro-carcinogenic [278,287,288,289]. The initial AFB_1_-DNA adduct is unstable in vivo; it either depurinates to give an AFB_1_-guanine residue which can be detected in the urine, or forms a more stable ring opened formamidopyrimidine derivative measurable in cellular DNA. AFB_1_-DNA adducts show high correlation with tumor incidence, but no threshold for hepatic DNA adduct formation was reported [25]. AFB_1_ also elicited DNA repair synthesis in cultured human hepatocytes [290] and γH2AX induction in human cell lines derived from hepatoblastoma, renal cell adenocarcinoma, and epithelial colorectal adenocarcinoma [291]. DNA adduct formation has been also reported after AFG_1_ and AFM_1_ exposures [292,293].

*Biotransformation*: The genotoxic and carcinogenic AF, AFB_1_, is metabolically activated predominantly by CYP3A4 oxidation at the 8–9 positions (Figure 2(1)) to form an AFB_1_-8,9-epoxide, which is highly reactive and binds to the *N*7 position of guanine residues in DNA [287,288,289,294]. There is abundant evidence that in humans AFB_1_ is bioactivated by CYP1A2, 2B6, 3A4, 3A5, 3A7 and GSTM1 enzymes [281]. Ramsdell and Eaton [279] reported that mouse and monkey microsomes formed AFB_1_-8,9-epoxide at higher rates compared to rat and human; however, at lower substrate concentrations, conversion to AFB_1_-8,9-epoxide increased with rat and human microsomes, but not with mouse of monkey microsomes. Thus, the authors attributed interspecies differences in carcinogenic potency of AFB_1_ to differences in patterns of epoxide formation. AFG_1_ and AFM_1_, which also have a double bond at the 8,9-position (Figure 2(3,4)), can form epoxides; however, they are less DNA-reactive compared to AFB_1_-8,9-epoxide [281]. Non- or weakly carcinogenic AFs, e.g., AFB_2_, AFG_2_, and AFM_2_, lack the double bond in the 8–9 position (Figure 2(4–6)) [276] and, except in the duck, are not metabolized to detectable levels of AFB_1_ [295]. CYP3A4 and CYP1A2 can also metabolize AFB_1_ to hydroxylated metabolites, AFM_1_ and AFQ_1_. Roebuck and Wogan [296] reported that AFQ_1_ was the principal metabolite produced by monkey, human, and rat liver, whereas duck liver produced mainly chloroform-insoluble derivatives. Monkey, human, and mouse liver also produced AFP_1_, which was not observed in duck and rat. The authors noticed that duck, monkey, and human livers were most active, each metabolizing approximately 80% of available substrate in half an hour. In comparison, activity of rat and mouse livers was lower, each metabolizing from 15 to 20% of substrate. No consistent pattern of metabolism that could explain interspecies differences in susceptibility to AFB_1_ carcinogenicity was detected. Detoxication of AFB_1_ occurs predominantly via conjugation with glutathione (GSH), and extent of this reaction differs among species, with mouse showing the highest and humans having the lowest conjugation rates [281].

*MoA*: Covalent binding of AFB_1_-8,9-epoxide to *N*7 of guanine in DNA is considered to be the primary MoA of AFB_1_ carcinogenicity [275,278,281,289,297]. The adduct is believed to induce mutations of *TP53* gene in humans [275,276]. In addition, AFB_1_ epoxide reacts with serum proteins, including albumin. All have been used as biomarkers to assess AFB_1_ exposure [298,299]. Such studies have led to the clear association of AFB_1_ exposure and hepatocellular carcinoma, particularly in those infected with hepatitis B virus [278,281,300]. This is believed to be due to enhanced liver cell proliferation with hepatitis [300]. A strong correlation of urinary adducts indicative of AFB_1_ exposure, notably AFB_1_-*N*7-guanine, serological markers of hepatitis B infection, and liver cancer risk exists [301]. Induction of oxidative stress, immunomodulation and epigenetic modification also play a role in carcinogenicity of AFB_1_ [278,281].

*Human Exposure*: Overall, exposure to AFB_1_ results from ingestion of foods contaminated with *Aspergillus flavus*. Total EDI to AFs ranges from 0.0001 to 0.049 μg/kg bw/day in developing countries and is generally less than 0.001 μg/kg bw/day in developed countries [276]. In parts of the worlds where *Aspergillus* contamination of food is prevalent, AFB_1_ occurs in such foods at significant levels [278,302]. In the United States, consumption of food contaminated with up to 20 ppb AFB_1_, mainly corn and peanuts, is permitted [303], with the exception of milk, which is required to contain less than 0.5 ppb [304], corresponding to about 30 µg/day for a 70 kg adult. Obviously, high exposures are occurring in parts of the world where crop contaminations are not well controlled and accordingly, the cancer risk is much higher.

*Human Effects:* In humans, exposure to AFs is associated with increased risk of liver cancer, particularly in association with concurrent hepatitis B [68,275,281,299,300].

*Risk*: IARC [275] considers AFs to be carcinogenic to humans (Group 1) (Table 2). JECFA estimated the cancer potency for exposure to AFB_1_ per 100,000 population at 0.001 μg/kg bw/day, and recommended that efforts to reduce aflatoxin exposure continue [276]. The Committee also noticed that AFM_1_ will generally make a negligible (<1%) contribution to aflatoxin-induced cancer risk for the general population. EFSA estimated that MoEs, which range from 5000 to 29 for AFB_1_ and from 100,000 to 508 for AFM_1_ exposures, respectively, raise a concern for human health [281].

#### 3.2.2. Ochratoxin A

*Occurrence: Ochratoxin A* (OTA) (*N*-[(3R)-5-chloro-8-hydroxy-3-methyl-1-oxo-3,4-dihydro-1H-isochromen-7-yl]carbonyl-L-phenylalanine) (Figure 2(7)), is a mycotoxin produced by a single *Penicillium* and several *Aspergillus* fungal species [305,306,307]. The ochratoxins are pentaketides, consisting of a dihydroisocoumarin coupled to 8-phenylalanine (Figure 2(7)) and, unusually for natural products, OTA is chlorinated. OTA is formed in improperly stored foods which have been produced mainly in Europe and Canada, including cereals, beans, ground nuts, oleaginous seeds, meat and wine [306].

*Carcinogenicity*: In several strains of mice, OTA fed in the diet induced kidney neoplasms, including carcinomas, at a concentration of 40 mg/kg bw, and liver neoplasms at 1 mg/kg bw. When administered by gavage to rats it induced renal tumors starting at 70 µg/kg bw, [305,308]. Male rats were considerably more susceptible than females. A feature of the renal toxicity of OTA is formation of karyomegalic nuclei in the tubular epithelia, predominantly in the corticomedullary zone [309].

*Genotoxicity/DNA Binding (Adducts)*: OTA was consistently negative in studies assessing mutagenicity in *Salmonella typhimurium*, both with and without exogenous metabolic activation. In contrast to bacteria, however, overall results from genotoxicity tests in mammalian cell systems provide some evidence for a weak genotoxic activity of OTA [305,307,310,311,312]. It elicited DNA repair synthesis in cultured rat and mouse hepatocytes at cytotoxin doses, increased DNA strand breaks levels and mutagenicity in target tissue, kidney, in rodents [307,312]. Controversy exits over whether OTA reacts directly with DNA. OTA did not form DNA adducts in the kidneys of male rats when measured using radiolabeled OTA and accelerator mass spectrometry [313], while others obtained positive results in isolated DNA and cell culture by dehalogenation and redox reactions analyzed by nucleotide ^32^P-postlabeling (NPL) [314]. Mantle et al. [315] was able to identify a small amount (20–70 adducts per 10^9^ normal nucleotides) of a single DNA adduct in the kidneys of rats using refined NPL methodology. These conflicting data have been reviewed [306,310,312]. EFSA concluded that while formation of covalent OTA-DNA adducts remains controversial, OTA mutagenicity cannot not simply be a consequence of oxidative DNA damage [307].

*Biotransformation*: OTA is characterized by rapid absorption and distribution, but slow elimination and excretion [307]. The major metabolite of OTA forms as a result of hydrolyses of amide bond between phenylalanine and dihydroisocoumaric acid. OTA also undergoes oxidative dichlorination in the presence of CYPs, generating electrophilic quinone, which can be further reduced to hydroquinone metabolite and excreted in urine, as has been shown in rats and humans [307,312]. In addition, peroxidase enzymes are involved in oxidation of OTA to electrophilic phenolic radical, which is believed to cause oxidative stress. Radical and benzoquinone intermediates formed during metabolism of OTA can covalently bind to DNA, generating C-bound C8-dG adducts [306].

*MoA:* The definitive MoA for carcinogenicity of OTA remains unclear, and most likely involves a combination of mutagenicity and increased reactive oxygen species (ROS) level leading to oxidative DNA damage [306,310,312,316,317]. Alternatively, an epigenetic MoA for renal carcinogenicity has been postulated to be a combination of inhibition of histone acetyltransferase, producing mitotic disruption leading to increased cell proliferation and genetic instability [310]. The mitotic disruption may be the basis for karyomegaly observed in rodent kidneys [318]. Thus, MoA other than DNA reactivity are possible for OTA. The pathogenesis of the renal tumors in male rats probably does not involve an α2u-globulin MoA [319]. Moreover, sex and strain differences are suggestive of biotransformation being important [320].

*Human Exposure:* In European Union, dietary exposures range between 0.00064 to 0.0178 μg/kg bw/day across all age groups [307]. The EDI values for OTA calculated from food products range from 1 × 10^−7^ to 0.0252 μg/kg bw/day [306]. In areas where contamination occurs, biomarkers of OTA exposure are measurable in human blood, urine and milk [306].

*Human Effects:* OTA is suspected to be the main etiologic agent for human Balkan endemic nephropathy and the associated urinary tract tumors [311,321].

*Risk:* IARC [305] classified OTA as possibly carcinogenic to humans (Group 2B) based on sufficient evidence for carcinogenicity in experimental animals (Table 2). JECFA concluded that maximum levels of at 5 or 20 µg/kg in contaminated cereal grains would be unlikely to have an impact on dietary exposure to OTA, and established a provisional tolerable weekly intake of 0.112 μg/kg bw [322]. EFSA estimated that MoE for chronic neoplastic effects ranged from 22,615 to 815, indicating possible health concern for high-level consumers and breastfed infants [307].

### 3.3. Carcinogens Formed during Food Processing

While many carcinogens associated with food processing are generated during heating (see next section), some can be formed through nonthermal process or during storage [5]. Such chemicals include benzene, cholopropanols and ethyl carbamate.

#### 3.3.1. Benzene

*Occurrence: Benzene* (BZ) (Figure 3(1)) is present at low levels in a wide variety of foods [323], in particular processed products, as well as in drinking water and soft beverages [324,325]. Highest concentrations of BZ, up to 2100 ppb, have been reported in eggs, haddock, beef and butter [326]. It is formed from a reaction between sodium or potassium benzoate and ascorbic acid, which are often used as food preservatives [327], this can result in BZ concentrations ranging from 0.001 to 0.038 μg/g in some products such as eggs [323]. In addition, BZ can be introduced to foods from packaging materials [328]. In soft beverages BZ is present in small amounts, below 5 μg/kg [329,330].

*Carcinogenicity*: BZ produced leukemia, and other neoplasms, in rats and in mice with inhalation exposure [68,324,331,332]. With oral administration, BZ at doses up to 200 mg/kg bw is reported to produce oral cavity and skin tumors in rats, Zymbal gland carcinoma in rats and mice, malignant lymphoma, lung cancer, preputial gland carcinoma and cancer of the mammary gland in mice [324,333].

*Genotoxicity/DNA Binding (Adducts):* The genotoxicity data are mixed, although, DNA damage and chromosomal aberrations were often reported in animals and occupationally exposed humans [324,331,334,335,336,337,338,339]. In addition, BZ metabolites form DNA adducts in vitro, in mice and human hematopoietic cells [324,340,341,342,343,344]. Importantly, hydroquinone (HQ), a major metabolite of BZ and a food component, does not form DNA adducts in vivo [343,345,346]. *p*-Benzoquinone is a possible candidate for the genotoxic metabolite of BZ [347,348,349], although other metabolites have been considered, including indirect mechanisms involving oxidative DNA damage [324,331,334,350].

*Biotransformation*: BZ is biotransformed by CYPs, mainly CYP2E1, to benzene oxide, which is further metabolized by various pathways to phenol, HQ, catechol and *trans*,*trans*-muconic acid in experimental animals and humans [324,331,351].

*MoA*: The exact molecular mechanism which BZ exerts its carcinogenicity remains to be elucidated, especially by the oral route. Oxidative DNA damage, genotoxicity, aneugenicity and clastogenicity, as well as interference with cell cycle and immunosuppression may be involved [324,334,336,350].

*Human Exposure*: Predominant exposure to BZ in general population occurs through air, rather than foods [68,324,326,352,353]. Similarly, while detectable BZ levels are present in human milk, infant exposure occurs predominantly from the air [354]. A dietary exposure to BZ through various sources was estimated to be in the range of 0.003 to 0.05 μg/kg bw/day [326,355]. In Canada, intake level of BZ from food and water was estimated at approximately 10 μg/day [356]. Currently, no limits for BZ are established in foods and beverages, while in water it ranges from 1 to 10 ppb in different countries [325].

*Human Effects*: In humans, BZ is associated with increased risk of leukemia, myelodysplastic syndrome and other hematopoietic disorders with airborne occupational exposures [324,351,357]. Some studies also report association between BZ exposure in occupational settings and cancers in other organs, including respiratory, gastrointestinal, urinary, central nervous systems and skin [324]. No data on carcinogenicity via food exposure are available [326].

*Risk*: BZ is recognized by IARC [324] as carcinogenic to humans (Group 1) (Table 2). JECFA concluded that based on known effects, BZ in not suitable for use as an additive in food [358]. Using probabilistic modelling, Cheasley et al. [356] estimated that lifetime excess cancer risk associated with BZ dietary intake was 35 per million. Nevertheless, MoEs calculated based on the estimated dietary intake did not indicate human risk from dietary exposures [325,326,355,359], however more studies are warranted.

#### 3.3.2. Chloropropanols

*Occurrence*: *3-Chloro-1,2-propanediol* (CP) (Figure 3(2)) and *1,3-dichloro-2-propanol* (DP) (Figure 3(3)) are formed during the acid hydrolysis of vegetable proteins through the reaction of chloride ions with triglycerides [115,360,361,362]. Several are present at low levels ranging from 9.6–82.7 µg/kg, in various foods, most notably refined oils, acid-hydrolyzed proteins and soy sauces; however, some sauces contain as high as 18 mg/kg or 876 ppm [115,276,361,363,364,365]. Chloropropanols can be also found in paper-based food contact materials [366,367].

*Carcinogenicity*: In a two-year bioassay in rats, CP produced increases in kidney, Leydig cell, and mammary neoplasms administered at doses up to 400 ppm (29.5 mg/kg bw/day) in drinking water [115,276]. DP produced increases in neoplasms in the liver, kidney and tongue in rats at doses up to 30 mg/kg bw/day [115,365,368,369].

*Genotoxicity/DNA Binding (Adducts):* CP was genotoxic in some in vitro assays, but not in vivo [115,370,371]. In contrast, DP was clearly genotoxic in vivo and in vitro, with or without metabolic activation [115,276,361,365,368]; however, formation of DNA adducts has not been reported. Genotoxicity of DP was attributed to formation of epoxide intermediate [368].

*Biotransformation*: CP is metabolized by alcohol dehydrogenase to chlorolactic acid, while DP is metabolized by CYP2E1, to cytotoxic metabolites, including 1,3-dichloroacetone [115,361].

*MoA*: No clear MoA has been established for carcinogenicity of CP and DP. There is evidence that CP induces tumors by a hormonally mediated and/or cytotoxic MoA [362,372]. Oxidative damage has been also implicated [373]. Nevertheless, genotoxic MoA cannot be excluded for CP and DP [115].

*Human Exposure*: Mean EDI for CP was calculated to range from 0.2 to 3.8 µg/kg bw/day in adults and to be 1.3 µg/kg bw/day in children [276,362]. Mean EDI for DP was estimated to be 7 µg/person/day from soy sauce consumption, and 0.1 µg/person/day from dietary sources other than soy sauce [365,369].

*Human Effects*: No adequate data are currently available to assess the potential carcinogenicity of the chloropropanols in humans [115].

*Risk*: IARC [115] classifies CP and DP as possibly carcinogenic to humans (Group 2B) (Table 2). JECFA [276] set a provisional maximum tolerable daily intake (PMTDI) of 4 µg/kg bw/day for CP, while EFSA [362] established a much lower TDI of 2 µg/kg bw/day. JECFA concluded that no TDI can be estimated for DP based on its effects [365,369]; however, based on calculated MoE, DP in diet was considered to be of low concern for human health [361].

#### 3.3.3. Ethyl Carbamate (Urethane)

*Occurrence*: *Ethyl Carbamate* (EC) (Figure 3(4)), also referred to as urethane, is a fermentation product formed from the reaction of ethanol and carbamyl phosphate [374,375]. It is present as a natural trace constituent in various alcoholic beverages and in fermented food items, including cheese, bread, yogurt, soy sauce and fermented soybean products [374,376,377,378,379]. Mean concentrations of EC in some spirits, particularly in stone-fruit brandies, have been measured in a range of 4 to 122 µg/kg (or 0.1 to 1400 µg/L), while in foods lower concentrations, ranging from 0.2 to 16 µg/kg, were observed [374,376,377,378]. EC content in foods can also increase with thermal processing [380].

*Carcinogenicity*: With oral administration to mice, EC up to 600 ppm induced mainly liver, lung, harderian gland, skin, mammary gland, ovaries, blood vessels and forestomach neoplasms [377,381,382]. In rats, oral administration of EC resulted in an increased incidence of Zymbal and mammary gland carcinomas [68,377].

*Genotoxicity/DNA Binding (Adducts)*: Genotoxicity and clastogenicity of EC has been demonstrated in vitro and in vivo [377,382,383,384]. The formation of etheno DNA adducts was reported in the liver [385], lung [386] and other organs [387] in rats and mice.

*Biotransformation*: EC is metabolized predominantly by CYP2E1 to reactive metabolites, vinyl carbamate and vinyl carbamate epoxide [377,382,388,389,390]. Formation of vinyl carbamate was also reported after incubation of human liver and lung microsomes with EC [391,392], suggesting similarities in metabolism of EC between humans and rodents.

*MoA*: Formation of reactive metabolite and consequent transition mutations in *Kras* oncogene is considered as major mechanism involved in tumorigenesis of EC [377,388,393]. Other potential MoAs may involve proinflammatory signaling, mitochondrial dysfunction and ROS formation [376].

*Human Exposure*: Under normal dietary habits, excluding alcoholic beverages, the EDI was in the range of 0.01 to 0.02 µg/kg bw/day, or an average of 0.015 µg/kg bw/day [378,394]. Mean EC intake from diet and alcoholic beverages rages from 0.015 to 0.065 µg/kg bw/day [374].

*Human Effects*: No epidemiologic studies are currently available to assess hazardous effects of EC in humans [377,378].

*Risk:* IARC [377] evaluated EC as probably carcinogenic to humans (Group 2A) (Table 2). JECFA [378] and EFSA [379] concluded that exposure to EC in food, excluding alcoholic beverages, poses low concern for human health. Nevertheless, health concern exists for consumers of alcoholic beverages, and mitigation measures should the implemented to reduce levels of EC in certain spirits. Schlatter and Lutz [395] calculated a virtually safe dose for EC of 0.02 to 0.08 µg/kg bw/day, which represents negligible risk to human health. Nevertheless, consumption of alcoholic beverages, in particular stone-fruit distillates, increases cancer risk to approximately 0.01%.

### 3.4. Heat-Generated Carcinogens

Heating and combustion of organic materials (e.g., tobacco) is well established to generate carcinogens. With respect to foods, three major types are acrylamide, heterocyclic amines and polycyclic aromatic hydrocarbons [396].

#### 3.4.1. Acrylamide

*Occurrence:Acrylamide* (AC) (propen-2-amide) is an unsaturated amide (Figure 4(1)), which is formed in heated foods, especially those rich in carbohydrates, by a reaction of reducing sugars with asparagine [397,398,399] and consequently is present in a variety of food products, notably baked or fried foods [68,400,401,402,403]. Efforts to reduce AC formation in foods have been active. For example, asparaginase has been proposed for use in food manufacture to convert asparagine to aspartic acid, thereby depleting one of the precursors of AC formation [403,404,405].

*Carcinogenicity:* AC was tested for carcinogenicity in rats by oral administration. In males, it increased the incidences of peritoneal mesotheliomas found in the region of the testis and of follicular adenomas of the thyroid gland. In females, thyroid follicular tumors, mammary gland tumors, glial tumors of the central nervous system, oral cavity papillomas, uterine adenocarcinomas and clitoral gland adenomas were increased. In four screening bioassays in mice, AC, given either orally or intraperitoneally, increased both the incidence and multiplicity of lung tumors in all experiments [402,406,407]. In a two-year rodent carcinogenicity bioassay [408], AC produced clear evidence of carcinogenic activity in rats and mice. Specifically, administration of up to 50 ppm AC in drinking water resulted in increased incidences of thyroid gland and heart tumors in rats of both sexes, of malignant mesotheliomas and cancer in the pancreatic islets in male rats and of cancers in the clitoral gland, liver, mammary gland, skin, and mouth or tongue in female rats. Increased incidences of cancer in the harderian gland, lung, and stomach were observed in male and female mice, in addition, female mice also had increased incidences of cancer in the mammary gland, skin, and ovary.

*Genotoxicity/DNA Binding (Adducts)*: AC is genotoxic and forms DNA adducts in target as well as non-target tissues of carcinogenicity, including the liver, lung, kidney, leucocytes and, testes in mice and in the liver, brain, thyroid, leukocytes and testes or mammary gland in rats [37,409]. In a dose–response study, a 0.1 μg/kg bw was established as a NOAEL for DNA adduct formation [410], suggesting plausibility of thresholds for carcinogenicity. In rats dosed with AC at 2 or 15 mg/kg bw for up to 28 days, DNA synthesis was increased in target tissues, but not in a non-target tissue, the liver [411]. In mice, administration of AC at 7.5, 15 and 30 mg/kg bw/day by gavage for 28 days produced a significant increase in micronuclei formation in the peripheral blood and an increase in *gpt* mutation frequencies in testes and lungs [412]. In addition, AC induced DNA strand breaks in various tissues in rats and mice [413,414,415].

*Biotransformation*: AC has two potentially reactive sites, a conjugated double bond and an amide group (Figure 4(1)) [399]. In vivo, AC is epoxidized at its double bond to glycidamide (GA) [416]. Both, AC and GA, are reactive and while there is some evidence for genotoxicity of AC [417], GA appears to be the DNA-reactive metabolite of AC [418]. GA reacts readily with DNA [407,419,420], forming purine adducts [410,421]. In rats, following administration of AC at 3 mg/kg bw, the majority of metabolites excreted in urine were AC-GSH conjugates, while a substantial proportion of the remainder consisted of two GA-derived mercapturic acids [422]. GA and dihydroxypropionamide were not detected at this dose level. The metabolism of AC in humans was investigated in a study in which male volunteers were administered 3 mg/kg AC orally. At 24 h, urine contained a third of the administered dose, and the majority of metabolites was derived from direct conjugation of AC with GSH [422]. GA, dihydroxypropionamide and one unidentified metabolite were also detected in urine. This study indicated both similarities and differences in the metabolism of AC between humans and rodents.

*MoA*: The carcinogenicity of AC may result from a combination of DNA reactivity and increased cell proliferation in target tissues. However, non-genotoxic MoA, such as alterations of calcium signaling, might be more relevant for tumorigenicity of AC, since evidence of its genotoxicity are weak [25]. In addition, some studies provide evidence of oxidative DNA damage by AC, as well as epigenetic modifications which might be involved in tumorigenesis [423,424,425]. Several possible MoAs have been reviewed [409].

*Human Exposure:* Dietary exposure to AC results from consumption of foods preparation of which involves cooking at high temperatures (e.g., French fries and potato chips), other exposure routes may involve dermal contact and inhalation. EDI of AC in several European populations was estimated to range from 0.4 to 1.9 µg/kg bw/day [425], with average dietary intake of 0.5 µg/kg bw/day in adults worldwide [426].

*Human Effects:* Numerous epidemiologic studies have examined the relationship of dietary consumption of AC and risk for cancers of the kidney, large bowel, urinary bladder, oral cavity and pharynx, esophagus, larynx, breast, and ovary [406,427]. No evidence of any association was found. Individual susceptibility, however, may be related to genetic polymorphisms in enzymes involved in activation and detoxification of AC [428]. Two cohort mortality studies were conducted among workers exposed to AC. The first showed no significant excess of cancer but was of small size, short duration of exposure and short latency. In the other study, in one plant in the Netherlands and three in the US, a nonsignificant increase was found in deaths from pancreatic cancer, but there was no trend with increasing exposure. In a prospective study, increased risks were found for postmenopausal endometrial and ovarian cancer with increasing dietary AC intake, particularly among never-smokers. Risk of breast cancer was not associated with AC intake [429].

*Risk:* Based on sufficient evidence for carcinogenicity in experimental animals and inadequate evidence in humans, IARC [402] classified AC as “probably carcinogenic to humans” (Group 2A) (Table 2). JECFA [403] concluded that estimated MoE for cancer events of 310 for general population and 78 for population with high exposure indicates a human health concern. EFSA [425] also concluded that although the epidemiological studies have not demonstrated AC to be a human carcinogen, MoE indicates a concern for neoplastic effects based on animal evidence. However, based on the dietary intakes [400,425], exposures to AC are mainly at or below those considered acceptable for AFB_1_, which is a more potent carcinogen in animal models (Table 2). It would therefore seem unlikely that a significant risk exists for the general population. Furthermore, an expert panel convened by the German Federal Agency of Consumer Health Protection and Veterinary Medicine opined that while AC was a genotoxic carcinogen, it was likely to show a non-linear dose–response curve with respect to carcinogenic effect [430]. In support of this, Baum et al. [431] have shown that at concentrations added to human blood which are comparable to those achieved by intake from food, AC preferentially reacts with protein components of blood, and is ‘quenched’ without affecting DNA in lymphocytes.

#### 3.4.2. Heterocyclic Amines

*Occurrence:Heterocyclic Amines* (HCAs) are generated in meats cooked at high temperatures which produce protein decomposition [4,305,396,432]. A variety of different HCAs have been identified, representing several structural types reflecting the chemistry of their formation. The major subclass of HCAs, aminoimidazoazaarenes, which is the most abundant in food, includes *2-amino-3-methylimidazo [4,5-f]quinoline* (IQ) (Figure 4(2)), *2-amino-3,4-dimethylimidazo [4,5-f]quinoline* (MeIQ) (Figure 4(3)), *2-amino-3,8-dimethylimidazo [4,5-f]quinoline* (MelQx) (Figure 4(4)), *2-amino-3,4,8-trimethylimidazo[4,5-f]quinoline* (diMeIQx) (Figure 4(5)), and *2-amino-1-methyl-6-phenylimidazo [4,5-b]pyridine* (PhIP) (Figure 4(6)) [433].

*Carcinogenicity:* HCAs are potent multisite carcinogens in several species [68,305]. Specifically, oral administration of IQ, MeIQ and MeIQx to rats or mice caused increases in the incidences of tumors in the liver, small and large intestine, forestomach, lung, Zymbal gland, skin, mammary and clitoral glands, as well as lymphomas and leukemias. In the case of MeIQx, hepatic neoplastic lesions were observed only at high doses, indicating possibility of thresholds [45]. In rats, carcinogenicity of IQ in several target tissues was potentiated with high fat diet [434]. IQ was also shown to induce hepatocellular carcinomas in cynomolgus monkeys after chronic dosage of 10 or 20 mg/kg for 5 days/week. PhIP administered orally caused lymphoma in male rats and in mice of both sexes. Moreover, several cancers associated with the Western diet, specifically carcinoma of prostate gland, adenocarcinoma of the small intestine and colon and mammary gland carcinoma were observed in rats orally exposed to PhIP. Neonatal mice are also extremely sensitive to carcinogenic HCAs [68,305]. The order of carcinogenic potencies of selected HCAs is as follows: IQ > MeIQ > MeIQx > PhIP (Table 2).

*Genotoxicity/DNA Binding (Adducts)*: HCAs are potent genotoxic mutagens in various systems in vitro and in vivo [68,305,435,436], including human cells [437]. IQ, MeIQ, MeIQx and PhIP were shown to induce DNA damage and chromosomal aberrations, SCE, micronucleus formation and UDS. HCAs have been shown to form DNA and protein adducts in vitro and in vivo in various species, including humans [305,438,439,440,441,442,443]. Data from studies investigating formation of PhIP DNA adducts reported that in human tissues at dietarily relevant exposures DNA adducts form with greater efficiency compared to rodents [436]. There was a liner correlation between the dose and DNA-binding of some HCAs in the liver [25].

*Biotransformation*: The bioactivation of HCAs involves mainly *N*-hydroxylation, usually by CYP1A2 [444], and subsequent acetylation by *N*-acetyltransferase type 2 [445], leading to formation of a reactive nitrenium ion, as with other aromatic amines. Nitrenium ion primarily binds to C8 atom of guanine bases [432,436]. Genetic polymorphism of these enzymes in humans might play a role in susceptibility to genotoxicity and carcinogenicity of HCAs [446]. For example, high levels of DNA damage were observed in cell cultures with rapid acetylation [447] and individuals with rapid acetylator phenotype are believed to have higher risk of certain cancers after exposure to HCAs compared to slow-acetylators [432,445].

*MoA:* Carcinogenicity of HCA most likely results from formation of DNA adducts which lead to mutations in proto-oncogenes and tumor-suppressor genes, including *K-ras*, *Ha-ras*, *Apc*, *β-catenin*, and *TP53* [68,432].

*Human Exposure:* Human intake of HCAs is estimated to range from 0.001 to 0.017 μg/kg bw/day [448] with some intakes as high as 1900 ng [449]. The average lifetime time-weighted consumption of HCAs for US population is estimated to be approximately 0.009 μg/kg/day, with PhIP comprising two thirds of the intake [450].

*Human Effects:* HCAs are reasonably anticipated to be human carcinogens [68,432]. They have been implicated in causing cancers of the breast [451], colon and rectum [449], stomach and esophagus [452], and lung [453]. Estimated consumption by humans of at least one HCA, PhIP, was associated with increased levels of DNA adducts in breast [454] and prostate tissues [455] of patients with cancers at those sites. While consumption of cooked or grilled meat has been associated with various types of cancers, the data do not definitively implicate HCAs as the causative component of these associations [449].

*Risk:* IARC [305] classifies HCAs as either possible (Group 2A) (e.g., IQ) or probable (Group 2B) (e.g., MeIQ, MeIQx, PhIP) human carcinogens (Table 2). An upper-bound risk for US population from dietary exposures to HCAs was estimated to be 28,000 cancers, with PhIP accounting for almost half (46%) of the total risk [448]. The consumption of cooked meat and fish was the primary contributor to cancer risk in humans. Nevertheless, currently no regulations targeting reduction of exposure to HCAs exist [68].

#### 3.4.3. Polycyclic Aromatic Hydrocarbons

*Occurrence:Polycyclic Aromatic Hydrocarbons* (PAHs) is a group of compounds composed of two or more fused aromatic rings, which are present in many foods, either from deposition from air pollution or formed during cooking processes such as with char broiling of meats [275,378,456,457]. Heating of food above 350–400 °C leads to formation of PAHs, notably, the prototypical PAH, *benzo[a]pyrene* (BaP) (Figure 4(7)). BaP is found in smoked foods, charcoal-broiled steaks and ground meats [458,459,460]. The highest levels of BaP are found in grilled meats, at up to 4 ng BaP/g of cooked meat, [460].

*Carcinogenicity:* A variety of PAHs produced sufficient evidence of carcinogenicity in experimental animals [68,461]. In particular, BaP produced tumors in multiple species, including mouse, rat, hamster, guinea pig, rabbit, duck, newt, and monkey, following exposure by many different routes [68,275]. When administered orally, either via gavage or with diet, BaP at dosages up to 14 mg/kg bw/day increased incidences of tumors in lymphoid and haematopoeitic systems and in several organs in mice, including the lung, forestomach, liver, oesophagus and tongue [68,275,378]. Administration of BaP to rats by gavage for two years, produced liver tumors and tumors of forestomach at the lowest dose of 10 mg/kg bw and higher [462]. PAH mixtures, in particular creosote oils, coal-tar pitches, shale oils, anthracene oils, and certain bitumens have been shown to induce skin tumors in mice upon topical application [461,463].

*Genotoxicity/DNA Binding (Adducts):* PAHs, including BaP, are mutagenic and genotoxic in a variety of test systems, both in vitro and in vivo [47,275,378]. Reactive metabolites of PAHs can covalently bind to DNA, predominantly at the *N*2 position of desoxyguanosine [378]. A linear correlation between DNA adduct formation and mutagenicity was reported, providing evidence against the existence of thresholds for BaP effects [25].

*Biotransformation*: BaP and other PAHs with appropriate structures are bioactivated to oxides and dihydrodiols, which in turn are oxidized to diol epoxides, in a multi-step, inducible pathway involving CYP and epoxide hydrolase microsomal enzyme systems [464,465]. The dihydrodiol epoxide intermediate(s) (e.g., benzo(a)pyrene-7,8-dihydrodiol-9,10-epoxide (Figure 4(8))) form stable and depurinating DNA adducts, which are primarily responsible for the mutagenic and carcinogenic action of BaP and other PAHs [47,275]. PAHs lacking the structural basis for formation of epoxides which can open and generate relatively stable carbonium ions with appropriate conformations of their hydroxyl groups are at most weakly carcinogenic [466]. For PAHs with lower ionization potential, the one-electron oxidation pathway, which results in the formation of unstable DNA adducts, might also be important [47].

*MoA:* The formation of DNA adducts by reactive metabolites, oxides and diol epoxides, is considered to be an initiating event in the development of tumors caused by BaP and some other PAHs [47,68]. These adducts were shown to induce mutations in oncogenes and tumor suppressor genes, such as *K-ras* and *TP53,* in humans and rodents [275]. However, due to poor quantitative relationship between levels of DNA adduct in target tissues and tumor formation, other factors involved in MoA should be considered. For example, induction of oxidative stress [467], immunosuppression [468], alterations of cell cycle [469] and epigenetic modifications [470] might also contribute to carcinogenic effects of PAHs.

*Human Exposure:* Food contaminated with PAHs, either from environmental sources or during processing and cooking, is the major source of exposure in non-smokers [47,457]. JECFA [378] reported EDI for BaP to be in a range of 0.0006 to 2.04 μg/kg bw/day, and for other PAHs EDI varies from 0.0001 to 0.015 μg/kg bw/day. Intake of PAHs in children is approximately double of the intake in adults [378]. EFSA [47] identified cereals and cereal products as well as seafood as the two highest contributors to the dietary exposure to PAHs. The European Union legislation (Regulations EC No. 835/2011 and No. 2020/1255) provides specific regulations for maximum levels of PAHs in various foods, which, depending on the product, ranges from 1 to 10 μg/kg for BaP and from 1 to 50 μg/kg for all PAHs [457,471,472]. In US, no maximum limits for PAHs in foods has been established, with exception of maximum permissible level of BaP in bottled water of 0.0002 mg/L [68,457].

*Human Effects:* No epidemiological studies on association between exposure to the individual PAHs and human cancers have been conducted, and data on the carcinogenic effects of PAHs in humans is available only for mixtures [68,275,461,463]. Thus, studies of smokers and consumers of certain meat products uncovered evidence of the carcinogenicity of BaP and other PAHs in humans. Lung cancer has been shown to be induced in humans by cigarette smoke, and by exposures to roofing tar and coke oven emissions, all of which contain mixtures of PAH [68]. A recent case control study reported an association between exposure to BaP in the diet and an increased risk for colorectal adenoma [473].

*Risk*: IARC [275] concluded that BaP is a human carcinogen (Group 1) based on the biological plausibility of mechanism of carcinogenicity in humans (Table 2). In a study of human intake in Korea, a possible excess cancer risk ascribed to PAHs using the cancer potency of BaP was calculated to be 2.3 cases per 100,000 persons [474]. Based on the MoE of 25,000 (mean) and 10,000 (high), and a human exposure estimate of 0.004 (mean) to 0.01 (high) μg/kg bw/day, JECFA [378] concluded that the estimated oral intakes of PAHs were of low concern for human health. Similarly, EFSA [47] established that MoE of 17,900 for BaP is indicative of low concern for consumer health at the average estimated dietary exposures; however, for high-level consumers potential concern exists.

### 3.5. Carcinogens Formed Exogenously and Endogenously

Consumer exposure to carcinogens is multifactorial and, in some cases, in addition to exogenous sources might involve endogenous formation of hazardous chemicals as a result of metabolic reactions [6]. Such endogenous exposures can significantly contribute to total exposures and complicate risk assessment.

#### 3.5.1. Ethylene Oxide

*Occurrence*: *Ethylene oxide* (EtO) (Figure 5(1)) is primarily used as an intermediate in the production of ethylene glycol [275]. However, in some countries, including USA, Canada and India, EtO has been also used as a fumigant for its disinfectant properties, and hence it may be present as a residue on contaminated foods including spices, nuts, sesame seeds, dry fruits and vegetables, milk powder and cereal, at various concentrations exceeding 0.05 mg/kg and even reaching 1800 mg/kg in some herbal teas [475,476,477]. Due to known hazardous effects, use of EtO in food production in Europe is prohibited (Regulation (EC) No 1107/2009) [478], and maximum residue levels are established between 0.02 and 0.1 mg/kg (Regulation (EC) No 396/2005) [479]. Such regulations led to a recent recall of a variety of products containing EtO contaminated sesame seeds or locust bean gum, including bread, sauces, ice cream and other fermented milk products [480,481]. In addition to exogenous sources, EtO can be also formed endogenously as a result of lipid peroxidation reactions, metabolic activity of microbiota or following metabolism of ethylene [482,483,484]. Endogenous levels of EtO in humans were estimated to range from 0.13 to 6.9 ppb [485].

*Carcinogenicity*: EtO is a multisite carcinogen in rodents, with target organs including the hematopoietic system in mice and rats, the lung, Harderian gland, mammary gland, and uterus in mice, and the brain and mesothelium in rats [68,275,486,487,488,489]. Oral administration of up to 30.5 mg/kg bw of EtO through gavage twice a week produced an increase in the forestomach squamous cell carcinoma in rats [490].

*Genotoxicity/DNA Binding (Adducts)*: EtO exhibited genotoxic and mutagenic effects, albeit weak, in experimental systems in vitro and in vivo, moreover, cytogenic alterations and DNA damage, including chromosomal aberrations, SCE, *hprt* mutations, micronucleus formation, and DNA single-strand breaks were reported in peripheral blood of humans with occupational exposures [275,487,488,489,491,492,493,494,495]. As a direct alkylating agent, EtO covalently binds to DNA, predominantly at the *N*7 position of guanine [487,496,497,498,499].

*Biotransformation*: EtO can be either hydrolyzed, spontaneously or in the presence of microsomal epoxide hydrolase, to ethylene glycol with subsequent conversion to oxalic acid, formic acid and carbon dioxide, or conjugate with GSH to form mercapturic and thiodiacetic acids [275,488,500]. Several studies implied that polymorphism in human GST genotype, in particular GSTT1, might underly the difference in susceptibility to adverse effects of EtO [501,502,503,504].

*MoA*: While carcinogenicity of EtO is attributed to its genotoxicity and mutagenicity [275,489], formation of *N*7-guanine DNA adducts are not likely to contribute to the carcinogenic MoA. These adducts are not pro-mutagenic and are steadily repaired not leading to accumulation of abasic sites [497,505]. Hence, mutagenicity of EtO was attributed to minor adducts, *N*3-2-hydroxyethyladenine and O^6^-2-hydroxyethylguanine [506]. Due to high repair rate of DNA adducts formed by alkylating agents, existence of thresholds for EtO genotoxicity it plausible [489,507]. Several studies also attempted to use dose–response data for genotoxicity endpoints to estimate safe exposure levels to EtO [508,509].

*Human Exposure*: Exposures to EtO occur predominantly through inhalation, while dietary exposures are negligible [275]. Thus, EDI from all food sources amounts to 10 μg/person/day, which is lower than endogenous production of EtO by bacteria (15–20 μg/day) [510]. Average per capita consumption of EtO with spices was estimated to range from 0.21 μg/person/day in New Zealand to 1.6 μg/person/day in US [511].

*Human Effects:* There is no evidence of adverse health effects related to the consumption contaminated foods, mainly due to the rapid breakdown of EtO. While some epidemiological studies report association between occupational exposure (primarily through inhalation) to EtO and higher risk of lympho-haematopoietic [512] and breast cancers [513], the evidence is limited and not supported by meta-analysis studies [68,275,487,489,514,515,516].

*Risk:* Despite only limited evidence of carcinogenicity in humans, IARC [275] classified EtO as human carcinogen (Group 1) (Table 2), based on the mechanistic evidence of its genotoxicity in workers. While the NTP and the US EPA [68,517] reached the same conclusion, systemic analyses of carcinogenicity and toxicity data conducted by several authors suggests that such classification grossly overestimates the risk of EtO [489,516]. One study estimated that cancer risk from consumption of EtO with contaminated spices was negligible [511]. The German Federal Institute for Risk Assessment also established that EtO intake at or below 0.037 μg/kg bw/day should be considered of low concern [518].

#### 3.5.2. *N*-Nitroso Compounds

*Occurrence*: *N-Nitroso compounds* (Figure 5(2,3)) are formed in food and in vivo at acidic pH by nitrosation of secondary and tertiary amino compounds [68,519,520,521]. Their formation also occurs in vivo at neutral pH by nitric oxide generated by bacteria converting nitrates and nitrites, or macrophages or endothelial cells metabolizing arginine [522,523,524]. *N*-Nitroso compounds have been found in over 200 foods, including fruits, vegetables, beverages, meats and cereals, and can be present in drinking water [456,519,521,525,526]. Concentrations of nitrosamines in food depend on the method, time and temperature of cooking or fat composition [527]. Thus, mean levels of *N*-nitroso compounds were 0.5 μmol/kg of fresh meat and over 5.5 μmol/kg of frankfurters and salted, dried fish [526]. Nevertheless, the levels in most foods are generally below 10 ppb [519,528] or less than 10 μg/kg, with exception of fired food, such as bacon, in which an average concentration is 36 μg/kg [521].

*Carcinogenicity*: Various dialkyl and cyclic nitrosamines have been found to produce tumors at multiple sites in a range of species, including rats, mice and hamsters. Target organs of carcinogenicity include the liver, esophagus, nasal and oral mucosa, kidney, pancreas, urinary bladder, lung and thyroid [8,68,521,529,530,531,532]. *N-nitrosodiethylamine* (NDEA) (Figure 5(2)) is considered to be an exceptionally potent carcinogen, compared to other nitrosamines [533] (Table 2).

*Genotoxicity/DNA Binding (Adducts)*: As a class, nitrosamines, particularly the volatile nitrosamines, are potent mutagens/genotoxins, both in vitro and in vivo [68,521,529,530]. Many of the nitrosamines act as alkylating agents [534,535] leading to pro-mutagenic lesions, including alkylation at the *N*7 and O^6^ positions of guanine or O^4^ position of thymidine [536,537,538]. For example, NDEA (Figure 5(2)) and *N-nitrosodiethanolamine* (Figure 5(3)) form poorly repaired O^4^-ethyldeoxythymidine and O^6^-2-hydroxyethyl-deoxyguanosine DNA adducts [536,539]. Some organ specificity of nitrosamines may arise because, unlike the liver, sensitive tissues such as the brain lack a DNA-repair enzyme, alkyltransferase, that regenerates guanine from O^6^-alkylguanine [540].

*Biotransformation*: *N*-Nitroso compounds are bioactivated to diazonium ions by hydroxylation involving several CYP isozymes [521,541], in particular CYP2E1 [536,542]. The organ specificity probably stems from tissue specific CYPs that activate the nitrosamines which alkylate DNA in the organ where they are activated.

*MoA:* Formation of alkylated DNA adducts and consequent mutagenesis and genomic instability are likely the most prevalent cancer-related mechanism of nitrosamines [529,533,536,543]

*Human Exposure*: In Europe, EDI of volatile *N*-nitrosamines ranges from 0.001 to 0.02 μg/kg bw/day [521]. In US the average daily intake was calculated to be 1.8 μg/day in a vegetarian diet and 1.9 μg/day in a Western diet [544]. The highest values of nitrosamines, up to 0.531 μg/serving, were found in alcohol, meat and dairy products [525].

*Human Effects*: In humans, indirect evidence exists of the carcinogenic action of nitrosamines through reported associations between gastrointestinal (esophageal, gastric, colorectal), pancreatic, bladder cancers and the consumption of foods containing relatively high concentrations of nitrosamines, nitrites and nitrates [68,521,529,530]. Additionally, nasopharyngeal cancers associated with consumption of salted fish have been attributed to *N*-nitroso compounds (see below) [545].

*Risk*: IARC classified the majority of food-borne nitrosamines as either probable (Group 2A) or possible (Group 2B) human carcinogens [67], although, certain practices known to result in increased cancer risks, including consumption of processed meat and fish, smoking, and betel quid chewing, and certain occupations in the rubber industry, result in exposures to various nitrosamines. The lack of identification of nitrosamines as “known human carcinogens” is largely a consequence of the low levels of human exposure to these compounds. Using the benchmark approach, permissible daily exposures (PDE) for cancer and mutagenicity were calculated to be 6.2 and 0.6 μg/person/day for *N*-nitrosodimethylamine and 2.2 and 0.04 μg/person/day for NDEA, respectively [543].

#### 3.5.3. α,β-Unsaturated Aldehydes

*α,β-Unsaturated aldehydes* (Figure 5(4–8)) compose a wide ranging class of aldehydes which naturally occur in a variety of foods and can be added as flavor ingredients. In addition, they can be formed endogenously through lipid peroxidation [546,547,548,549]. They are formed from the polyunsaturated fatty acids (PUFA) in triglycerides, as well as from any free fatty acids, which are susceptible to auto oxidation [548,550].

##### 3.5.3.1. Malondialdehyde, 4-Hydroxynonenal, Crotonaldehyde, *trans,trans*-2,4-hexadienal

*Occurrence:* Several α,β-unsaturated aldehydes, including *malondialdehyde* (MDA) (Figure 5(4)), in its enolic form, *4-hydroxynonenal* (HNE) (Figure 5(5)) and *crotonaldehyde* (CA) (Figure 5(6)), occur as contaminants in food, especially edible oils [548,551,552]. *Trans,trans-2,4-hexadienal* (Figure 5(7)) is used as a flavoring agent [548,549,553] and was detected in a variety of food products, including olives, caviar, chicken and beef [115].

*Carcinogenicity:* The α,β-unsaturated aldehydes that have been tested in standard 2-year bioassays in rodents include CA [554], MDA [555], and *trans*,*trans*-2,4-hexadienal [556,557]. In the rat, CA administered at 0.6 and 6.0 mM/L in drinking water for 113 weeks was associated with development of neoplastic nodules of the liver [554] in conjunction with overt hepatotoxicity (necrosis, fibrosis, cholestasis, and inflammation). In the same strain of rats, the incidence of thyroid follicular cell neoplasms was increased following 103 weeks of administration of MDA at 100 mg/kg bw/day (5 days/week) by oral gavage, in addition, pancreatic islet cell adenomas were observed in male rats in the group that received 50 mg MDA/kg [555,558]. Dosing of rats and mice with *trans*,*trans*-2,4-hexadienal by gavage in com oil at dosages greater than 45 mg/kg, 5 days/week, for up to 105 weeks resulted in an increased incidence of squamous-cell papillomas and carcinomas of the forestomach in both species [115,556]. In a neonatal mouse model, no tumors were observed after administration of CA, MDA and HNE via intraperitoneal injections up to 3000 nmol [559].

*Genotoxicity/DNA Binding (Adducts)*: CA [552,560,561], HNE [562], MDA [551,563], and *trans*,*trans*-2,4-hexadienal [115,557,560,561] have been shown to be genotoxic, especially in vitro [548]. Unsaturated aldehydes are considered to be strong alkylating agents, and as such they can covalently bind to DNA and proteins. In particular, formation of DNA adducts was detected in vitro and in vivo in multiple tissues of rats and mice after exposure to CA [552], MDA [551], and *trans*,*trans*-2,4-hexadienal [115,556]. DNA adducts of CA have been also detected in exposed humans [552,564]. Another lipid peroxidation product, HNE, reacts with DNA chemically and can form DNA adducts [565,566], however it may be too reactive with proteins for DNA adducts to be formed in vivo if administered directly. Thus, in serum-containing medium HNE was not mutagenic to cultured cells, whereas a protected form was [567]. Depletion of GSH and resulting oxidative stress are thought to be prerequisites for formation of DNA adducts by unsaturated aldehydes [548].

*Biotransformation*: The oxidation of fatty acids leads to formation of hydroperoxides, which in turn, decompose in a terminal reaction to form aldehydes from the methyl terminus of the fatty acid chain [548]. The levels of hydroperoxides formed are often estimated by assay of thiobarbituric acid-reactive substances [568,569], but more precise measurement techniques are available [570]. Detoxication of unsaturated aldehydes occurs primarily through reactions with GSH, yielding metabolites that are excreted in the urine of rats and humans [548,552].

*MoA:* In addition to direct DNA-reactivity and mutagenicity, oxidative stress and immunomodulation might also play a role in the carcinogenic MoA of α,β-unsaturated aldehydes [115,548,552]. Based on the findings in vitro and in vivo, EFSA ruled out genotoxicity concern for *trans*,*trans*-2,4-hexadienal [557].

*Human Exposure:* Humans are exposed to α,β-unsaturated aldehydes from food and alcoholic beverages, as well as endogenously, particularly in some disease states [548,571]. Levels of dietary exposure are low, particularly in the case of the flavoring agent *trans*,*trans*-2,4-hexadienal whose per capita intake is estimated at 100 μg/kg of bw/day [549].

*Human Effects:* Several epidemiological studies provided little evidence of a positive association between CA exposure with the lung [572,573], oral cavity, stomach, and colon [574] cancer risks in humans [552].

*Risk:* IARC [115,552] classified CA and *trans*,*trans*-2,4-hexadienal as possibly carcinogenic to humans (Group 2B), while MDA was considered to be not classifiable as to its carcinogenicity to humans (Group 3) [558] (Table 2). In a risk assessment of CA [575], a conclusion was made that based on an analysis of the doses which produced DNA adducts, use of the hepatocellular tumor data from the rat drinking water carcinogenicity study is likely to overestimate human cancer risk. This is indicative of a practical threshold for the genotoxic and carcinogenic effect of CA. The JECFA evaluation [549] of the NOAEL for *trans*,*trans*-2,4-hexadienal of 15 mg/kg bw/day was based on the NTP Report [556] and was estimated to be >100,000 times its current EDI when used as a flavoring agent, thus JECFA concluded that this flavoring agent does not pose a safety concern. FEMA reaffirmed α,β-unsaturated aldehydes as GRAS, based on the lack of evidence of potential hazard to human health at concentrations present in food [548].

##### 3.5.3.2. Trans-2 hexenal

*Occurrence*: *Trans-2-hexenal* (2-HEX) is a 6-carbon aliphatic unsaturated aldehyde (Figure 5(8)), which accounts for over 65% of total annual volume of unsaturated aldehydes used as flavor ingredients [548,549]. 2-HEX has been identified in a variety of plant species, including peppers, tomatoes and potatoes, and is referred to as leaf aldehyde [576]. The highest content of 2-HEX was reported in bananas, which contain approximately 32 ppm or 76 mg/kg [548,577].

*Carcinogenicity*: 2-HEX has not been tested for carcinogenicity in a 2-year bioassay [578]; however, based on structural similarities with *trans*,*trans*-2,4-hexadienal (Figure 5(7,8)), it can be expected to produce forestomach tumors in rodents when administered by oral gavage [548]. Some evidence of tumorigenicity was found in rats and mice that received three intraperitoneal injections of 2-HEX at the total dose of 150 mg/kg bw 18-month after the exposure [579]. Specifically, higher incidences of leukemia, liver and kidney tumors were described in mice, while in rats, tumors of parotid gland and lungs were reported.

*Genotoxicity/DNA Binding (Adducts)*: 2-HEX was genotoxic in several assays in vitro producing DNA damage, mutagenicity, clastogenicity and aneugenicity [548,553,578,580]. No induction of gene mutations, direct DNA strand breaks or micronucleus formation was reported in rodents in vivo [553,579]. In humans, non-smoking volunteers that consumed three to six bananas per day for 3 days showed at least a doubling of micronuclei in exfoliated buccal cells [581]. Rinsing the oral cavity with water containing 10 ppm 2-HEX produced a more pronounced effect. Subsequent experiments in rats [575,582] concluded that such exposures posed a negligible risk except in very special situations. 2-HEX has been also shown to form adducts with DNA and proteins [548,553]. Specifically, cyclic 1,*N*2-propanodeoxyguanosine adducts were detected in vitro and in several tissues of rats, including forestomach, esophagus, liver and kidneys, following oral doses of up to 500 mg/kg bw; however, the covalent binding index was calculated to be extremely low (0.06) [583,584,585,586]. Using a physiologically based in silico model developed in rats, formation of 2-HEX DNA adducts in humans from current levels of dietary intake was predicted to be three orders of magnitude lower compared to endogenous DNA adduct levels [587].

*Biotransformation*: 2-HEX is readily oxidized to *trans*-2-hexenoic acid in vitro by mouse cytosolic fraction and isoenzymes of rat aldehyde dehydrogenase [548,553]. Conjunction with GSH is a major mechanism involved in detoxication [548,549,587].

*MoA*: While DNA adduct formation can be related to mutagenicity of 2-HEX in vitro, EFSA concluded that based on the in vivo findings, concern for genotoxicity for this compound can be ruled out [553]. Induction of oxidative damage, exacerbated by GSH depletion, can potentially play an important role in carcinogenicity [588].

*Human Exposure*: Combined daily per capita intake of 2-HEX from foods was calculated to be 2390 μg/person per day or 31–165 μg/kg bw/day, with majority of exposures occurring with consumption of bananas [548,575]. An EDI based on the maximized survey-derived daily intake of 2-HEX as a flavoring substance was calculated to be 409 and 2800 μg/capita per day (or 0.007 and 0.05 mg/kg bw per day) in US and Europe, respectively [578].

*Human Effects*: Data assessing association of human cancer risk with 2-HEX exposure are currently lacking.

*Risk*: JECFA [549] and EFSA [578] concluded that 2-HEX would not pose a safety concern at the current levels of intake as a flavoring substance. Based on low covalent binding of 2-HEX, estimated cancer risk of 1–5 per 10^7^ lives was considered to be negligible; however, under certain circumstances, utilization of 2-HEX as a flavoring agent or fungicide can increase cancer risk to 2–6 per 10^4^ lives [575,582].

### 3.6. Carcinogenicity of Preserved and Processed Foods

Smoking and pickling of foods have long been suspected of leading to formation of carcinogens, particularly *nitrosamines* [519,589,590].

#### 3.6.1. Preserved Vegetables

Consumption of pickled vegetables, prepared with or without salting, which is predominant in some regions of Asia, such as China, Japan and Korea, showed some association with higher risk of stomach, nasophageal or esophageal cancers; however, the evidence is not consistent [305,591]. In a meta-analysis of 16 case-control studies, the highest versus lowest preserved vegetable intake was associated with a 2-fold increase in the risk of nasopharyngeal cancer, whereas consumption of non-preserved vegetables was associated with reduced risk [592]. The increased risk was attributed to the content of nitrates and nitrosamines. Based on the limited evidence for carcinogenicity of pickled vegetables in humans and inadequate evidence for carcinogenicity in experimental animals, IARC [305] classifies pickled vegetables as possibly carcinogenic to humans (Group 2B) (Table 2).

#### 3.6.2. Red and Processed Meat

In many countries, consumption of red meat, usually cooked, and processed meats that have been prepared thorough salting, curing, fermentation and smoking varies from 50 to 200 g/day [593]. While the evidence of carcinogenicity of consumption of either red or processed meat in experimental animals is inadequate, a variety of studies reported an association between the consumption of such meats with higher risk of colorectal, pancreatic, prostate, breast, endometrial, liver and gastric cancers in humans [593,594,595,596]. Cooking and processing of meat results in formation of various genotoxic carcinogens, including *N*-nitroso compounds, HACs and PAHs (discussed above), which are capable of inducing pro-mutagenic DNA damage, contributing to carcinogenesis [593,597]. In addition, consumption of processed meat can produce oxidative stress and formation of lipid peroxidation products that could contribute to carcinogenic MoA [593]. Based on the available data, IARC [593] classified consumption of red meat as probably carcinogenic to humans (Group 2A), while consumption of processed meat was considered to be carcinogenic to humans (Group 1) (Table 2).

#### 3.6.3. Salted Fish

Salted fish is produced and consumed primarily in Southeast Asia and northern Europe. Low levels of several volatile nitrosamines (discussed above) have been detected in Chinese-style salted fish [598], which is prepared by treating with dry salt or an aqueous salt solution followed by drying in the sun, and high levels of *N*-nitrosodimethylamine have been reported in some samples [599,600,601].

Several experiments have demonstrated that feeding of high concentrations (i.e., >5%) of Chinese-style salted fish in the diet induced nasal cavity tumors in rats [602,603,604], a site for carcinogenicity of nitrosamines [531]. Administration of an extract of nitrate-treated fish induced glandular stomach cancer in rats [605]. In addition, *N*-nitrosamines-specific DNA adducts were detected in the livers and kidneys of rats which were fed Chinese salted fish [606].

In humans, association between consumption of salted fish and cancer incidences has been demonstrated [599]. A population-based case-control study [607] showed that individuals with the highest intake of salted fish had an 80% increase in risk of nasopharyngeal carcinoma and a linear trend with respect to protein-containing preserved foods. In addition, a meta-analysis of cohort studies in Korea, China and Japan supported the evidence that consumption of salted fish is associated with a 1.2-fold increase in the risk of gastric cancer [591], 1.2- to 1.45-fold increased risk for nasopharyngeal carcinoma [608], as well as higher risk of stomach and colon cancer [609]. Potential association of nasopharyngeal tumors in endemic areas with Epstein-Barr virus cannot be excluded [599]. IARC [599] concluded that there was sufficient evidence in humans for causation of nasopharyngeal carcinomas by Chinese-style salted fish, and classified it as carcinogenic to humans (Group 1) (Table 2).

## 4. Epigenetic Carcinogens or Carcinogens with Uncertain Mode of Action and Related Chemicals Present in Food

**Figure 6:**
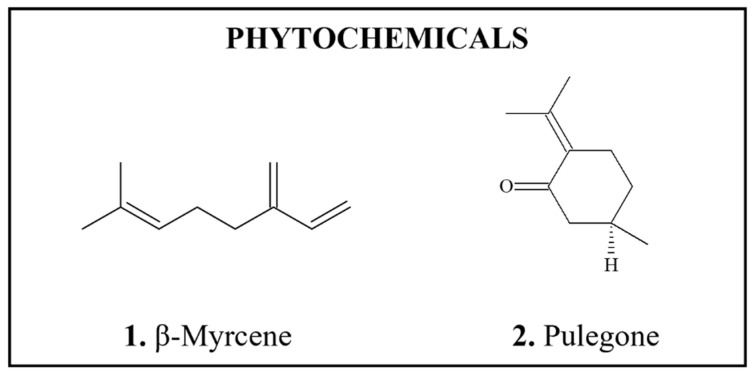
Chemical structures of non-DNA-reactive carcinogenic phytochemicals present in foods.

**Figure 7:**
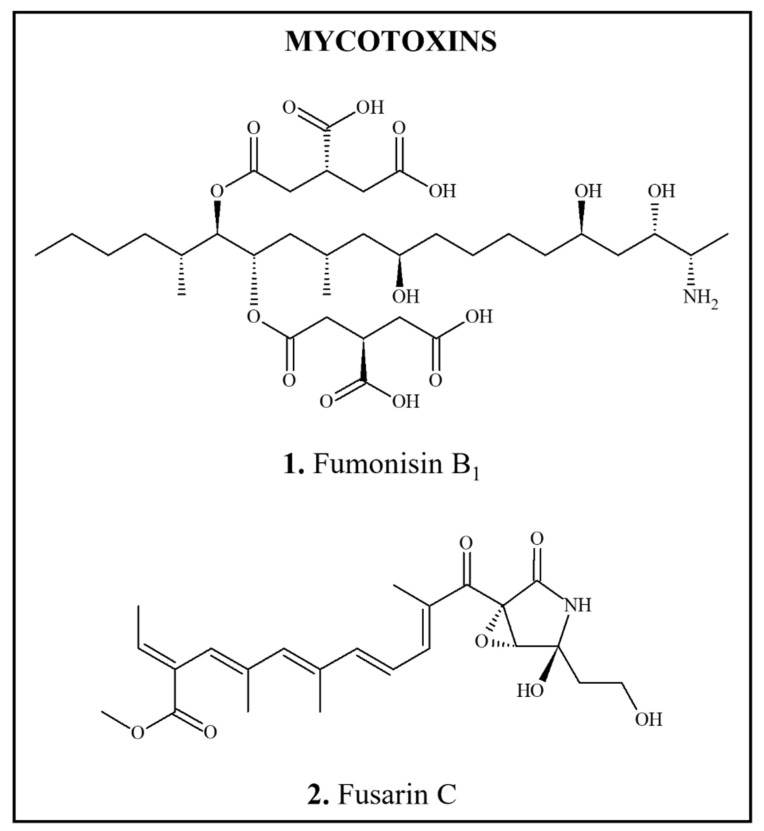
Chemical structures of non-DNA-reactive carcinogenic mycotoxins and related chemicals present in foods.

**Figure 8:**
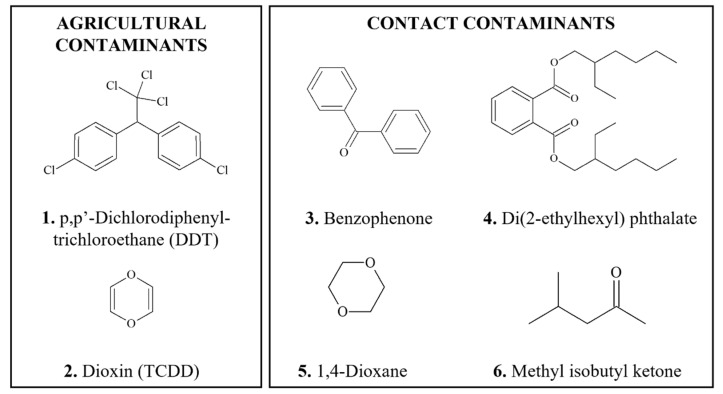
Chemical structures of non-DNA-reactive carcinogenic contaminants in food.

**Figure 9:**
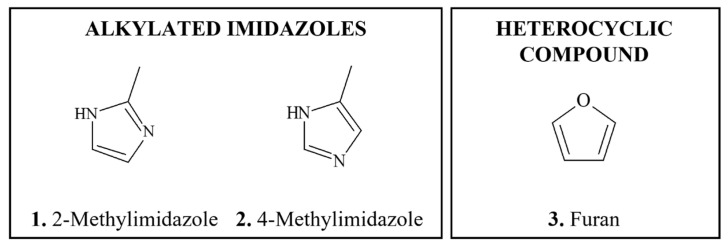
Chemical structures of non-DNA-reactive carcinogens formed during processing, packaging and storage of food.

**Figure 10:**
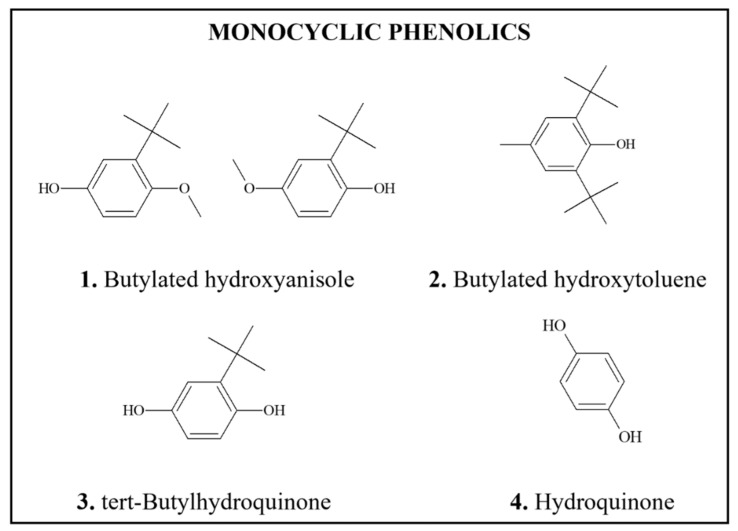
Chemical structures of non-DNA-reactive carcinogenic food additives.

This section provides an overview of food-derived carcinogens that are typically negative in genotoxicity assays in vitro and in vivo, and which facilitate neoplastic development through molecular and cellular mechanisms other than direct DNA reactivity. This section also includes carcinogens that do not have enough mechanistic data for classification. Chemical structures of carcinogens and related chemicals discussed in this section are provided in Figure 6, Figure 7, Figure 8, Figure 9 and Figure 10.

### 4.1. Phytotoxins

In 2018, FDA announced a ban on seven synthetically derived agents, including methyl eugenol, myrcene, pulegone, benzophenone, ethyl acrylate, pyridine and styrene for use as flavoring substances [610]. The majority of these substances have natural counterparts, discussed in this manuscript.

#### 4.1.1. β-Myrcene

*Occurrence: β-myrcene* (Figure 6(1)) is an acyclic monoterpene, which occurs naturally in a variety of plants, including verbena, lemongrass, bay, rosemary, basil, cardamon and is a constituent in many fruits, vegetables and beverages, such as citrus peel oils and juices, pineapple, celery, carrot, beer, white wine, and many others [611,612,613]. The highest levels of β-myrcene, up to 10 g/kg dry weight, were reported in hops [611]. It is also widely used as a flavor and fragrance material.

*Carcinogenicity:* Oral administration of β-myrcene by gavage up to 1000 mg/kg bw, 5 days a week, induced a significant increase in liver tumors (adenomas and carcinomas) in male and female mice. In rats, increased incidences of renal tubular adenomas and carcinomas were reported [611,614,615,616].

*Genotoxicity/DNA Binding (Adducts)*: β-myrcene lacks genotoxicity and mutagenicity in vitro and in vivo [611,613,614,616,617,618,619], accordingly, no covalent DNA binding was reported.

*Metabolism*: Metabolism of β-myrcene involves oxidation of the carbon–carbon double bond (Figure 6(1)) to an epoxide intermediate, which after hydrolysis gives rise to diol conjugates, 10-hydroxylinalool and 7-methyl-3-methyleneoct-6-ene-1,2-diol, that were detected in the urine of rabbits and rats [611,620,621,622]. Diols are further oxidized to corresponding aldehydes and hydroxy acids [613]. These reactions are likely metabolized by CYPs [619], and β-myrcene was shown to inhibit activity of CYP2B1 in vitro and induce CYP2B1/B2 in vivo [623,624].

*MoA*: The mechanism of tumor induction by β-myrcene remains largely unknown [612]. Analyses of cancer data by FEMA [619,622] suggested that hepatocarcinogenesis in mice and renal tumors in rats are secondary to cytotoxicity of β-myrcene at high carcinogenic doses, and in the kidney are related to the chronic progressive nephropathy and possibly unusual nephrosis. While it is structurally similar to another terpene, d-limonene, which is known to bind to α_2u_-urinary globulin producing nephropathy, IARC [611] concluded that β-myrcene did not meet all of the criteria to explain its carcinogenicity by a α_2u_-globulin-associated mechanism. Histopathologic assessment of the kidneys from rats chronically dosed with β-myrcene confirmed that due to complex renal pathology, α_2u_-globulin nephropathy cannot be the sole MoA of carcinogenicity [625].

*Human Exposure*: Daily per capita intake of β-myrcene in US was calculated to be 3 µg/kg bw/day [622,626]. Another, more recent FDA estimation suggested an EDI of 1.23 μg/kg bw/day [612]. In Europe, estimated per capita intake was calculated to be 4.8 μg/kg bw/day [616].

*Human Effects*: No findings on human carcinogenicity are available [611].

*Risk*: IARC [611] classifies β-myrcene as possibly carcinogenic to humans (Group 2B) (Table 2). Safety assessment of β-myrcene by FEMA concluded that MoA of carcinogenicity in rodents is not relevant to humans and rodent carcinogenicity is not indicative of a health risk [619,622]. JECFA and EFSA [616,626] concluded that at estimated current dietary intake, β-myrcene would not pose a safety concern. Despite these conclusions, FDA recently withdrew authorization for use of a synthetic form of myrcene as a food additive due to its carcinogenicity in accordance with the Delaney Clause [610,612,627].

#### 4.1.2. Pulegone

*Occurrence*: *Pulegone* (PUL) ((R)-5-methyl-2-(1-methylethylidine)cyclohexanone) (Figure 6(2)) is a naturally occurring monoterpene ketone found in a variety of plants, in particular mint species, such as *Nepeta cataria* (catnip), *Mentha piperita*, and pennyroyal and is used as a flavoring agent [628,629,630,631].

*Carcinogenicity:* Oral administration of PUL to mice at up to 150 mg/kg bw in corn oil by gavage, 5 days/week for 105 weeks, significantly increased incidences of hepatocellular adenoma in both sexes and incidences of hepatoblastoma in male mice [628,632]. In rats, increased incidences of urinary bladder neoplasms were observed in females only, while no evidence of carcinogenic activity was observed in males.

*Genotoxicity/DNA Binding (Adducts)*: PUL was not genotoxic or mutagenic in vitro and in vivo [628,629,631,632,633]. Genotoxicity studies with herbal preparations containing PUL, such as peppermint oil, also yielded negative result [631]. No covalent DNA binding has been reported, although reactive metabolites of PUL can covalently bind to proteins [634].

*Metabolism*: PUL is metabolized by different pathways, including hydroxylation in the 9-position to toxic metabolite menthofuran or in the 5-position to piperitenone; reduction of the carbon-carbon double bond, which results in formation of menthone and isomenthone; or conjugation with GSH [631,635,636]. Hydroxylation to menthofuran involves multiple CYPs, including human CYP2E1, CYP1A2, CYP2C19 and CYP3A4 [637,638,639]. The major metabolites of PUL detected in humans were 10-hydroxypulegone, 8- and 1-hydroxymenthone, and menthol [640]. Metabolism of PUL to menthofuran can result in formation of reactive metabolites, in particular, epoxide pulegone 8-aldehyde (γ-ketoenal) and *p*-cresol, that can bind to proteins and deplete GSH levels [628,631,634,639,641].

*MoA:* An epigenetic MoA for PUL-induced urinary bladder tumors in female rats was proposed to involve chronic exposure to high concentrations resulting in excretion and accumulation of PUL and its cytotoxic metabolites, particularly piperitenone, in the urine, leading to urothelial cytotoxicity and sustained regenerative urothelial cell proliferation eventually resulting in development of urothelial tumors [642]. In addition, toxicity of menthofuran and covalent binding of its metabolites to proteins can lead to chronic regenerative cell proliferation, which can contribute to liver and urinary bladder carcinogenesis [628,630,631].

*Human Exposure:* Dietary exposure to PUL results primarily from ingestion of products flavored with spearmint or peppermint oil, such as confectionery, chewing gum, as well as alcoholic and non-alcoholic beverages [630,631]. JECFA [629] estimated an intake for PUL of approximately 2 µg/person/day or 0.04 µg/kg bw/day in Europe and 12 µg/person/day or 0.03 µg/kg bw per day in USA. The European Commission (Regulation EC No. 1334/2008) [106] set a limit of 20 mg/kg for PUL and menthofuran in foods and beverages.

*Human Effects:* No epidemiological studies linking PUL to human cancer risk have been conducted [628].

*Risk:* IARC [628] concluded that PUL was possibly carcinogenic to humans (Group 2B) (Table 2) based on sufficient evidence for carcinogenicity in experimental animals but inadequate evidence in humans. JECFA [629] found no safety concern when PUL is used as a flavoring agent. EMA [631] suggested that MoA for tumor induction in rodents is not relevant for carcinogenicity risk in humans, and recommended an acceptable exposure limit of 0.75 mg/kg bw/day.

### 4.2. Mycotoxins

Fumonisin B_1_ and Fusarin C are the major toxins derived from *Fusarium* fungi species, *Fusarium verticilloides* (also known as *moniliforme*) and *proliferatum*, which are common contaminants on crops, in particular corn [247,276,305,643].

#### 4.2.1. Fumonisins

*Occurrence: Fumonisin B*_1_ (FB_1_) (Figure 7(1)), is the most prevalent member of fumonisins class. It has the chemical structure of a substituted 2-amino-icosane diester, which has features in common with the sphingoid base backbone of sphingolipids [644,645]. The highest concentrations of FB_1_, which range from 310 to up to 23,800 μg/kg, were reported in maize and maize-based cereal products [276].

*Carcinogenicity*: In female mice, oral administration of FB_1_ caused an increase in hepatocellular adenomas and carcinomas. In the study in male rats, an increase in cholangocarcinomas and hepatocellular carcinomas were observed, while in the other rat study, FB_1_ induced renal tubule carcinomas in males exposed to up to 100 ppm [247,646,647]. FB_1_ was reported to have liver cancer initiating activity, as evidenced by induction of preneoplastic foci in rats by 7 weeks of dosing [648]. Some studies suggest that it also has tumor promoting activity [649,650]. For example, FB_1_ administered in diet had promotional activity on liver tumors initiated by AFB_1_ and *N*-methyl-*N*′-nitro-*N*-nitrosoguanidine in trout [651].

*Genotoxicity/DNA Binding (Adducts):* The structure of FB_1_ (Figure 7(1)) lacks features that confer DNA reactivity. Accordingly, it was not mutagenic in bacteria; however, a positive result was reported in a luminescence induction assay in the absence of metabolic activation [247,647]. The compound did not induce DNA repair synthesis in the liver cells of rats in vitro or in vivo and no evidence for DNA adduct formation with oligonucleotides in vitro was found [247]. However, evidence for induction of DNA damage by FB_1_ was reported in rat brain glioma cells and human fibroblasts in vitro, and in spleen and liver cells isolated from exposed rats [652,653,654]. In addition, FB_1_ caused DNA fragmentation in rat liver and kidney [655]. Positive results were obtained in micronucleus assays in vitro with human-derived hepatoma (HepG2) cells but not with rat hepatocytes [656]. In bone marrow of mice, an increase in formation of micronuclei was found after intraperitoneal injection of FB_1_ [657]. Positive results were obtained in chromosomal aberration assays with rat hepatocytes.

*Metabolism*: There is little or no evidence that fumonisins are metabolized in vivo or in vitro [247,646,647]. Nevertheless, FB_1_ induced CYP1A activity in hepatoma cell line, and CYP2E activity in rats, while inhibiting CYP2C11 and CYP1A2 enzymes [658,659]. Liver and kidney retain most absorbed material [660]. Hydrolyzed FB_1_ is more toxic compared to the parental form, and recently, hydrolyzed metabolites were detected in the kidney and liver of rats administered FB_1_ by intraperitoneal injections [661].

*MoA:* One postulated MoA for FB_1_ carcinogenicity involves disruption of sphingolipid metabolism, either through inhibition of ceramide synthesis [662] or due to changes in polyunsaturated fatty acid and phospholipid pools [649], leading to alteration of signaling pathways that control cell behavior and DNA synthesis [247,645,660,663,664,665,666]. Such perturbations produce alterations in cell turnover. Another proposed MoA involves oxidative stress, which is likely to mediate DNA damage observed in some assays [276,291,652,654,660]. In support of this hypothesis, FB_1_ was demonstrated to increase lipid peroxidation in rat kidney and liver and decrease levels of antioxidant enzymes [667,668]. In addition, dosing of rats with 100 µg/kg bw for 12 weeks resulted in downregulation of hepatic antioxidant genes [655].

*Human Exposure:* In Europe and North America, EDI to FB_1_ ranges from 0.01 to 0.2 μg/kg bw/day, while in other countries with different climate, cultivation practices and higher consumption of maize and maze-based products, EDI levels are much higher, reaching up to 354.9 μg/kg bw/day in South America and Africa, and up to 740 μg/kg bw/day in China [660]. The highest levels of chronic dietary exposure, ranging from 0.18 to 3.9 μg/kg bw/day for FB_1_ and from 0.27 to 6.4 μg/kg bw/day for total fumonisins were reported in children, with cakes, cookies and pies, cereal-based foods and cereal grain being the main contributors [276]. JECFA reported that in adults, mean chronic exposures to FB_1_ did not exceed 0.56 μg/kg bw/day for FB_1_ and 0.82 μg/kg bw/day for total fumonisins, respectively.

*Human Effects:* Epidemiological evidence shows a link between exposure to *F. moniliforme* contaminated corn and esophageal and hepatocellular cancer [247,305,669,670,671,672] but these reports do not indicate the specific compounds involved. Others [673,674] investigated FB_1_ specifically, but were not able to find significant association between exposure and cancer risk [276].

*Risk*: IARC [247] evaluated FB_1_ as possibly carcinogenic to humans (Group 2B) (Table 2) based on sufficient evidence for carcinogenicity in experimental animals and inadequate evidence in humans. JECFA [276,647] established a PMTDI of 2 μg/kg bw/day for FB_1_ alone or in combination with other fumonisins, and recommended to reduce exposures to fumonisins, especially in the areas where maize is consumed at higher levels.

#### 4.2.2. Fusarin C

*Occurrence*: *Fusarin C* (FC) (Figure 7(2)) belongs to 2-pyrrolidinone metabolites produced by various species of the fungus *Fusarium*, including *Fusarium moniliforme* and *oxysporum* [305,675]. FC has been detected in corn and maize grain in concentrations ranging from 28 to 83 mg/kg [305,676,677]. While unstable to heat, FC may survive cooking process [678].

*Carcinogenicity:* FC induced papillomas and carcinomas of the oesophagus and forestomach in mice and rats when administered by oral gavage at 0.5 or 2 mg twice a week to mice or rats, respectively [305]. FC did not act as a promoter in rat liver [679].

*Genotoxicity/DNA Binding (Adducts)*: FC was genotoxic in vitro in the presence of exogenous bioactivation, producing mutagenicity, SCE, chromosomal aberration and micronuclei formation [305,680,681]. Only marginal effect was observed in UDS assay in rat hepatocytes [682]. Currently, no in vivo genotoxicity studies with FC were reported. While crude extracts of *Fusarium moniliforme* produced direct mutagenicity in bacteria as well as positive results in NPL assays, no DNA adducts were measured by NPL assay with pure FC [683,684].

*Metabolism*: FC accumulates mainly in the intestines, stomach and liver after administration by gavage to rats. Studies utilizing rat liver microsomal enzymes showed that FC is metabolized by carboxyesterase to water-soluble fusarin PM_1_, while monooxygenase is involved in the FC conversion to a mutagenic metabolite [305,685,686]. Hydroxylation at the 1-position resulted in production of two genotoxic metabolites, fusarin Z, which was the most potent mutagen in vitro, and fusarin X [687].

*MoA*: The role of mutagenic effects of FC in the development of cancer is not clear. One study suggested that FC may act as an estrogenic agonist in vitro [688]; however, no effects on mammary glands were detected in carcinogenicity studies [305].

*Human Exposure:* Major source of exposure to FC is maize and maize grain [305,676,677]. Currently, no data on dietary intake levels of FC in humans have been reported.

*Human Effects:* FC has been suggested to be responsible for the high incidence of esophageal cancer in China [680] and South Africa [689]. Changes in the staple diet of Black South Africans from sorghum to maize (corn), on which the fungus grows more easily, has been associated with the epidemic of squamous carcinoma of the esophagus in that area [671].

*Risk:* IARC [305] classifies FC, similar to other toxins derived from *Fusarium moniliforme* as possibly carcinogenic to humans (Group 2B) (Table 2).

### 4.3. Environmental, Agricultural and Industrial Contaminants

A variety of industrial contaminants and chemicals used in crop protection and production have caused cancers in experimental models and have been considered to be likely human carcinogens [67,275,690,691]. Traces of these chemicals can contaminate food, albeit at extremely low levels. The cancer risk that such exposures might pose has been a matter of debate.

#### 4.3.1. Agricultural Contaminants

##### 4.3.1.1. *p,p′*-dichlorodiphenyltrichloroethane

*Occurrence*: Technical grade *p*,*p*’-*dichlorodiphenyltrichloroethane* (DDT) (Figure 8(1)) is a complex mixture of DDT, its isomers and related compounds. As an organochlorine insecticide, DDT, had a major impact on the incidence of malaria and typhus as a cheap and effective method of killing the female *Anopheles* mosquito, which is the malaria parasite vector, and lice, which spread *Rickettsia prowazekii*, the cause of epidemic typhus [691]. Cost-effectiveness analyses shows that DDT is the least expensive, yet effective insecticide for prevention of malaria that kills thousands of people each day [692,693]. Nevertheless, owing to the extensive use of DDT in agriculture to control insects, such as the pink boll worm (*Pectinophora gossypiella*) on cotton, codling moth (*Cydia pomonella*) on deciduous fruit, Colorado potato beetle (*Leptinotarsa decemlineata*), and the European corn borer (*Ostrinia nubilalis*), and DDT’s resistance to degradation or metabolism, which results in bioaccumulation in the food chain, it has been largely banned in the early 1970s with the expectation that all use would be stopped [694]. In 2006, however, the World Health Organization (WHO) reversed a 30-year policy by endorsing the use of DDT for malaria control [695]. DDT and its metabolites and degradation products, *p*,*p*′-dichlorodiphenyldichloroethylene (DDE) and *p*,*p*′-dichlorodiphenyldichloroethane (DDD), have been found in human breast milk [696,697], as well as some of the foods, including American cheese, butter, catfish, carrots, summer squash, celery, and salmon [694]. IARC [691] and Smith [696] reported that the mean concentrations of DDT in population have declined in much of the world: from 5000–10,000 µg/kg to around 1000 µg/kg of milk fat or even lower over the last three decades. Although different concentrations are found in different regions, the declines seen in various countries correspond to their restrictions on use of DDT.

*Carcinogenicity:* In some studies, DDT produced liver tumors in rats and mice at doses exceeding 46 mg/kg bw by gavage or 250 mg/kg in diet, as well as increases in incidences of malignant lymphomas and lung neoplasms in mice [68,691,694]. In contrast, US National Cancer Institute [698] bioassays at up to 642 ppm in male rats and 175 ppm in female mice detected no evidence for carcinogenicity of DDT. The DDT metabolites, DDE and DDD, also were hepatocarcinogenic in mice [699].

*Genotoxicity/DNA Binding (Adducts)*: The genotoxicity data on DDT and related compounds were overwhelmingly negative; however, some evidence of DNA damage, chromosome aberrations, and micronuclei formation was reported in human lymphocytes exposed to DDT in vitro [691,694,699,700]. No covalent DNA binding has been reported.

*Metabolism:* Due to its high lipophilicity, DDT, DDE and DDD tend to accumulate in the adipose tissue [694,701,702]. In mammals, including humans, DDT is primarily dehydrochlorinated to DDD, which is further metabolized to easily excreted 2,2-bis-chlorophenyl acetic acid isomers. To a lesser degree, DDT is also converted to DDE, which tends to bioaccumulate in lipid-rich tissues [691,700,702]. In rats, DDT and its metabolites has been shown to induce several CYPs, including CYP2B and CYP3A [703]. DDT can be also biotransformed to methylsulfonyl intermediates, which exhibit toxicity, in particular in adrenal gland [704].

*MoA*: DDT was shown to have a liver tumor promoting effect in mice [705], which, based on mechanistic studies [706], was attributed to its accumulation in the lipid layer of liver cell membranes and reduction of cell-cell communication, thereby diminishing tissue homeostatic control of incipient neoplastic cells. This MoA implies a requirement for a sufficient exposure over time to maintain the interference with intercellular communication throughout the liver. IARC also found strong evidence that DDT acts as endocrine disruptor, is immunosuppressive and can induce oxidative stress, all of these MoAs are operable in humans [691].

*Human Exposure*: Numerous studies have investigated human exposure to organochloride pesticides such as DDT, specifically due to concerns over its ability to bioaccumulate in the body and persist in the environment. However, due to the ban of DDT and declining levels of DDT and its metabolites in humans, more recent exposure data are scarce. It has been estimated that over 90% of the DDT detected in the general population is derived from food, particularly from meat, fish, poultry, and root and leafy vegetables [691,694]. The highest average daily intake ranging from 24.2 to 27.8 μg/day, was observed in Arctic populations, that consume foods such as seal or whale [694]. Nevertheless, most countries have seen a significant decline in DDT intake, ranging from 20 to 40% [700]. For example, in Europe total dietary exposure to DDT and its metabolites decreased from 0.00627 μg/kg bw/day in 1997 to 0.0051 μg/kg bw/day in 2005, and in US a decline from 0.0213 to 0.0056 μg/kg bw/day was observed from 1984 to 1991 [700]. EFSA also concluded that in most European countries, current EDI values for DDT, which range from 0.005 to 0.03 μg/kg bw/day in adult and children and up to 1 μg/kg bw in breastfed infants, are below the established provisional TDI of 0.01 mg/kg bw.

*Human Effects*: While some positive associations between DDT and cancers of the liver and testis, and non-Hodgkin lymphoma were reported, there seems to be limited evidence that DDT and related compounds from any source increase cancer rates in humans [691,699,707,708,709,710], even in agricultural workers [711]. This absence of carcinogenicity may be due to insufficient exposures, although some occupational exposures have been substantial, or it may reflect the fact that most human populations do not display rates of spontaneous liver tumor development that are as high as sensitive rodent models, indicating a low background of initiation available for promotion to tumor formation.

*Risk*: In 2018, IARC [691] upgraded classification for DDT from possibly (Group 2B) to probably carcinogenic to humans (Group 2A) (Table 2), based on sufficient evidence of carcinogenicity in experimental animals and strong mechanistic evidence that MoA for DDT carcinogenicity can operate in humans.

##### 4.3.1.2. Dioxins and Dioxin-Like Compounds

*Occurrence: Dioxins* and related *Dioxin-Like-Compounds* (DLCs) refer to a complex family of chlorinated compounds with similar structures and biological effects. 2,3,7,8-Tetrachloro-*p*-dioxin (TCDD) (Figure 8(2)) is one of the most potent and prominent dioxins in the environment and is often referred synonymously as “dioxin”. Dioxin and DLCs, including polychlorinated dibenzo-para-dioxins (PCDDs), dibenzofurans (PCDFs) and the polychlorinated biphenyls (PCBs), are formed by dimerization of chlorophenols produced during the synthesis of chlorophenoxy acetic acid herbicides [275]. The dimerization of 2,4,5-trichlorophenol yields TCDD while the heterodimerization of 2,4,5-trichlorophenol with related phenols such as 2,4-dichlorophenol yields tri- through heptachlorinated dibenzodioxins and dibenzofurans. Other sources of TCDD include the use of chlorophenol as wood preservatives, use of chlorine in pulp bleaching, incineration of halogen containing materials [712,713]. DLCs usually occur as mixtures and, in order to express the expected biological activity of mixtures by a common dose metric, toxic equivalency factors (TEFs) relative to the activity of TCDD have been developed [275,365,690,714]. Using TEFs and mass concentrations, dioxin toxic equivalents (TEQs) for a source can be calculated. Based on these values, there are at least 7 PCDDs, 10 PCDFs and 12 PCBs that have dioxin-like activity [715,716]. Food-mediated human exposure to TCDD and DLCs occurs when contaminants from the above-described sources are ingested by animals, including fish, which in turn are used as human foods [275,690,717]. Dioxins have also been detected in human milk, ranging from 5 to 15 ng TEQ/kg lipid [717,718,719]. Levels of TCDD and DCLs in the environment, and consequently in food, have been declining since the late 1970s because of reduced industrial emissions [720].

*Carcinogenicity:* In rodents, several DLCs, including TCDD, induced neoplasia, mainly of the liver [275,690,721,722,723,724,725]. Other target organs and tissues included thyroid gland, lungs and oral mucosa. TCDD acted as tumor promoter when administered with potent tumor initiators, such as nitrosamines [275,365,724].

*Genotoxicity/DNA Binding (Adducts):* TCDD is not DNA-reactive in vitro or in vivo and does not covalently bind to DNA [365,723,726,727,728]. Similarly, PCBs are mainly not DNA-reactive, although some evidence of DNA damage, SCE and chromosomal aberrations were observed in human lymphocytes [690,729]. PCBs can be metabolically activated to electrophilic quinoid intermediates, and can produce DNA adducts in vitro; however, no DNA adducts were observed in vivo [730].

*Metabolism:* Similar to DDT described in the section above, TCDD and DLCs are highly lipophilic and thus, tend to accumulate in the adipose-rich tissues, liver has been also shown as a primary site of TCDD accumulation in rodents [365,731,732,733,734,735]. TCDD metabolism is very slow and limited and the compound is eliminated mainly unchanged in the feces [734,735], although it induces activities of CYP1A1, CYP1A2, and CYP1B1 enzymes, which are also involved in the hydroxylation of PCDDs and PCBs [736], in mice and rats [365,734]. Rat hepatocytes show greater rate of TCDD metabolism compared to that in guinea pigs, this feature may underly the intraspecies differences in susceptibility to the toxicity of TCDD [737].

*MoA:* Dioxins are not DNA-reactive, but enhance liver tumor development through epigenetic mechanisms mediated by binding to the aryl hydrocarbon receptor leading to toxicity and enhanced cell proliferation [275,724,738,739,740]. Accordingly, for these events a threshold can be established. For example, NOAELs for hepatocyte proliferation in the NTP bioassays were 0.003 µg/kg at the 14-week interim evaluation and 0.022 µg/kg at 53 weeks for TCDD [721] and between 10 µg and 100 µg for 3,3′,4,4′-tetrachloroazoxybenzene (PCB 126) [722]. The hepatocarcinogenicity of TCDD in rats was greater in females than in males apparently due to the influence of estrogenic hormones [741], although the specific mechanism(s) has not been elucidated. Induction of oxidative damage can also play a role in carcinogenicity of dioxins. These MoAs are considered to be operational in humans [275].

*Human Exposure:* Mean dietary exposure to all dioxins in adults occurs primarily through consumption of food of animal origin, such as meat, dairy products, eggs and some fish, and is estimated to be 0.3–3 pg/kg bw day [275,365,716,717,742]. In 2010–2021 EDI for PCDDs and PCDFs varied from 0.001 pg TEQ/kg bw/day to 74.31 pg WHO-TEQ/day [743] depending on the country and method used for estimation of intake. Per capita intake of dioxins in US population is estimated to be lower (17 to 24 pg per capita) compared to that of European population (29 to 97 pg per capita) [365]. In nursing infants, dietary intake of dioxins can reach up to 53 pg TEQ/kg bw/day for TCDD [742] and over 150 WHO-TEQ/kg bw/day for PCDDs and PCBs [719]. Due to limitations in use, intake of dioxins has substantially reduced over the years.

*Human Effects:* There is no epidemiological evidence that implicates consumption of low-level DCLs-containing foods in human cancer causation [713,744]. Nevertheless, continuing evaluation of highly exposed individuals is strengthening the observations of increased cancer risk with dioxin exposure, in particular for lung cancer, soft-tissue sarcoma and non-Hodgkin lymphoma, although the increases are small for these relatively high exposures [745,746]. In addition to carcinogenicity, exposure to dioxins is associated with a variety of adverse effects, including dermatological effects (chloracne), cardiovascular diseases, endocrine disorders (diabetes, affected thyroid function), reproductive effects, neurological disorders and an increase in hepatic enzymes [365].

*Risk:* TCDD and DLCs have been classified by IARC [275,690] to be human carcinogens (Group 1) (Table 2) based on sufficient epidemiological information, animal carcinogenicity data and strong mechanistic considerations. In 1998, WHO modified a previously established TDI for TCDD from 10 pg/kg bw to a range of 1–4 pg TEQs kg bw/day [747,748], while the SCF [749] and JECFA [365] established TDI for dioxins of 2 and 2.3 pg/kg bw, respectively. In contrast, the US EPA [750] has proposed that dioxin doses in the range of 1 pg/kg might represent a cancer risk. This assessment was criticized as overly conservative [751,752,753,754], and the EPA has yet to issue a reanalysis of the cancer TCDD dose response reassessment.

#### 4.3.2. Food Contact Materials

##### 4.3.2.1. Benzophenone

*Occurrence: Benzophenone* (diphenylketone) (Figure 8(3)) is an aryl ketone that can occur in foods naturally or due to migration from packaging or its use as a food additive [115,365,755,756]. Naturally, benzophenone mainly occurs in grapes at concentrations up to 0.13 mg/kg, it is also a constituent of vanilla (up to 0.48 mg/kg), passion fruit (0.045 mg/kg) and papaya (less than 0.01 mg/kg). Benzophenone can migrate into foodstuff from paperboard packaging when used as photoinitiator for UV printing inks, or from plastic food packaging when used as a UV filter [757,758,759,760]. Concentrations of benzophonone residues migrated into food ranged from 0.01 to over 5 mg/kg, with the highest levels, 7.3 mg/kg, detected in confectionery products with high fat content [115,758]. As a flavoring agent, benzophenone is used at 0.5 to 1.28 mg/kg in non-alcoholic beverages and at 2 mg/kg in foods in general [115].

*Carcinogenicity:* Long-term oral administration of benzophenone in diet up to 1250 ppm (equivalent to 60 mg/kg bw in rats and 160 mg/kg bw in mice) produced some evidence of carcinogenic activity evident by increases in the incidences of mononuclear cell leukemia and renal tubular adenoma in male rats as well as liver tumors in male mice and histiocytic sarcoma in female mice [115,755,756,761,762,763,764].

*Genotoxicity/DNA Binding (Adducts)*: Results of in vitro and in vivo genotoxicity testing for benzophenone were mainly negative [755,756,763,765]. However, in the presence of recombinant human CYP2A6 and NADPH-CYP reductase, benzophenone induced *umu* gene expression in *S. typhimurium*, which is an indicator of DNA damage [766]. Photoactivated benzophenone has been reported to react with DNA in vitro, producing single strand breaks, DNA-protein cross-links and abasic sites [767,768].

*Metabolism*: In rats, benzophenone is metabolized by reduction to benzhydrol or by oxidation to 4-hydroxybenzophenone, these metabolites and a sulphate conjugate of 4-hydroxybenzophenone were also detected in vitro [769,770].

*MoA:* Carcinogenic MoA of benzophenone is not well understood and likely involves multiple mechanisms, including endocrine-disrupting effects and oxidative damage [115]. Thus, benzophenone and its metabolite, 4-hydroxybenzophenone, exhibited estrogenic effects in vitro and in vivo [771,772,773]. In the subchronic and chronic rodent studies, oral administration of benzophenone induced CYP enzymes and consequent hepatocellular hypertrophy [761,762,763], indicating that these changes can be involved in hepatocarcinogenesis. Renal tumors in male rats were associated with the exacerbation of ageing chronic nephropathy, suggesting that this MoA largely contributes to renal tubular proliferation induced by benzophenone [764]. This MoA is not relevant to human renal carcinogenesis [764].

*Human Exposure:* Combined dietary exposures to benzophenone range from 8.5 μg/kg bw/day in adults to 22 μg/kg bw/day in children [755]. Similar findings were made in a study involving Taiwan population, where an average daily doses of benzophenone from dietary exposures were estimated to range from 4.54 to 25.8 μg/kg bw/day [774]. Daily per capita intakes of benzophenone based on its use as a flavoring ingredient were estimated to be 0.2 μg/kg bw/day in US and 0.4 μg/kg bw/day in Europe [365,756]. IARC estimated that dietary exposure to benzophenone from consumption of muscat grapes is approximately 0.3 μg/kg bw/day [115].

*Human Effects:* No data linking benzophenone and increased human cancer risk are currently available [115].

*Risk:* IARC [115] classified benzophenone as possibly carcinogenic to humans (Group 2B) (Table 2), stating that while evidence of rodent carcinogenicity is weak, relevance of carcinogenic MoA to humans cannot be excluded. EFSA estimated TDI for benzophenone of 0.03 mg/kg bw [755], and the current migration limit from packaging into foods is 0.6 mg/kg. Despite conclusions made by JECFA [365] and EFSA [755] that benzophenone poses no safety concerns at current levels of intake when used as a flavoring agent, FDA no longer allows use of synthetic benzophenone as a flavoring substance under the Delaney clause [610].

##### 4.3.2.2. Di(2-ethylhexyl) Phthalate

*Occurrence: Di(2-ethylhexyl) phthalate (DEHP)* (Figure 8(4)) is produced by reaction of 2-ethylhexanol with phthalic anhydride and is primarily used as a plasticizer in the production of polyvinyl chloride [115]. Due to its wide presence in packaging materials, DEHP mainly contaminates food by leaching [775]. Concentrations of DEHP in food range from 0.001 to 7.5 mg/kg, with the highest levels of DEHP, exceeding 0.3 mg/kg and, in some cases, reaching 17 mg/kg, reported in foods with high fat content, namely oils, milk, butter, cheese, mayonnaise, fresh meat and fish products [775,776,777,778,779,780,781]. In soft drinks, DEHP occurs at concentrations ranging from 0.00003 to 0.0035 µg/L [115,782].

*Carcinogenicity:* Administration of DEHP in the diet up to 6000 ppm (equivalent to over 350 mg/kg/day) resulted primarily in development of hepatocellular adenomas and carcinomas in rats and mice of both sexes [68,115,777,783,784,785,786,787]. In addition, higher incidences of benign Leydig cell tumors and pancreatic adenomas were observed after DEHP administration. DEHP also showed tumor promoting activity on hepatocellular lesions induced by NDEA and skin tumors induced by 7,12-dimethylbenz[a]anthracene (DMBA) in mice [788,789].

*Genotoxicity/DNA Binding (Adducts)*: DEHP and its major metabolite, mono(2-ethylhexyl) phthalate, produced mainly negative results in the in vitro genotoxicity tests with and without exogenous metabolic activation system; however, some positive responses were observed in cell transformation and DNA damage assays [115,777,780,784,790,791]. In vivo results were mixed [115,339,792,793] although genotoxic effects were likely secondary to oxidative stress [777,794,795,796,797,798]. DEHP did not covalently bind to liver DNA in mice and rats [799,800,801].

*Metabolism*: In rodents and humans, DEHP is hydrolyzed in the presence of carboxyesterases, in particular pancreatic lipases, to mono(2-ethylhexyl) phthalate, which is further metabolized to phthalic acid and oxidative metabolites, such as mono-(2-ethyl-5-hydroxyhexyl)phthalate, mono-(2-ethyl-5-oxohexyl)phthalate, mono-(2-ethyl-5-carboxypentyl)phthalate and mono-[2-(carboxymethyl)hexyl]phthalate, that can be detected in urine and feces either as glucuronide conjugates or unconjugated [115,802,803,804]. Ito and colleagues [805] reported species differences in the activities of enzymes that participate in the metabolism of DEHP, specifically lipase, UDP-glucuronyltransferase, alcohol dehydrogenase and aldehyde dehydrogenase in various tissues of rats, mice and marmosets. When comparing metabolic activity of human and mouse microsomes, activity of most DEHP-metabolizing enzymes was significantly higher in mice compared to that in humans; however, inter-individual variability varied from 10- to 26-fold [806].

*MoA:* Activation of peroxisome proliferator-activated receptor alpha (PPARα) pathway and downstream events are likely the major MoA involved in the hepatocarcinogenesis produced by phthalates, including DEHP [115,787,807,808]. While PPARα-dependent pathway is not relevant to humans [809], other molecular pathways might be involved in carcinogenicity of DEHP, including activation of nuclear receptors, nuclear factor kappa B (NFκB) and constitutive androstane receptor (CAR) [791,807,810]. Oxidative stress may also play a role. Production of benign testicular tumors is most likely caused by reproductive effects of DEHP, specifically, reduction of testosterone production [616].

*Human Exposure:* Exposure of general population to DEHP occurs mainly through consumption of contaminated foods, including dairy products, meat, cereal, fish and seafood [115,777]. EDI ranges from 0.45 to 3.5 μg/kg bw per day in Europe [780] and from 1 to 30 μg/kg bw per day in USA, with an average of 0.673 μg/kg/day [777,811,812]. Worldwide exposures to DEHP have declined over the years, from 4.40 μg/kg bw/day in years prior to 2000 to 2.23 μg/kg bw/day in 2015–2017, however children still have the highest levels of exposure, reaching 5.50 μg/kg bw/day [813].

*Human Effects:* Only limited data assessing association between human cancer, in particular breast, prostate and thyroid cancers, and exposure specifically to DEHP are available [68,115]. No significant associations between dietary exposure to phthalates and breast cancer was found in a recent population-based study [814].

*Risk:* IARC [115] classified DEHP as possibly carcinogenic to humans (Group 2B) (Table 2). EFSA derived a TDI for phthalates of 0.05 mg/kg bw per day [780].

##### 4.3.2.3. 1,4-dioxane

*Occurrence: 1,4-dioxane* (1,4-diethylene dioxide) is an oxygen-containing single ring molecule (Figure 8(5)), which is mainly used as a solvent and stabilizer [558]. It can occur in some foods, including meat, tomatoes, shrimp and coffee as a natural constituent, as a contact material from food packaging or contaminated water, or as an impurity in food additives, such as polyethylene glycol and polysorbate [815,816,817]. Analysis of food products in Japan, revealed that content of 1,4-dioxane ranged from 3 to 13 µg/kg [818].

*Carcinogenicity:* Administration of 1,4-dioxane in drinking water induced hepatocellular adenomas or carcinomas in rats and mice of both sexes and in male guinea pigs [68,558,815,816,819,820]. Other target organs of carcinogenicity included nasal cavity in rats of both sexes, mammary gland in female and abdominal cavity in male rats, gallbladder in male guinea pigs. Administration of 1,4-dioxane in drinking water at 25,000 ppm to rats of both sexes in a 13-week study induced glutathione S-transferase (GST) placental form-positive hepatocellular foci, which are known preneoplastic lesions [821]. In addition, 1,4-dioxane promoted hepatocellular foci produced by administration of diethylnitrosamine [822].

*Genotoxicity/DNA Binding (Adducts):* 1,4-dioxane was non-genotoxic in vitro, in vivo genotoxicity studies in rodents were also mainly negative although some positive results suggesting weak genotoxicity were observed at high cytotoxic doses exceeding 1500 mg/kg [815,816,823,824,825,826]. More recent studies provide evidence that 1,4-dioxane induced chromosomal breaks, DNA damage and mutagenicity in the liver of rats or mice [827,828,829,830]; however, these studies also used high dose levels of 1,4-dioxane (above 1000 mg/kg) and thus, no clear conclusion concerning genotoxicity of 1,4-dioxane can be made. DNA adductome analysis detected several DNA adducts with unidentified chemical structure after administration of 1,4-dioxane to male rats in the drinking water at 200 and 5000 ppm; however, these adducts could have resulted from oxidative damage, rather than direct covalent binding [831].

*Metabolism:* 1,4-dioxane is mainly metabolized in the presence of mixed-function oxidases to 1,4-dioxane-2-one and then to β-hydroxyethoxyacetic acid, which is excreted in urine in rats and humans [558,815,816,832]. Induction of CYP2B1/2, CYP2C11, and CYP2E1 in the liver and only CYP2E1 in the kidney and nasal mucosa was observed in rats exposed to 1,4-dioxane in the drinking water, while dosing by gavage induced CYP3A activity in the liver [833].

*MoA:* The mechanism(s) of carcinogenicity of 1,4-dioxane has not been elucidated but is unlikely to involve genotoxicity. Studies suggested that hepatocarcinogenicity of 1,4-dioxane likely results from cytotoxicity followed by regenerative hyperplasia, in addition, mitogenic response was suggested as a key initiating event [815,834,835,836,837]. Such effects are threshold-dependent. Oxidative damage might also play a role [838]. Tumors of nasal passages were attributed to inhalation of drinking water containing 1,4-dioxane [815,839]. SCF [817] concluded that since 1,4-dioxane is likely to exert its carcinogenic effects by non-genotoxic mechanisms, use of a threshold approach to determine acceptable levels of exposure is justified.

*Human Exposure:* Dietary exposures to 1,4-dioxane is a minor exposure route, in contrast to inhalation. FDA [840] estimated per capita dietary intake of 1,4-dioxane to be low, averaging at 0.6 µg/person/day. Analyses of Japanese foods revealed an EDI of 1,4-dioxane averaging from 0.44 to 4.5 µg/kg bw/day [818,841]. SCF established that an estimated maximum exposure to 1,4-dioxane as a constituent of food additives, polysorbates, in bread ranges between 0.008 to 0.05 µg/kg bw/day [817].

*Human Effects:* No epidemiological studies investigating association of oral exposure of humans to 1,4-dioxane and cancer are currently available [68,558,816]. Limited occupational studies found no excess of death from cancer associated with 1,4-dioxane exposure [815].

*Risk:* IARC [558] assigned 1,4-dioxane to a group of chemicals which are possibly carcinogenic to human (Group 2B) (Table 2). SCF [817] established that exposure to 1,4-dioxane in food additives is significantly lower than the established NOAEL of 10 mg/kg bw/day, and thus is of no toxicological concern.

##### 4.3.2.4. Methyl Isobutyl Ketone

*Occurrence*: *Methyl isobutyl ketone* (MIBK) (4-methylpentan-2-one) (Figure 8(5)) is produced from acetone by aldol condensation and is used primarily as denaturant and solvent [115,842,843]. It is also a natural constituent of many foods including orange and lemon juice, grapes, papaya, ginger, cooked eggs, meat, milk and cheeses, beer, mushrooms, coffee and tea, as at concentrations ranging from 0.008 to 6.5 mg/kg, and as a flavoring agent in meat products, dairy and non-alcoholic beverages, baked goods and puddings at maximum reported level of 25 mg/kg [115,844]. As a component of adhesive, MIBK can also migrate into foods from packaging at levels around 10 to 12 mg/kg.

*Carcinogenicity*: Currently, no studies assessed carcinogenicity of MIBK following oral exposure. Some evidence of carcinogenic activity were observed in inhalation studies, which reported increased incidences of renal tubule neoplasms in rats and hepatocellular neoplasms in mice at the highest tested dose of 1800 ppm (equivalent to 1725 mg/kg/day for rats and 3171 mg/kg/day for mice) [115,842,844,845,846].

*Genotoxicity/DNA Binding (Adducts)*: MIBK produced overwhelmingly negative results in genotoxicity testing battery in vitro and in vivo [115,843,846,847,848,849] and thus, is not considered to be of concern for genotoxicity [842,844].

*Metabolism*: MIBK is metabolized in vivo by reduction in the presence of alcohol dehydrogenases to 4-hydroxyMIBK and by oxidation in the presence of CYP-dependent monooxygenase to 4-methyl-2-pentanol [843,850,851,852,853]. The letter metabolite was not detected with oral administration [852,854]. MIBK has been shown to induce liver and renal CYPs, potentiating hepato- and nephrotoxicity produced by chloroform and carbon tetrachloride [853,855,856].

*MoA*: In the carcinogenicity bioassay, histopathologic changes observed in the kidneys of rats were characteristic of α_2u_-globulin nephropathy [846], suggesting that α_2u_-globulin-mediated MoA is involved in renal carcinogenesis [275,845,857,858]. This MoA is not considered relevant to humans [73]. MoA underlying hepatocarcinogenicity of MIBK in mice is not well understood. While it potentiated hepatotoxicity and cholestasis produced by other chemicals [853,855,856,859,860] no evidence of hepatotoxicity was observed when MIBK was administered alone [275,843]. A study by Hughes and colleagues [861] suggested involvement of receptor-mediated mechanism, specifically, activation of the CAR/PXR nuclear receptors, which results in hepatocellular proliferation consequently leading to tumor development.

*Human Exposure*: Dietary per capita exposure to MIBK was calculated to be 7 µg/person/day in Europe and 2 µg/person/day in USA [849]. More recent estimations suggest lower levels of intake of 0.02 µg/kg/day [115].

*Human Effects*: No data on human carcinogenicity of MIBK are currently available [115]. Long term exposure in occupational settings was reported to cause cognitive impairment [862].

*Risk*: IARC [275] classified MIBK as possibly carcinogenic to humans (Group 2B) (Table 2). JECFA [849] concluded that at the current levels of intake as a flavoring agent MIBK is unlikely to pose any hazard to human health.

### 4.4. Carcinogens Formed during Processing, Packaging and Storage of Food

Food processing contaminants are generated through cooking practices or as a result of food packaging and storage. Some of the carcinogens belonging to this type were discussed in the section on DNA-reactive carcinogens. Examples of epigenetic processing carcinogens in food include alkylated imidazoles and furan.

#### 4.4.1. Alkylated Imidazoles

*Occurrence: 2-methylimidazole* (2-MI) (Figure 9(1)) and *4-methylimidazole* (4-MI) (Figure 9(2)) are formed during fermentation and cooking by ammoniation of simple sugars [115,863,864,865]. They have been identified as by-products in foods including caramel coloring (Classes III and IV) and caramel-colored syrups, cola, ammoniated molasses, wine, Worcestershire sauce, and soy sauce [115,863,866,867,868,869,870,871]. 4-MI has been also detected in the milk from cows fed ammoniated forage [872,873]. Alkylated imidazoles can be also formed during thermal processing of natural constituents not containing caramel coloring, thus up to 466 µg/kg of 4-MI and up to 135 µg/kg of 2-MI were detected in roasted barley, malt and cocoa powder [874].

*Carcinogenicity*: Both, 2-MI and 4-MI, were carcinogenic in rodent studies [115,875,876,877,878]. Specifically, 2-MI induced thyroid follicular cell hypertrophy in mice and hyperplasia in rats by 15 days [879]. In a 2-year feed study, there was some evidence of carcinogenic activity of 2-MI in male rats based on increased incidences of thyroid gland follicular cell neoplasms and clear evidence of carcinogenic activity in female rats based on increased incidences of thyroid gland follicular cell neoplasms [876,878]. In addition, increased incidences of hepatocellular adenoma in male and female rats may have been related to exposure. In mice, there was some evidence of carcinogenic activity of 2-MI, based on increased incidences of thyroid gland follicular cell adenoma and hepatocellular neoplasms in males and increased incidences of hepatocellular adenoma in females [876,878]. In NTP bioassays, 4-MI fed to groups of male and female mice in diet containing 312 ppm (equivalent to 80 mg/kg bw/day) and greater for 106 weeks, increased incidences of pulmonary alveolar/bronchiolar adenoma in all dosed groups of females, alveolar/bronchiolar carcinoma in males given 1250 ppm, and alveolar/bronchiolar adenoma or carcinoma (combined) in males fed 1250 ppm and in females fed 625 and 1250 ppm [875,877]. In male and female rats fed diets containing 4-MI at up to 2500 ppm to males or 5000 ppm to females (115 and 260 mg/kg bw/day, respectively) for 106 weeks, there was no evidence of carcinogenic activity in males and only equivocal evidence in females based on modest increases in the incidences of mononuclear cell leukemia [875,877].

*Genotoxicity/DNA Binding (Adducts)*: 2-MI and 4-MI were negative in bacterial mutation assays when tested either with or without an exogenous bioactivation system [115,865,880,881]. 2-MI yielded mixed results in vivo for induction of chromosomal damage, as measured by micronucleated erythrocyte frequency and was negative in bone marrow micronucleus tests in rats and mice when administered intraperitoneally three times at 24-h intervals [115]. In a 14-week study of 2-MI; however, a significant exposure-related increase in the frequency of micronucleated erythrocytes was noted in peripheral blood of male and female mice [865,878]. While 4-MI produced SCE, chromosome aberrations and micronuclei induction in human peripheral lymphocytes in vitro [882], no increase in the frequencies of micronucleated erythrocytes was seen in the bone marrow of male rats or mice which were administered 4-MI by intraperitoneal injection, or in peripheral blood samples from male and female mice dosed in feed for 14 weeks [115,865,875,881].

*Metabolism*: 2- and 4-MI are rapidly absorbed and quickly eliminated in the urine mainly unchanged [871,883,884]. Mice cleared 2-MI faster than rats [878]. In rats, 2-MI is distributed to several tissues, including the thyroid [885], while 4-MI is mainly distributed to intestines, liver and kidney [115]. The principal urinary metabolite of 2-MI is the ring oxidized 2-MI, which possesses nucleophilicity [886,887]. Metabolism of 4-MI in rats and mice is similar, and the major metabolite detected in both species was hydroxylated 4-MI [884].

*MoA:* The MoA of 2-MI for induction of thyroid neoplasms likely involves interference with thyroid homeostasis, as described for several other chemicals [888]. 2-MI produced exposure-related reduction in thyroxine (T4) and increases in thyroid-stimulating hormone (TSH) in rats and had a lesser effect on T4 in mice [878]. The decrease in T4 can be attributed to increased hepatic UDP-glucuronyl transferase activity [876], which would lead to increased conjugation and excretion of T4. In response to T4 reduction, pituitary production of TSH increases which stimulates function and growth of the thyroid [888]. Likewise, the MoA of 2-MI for the liver tumors appears to involve a trophic effect on the liver reflected by liver weight and enzyme increases [876]. Both of these MoAs represent adaptive effects [889], which would be anticipated to be reversible. Nevertheless, IARC concluded that relevance of such tumor response in animals to humans cannot be excluded [115]. The MoA of 4-MI in induction of lung tumors in mice remains unclear, but does not involve genotoxicity, cytotoxicity or mitogenicity [115,890,891].

*Human Exposure:* The overall EDI for 4-MI for US population ranges from 0.13 to 0.51 µg/kg bw/day, with cola-type carbonated beverages being the highest contributor [892]. The average dietary intake for 4-MI in Europe was estimated to be between 0.4 to 3.7 µg/kg bw/day [893]. EDI of 4-MI from caramel colors ranges from 6 to 11 µg/kg bw/day for Class III and 7 to 9 µg/kg bw/day for Class IV [871].

*Human Effects:* No epidemiologic studies assessing human cancer risk of 2-MI or 4-MI were found [115].

*Risk:* IARC classifies 2-MI and 4-MI as possibly carcinogenic to humans (Group 2B) (Table 2). JECFA limits level of 4-MI to 200 and 250 mg/kg in caramel colors Classes III and IV, respectively [894]. EFSA suggest ADI of 300 mg/kg bw/day for all classes of caramel color [870]. FDA and EFSA concluded that at levels present in caramel colors 4-MI is not expected to be a concern to human health [871].

#### 4.4.2. Furan

*Occurrence*: *Furan* (oxacyclopentadiene) (Figure 9(3)) is a volatile contaminant formed in some foods during heat treatment techniques such as canning and jarring where the furan cannot escape [895]. The sources for the formation of furan include the oxidation of polyunsaturated fatty acids or the decomposition of carbohydrates or amino acids, but the relative contributions of these processes in actual foods is not known [896,897]. Analysis of approximately 300 food samples found furan levels ranging from nondetectable (below the limits of detection of the method) to 175 ppb [898]. Particularly high levels were found in foods that are roasted (e.g., coffee, cocoa, nuts, toasted bread, popcorn) or heated in closed containers (e.g., canned food, ready meals and baby food) [896,897,898,899].

*Carcinogenicity:* In rodent carcinogenicity studies [68,900,901], furan, administered to rats by gavage at 8 mg/kg bw, 5 days/week, induced a high incidence of cholangiocarcinomas in both males and females, at lower doses these tumors were reclassified as cholangiofibrosis [896,902]. In addition, incidences of mononuclear cell leukemia were increased in both sexes, and in males, a high incidence of hepatocellular neoplasms was also produced, while in females the incidence was moderate. In mice, hepatocellular neoplasms were induced at 8 mg/kg bw/day, 5 days/week [900]. In female mice, increased incidence of hepatocellular altered foci and hepatocellular tumors were preceded by a dose-dependent increase in cell proliferation [903]. At lower dosages (up to 2 mg/kg bw), furan administered to male rats by gavage induced malignant mesotheliomas in the epididymis and testicular tunics, dose-related increases in the incidence of mononuclear cell leukemia and dose-related increasing trend in the incidence of hepatocellular adenomas [902].

*Genotoxicity/DNA Binding (Adducts):* Furan produced some genotoxicity in bacterial and mammalian cells in vitro [896,897,901], and chromosomal aberrations in mice, but was negative in SCE, in the mouse bone marrow erythrocyte micronucleus assay, and did not induce UDS in rats or mice [900,904,905]. In Big Blue rats, furan produced mainly negative responses in micronucleus or mutagenicity assays; however, some DNA damage was reported in the comet assay only at cytotoxic doses [906]. DNA strand breaks reported in rat liver but not in the bone marrow were associated with oxidative stress [907]. DNA-protein crosslinking were observed in turkey embryos after dosing with furan [908]. A major metabolite of furan, cis-2-butene-1,4-dial, is a direct acting mutagen [909], which can covalently bind to DNA in vitro [910], but not in vivo [911]. Thus, some DNA binding observed in liver and kidney DNA was attributed to other furan metabolites [912].

*Metabolism*: Furan undergoes oxidation by CYP2E1 resulting in ring scission and formation of the α-unsaturated dicarbonyl cis-2-butene-1,4-dial [886,913,914], which is likely the toxic metabolite [915].

*MoA:* The MoA of furan carcinogenicity is uncertain, but likely involves chronic toxicity with increased cell proliferation, which results from binding of furan and cis-2-butene-1,4-dial to GSH and proteins [896,897]. Nevertheless, in vivo DNA reactivity has not been rigorously excluded. In addition, there is evidence that oxidative stress and epigenetic alterations play a role [896,916,917,918].

*Human Exposure:* As mentioned above, furan is formed in a variety of heat-treated foods by thermal degradation of natural food constituents. Mean dietary exposure to furan in Europe may be as high as 1.23 and 1.01 μg/kg bw/day for adults and 3- to 12-month-old infants, respectively [896]. In the US, FDA [919] calculations estimated that mean daily furan exposures ranged from 0.26 μg/kg bw/day for adults to 0.41 μg/kg/day for infants consuming baby food and 0.9 μg/kg/day for those consuming infant formula. EDI calculated based on the Dortmund Nutritional and Anthropometric Longitudinally Designed (DONALD) study for consumers of commercially jarred foods ranged between 0.182 and 0.688 μg/kg/day [920].

*Human Effects:* Currently, data on effects if furan in humans is limited, and no association with carcinogenicity of furan in humans has been investigated.

*Risk:* IARC [901] classified furan as possibly carcinogenic to humans (Group 2B) (Table 2). EFSA [896] concluded that exposure to furan indicates health concern, due to uncertainties regarding the MoA of furan carcinogenicity; however, current furan exposures are lower than established MoE of concern.

### 4.5. Food Additives

Food additives are added to food in order to improve or maintain certain characteristics, such as taste, texture, appearance or safety. Some are added to foods directly, while others migrate into foods in trace amounts during packaging, storage or handling [921]. According to FDA regulations established in late 1950s, any direct food additive with a carcinogenic potential should not be added to food; however, current advanced understanding of different mechanisms involved in chemical carcinogenesis puts relevance of such regulations under scrutiny [922,923].

#### Monocyclic Phenolics, Synthetic and Natural

*Occurrence:* The synthetic phenolic antioxidants *butylated hydroxyanisole* (BHA) (FigFigure 10(1)), *butylated hydroxytoluene* (BHT) (Figure 10(2)), *tert-butylhydroquinone* (BHQ) (Figure 10(3)), and *hydroquinone* (HQ) (Figure 10(4)) are widely used as food additives to prevent oxidation of lipids [193,849,924,925,926,927,928]. HQ (Figure 10(4)) can also occurs naturally in food, mainly as a glucose conjugate, 4-hydroxyphenyl-P-glucopyranoside, known as arbutin, but can be also found in the free form [558,929]. Foods rich in arbutin include wheat cereal, bread, coffee and pears, with wheat products and pears having the highest levels of arbutin, 10 and 15 ppm, respectively [929].

*Carcinogenicity:* When fed in the diet, BHA elicited increased forestomach neoplasms in rats, hamsters and mice at doses greater than 2% [193,926,930,931]. BHT produced an increase in mouse lung neoplasms at doses of 0.75% [193,927,931]. BHQ produced no neoplasms in rats or mice when fed up to 5000 ppm, in spite of the positive genotoxicity findings; however, in a 6-week feeding study, preneoplastic lesions and papillomas were observed in forestomach of rats [932,933]. In two-year rodent bioassays, HQ administration by gavage at 25 or 50 mg/kg in water led at both doses to increases in renal tubular adenomas in male rats and mononuclear cell leukemias in female rats [558,934]. While there was no evidence of carcinogenic activity in male mice administered 50 or 100 mg HQ/kg bw in water, 5 days/week by gavage, there was some evidence of carcinogenic activity in female mice, as shown by increases in hepatocellular neoplasms, mainly adenomas, and thyroid follicular cell adenoma at the same doses [558,934].

*Genotoxicity/DNA Binding (Adducts):* The synthetic phenolics are non-genotoxic [924,931,935,936], although BHQ has yielded some positive findings in vitro, but not in vivo [937,938]. No DNA binding was detected in the target tissue, forestomach, of rats administered BHA or its metabolites up to 1000 mg/kg [939]. HQ tested positive in some in vitro and in vivo genotoxicity assays [344,558,940], but did not form adducts with DNA in vivo [345,346].

*Metabolism:* BHA is mainly metabolized to glucuronide and sulphate conjugates or is demethylated to free phenols, including BHQ [926]. BHQ can further undergo either oxidation to a quinone metabolite or GSH conjugation, as has been shown in rats [941]. Metabolism of BHT in vitro, using mouse microsomes, produced quinone methides, while in vivo, it is oxidized at one or both *tert*-butyl group(s) by microsomal oxygenase, followed by conjugation with glucuronide [927]. HQ is metabolized mainly to sulfate and glucuronide conjugates, but a small percentage may be converted to 1,4-benzoquinone, which can be either conjugated with GSH or form DNA adducts in vitro [558].

*MoA:* The MoAs of BHA and BHT involve epigenetic mechanisms ultimately leading to promotion of background neoplasia [924,930,933,942]. A lifetime dose–response study of tumor promoting effect of BHA in the rat forestomach identified positive effects at 6000 ppm and above, and a NOAEL at 3000 ppm [943]. In the case of BHT, its MoA appears to involve infiltration of monocytes into the pulmonary alveoli and stimulation of proliferation of type II pneumocytes [944,945]. The MoA for the kidney tumor induction in male rats by HQ has been suggested to involve cytotoxicity leading to increased cell proliferation and exacerbation of chronic progressive nephropathy [946,947,948,949].

*Human Exposure:* The mean daily intake of BHA varies from 2 to 300 µg/kg bw/day, depending on consumer age, region and estimate methods, and the main sources of exposure, similar to other phenolics, include baked goods, snacks and processed potato products [849,924,926]. EFSA reports [927] a mean dietary exposure to BHT is in the range of 10 to 30 µg/kg bw/day for adults and 10 to 90 µg/kg bw/day for children, while JECFA [849] calculated EDI to be between 700 and 990 µg/kg bw. The EDI for BHQ ranges from 4 to 140 µg/kg bw/day based on poundage data and from 370 to 690 µg/kg bw/day, based on model diets [849]. EFSA [928] determined that exposure to BHQ as a food additive averages to approximately 5 µg/kg bw/day in adults and 257 µg/kg bw/day in children.

*Human Effects:* No epidemiological study has implicated BHA or BHT as human carcinogens [193]. To the contrary, studies has suggested an inverse relationship with cancers of gastrointestinal tract [950,951], which is consistent with demonstrated anticarcinogenicity of monocyclic phenolics against a variety of DNA-reactive carcinogens in animal models [345,951,952]. No evidence for human carcinogenicity was found in studies of occupational exposures or dermatological use of HQ [558,953]. No study of cancer risk with dietary exposures was reported.

*Risk:* IARC [193,558] classified BHA as possibly carcinogenic to humans (Group 2B), while BHT and HQ were considered as not classifiable as to human carcinogenicity (Group 3) (Table 2). BHA and BHT are not considered to pose carcinogenic risks to humans based on a threshold-dependent MoAs that are not relevant to potential human exposure levels [930]. Human exposures are not expected to exceed the ADI values of 0.5, 0.3 and 0.7 mg/kg bw, which have been allocated by JECFA to BHA, BHT and BHQ, respectively [849,954]. EFSA [926,927,928] arrived at a similar conclusion after re-evaluating ADIs for BHA, BHT and BHQ to be 1, 0.25 and 0.7 mg/kg bw/day, respectively, and comparing them to the mean dietary intakes in children and adult populations.

## 5. Food-Borne Chemopreventive Agents

A growing body of evidence suggests that a variety of food-derived constituents may exhibit properties that are preventive and/or protective of cancer formation [1,955,956,957,958,959]. Since 1940, almost half of antitumor drugs have been developed from natural products or their derivatives [960]. In contrast, use of naturally occurring chemicals present in diet for cancer prevention and suppression (also referred to as chemoprevention) remains a highly debatable topic due to the lack of success in clinical trials [961,962,963,964].

The MoA underlying chemopreventive properties of naturally occurring agents varies [955]. Some compounds block tumor induction during the initiation stage, others exhibit inhibitory effects during promotion stage, and certain anticarcinogenic substances affect process of carcinogenesis at multiple points [963,965].

Inhibition of preneoplastic and neoplastic effects produced by established chemical carcinogens (including chemicals discussed in this review), has been described in experimental settings for several food constituents. For example, naturally occurring coumarins found in citrus fruits, tonka beans, parsnip, parsley, cinnamon bark oil and peppermint oil, including simple coumarins (coumarin, limettin) and linear furanocoumarins (imperatorin and isopimpinellin) inhibited formation of pro-mutagenic DNA adducts by DMBA in mouse mammary gland and skin [966,967] and formation of BaP DNA adducts and skin tumors in mice [966]. Significant inhibition of DNA adduct levels produced by the heterocyclic amine, PhIP, in rat colonic tissue has been reported after administration of black tea, constituent of mustard plant and papaya seeds, benzylisothiocyanate, and diterpenes, kahweol and cafestol, found in coffee beans [968]. In addition, kahweol and cafestol prevented covalent DNA binding of AFB_1_ in rat livers [969] and restricted tumor formation and growth caused by DMBA in hamster buccal pouch [970]. The flavonoid, nevadensin, which is present in basil and peppermint, significantly inhibited formation of ME specific DNA adducts and incidences of preneoplastic hepatocellular altered foci in the rat liver [971]. Naturally occurring in turmeric, curcumin has been shown to inhibit initiation and promotion of BaP-induced forestomach tumors and DMBA-induced skin tumors in mice [972] and stomach tumors induced by *N*-methyl-*N*′-nitro-*N*-nitrosoguanidine in rats [973]. Synthetic phenolic antioxidant BHT, which is often added to processed food, and its oxidative metabolites, 2,6-di-tert-butyl-4-hydroxymethylphenol and 2,6-di-tert-butyl-1,4-benzoquinone, inhibited DMBA-DNA adducts formation and tumorigenesis in mammary gland of rats [974]. BHT and BHA were also shown to inhibit the hepatocarcinogenicity of AFB_1_ in rats [952,975,976]. Monocyclic phenolics, HQ, inhibited cancer-initiating effects of 2-acetylaminofluorene, including formation of DNA adducts, cell proliferation and formation of preneoplastic foci, in rat liver [345]. Chlorophyll [977], which is available in green vegetables, as well as constituents of cruciferous vegetables, 5-(2-pyrazinyl)-4-methyl-1,2-dithiol-3-thione (oltipraz) and related 1,2-dithiol-3-thiones and 1,2-dithiol-3-ones [978], inhibited experimental hepatocarcinogenicity of AFB_1_ in rats. Other indoles, including indole-3-carbinol and 3,3′-diindolylmethane, also inhibited DMBA-induced mammary gland tumors in rats and BaP-induced forestomach neoplasms in mice [979]. A number of carotenoids, which are pigments present in yellow-orange vegetables, with some representatives of this class also serving as vitamin A precursors, mitigated bacterial mutagenicity of AFB_1_, BaP, IQ, and cyclophosphamide in *Salmonella typhimurium*, and clastogenicity of BaP and cyclophosphamide in mouse bone marrow micronucleus assay [980], and were shown to decrease risk of cancers in different sites, including skin, lung, liver and colon [981].

One of the major mechanisms believed to be involved in cancer prevention by chemicals present in diet is modification in the activity of enzymes which are involved in xenobiotic metabolism. Thus, inhibition of phase I enzymes blocks bioactivation of pro-carcinogens to reactive metabolites, while induction of phase II enzymes leads to increased detoxication and excretion of carcinogens. For example, the inhibitory effect of naturally occurring coumarins on formation DMBA and BaP DNA adducts, is suggested to be a result of CYP1A1/1B1 inhibition and induction of GST activities [966,967]. Inhibition of tumorigenic effects of a wide array of carcinogens has been also linked to modification of phase I and phase II enzyme activities. Such activity was suggested for constituents of cruciferous vegetables, isothiocyanates (sodium cyanate, tert-butyl isocyanate, phenethyl isothiocyanate, benzyl isothiocyanate and sulforaphane), which inhibited carcinogenicity of ethionine, 2-acetylaminofluorene, 3,3′-diaminobenzidine, m-toluylenediamine and *N*-butyl-*N*-(4-hydroxybutyl)nitrosamine in rat liver, DMBA in rat mammary gland, BaP and 4-(methylnitrosamino)-1-(3-pyridyl)-1-butanone in mouse lung and 1,2-dimethylhydrazine in large intestine of mice [951,982,983,984]. Moreover, consumption of cruciferous vegetables, including broccoli, brussels sprouts and cauliflower, was demonstrated to significantly enhance detoxication and excretion of PhIP present in cooked meat in humans, due to induction of phase I (CYP1A2) and phase II (glucuronidation) metabolism [985]. In addition, isothiocyanate compound, sulforaphane, exhibits anti-inflammatory and pro-apoptotic properties, thus, contributing to chemoprevention [986]. It should be noted, however, that persistent enzyme induction may be undesirable for humans.

In addition to the effects mentioned above, many plant components (often referred to as phytochemicals), such as flavone derivatives, isoflavones, catechins, coumarins, phenylpropanoids, polyfunctional organic acids, phosphatides, tocopherols, ascorbic acid, and carotenes act as antioxidants counteracting formation of ROS and thereby, preventing oxidative stress and oxidative DNA damage [987,988,989]. For example, antioxidant effects of resveratrol were associated with inhibition of formation and promotion of skin and breast tumors induced by DMBA in mice or rats [990]. Evidence suggests that food-derived phytochemicals can potentiate antioxidant effects of each other, emphasizing the importance of whole food diets rich in fruits and vegetables [988].

Elucidation of mechanisms of tumor prevention on the molecular level, led to discovery of various signaling pathways and molecular targets that are affected by chemopreventive agents [955,963,965]. For example, butyrate-containing structured lipids and tributyrin, which can be found in wholegrains, vegetables, fruits, nuts and beans, have been shown to prevent and/or inhibit activation of major oncogenes and induce apoptosis at early stages of hepatocarcinogenesis [991]. Tumor-suppressive activity of tributyrin was enhanced by concurrent administration of folic acid [992]. Resveratrol (3,4′,5-trihydroxy-trans-stilbene), which is present in the grape skins, peanuts and red wine, in addition to antioxidant effects, has been shown to modulate cell-cycle regulating pathways, such as MAPKs and NF-κB/AP-1, inducing apoptosis in carcinoma cell lines [993].

It should be noted, however, that some of the compounds described in this section can demonstrate dual effects in experimental settings. Thus, indole-3-carbinol, BHT and coumarin in addition to chemopreventive properties, under certain conditions, usually involving high exposures, can act as tumor promoters [935,994,995]. Nevertheless, presence of chemopreventive agents in diet and their beneficial effects on cancer prophylaxis in humans warrants further investigation.

## 6. Discussion and Conclusions

A wide variety of carcinogens is present in foods and beverages consumed by humans. Categorization of these as operating through MoAs that are either DNA-reactive or epigenetic reveals important differences. Dietary carcinogens of the DNA-reactive type often produce hazardous effects at much lower doses compared to the epigenetic carcinogens. In addition, they frequently affect multiple target tissues, whereas epigenetic carcinogens often affect no more than two sites, where their MoA is exerted. Most importantly, several DNA-reactive carcinogens found in diet are recognized as causes of human cancer, including aflatoxins, aristolochic acid, benzene, benzo[a]pyrene, ethylene oxide, and preserved food components. Other food-derived DNA-reactive chemicals that are considered likely to contribute to human cancer include nitrosamines from several sources, as well as polycyclic aromatic hydrocarbons and heterocyclic amines formed during processing or cooking of food. In contrast, the only food-borne epigenetic carcinogen considered by some authorities to be associated with increased cancer in humans, although not from low-level food exposure, is dioxin (TCDD). Accordingly, DNA-reactive carcinogens represent a much greater risk than epigenetic carcinogens.

## Figures and Tables

**Table 1:** Sources of detectable carcinogens in food.

Source	Examples ^a^	
* 1. Naturally occurring *		
Plant:	alkenylbenzene derivatives aristolochic acid cycasin ptaquiloside d-limonene	psoralen pyrrolizidine alkaloids pulegone β-myrcene
Microbial/Fungal:	various mycotoxins	
* 2. Contaminants *		
Introduced before processing:	daminozide dioxins	DDT flumequine
Introduced during processing:	trichloroethylene	methylene chloride
Food contact materials:	plastics (polyolefins, polyesters, polystyrene, polyamides, etc.) polymeric coatings	monomers (vinyl chloride, styrene, acrylonitrile)
* 3. Additives *		
Anthropogenic:	α,β-aldehydes butylated hydroxyanisole and butylated hydroxytoluene	hexenal saccharin
* 4. Formed from food components *		
During processing:	acrylamide chloropropanols ethyl carbamate (urethane)	furan various nitrosamines alkylated imidazoles
During packaging:	bisphenol A furan	phthalates
During storage:	benzene	
During cooking:	acrylamide benzo[a]pyrene	various heterocyclic amines
In the body:	nitrosamines and nitrosamides α,β-aldehydes	ethylene oxide

^a^ Many of the agents listed are detectable only at minute levels by highly sensitive analytical techniques.

**Table 2:** Classifications and characteristics of food-borne carcinogens.

Chemical Name	CAS Registry Number	Classification		Carcinogenic Potency (TD_50_, mg/kg/d) ^c^	MoA
		IARC ^a^	NTP ^b^		
* 1. Human carcinogens *					
Aflatoxins		1	1	0.343 (mouse) 0.0032 (rat)	GTX
Aristolochic acid I	313-67-7	1	1	N/A	GTX
Benzene	71-43-2	1	1	77.5 (mouse) 169 (rat)	GTX
Benzo[a]pyrene	50-32-8	1	2	3.47 (mouse) 0.956 (rat)	GTX
Dioxin (TCDD)	1746-01-6	1	1	0.000156 (mouse) 0.0000235 (rat)	EPI
Dioxin-like compounds (PBCs)		1	N/L	N/A	EPI
Ethylene oxide	75-21-8	1	1	63.7 (mouse) 21.3 (rat)	GTX
Methoxsalen with UV A radiation	298-81-7	1	1	32.4 (rat)	GTX
Processed meat		1	N/L	N/A	GTX
Salted fish		1	N/L	N/A	GTX
* 2. Likely to be human carcinogens *					
Acrylamide	79-06-1	2A	2	3.75 (rat)	GTX
2-Amino-3-methylimidazo[4,5-*f*]quinoline	76180-96-6	2A	2	19.6 (mouse) 0.812 (rat)	GTX
*p*,*p*′-Dichlorodiphenyl-trichloroethane (DDT)	50-29-3	2A	2	12.8 (mouse) 84.7 (rat)	EPI
Ethyl carbamate (urethane)	51-79-6	2A	2	16.9 (mouse) 41.3 (rat)	GTX
5-Methoxypsoralen	484-20-8	2A	N/L	N/A	GTX
*N*-nitrosodiethylamine	55-18-5	2A	2	0.0265 (rat)	GTX
Red meat		2A	N/L	N/A	GTX
2-Amino-3,4-dimethylimidazo[4,5-*f*]quinoline	77094-11-2	2B	2	15.5 (mouse)	GTX
2-Amino-3,8-dimethylimidazo[4,5-*f*]quinoline	77500-04-0	2B	2	24.3 (mouse) 1.66 (rat)	GTX
2-Amino-1-methyl-6-phenylimidazo[4,5-*b*]pyridine	105650-23-5	2B	2	33.2 (mouse) 1.78 (rat)	GTX
Benzophenone	119-61-9	2B	N/L	152 (rat) 379 (mouse)	EPI
Bracken fern		2B	N/L	N/A	GTX
Butylated hydroxyanisole	25013-16-5	2B	2	5530 (mouse) 405 (rat)	EPI
3-Chloro-1,2-propanediol	96-24-2	2B	N/L	117 (rat)	Uncertain
Crotonaldehyde	4170-30-3	2B	N/L	4.2 (rat)	GTX
Cycasin	14901-08-7	2B	N/L	N/A	GTX
1,3-Dichloro-2-propanol	96-23-1	2B	N/L	46.4 (rat)	GTX
Di(2-ethylhexyl) phthalate	117-81-7	2B	2	476 (rat) 484 (mouse)	EPI
1,4-Dioxane	123-91-1	2B	2	204 (mouse) 267 (rat)	Uncertain/EPI
Fumonisin B_1_	116355-83-0	2B	N/L	6.79 (mouse) 5.75 (rat)	Uncertain/EPI
Fusarin C	79748-81-5	2B	N/L	N/A	Uncertain/EPI
Furan	110-00-9	2B	2	2.72 (mouse) 0.396 (rat)	EPI
Lasiocarpine	303-34-4	2B	N/L	0.389 (rat)	GTX
Methyl eugenol	93-15-2	2B	2	19.3 (mouse) 19.7 (rat)	GTX
Methylazoxymethanol	592-62-1	2B	N/L	N/A	GTX
2-Methylimidazole	693-98-1	2B	N/L	782 (mouse) 868 (rat)	EPI
4-Methylimidazole	822-36-6	2B	N/L	387 (mouse) 317 (rat)	EPI
Methyl isobutyl ketone	108-10-1	2B	N/L	612 (rat)	EPI
Monocrotaline	315-22-0	2B	N/L	0.94 (rat)	GTX
β-Myrcene	123-35-3	2B	N/L	15,400 (rat)	EPI
*N*-nitrosodiethanolamine	1116-54-7	2B	2	3.17 (rat)	GTX
Ochratoxin A	303-47-9	2B	2	6.41 (mouse) 0.136 (rat)	GTX/EPI
Pickled vegetables		2B	N/L	N/A	GTX
Pulegone	89-82-7	2B	N/L	232 (mouse) 156 (rat)	EPI
Riddelliine	23246-96-0	2B	2	1.97 (mouse) 0.119 (rat)	GTX
Safrole	94-59-7	2B	2	51.3 (mouse) 441 (rat)	GTX
*trans,trans*-2,4-Hexadienal	142-83-6	2B	N/L	176 (mouse) 62.2 (rat)	GTX
* 3. Unknown carcinogenic potential *					
Agaritine ^d^	2757-90-6	3	N/L	N/A	GTX
Butylated hydroxytoluene	128-37-0	3	N/L	653 (mouse)	EPI
Carrageenan (native) ^d^	9000-07-1	3	N/L	N/A	
Chlorate (sodium salt) ^d^	7775-09-9	3	N/L	69.1 (mouse) 0.865 (rat)	EPI
Eugenol ^d^	97-53-0	3	N/L	N/A	
Furfural ^d^	98-01-1	3	N/L	197 (mouse) 683 (rat)	Uncertain
Hydroquinone	123-31-9	3	N/L	225 (mouse) 82.8 (rat)	EPI
Isatidine ^d^	15503-86-3	3	N/L	0.716 (rat)	GTX
d-Limonene ^d^	5989-27-5	3	N/L	204 (rat)	EPI
Malondialdehyde	24382-04-5	3	N/L	14.1 (mouse) 122 (rat)	GTX
Patulin ^d^	149-29-1	3	N/L	N/A	Uncertain
Ptaquiloside	87625-62-5	3	N/L	N/A	GTX
Quercetin ^d^	117-39-5	3	N/L	10.1 (rat)	EPI
Retrorsine ^d^	480-54-6	3	N/L	0.862 (rat)	GTX
Senkirkine ^d^	2318-18-5	3	N/L	1.7 (rat)	GTX
Sodium saccharin ^d^	128-44-9	3	N/L	2140 (rat)	EPI
Symphytine ^d^	22571-95-5	3	N/L	1.91	GTX
Zearalenone ^d^	17924-92-4	3	N/L	39 (mouse)	EPI
* 4. Not classified by IARC/NTP *					
Daminozide ^d^	1596-84-5	N/L	N/L	1030 (mouse) 2500 (rat)	EPI
Estragole	140-67-0	N/L	N/L	51.8 (mouse)	GTX
Genistein ^d^	446-72-0	N/L	N/L	27.1 (rat)	EPI
*N*-methyl-*N*-formylhydrazine ^d^	758-17-8	N/L	N/L	1.37 (mouse)	GTX

^a^ IARC group 1—carcinogenic to humans; group 2A—probably carcinogenic to humans; group 2B—possibly carcinogenic to humans; group 3—not classifiable as to its carcinogenicity to humans; group 4—probably not carcinogenic to humans. Source—Agents Classified by the IARC Monographs, Volumes 1–131 [67] . ^b^ 1—known to be a human carcinogen; 2—reasonably anticipated to be a human carcinogen. Source—NTP Report on Carcinogens, 15th Edition [68] . ^c^ Only rodent data was included for comparison; Source—Lhasa Carcinogenicity Database, https://carcdb.lhasalimited.org/ (accessed on 9 July 2022). ^d^ Not discussed in this review. EPI, epigenetic modifications; GTX, genotoxicity; N/A, not available N/L, not listed.

## Data Availability

Data sharing not applicable. No new data were created or analyzed in this study. Data sharing is not applicable to this article.

## Abbreviations

2-HEX, *Trans*-2-hexenal; 2-MI, 2-methylimidazole; 4-MI, 4-methylimidazole; AC, Acrylamide; AA, Aristolochic acid; AB, Alkenylbenzenes; ADI, Acceptable daily intake; AF, Aflatoxin; BaP, Benzo[a]pyrene; BHA, Butylated hydroxyanisole; BHQ, tert-Butylhydroquinone; BHT, Butylated hydroxytoluene; bw, body weight; BZ, Benzene; CA, Crotonaldehyde; CAR, Constitutive androstane receptor; CP, 3-chloro-1,2-propanediol; CONTAM, Panel on Contaminants in the Food Chain; CYP, Cytochrome P450; DDD, *p*,*p*′-dichlorodiphenyl-dichloroethane; DDE, *p*,*p*′-dichlorodiphenyl-dichloroethylene; DDT, *p*,*p*′-dichlorodiphenyl-trichloroethane; diMeIQx, 2-amino-3,4,8-trimethylimidazo[4,5-*f*]quinoline; DEHP, Di(2-ethylhexyl) phthalate; DMBA, 7,12-dimethylbenz[a]anthracene; DLC, Dioxin-like compound; DP, 1,3-dichloro-2-propanol; EC, Ethyl carbamate; EDI, estimated daily intake; EFSA, European Food Safety Authority; EMA, European Medical Agency; EtO, Ethylene oxide; FAO, Food and Agriculture Organization; FB_1_, Fumonisin B_1_; FC, Fusarin C; FDA, US Food and Drug Administration; FEMA, Flavor and Extract Manufacturers Association; GA, Glycidamide; GSH, Glutathione; GST, Glutathione S-transferase; HCA, Heterocyclic amine; HNE, 4-hydroxynonenal; HQ, Hydroquinone; IARC, International Agency for Research on Cancer; IQ, 2-amino-3-methylimidazo[4,5-*f*]quinoline; JECFA, WHO/FAO Joint Expert Committee of Food Additives; MAM, Methylazoxymethanol; MDA, Malondialdehyde; ME, Methyl eugenol; MeIQ, 2-amino-3,4-dimethylimidazo[4,5-*f*]quinoline; MelQx, 2-amino-3,8-dimethylimidazo[4,5-*f*]quinoline; MIBK, Methyl isobutyl ketone; MoA, Mechanism of action; MoE, Margins of exposure; NDEA, *N*-nitrosodiethylamine; NOAEL, No-observed-adverse-effect-level; NPL, 32P-nucleotide postlabeling; OTA, Ochratoxin A; PA, Pyrrolizidine alkaloid; PAH, Polycyclic aromatic hydrocarbon; PCB, polychlorinated biphenyl; PCDD, polychlorinated dibenzo-para-dioxin; PCDF, polychlorinated dibenzofuran; PhIP, 2-amino-1-methyl-6-phenylimidazo[4,5-*b*]pyridine; PMTDI, Provisional maximum tolerable daily intake; PPARα, peroxisome proliferator-activated receptor alpha; PUFA, Polyunsaturated fatty acids; PUL, Pulegone; ROS, reactive oxygen species; SAF, Safrole; SCE, Sister chromatid exchange; SCF, Scientific Committee on Food; T4, Thyroxine; TCDD, Dioxin; TD_50_, Carcinogenic potency; TDI, Tolerable daily intake; TEF, Toxic equivalency factor; TSH, Thyroid stimulating hormone; UDS, Unscheduled DNA synthesis; WHO, World Health Organization.

## References

[1] National Research Council Committee, on Comparative Toxicity of Naturally Occurring Carcinogens (1996). Carcinogens and Anticarcinogens in the Human Diet: A Comparison of Naturally Occurring and Synthetic Substances.

[2] Williams G.M. (1986). Food-borne carcinogens. Prog. Clin. Biol. Res..

[3] Abnet C.C. (2007). Carcinogenic food contaminants. Cancer Investig..

[4] Sugimura T. (2000). Nutrition and dietary carcinogens. Carcinogenesis.

[5] Jackson L.S. (2009). Chemical food safety issues in the United States: Past, present, and future. J. Agric. Food Chem..

[6] Rietjens I.M.C.M., Michael A., Bolt H.M., Siméon B., Andrea H., Nils H., Christine K., Angela M., Gloria P., Daniel R. (2022). The role of endogenous versus exogenous sources in the exposome of putative genotoxins and consequences for risk assessment. Arch. Toxicol..

[7] Hecht S.S., Hoffmann D. (1998). N-nitroso compounds and man: Sources of exposure, endogenous formation and occurrence in body fluids. Eur. J. Cancer Prev..

[8] Tricker A.R., Preussmann R. (1991). Carcinogenic N-nitrosamines in the diet: Occurrence, formation, mechanisms and carcinogenic potential. Mutat. Res./Genet. Toxicol..

[9] Key T.J., Bradbury K.E., Perez-Cornago A., Sinha R., Tsilidis K.K., Tsugane S. (2020). Diet, nutrition, and cancer risk: What do we know and what is the way forward?. BMJ.

[10] Reddy B.S., Cohen L.A., David McCoy G., Hill P., Weisburger J.H., Wynder E.L. (1980). Nutrition and its relationship to cancer. Adv. Cancer Res..

[11] Clapp R.W., Howe G.K., Jacobs M.M. (2007). Environmental and occupational causes of cancer: A call to act on what we know. Biomed. Pharm..

[12] Doll R., Peto R. (1981). The causes of cancer: Quantitative estimates of avoidable risks of cancer in the United States today. JNCI J. Natl. Cancer Inst..

[13] Rumgay H., Shield K., Charvat H., Ferrari P., Sornpaisarn B., Obot I., Islami F., Lemmens V., Rehm J., Soerjomataram I. (2021). Global burden of cancer in 2020 attributable to alcohol consumption: A population-based study. Lancet Oncol..

[14] Cogliano V.J., Baan R., Straif K., Grosse Y., Lauby-Secretan B., El Ghissassi F., Bouvard V., Benbrahim-Tallaa L., Guha N., Freeman C. (2011). Preventable exposures associated with human cancers. J. Natl. Cancer Inst..

[15] IARC, International Agency for Research on Cancer (2012). Pharmaceuticals. Volume 100 A. A review of human carcinogens. IARC Monogr. Eval. Carcinog. Risks Hum..

[16] Pflaum T., Hausler T., Baumung C., Ackermann S., Kuballa T., Rehm J., Lachenmeier D.W. (2016). Carcinogenic compounds in alcoholic beverages: An update. Arch. Toxicol..

[17] Kobets T., Iatropoulos M.J., Williams G.M. (2019). Mechanisms of DNA-reactive and epigenetic chemical carcinogens: Applications to carcinogenicity testing and risk assessment. Toxicol. Res..

[18] Williams G.M., Iatropoulos M.J., Enzmann H.G., Deschl U., Hayes A., Kruger C.L. (2014). Carcinogenicity of chemicals: Assessment and human extrapolation. Hayes’ Principles and Methods of Toxicology.

[19] Williams G.M. (2001). Mechanisms of chemical carcinogenesis and application to human cancer risk assessment. Toxicology.

[20] Weisburger J.H., Williams G.M. (2000). The distinction between genotoxic and epigenetic carcinogens and implication for cancer risk. Toxicol. Sci..

[21] Williams G.M. (2008). Application of mode-of-action considerations in human cancer risk assessment. Toxicol. Lett..

[22] Williams G.M. (1997). Chemicals with carcinogenic activity in the rodent liver; mechanistic evaluation of human risk. Cancer Lett..

[23] Miller E.C., Miller J.A. (1974). Biochemical mechanisms of chemical carcinogenesis. Mol. Biol. Cancer.

[24] Preston R.J., Williams G.M. (2005). DNA-reactive carcinogens: Mode of action and human cancer hazard. Crit. Rev. Toxicol..

[25] Hartwig A., Arand M., Epe B., Guth S., Jahnke G., Lampen A., Martus H.-J., Monien B., Rietjens I.M.C.M., Schmitz-Spanke S. (2020). Mode of action-based risk assessment of genotoxic carcinogens. Arch. Toxicol..

[26] Hanawalt P. (2003). Functional characterization of global genomic DNA repair and its implications for cancer. Mutat. Res./Rev. Mutat. Res..

[27] Tong C., Fazio M., Williams G.M. (1980). Cell cycle-specific mutagenesis at the hypoxanthine phosphoribosyltransferase locus in adult rat liver epithelial cells. Proc. Natl. Acad. Sci. USA.

[28] Kaufmann W.K., Kaufman D.G. (1993). Cell cycle control, DNA repair and initiation of carcinogenesis. FASEB J..

[29] Pagès V., Fuchs R.P. (2002). How DNA lesions are turned into mutations within cells?. Oncogene.

[30] Vogelstein B., Papadopoulos N., Velculescu V.E., Zhou S., Diaz L.A., Kinzler K.W. (2013). Cancer genome landscapes. Science.

[31] Williams G.M., Iatropoulos M.J., Jeffrey A.M. (2000). Mechanistic basis for nonlinearities and thresholds in rat liver carcinogenesis by the DNA-reactive carcinogens 2-acetylaminofluorene and diethylnitrosamine. Toxicol. Pathol..

[32] Cohen S.M., Arnold L.L. (2011). Chemical carcinogenesis. Toxicol. Sci..

[33] Poirier M.C. (2012). Chemical-induced DNA damage and human cancer risk. Discov. Med..

[34] Paini A., Scholz G., Marin-Kuan M., Schilter B., O’Brien J., van Bladeren P.J., Rietjens I.M.C.M. (2011). Quantitative comparison between in vivo DNA adduct formation from exposure to selected DNA-reactive carcinogens, natural background levels of DNA adduct formation and tumour incidence in rodent bioassays. Mutagenesis.

[35] Hwa Yun B., Guo J., Bellamri M., Turesky R.J. (2020). DNA adducts: Formation, biological effects, and new biospecimens for mass spectrometric measurements in humans. Mass Spectrom. Rev..

[36] Poirier M.C., Beland F.A. (1994). DNA adduct measurements and tumor incidence during chronic carcinogen exposure in rodents. Environ. Health Perspect..

[37] Doerge D.R., Gamboa da Costa G., McDaniel L.P., Churchwell M.I., Twaddle N.C., Beland F.A. (2005). DNA adducts derived from administration of acrylamide and glycidamide to mice and rats. Mutat. Res..

[38] Lafferty J.S., Kamendulis L.M., Kaster J., Jiang J., Klaunig J.E. (2004). Subchronic acrylamide treatment induces a tissue-specific increase in DNA synthesis in the rat. Toxicol. Lett..

[39] Pavanello S., Bollati V., Pesatori A.C., Kapka L., Bolognesi C., Bertazzi P.A., Baccarelli A. (2009). Global and gene-specific promoter methylation changes are related to anti-B[a]PDE-DNA adduct levels and influence micronuclei levels in polycyclic aromatic hydrocarbon-exposed individuals. Int. J. Cancer.

[40] Williams G.M., Iatropoulos M.J., Weisburger J.H. (1996). Chemical carcinogen mechanisms of action and implications for testing methodology. Exp. Toxicol. Pathol..

[41] Phillips D.H., Arlt V.M. (2009). Genotoxicity: Damage to DNA and its consequences. EXS.

[42] Neumann H.-G. (2009). Risk assessment of chemical carcinogens and thresholds. Crit. Rev. Toxicol..

[43] Kobets T., Williams G.M. (2016). Thresholds for hepatocarcinogenicity of DNA-reactive compounds. Thresholds of Genotoxic Carcinogens.

[44] Nohmi T. (2018). Thresholds of genotoxic and non-genotoxic carcinogens. Toxicol. Res..

[45] Kobets T., Williams G.M. (2019). Review of the evidence for thresholds for DNA-reactive and epigenetic experimental chemical carcinogens. Chem. Biol. Interact..

[46] Williams G.M., Iatropoulos M.J., Jeffrey A.M. (2012). Dose-effect relationships for DNA-reactive liver carcinogens. Cellular Response to the Genotoxic Insult: The Question of Threshold for Genotoxic Carcinogens.

[47] EFSA CONTAM Panel, European Food Safety Authority, Panel on Contaminants in the Food Chain (2008). Scientific Opinion of the Panel on Contaminants in the Food Chain on a Request from the European Commission on Polycyclic Aromatic Hydrocarbons in Food.

[48] Williams G.M. (1992). DNA reactive and epigenetic carcinogens. Exp. Toxicol. Pathol..

[49] Klaunig J.E., Kamendulis L.M., Xu Y. (2000). Epigenetic mechanisms of chemical carcinogenesis. Hum. Exp. Toxicol..

[50] Sawan C., Vaissière T., Murr R., Herceg Z. (2008). Epigenetic drivers and genetic passengers on the road to cancer. Mutat. Res./Fundam. Mol. Mech. Mutagen..

[51] Pogribny I.P., Rusyn I., Beland F.A. (2008). Epigenetic aspects of genotoxic and non-genotoxic hepatocarcinogenesis: Studies in rodents. Environ. Mol. Mutagen..

[52] Pogribny I.P., Rusyn I. (2013). Environmental toxicants, epigenetics, and cancer. Adv. Exp. Med. Biol..

[53] Williams G.M., Jeffrey A.M. (2000). Oxidative DNA damage: Endogenous and chemically induced. Regul. Toxicol. Pharmacol..

[54] Cooke M.S., Evans M.D., Dizdaroglu M., Lunec J. (2003). Oxidative DNA damage: Mechanisms, mutation, and disease. FASEB J..

[55] Klaunig J.E., Kamendulis L.M. (2004). The role of oxidative stress in carcinogenesis. Annu. Rev. Pharmacol. Toxicol..

[56] Jones P.A., Baylin S.B. (2007). The epigenomics of cancer. Cell.

[57] Baylin S.B., Jones P.A. (2016). Epigenetic determinants of cancer. Cold Spring Harb. Perspect. Biol..

[58] IARC, International Agency for Research on Cancer (2019). Preamble to the IARC Monographs (Amended January 2019).

[59] Wiltse J., Dellarco V.L. (1996). U.S. Environmental Protection Agency guidelines for carcinogen risk assessment: Past and future. Mutat. Res./Rev. Genet. Toxicol..

[60] Jeffrey A.M., Williams G.M. (2005). Risk assessment of DNA-reactive carcinogens in food. Toxicol. Appl. Pharm..

[61] Felter S.P., Bhat V.S., Botham P.A., Bussard D.A., Casey W., Hayes A.W., Hilton G.M., Magurany K.A., Sauer U.G., Ohanian E.V. (2021). Assessing chemical carcinogenicity: Hazard identification, classification, and risk assessment. Insight from a Toxicology Forum state-of-the-science workshop. Crit. Rev. Toxicol..

[62] Barlow S., Schlatter J. (2010). Risk assessment of carcinogens in food. Toxicol. Appl. Pharm..

[63] Raffaele K., Vulimiri S., Bateson T. (2011). Benefits and barriers to using epidemiology data in environmental risk assessment. Open Epidemiol. J..

[64] Kobets T., Williams G.M. (2018). Chemicals with carcinogenic activity primarily in rodent liver. Comprehensive Toxicology.

[65] Edler L., Hart A., Greaves P., Carthew P., Coulet M., Boobis A., Williams G.M., Smith B. (2014). Selection of appropriate tumour data sets for Benchmark Dose Modelling (BMD) and derivation of a Margin of Exposure (MoE) for substances that are genotoxic and carcinogenic: Considerations of biological relevance of tumour type, data quality and uncertainty assessment. Food Chem. Toxicol..

[66] Rosenkranz H.S. (2004). SAR modeling of genotoxic phenomena: The consequence on predictive performance of deviation from a unity ratio of genotoxicants/non-genotoxicants. Mutat. Res./Genet. Toxicol. Environ. Mutagen..

[67] IARC, International Agency for Research on Cancer (2022). Agents Classified by the IARC Monographs.

[68] NTP, National Toxicology Program (2021). Report on Carcinogens, (RoC).

[69] O’Brien J., Renwick A.G., Constable A., Dybing E., Müller D.J.G., Schlatter J., Slob W., Tueting W., van Benthem J., Williams G.M. (2006). Approaches to the risk assessment of genotoxic carcinogens in food: A critical appraisal. Food Chem. Toxicol..

[70] Benford D., Bolger P.M., Carthew P., Coulet M., DiNovi M., Leblanc J.-C., Renwick A.G., Setzer W., Schlatter J., Smith B. (2010). Application of the Margin of Exposure (MOE) approach to substances in food that are genotoxic and carcinogenic. Food Chem. Toxicol..

[71] Herceg Z. (2007). Epigenetics and cancer: Towards an evaluation of the impact of environmental and dietary factors. Mutagenesis.

[72] Braakhuis H.M., Slob W., Olthof E.D., Wolterink G., Zwart E.P., Gremmer E.R., Rorije E., van Benthem J., Woutersen R., van der Laan J.W. (2018). Is current risk assessment of non-genotoxic carcinogens protective?. Crit. Rev. Toxicol..

[73] Swenberg J.A., Lehman-McKeeman L.D. (1999). alpha 2-Urinary globulin-associated nephropathy as a mechanism of renal tubule cell carcinogenesis in male rats. IARC Sci. Publ..

[74] Williams G.M., Whysner J. (1996). Epigenetic carcinogens: Evaluation and risk assessment. Exp. Toxicol. Pathol..

[75] van den Berg S., Restani P., Boersma M., Delmulle L., Rietjens I. (2011). Levels of genotoxic and carcinogenic compounds in plant food supplements and associated risk assessment. Food Nutr. Sci..

[76] Rietjens I.M., Cohen S.M., Fukushima S., Gooderham N.J., Hecht S., Marnett L.J., Smith R.L., Adams T.B., Bastaki M., Harman C.G. (2014). Impact of structural and metabolic variations on the toxicity and carcinogenicity of hydroxy- and alkoxy-substituted allyl- and propenylbenzenes. Chem. Res. Toxicol..

[77] Eisenreich A., Götz M.E., Sachse B., Monien B.H., Herrmann K., Schäfer B. (2021). Alkenylbenzenes in foods: Aspects impeding the evaluation of adverse health effects. Foods.

[78] JECFA, Joint FAO/WHO Expert Committee on Food Additives (2009). Safety Evaluation of Certain Food Additives. Prepared by the Sixty-Ninth Meeting of the Joint FAO/WHO Expert Committee on Food Additives.

[79] Smith R.L., Adams T.B., Doull J., Feron V.J., Goodman J.I., Marnett L.J., Portoghese P.S., Waddell W.J., Wagner B.M., Rogers A.E. (2002). Safety assessment of allylalkoxybenzene derivatives used as flavouring substances—Methyl eugenol and estragole. Food Chem. Toxicol..

[80] Al-Subeihi A.A., Spenkelink B., Rachmawati N., Boersma M.G., Punt A., Vervoort J., van Bladeren P.J., Rietjens I.M. (2011). Physiologically based biokinetic model of bioactivation and detoxification of the alkenylbenzene methyleugenol in rat. Toxicol. Vitr..

[81] Martati E., Boersma M.G., Spenkelink A., Khadka D.B., van Bladeren P.J., Rietjens I.M., Punt A. (2012). Physiologically based biokinetic (PBBK) modeling of safrole bioactivation and detoxification in humans as compared with rats. Toxicol. Sci..

[82] Rietjens I.M.C.M., Boersma M.G., van der Woude H., Jeurissen S.M.F., Schutte M.E., Alink G.M. (2005). Flavonoids and alkenylbenzenes: Mechanisms of mutagenic action and carcinogenic risk. Mutat. Res./Fundam. Mol. Mech. Mutagen..

[83] Jeurissen S.M.F., Punt A., Boersma M.G., Bogaards J.J.P., Fiamegos Y.C., Schilter B., van Bladeren P.J., Cnubben N.H.P., Rietjens I.M.C.M. (2007). Human cytochrome P450 enzyme specificity for the bioactivation of estragole and related alkenylbenzenes. Chem. Res. Toxicol..

[84] Randerath K., Putman K.L., Randerath E. (1993). Flavor constituents in cola drinks induce hepatic DNA adducts in adult and fetal mice. Biochem. Biophys. Res. Commun..

[85] Kobets T., Duan J.-D., Brunnemann K.D., Etter S., Smith B., Williams G.M. (2016). Structure-activity relationships for DNA damage by alkenylbenzenes in turkey egg fetal liver. Toxicol. Sci..

[86] Kobets T., Cartus A.T., Fuhlbrueck J.A., Brengel A., Stegmüller S., Duan J.-D., Brunnemann K.D., Williams G.M. (2019). Assessment and characterization of DNA adducts produced by alkenylbenzenes in fetal turkey and chicken livers. Food Chem. Toxicol..

[87] IARC, International Agency for Research on Cancer (1987). Overall Evaluations of Carcinogenicity: An Updating of IARC Monographs Volumes 1 to 42.

[88] Kamdem D., Gage D. (1995). Chemical composition of essential oil from the root bark of Sassafras albidum. Planta Med..

[89] IARC, International Agency for Research on Cancer (1976). Some naturally occurring substances. IARC Monographs on the Evaluation of the Carcinogenic Risk of Chemicals to Man. Environ. Pollut..

[90] SCF, Scientific Committee on Food (2002). Opinion of the Scientific Committee on Food on the Safety of the Presence of Safrole (1-allyl-3,4-Methylene Dioxy Benzene) in Flavourings and Other Food Ingredients with Flavouring Properties.

[91] Wiseman R.W., Miller E.C., Miller J.A., Liem A. (1987). Structure-activity studies of the hepatocarcinogenicities of alkenylbenzene derivatives related to estragole and safrole on administration to preweanling male C57BL/6J × C3H/HeJ F1 mice. J. Ethnopharmacol..

[92] Miller E.C., Swanson A.B., Phillips D.H., Fletcher T.L., Liem A., Miller J.A. (1983). Structure-activity studies of the carcinogenicities in the mouse and rat of some naturally occurring and synthetic alkenylbenzene derivatives related to safrole and estragole. Cancer Res..

[93] Daimon H., Sawada S., Asakura S., Sagami F. (1998). In vivo genotoxicity and DNA adduct levels in the liver of rats treated with safrole. Carcinogenesis.

[94] Kevekordes S., Spielberger J., Burghaus C.M., Birkenkamp P., Zietz B., Paufler P., Diez M., Bolten C., Dunkelberg H. (2001). Micronucleus formation in human lymphocytes and in the metabolically competent human hepatoma cell line Hep-G2: Results with 15 naturally occurring substances. Anticancer Res..

[95] Gupta K.P., van Golen K.L., Putman K.L., Randerath K. (1993). Formation and persistence of safrole-DNA adducts over a 10,000-fold dose range in mouse liver. Carcinogenesis.

[96] Randerath K., Haglund R.E., Phillips D.H., Reddy M.V. (1984). 32P-post-labelling analysis of DNA adducts formed in the livers of animals treated with safrole, estragole and other naturally-occurring alkenylbenzenes. I. Adult female CD-1 mice. Carcinogenesis.

[97] Lee J.-M., Liu T.-Y., Wu D.-C., Tang H.-C., Leh J., Wu M.-T., Hsu H.-H., Huang P.-M., Chen J.-S., Lee C.-J. (2005). Safrole–DNA adducts in tissues from esophageal cancer patients: Clues to areca-related esophageal carcinogenesis. Mutat. Res. /Genet. Toxicol. Environ. Mutagen..

[98] Zhou G.D., Moorthy B., Bi J., Donnelly K.C., Randerath K. (2007). DNA adducts from alkoxyallylbenzene herb and spice constituents in cultured human (HepG2) cells. Environ. Mol. Mutagen..

[99] Boberg E.W., Miller E.C., Miller J.A., Poland A., Liem A. (1983). Strong evidence from studies with brachymorphic mice and pentachlorophenol that 1′-sulfoöxysafrole is the major ultimate electrophilic and carcinogenic metabolite of 1′-hydroxysafrole in mouse liver. Cancer Res..

[100] Boberg E.W., Miller E.C., Miller J.A. (1986). The metabolic sulfonation and side-chain oxidation of 3′-hydroxyisosafrole in the mouse and its inactivity as a hepatocarcinogen relative to 1′-hydroxysafrole. Chem. Biol. Interact..

[101] Daimon H., Sawada S., Asakura S., Sagami F. (1997). Inhibition of sulfotransferase affecting in vivo genotoxicity and DNA adducts induced by safrole in rat liver. Teratog. Carcinog. Mutagen..

[102] Beyer J., Ehlers D., Maurer H.H. (2006). Abuse of nutmeg (Myristica Fragrans Houtt.): Studies on the metabolism and the toxicologic detection of its ingredients elemicin, myristicin, and safrole in rat and human urine using gas chromatography/mass spectrometry. Ther. Drug Monit..

[103] Chen C.L., Chi C.W., Chang K.W., Liu T.Y. (1999). Safrole-like DNA adducts in oral tissue from oral cancer patients with a betel quid chewing history. Carcinogenesis.

[104] Chung Y.T., Chen C.L., Wu C.C., Chan S.A., Chi C.W., Liu T.Y. (2008). Safrole-DNA adduct in hepatocellular carcinoma associated with betel quid chewing. Toxicol. Lett..

[105] FDA, Food and Drug Administration (2017). Title 21-Food and Drugs. Chapter I-Food and Drug Administration Department of Health and Human Services, Subchapter B-Food for Human Consumption (Continued). Part 189-Substances Prohibited from Use in Human Food. Subpart C-Substances Generally Prohibited from Direct Addition or Use as Human Food. Sec. 189.180 Safrole.

[106] Commission Regulation, (EU) (2008). Regulation (EC) No 1334/2008 of the European Parliament and of the Council of 16 December 2008 on flavourings and certain food ingredients with flavouring properties for use in and on foods and amending Council Regulation (EEC) No 1601/91, Regulations (EC) No 2232/96 and (EC) No 110/2008 and Directive 2000/13/EC. Off. J. Eur. Union.

[107] Liu T.Y., Chung Y.T., Wang P.F., Chi C.W., Hsieh L.L. (2004). Safrole-DNA adducts in human peripheral blood—An association with areca quid chewing and CYP2E1 polymorphisms. Mutat. Res..

[108] EMA, European Medicines Agency (2019). Public Statement on the Use of Herbal Medicinal Products Containing Estragole.

[109] NTP, National Toxicology Program (2011). Technical Report on the 3-Month Toxicity Studies of Estragole (CAS No. 140-67-0) Administered by Gavage to F344/N Rats and B6C3F1 Mice.

[110] Müller L., Kasper P., Müller-Tegethoff K., Petr T. (1994). The genotoxic potential in vitro and in vivo of the allyl benzene etheric oils estragole, basil oil and trans-anethole. Mutat. Res. Lett..

[111] Ding W., Levy D.D., Bishop M.E., Pearce M.G., Davis K.J., Jeffrey A.M., Duan J.D., Williams G.M., White G.A., Lyn-Cook L.E. (2015). In vivo genotoxicity of estragole in male F344 rats. Environ. Mol. Mutagen..

[112] Ishii Y., Suzuki Y., Hibi D., Jin M., Fukuhara K., Umemura T., Nishikawa A. (2011). Detection and quantification of specific DNA adducts by liquid chromatography-tandem mass spectrometry in the livers of rats given estragole at the carcinogenic dose. Chem. Res. Toxicol..

[113] Anthony A., Caldwell J., Hutt A.J., Smith R.L. (1987). Metabolism of estragole in rat and mouse and influence of dose size on excretion of the proximate carcinogen 1′-hydroxyestragole. Food Chem. Toxicol..

[114] Waddell W.J. (2002). Thresholds of carcinogenicity of flavors. Toxicol. Sci..

[115] IARC, International Agency for Research on Cancer (2013). Some Chemicals Present in Industrial and Consumer Products, Food and Drinking Water.

[116] Moshonas M.G., Shaw P.E. (1978). Compounds new to essential orange oil from fruit treated with abscission chemicals. J. Agric. Food Chem..

[117] NTP, National Toxicology Program (2000). Technical Report on the Toxicology and Carcinogenesis Studies of Methyleugenol (CAS NO. 93-15-2) in F344/N Rats and B6C3F1 Mice (Gavage Studies).

[118] Williams G.M., Iatropoulos M.J., Jeffrey A.M., Duan J.-D. (2013). Methyleugenol hepatocellular cancer initiating effects in rat liver. Food Chem. Toxicol..

[119] Waddell W.J. (2004). Correlation of tumors with DNA adducts from methyl eugenol and tamoxifen in rats. Toxicol. Sci..

[120] Al-Subeihi A.A., Spenkelink B., Punt A., Boersma M.G., van Bladeren P.J., Rietjens I.M. (2012). Physiologically based kinetic modeling of bioactivation and detoxification of the alkenylbenzene methyleugenol in human as compared with rat. Toxicol. Appl. Pharm..

[121] Lutz W., Gaylor D., Conolly R., Lutz R. (2005). Nonlinearity and thresholds in dose–response relationships for carcinogenicity due to sampling variation, logarithmic dose scaling, or small differences in individual susceptibility. Toxicol. Appl. Pharmacol..

[122] Smith B., Cadby P., Leblanc J.C., Setzer R.W. (2010). Application of the Margin of Exposure (MoE) approach to substances in food that are genotoxic and carcinogenic: Example: Methyleugenol, CASRN: 93-15-2. Food Chem. Toxicol..

[123] Chandra S.A., Nolan M.W., Malarkey D.E. (2010). Chemical carcinogenesis of the gastrointestinal tract in rodents: An overview with emphasis on NTP carcinogenesis bioassays. Toxicol. Pathol..

[124] Cohen S., Eisenbrand G., Fukushima S., Gooderham N., Guengerich F., Hecht S., Rietjens I., Harman C., Taylor S. (2018). GRAS 28 flavoring substances. Food Technol..

[125] Hermes L., Römermann J., Cramer B., Esselen M. (2021). Quantitative analysis of β-asarone derivatives in Acorus calamus and herbal food products by HPLC-MS/MS. J. Agric. Food Chem..

[126] Uebel T., Hermes L., Haupenthal S., Müller L., Esselen M. (2021). α-Asarone, β-asarone, and γ-asarone: Current status of toxicological evaluation. J. Appl. Toxicol..

[127] JECFA, Joint FAO/WHO Expert Committee on Food Additives (1981). Toxicological Evaluation of Certain Food Additives.

[128] SCF, Scientific Committee on Food (2002). Opinion of the Scientific Committee on Food on the Presence of β-Asarone in Flavourings and Other Food Ingredients with Flavouring Properties.

[129] Kim S.G., Liem A., Stewart B.C., Miller J.A. (1999). New studies on trans-anethole oxide and trans-asarone oxide. Carcinogenesis.

[130] Berg K., Bischoff R., Stegmüller S., Cartus A., Schrenk D. (2016). Comparative investigation of the mutagenicity of propenylic and allylic asarone isomers in the Ames fluctuation assay. Mutagenesis.

[131] Hasheminejad G., Caldwell J. (1994). Genotoxicity of the alkenylbenzenes α− and β-asarone, myristicin and elemicin as determined by the UDS assay in cultured rat hepatocytes. Food Chem. Toxicol..

[132] Haupenthal S., Berg K., Gründken M., Vallicotti S., Hemgesberg M., Sak K., Schrenk D., Esselen M. (2017). In vitro genotoxicity of carcinogenic asarone isomers. Food Funct..

[133] Stegmüller S., Schrenk D., Cartus A.T. (2018). Formation and fate of DNA adducts of alpha- and beta-asarone in rat hepatocytes. Food Chem. Toxicol..

[134] Cartus A.T., Stegmüller S., Simson N., Wahl A., Neef S., Kelm H., Schrenk D. (2015). Hepatic metabolism of carcinogenic β-asarone. Chem. Res. Toxicol..

[135] Cartus A.T., Schrenk D. (2016). Metabolism of the carcinogen alpha-asarone in liver microsomes. Food Chem. Toxicol..

[136] Cartus A.T., Schrenk D. (2020). Metabolism of carcinogenic alpha-asarone by human cytochrome P450 enzymes. Naunyn Schmiedebergs Arch. Pharm..

[137] Wu J., Zhang X.X., Sun Q.M., Chen M., Liu S.L., Zhang X., Zhou J.Y., Zou X. (2015). β-Asarone inhibits gastric cancer cell proliferation. Oncol. Rep..

[138] Chen M., Zhuang Y.W., Wu C.E., Peng H.Y., Qian J., Zhou J.Y. (2021). β-Asarone suppresses HCT116 colon cancer cell proliferation and liver metastasis in part by activating the innate immune system. Oncol. Lett..

[139] FDA, Food and Drug Administration (2013). Title 21-Food and Drugs. Chapter I-Food and Drug Administration Department of Health and Human Services, Subchapter B-Food for Human Consumption (Continued). Part 189-Substances Prohibited from Use in Human Food. Subpart C-Substances Generally Prohibited from Direct Addition or Use as Human Food. Sec. 189.110 Calamus and Its Derivatives.

[140] EMA, European Medicines Agency (2005). Public Statement on the Risks Associated with the Use of Herbal Products Containing Aristolochia Species.

[141] Arlt V.M., Stiborova M., Schmeiser H.H. (2002). Aristolochic acid as a probable human cancer hazard in herbal remedies: A review. Mutagenesis.

[142] Abdullah R., Diaz L.N., Wesseling S., Rietjens I.M. (2017). Risk assessment of plant food supplements and other herbal products containing aristolochic acids using the margin of exposure (MOE) approach. Food Addit. Contam. Part A Chem. Anal. Control Expo. Risk Assess..

[143] Cui M., Liu Z.H., Qiu Q., Li H., Li L.S. (2005). Tumour induction in rats following exposure to short-term high dose aristolochic acid I. Mutagenesis.

[144] Mengs U. (1988). Tumour induction in mice following exposure to aristolochic acid. Arch. Toxicol..

[145] Zhang H., Cifone M.A., Murli H., Erexson G.L., Mecchi M.S., Lawlor T.E. (2004). Application of simplified in vitro screening tests to detect genotoxicity of aristolochic acid. Food Chem. Toxicol..

[146] Chen R., Zhou C., Cao Y., Xi J., Ohira T., He L., Huang P., You X., Liu W., Zhang X. (2020). Assessment of Pig-a, micronucleus, and comet assay endpoints in Tg.RasH2 mice carcinogenicity study of aristolochic acid I. Environ. Mol. Mutagen..

[147] Bárta F., Dedíková A., Bebová M., Dušková Š., Mráz J., Schmeiser H.H., Arlt V.M., Hodek P., Stiborová M. (2021). Co-exposure to aristolochic acids I and II increases DNA adduct formation responsible for aristolochic acid I-mediated carcinogenicity in rats. Int. J. Mol. Sci..

[148] Abdullah R., Wesseling S., Spenkelink B., Louisse J., Punt A., Rietjens I. (2020). Defining in vivo dose-response curves for kidney DNA adduct formation of aristolochic acid I in rat, mouse and human by an in vitro and physiologically based kinetic modeling approach. J. Appl. Toxicol..

[149] Schmeiser H.H., Schoepe K.B., Wiessler M. (1988). DNA adduct formation of aristolochic acid I and II in vitro and in vivo. Carcinogenesis.

[150] McDaniel L.P., Elander E.R., Guo X., Chen T., Arlt V.M., Mei N. (2012). Mutagenicity and DNA adduct formation by aristolochic acid in the spleen of Big Blue^®^ rats. Environ. Mol. Mutagen..

[151] Mei N., Arlt V.M., Phillips D.H., Heflich R.H., Chen T. (2006). DNA adduct formation and mutation induction by aristolochic acid in rat kidney and liver. Mutat. Res..

[152] Rebhan K., Ertl I.E., Shariat S.F., Grollman A.P., Rosenquist T. (2020). Aristolochic acid and its effect on different cancers in uro-oncology. Curr. Opin. Urol..

[153] Grollman A.P. (2013). Aristolochic acid nephropathy: Harbinger of a global iatrogenic disease. Environ. Mol. Mutagen..

[154] Stiborová M., Frei E., Arlt V.M., Schmeiser H.H. (2008). Metabolic activation of carcinogenic aristolochic acid, a risk factor for Balkan endemic nephropathy. Mutat. Res..

[155] Fernando R.C., Schmeiser H.H., Scherf H.R., Wiessler M. (1993). Formation and persistence of specific purine DNA adducts by 32P-postlabelling in target and non-target organs of rats treated with aristolochic acid I. IARC Sci. Publ..

[156] Feldmeyer N., Schmeiser H.H., Muehlbauer K.R., Belharazem D., Knyazev Y., Nedelko T., Hollstein M. (2006). Further studies with a cell immortalization assay to investigate the mutation signature of aristolochic acid in human p53 sequences. Mutat. Res./Genet. Toxicol. Environ. Mutagen..

[157] Levi M., Guchelaar H.J., Woerdenbag H.J., Zhu Y.P. (1998). Acute hepatitis in a patient using a Chinese herbal tea—A case report. Pharm. World Sci..

[158] Schaneberg B.T., Applequist W.L., Khan I.A. (2002). Determination of aristolochic acid I and II in North American species of Asarum and Aristolochia. Pharmazie.

[159] Gökmen M.R., Lord G.M. (2012). Aristolochic acid nephropathy. BMJ.

[160] Nortier J.L., Martinez M.C., Schmeiser H.H., Arlt V.M., Bieler C.A., Petein M., Depierreux M.F., De Pauw L., Abramowicz D., Vereerstraeten P. (2000). Urothelial carcinoma associated with the use of a Chinese herb (Aristolochia fangchi). N. Engl. J. Med..

[161] Nortier J.L., Vanherweghem J.L. (2002). Renal interstitial fibrosis and urothelial carcinoma associated with the use of a Chinese herb (*Aristolochia fangchi*). Toxicology.

[162] Martena M.J., van der Wielen J.C.A., van de Laak L.F.J., Konings E.J.M., de Groot H.N., Rietjens I.M.C.M. (2007). Enforcement of the ban on aristolochic acids in Chinese traditional herbal preparations on the Dutch market. Anal. Bioanal. Chem..

[163] Bode A.M., Dong Z. (2015). Toxic phytochemicals and their potential risks for human cancer. Cancer Prev. Res..

[164] Sieber S.M., Correa P., Dalgard D.W., McIntire K.R., Adamson R.H. (1980). Carcinogenicity and hepatotoxicity of cycasin and its aglycone methylazoxymethanol acetate in nonhuman primates. J. Natl. Cancer Inst..

[165] Kuniyasu T., Tanaka T., Shima H., Sugie S., Mori H., Takahashi M. (1986). Enhancing effect of cholecystectomy on colon carcinogenesis induced by methylazoxymethanol acetate in hamsters. Dis. Colon Rectum.

[166] Reddy B.S., Maeura Y. (1984). Dose-response studies of the effect of dietary butylated hydroxyanisole on colon carcinogenesis induced by methylazoxymethanol acetate in female CF1 mice. J. Natl. Cancer Inst..

[167] Tanaka T., Shinoda T., Yoshimi N., Niwa K., Iwata H., Mori H. (1989). Inhibitory effect of magnesium hydroxide on methylazoxymethanol acetate-induced large bowel carcinogenesis in male F344 rats. Carcinogenesis.

[168] Lijinsky W., Saavedra J.E., Reuber M.D. (1985). Organ-specific carcinogenesis in rats by methyl- and ethylazoxyalkanes. Cancer Res..

[169] Hoffmann G.R., Morgan R.W. (1984). Review: Putative mutagens and carcinogens in foods. V. Cycad azoxyglycosides. Environ. Mutagen..

[170] Williams G.M., Laspia M.F., Mori H., Hirono I. (1981). Genotoxicity of cycasin in the hepatocyte primary culture/DNA repair test supplemented with beta-glucosidase. Cancer Lett..

[171] Kawai K., Furukawa H., Hirono I. (1995). Genotoxic activity in vivo of the naturally occurring glucoside, cycasin, in the Drosophila wing spot test. Mutat. Res..

[172] Matsushima T., Matsumoto H., Shirai A., Sawamura M., Sugimura T. (1979). Mutagenicity of the naturally occurring carcinogen cycasin and synthetic methylazoxymethanol conjugates in Salmonella typhimurium. Cancer Res..

[173] Cavanna M., Parodi S., Taningher M., Bolognesi C., Sciabà L., Brambilla G. (1979). DNA fragmentation in some organs of rats and mice treated with cycasin. Br. J. Cancer.

[174] Klaus V., Bastek H., Damme K., Collins L.B., Frötschl R., Benda N., Lutter D., Ellinger-Ziegelbauer H., Swenberg J.A., Dietrich D.R. (2017). Time-matched analysis of DNA adduct formation and early gene expression as predictive tool for renal carcinogenesis in methylazoxymethanol acetate treated Eker rats. Arch. Toxicol..

[175] Kisby G.E., Ellison M., Spencer P.S. (1992). Content of the neurotoxins cycasin (methylazoxymethanol beta-D-glucoside) and BMAA (beta-N-methylamino-L-alanine) in cycad flour prepared by Guam Chamorros. Neurology.

[176] Esclaire F., Kisby G., Spencer P., Milne J., Lesort M., Hugon J. (1999). The Guam cycad toxin methylazoxymethanol damages neuronal DNA and modulates tau mRNA expression and excitotoxicity. Exp. Neurol..

[177] Fiala E.S., Sohn O.S., Hamilton S.R. (1987). Effects of chronic dietary ethanol on in vivo and in vitro metabolism of methylazoxymethanol and on methylazoxymethanol-induced DNA methylation in rat colon and liver. Cancer Res..

[178] Sohn O.S., Puz C., Caswell N., Fiala E.S. (1985). Differential susceptibility of rat and guinea pig colon mucosa DNA to methylation by methylazoxymethyl acetate in vivo. Cancer Lett..

[179] Sohn O.S., Fiala E.S., Requeijo S.P., Weisburger J.H., Gonzalez F.J. (2001). Differential effects of CYP2E1 status on the metabolic activation of the colon carcinogens azoxymethane and methylazoxymethanol. Cancer Res..

[180] Kisby G.E., Fry R.C., Lasarev M.R., Bammler T.K., Beyer R.P., Churchwell M., Doerge D.R., Meira L.B., Palmer V.S., Ramos-Crawford A.L. (2011). The cycad genotoxin MAM modulates brain cellular pathways involved in neurodegenerative disease and cancer in a DNA damage-linked manner. PLoS ONE.

[181] Zhang Z.X., Anderson D.W., Mantel N., Román G.C. (1996). Motor neuron disease on Guam: Geographic and familial occurrence, 1956–1985. Acta Neurol. Scand..

[182] Borenstein A.R., Mortimer J.A., Schofield E., Wu Y., Salmon D.P., Gamst A., Olichney J., Thal L.J., Silbert L., Kaye J. (2007). Cycad exposure and risk of dementia, MCI, and PDC in the Chamorro population of Guam. Neurology.

[183] Chang S.S., Chan Y.L., Wu M.L., Deng J.F., Chiu T.F., Chen J.C., Wang F.L., Tseng C.P. (2004). Acute cycas seed poisoning in Taiwan. J. Toxicol. Clin. Toxicol..

[184] Alonso-Amelot M.E., Avendaño M. (2002). Human carcinogenesis and bracken fern: A review of the evidence. Curr. Med. Chem..

[185] Gil da Costa R.M., Bastos M.M., Oliveira P.A., Lopes C. (2012). Bracken-associated human and animal health hazards: Chemical, biological and pathological evidence. J. Hazard. Mater..

[186] Rasmussen L.H. (2021). Presence of the carcinogen ptaquiloside in fern-based food products and traditional medicine: Four cases of human exposure. Curr. Res. Food Sci..

[187] Virgilio A., Sinisi A., Russo V., Gerardo S., Santoro A., Galeone A., Taglialatela-Scafati O., Roperto F. (2015). Ptaquiloside, the major carcinogen of bracken fern, in the pooled raw milk of healthy sheep and goats: An underestimated, global concern of food safety. J. Agric. Food Chem..

[188] Aranha P.C., Hansen H.C., Rasmussen L.H., Strobel B.W., Friis C. (2014). Determination of ptaquiloside and pterosin B derived from bracken (Pteridium aquilinum) in cattle plasma, urine and milk. J. Chromatogr. B Anal. Technol. Biomed. Life Sci..

[189] Francesco B., Giorgio B., Rosario N., Saverio R.F., Francesco G., Romano M., Adriano S., Cinzia R., Antonio T., Franco R. (2011). A new, very sensitive method of assessment of ptaquiloside, the major bracken carcinogen in the milk of farm animals. Food Chem..

[190] Shahin M., Smith B.L., Prakash A.S. (1999). Bracken carcinogens in the human diet. Mutat. Res..

[191] Potter D.M., Baird M.S. (2000). Carcinogenic effects of ptaquiloside in bracken fern and related compounds. Br. J. Cancer.

[192] Prakash A.S., Pereira T.N., Smith B.L., Shaw G., Seawright A.A. (1996). Mechanism of bracken fern carcinogenesis: Evidence for H-ras activation via initial adenine alkylation by ptaquiloside. Nat. Toxins.

[193] IARC, International Agency for Research on Cancer (1986). Some Naturally Occurring and Synthetic Food Components, Furocoumarins and Ultraviolet Radiation. IARC Monographs on the Evaluation of the Carcinogenic Risk of Chemicals to Humans.

[194] Pamukcu A.M., Yalçiner S., Hatcher J.F., Bryan G.T. (1980). Quercetin, a rat intestinal and bladder carcinogen present in bracken fern (*Pteridium aquilinum*). Cancer Res..

[195] Hirono I. (1986). Carcinogenic principles isolated from bracken fern. Crit. Rev. Toxicol..

[196] Hirono I., Aiso S., Yamaji T., Mori H., Yamada K., Niwa H., Ojika M., Wakamatsu K., Kigoshi H., Niiyama K. (1984). Carcinogenicity in rats of ptaquiloside isolated from bracken. Gan.

[197] Hirono I., Ogino H., Fujimoto M., Yamada K., Yoshida Y., Ikagawa M., Okumura M. (1987). Induction of tumors in ACI rats given a diet containing ptaquiloside, a bracken carcinogen. J. Natl. Cancer Inst..

[198] Gil da Costa R.M., Neto T., Estêvão D., Moutinho M., Félix A., Medeiros R., Lopes C., Bastos M., Oliveira P.A. (2020). Ptaquiloside from bracken (*Pteridium* spp.) promotes oral carcinogenesis initiated by HPV16 in transgenic mice. Food Funct..

[199] Mori H., Sugie S., Hirono I., Yamada K., Niwa H., Ojika M. (1985). Genotoxicity of ptaquiloside, a bracken carcinogen, in the hepatocyte primary culture/DNA-repair test. Mutat. Res..

[200] Tourchi-Roudsari M. (2014). Multiple effects of bracken fern under in vivo and in vitro conditions. Asian Pac. J. Cancer Prev..

[201] Nagao T., Saito K., Hirayama E., Uchikoshi K., Koyama K., Natori S., Morisaki N., Iwasaki S., Matsushima T. (1989). Mutagenicity of ptaquiloside, the carcinogen in bracken, and its related illudane-type sesquiterpenes. I. Mutagenicity in *Salmonella typhimurium*. Mutat. Res..

[202] Gil da Costa R.M., Coelho P., Sousa R., Bastos M., Porto B., Teixeira J.P., Malheiro I., Lopes C. (2012). Multiple genotoxic activities of ptaquiloside in human lymphocytes: Aneugenesis, clastogenesis and induction of sister chromatid exchange. Mutat. Res..

[203] Matsuoka A., Hirosawa A., Natori S., Iwasaki S., Sofuni T., Ishidate M. (1989). Mutagenicity of ptaquiloside, the carcinogen in bracken, and its related illudane-type sesquiterpenes. II. Chromosomal aberration tests with cultured mammalian cells. Mutat. Res..

[204] Gomes J., Magalhães A., Michel V., Amado I.F., Aranha P., Ovesen R.G., Hansen H.C., Gärtner F., Reis C.A., Touati E. (2012). Pteridium aquilinum and its ptaquiloside toxin induce DNA damage response in gastric epithelial cells, a link with gastric carcinogenesis. Toxicol. Sci..

[205] Povey A.C., Potter D., O’Connor P.J. (1996). 32P-post-labelling analysis of DNA adducts formed in the upper gastrointestinal tissue of mice fed bracken extract or bracken spores. Br. J. Cancer.

[206] Freitas R.N., O’Connor P.J., Prakash A.S., Shahin M., Povey A.C. (2001). Bracken (Pteridium aquilinum)-induced DNA adducts in mouse tissues are different from the adduct induced by the activated form of the Bracken carcinogen ptaquiloside. Biochem. Biophys. Res. Commun..

[207] Shahin M., Smith B.L., Worral S., Moore M.R., Seawright A.A., Prakash A.S. (1998). Bracken fern carcinogenesis: Multiple intravenous doses of activated ptaquiloside induce DNA adducts, monocytosis, increased TNF alpha levels, and mammary gland carcinoma in rats. Biochem. Biophys. Res. Commun..

[208] Sardon D., de la Fuente I., Calonge E., Perez-Alenza M.D., Castaño M., Dunner S., Peña L. (2005). H-ras immunohistochemical expression and molecular analysis of urinary bladder lesions in grazing adult cattle exposed to bracken fern. J. Comp. Pathol..

[209] Shahin M., Moore M.R., Worrall S., Smith B.L., Seawright A.A., Prakash A.S. (1998). H-ras activation is an early event in the ptaquiloside-induced carcinogenesis: Comparison of acute and chronic toxicity in rats. Biochem. Biophys. Res. Commun..

[210] Gil da Costa R.M., Oliveira P.A., Bastos M.M., Lopes C.C., Lopes C. (2014). Ptaquiloside-induced early-stage urothelial lesions show increased cell proliferation and intact β-catenin and E-cadherin expression. Environ. Toxicol..

[211] Alonso-amelot M.E., Castillo U.F., Smith B.L., Lauren D.R. (1998). Excretion, through milk, of ptaquiloside in bracken-fed cows. A quantitative assessment. Lait.

[212] Alonso-Amelot M.E. (1997). The link between bracken fern and stomach cancer: Milk. Nutrition.

[213] Alonso-Amelot M.E., Avendaño M. (2001). Possible association between gastric cancer and bracken fern in Venezuela: An epidemiologic study. Int. J. Cancer.

[214] Liu R., Li A., Sun A., Kong L. (2004). Preparative isolation and purification of psoralen and isopsoralen from Psoralea corylifolia by high-speed counter-current chromatography. J. Chromatogr. A.

[215] Siskos E.P., Mazomenos B.E., Konstantopoulou M.A. (2008). Isolation and identification of insecticidal components from Citrus aurantium fruit peel extract. J. Agric. Food Chem..

[216] Finkelstein E.V.E., Afek U.Z.I., Gross E., Aharoni N., Rosenberg L., Halevy S. (1994). An outbreak of phytophotodermatitis due to celery. Int. J. Dermatol..

[217] McCloud E.S., Berenbaum M.R., Tuveson R.W. (1992). Furanocoumarin content and phototoxicity of rough lemon (Citrus jambhiri) foliage exposed to enhanced ultraviolet-B (UVB) irradiation. J. Chem. Ecol..

[218] Arigò A., Rigano F., Russo M., Trovato E., Dugo P., Mondello L. (2021). Dietary intake of coumarins and furocoumarins through citrus beverages: A detailed estimation by a HPLC-MS/MS method combined with the Linear Retention Index System. Foods.

[219] Melough M.M., Chun O.K. (2018). Dietary furocoumarins and skin cancer: A review of current biological evidence. Food Chem. Toxicol..

[220] Nagayo K., Way B.H., Tran R.M., Song P.S. (1983). Photocarcinogenicity of 8-methoxypsoralen and aflatoxin B1 with longwave ultraviolet light. Cancer Lett..

[221] Forbes P.D., Davies R.E., Urbach F., Dunnick J.K. (1990). Long-term toxicity of oral 8-methoxypsoralen plus ultraviolet radiation in mice. J. Toxicol. Cutan. Ocul. Toxicol..

[222] NTP, National Toxicology Program (1989). Toxicology and Carcinogenesis Studies of 8-Methoxypsoralen (CAS No. 298-81-7) in F344/N Rats (Gavage Studies).

[223] Müller L., Kasper P., Kersten B., Zhang J. (1998). Photochemical genotoxicity and photochemical carcinogenesis—Two sides of a coin?. Toxicol. Lett..

[224] Chételat A.A., Albertini S., Gocke E. (1996). The photomutagenicity of fluoroquinolones in tests for gene mutation, chromosomal aberration, gene conversion and DNA breakage (Comet assay). Mutagenesis.

[225] Yang A., Chen J., Ma Y., Wang L., Fan Y., He X. (2017). Studies on the metabolites difference of psoralen/isopsoralen in human and six mammalian liver microsomes in vitro by UHPLC-MS/MS. J. Pharm. Biomed. Anal..

[226] Ji L., Lu D., Cao J., Zheng L., Peng Y., Zheng J. (2015). Psoralen, a mechanism-based inactivator of CYP2B6. Chem. Biol. Interact..

[227] Girennavar B., Poulose S.M., Jayaprakasha G.K., Bhat N.G., Patil B.S. (2006). Furocoumarins from grapefruit juice and their effect on human CYP 3A4 and CYP 1B1 isoenzymes. Bioorg. Med. Chem..

[228] Santes-Palacios R., Romo-Mancillas A., Camacho-Carranza R., Espinosa-Aguirre J.J. (2016). Inhibition of human and rat CYP1A1 enzyme by grapefruit juice compounds. Toxicol. Lett..

[229] Zhuang X.M., Zhong Y.H., Xiao W.B., Li H., Lu C. (2013). Identification and characterization of psoralen and isopsoralen as potent CYP1A2 reversible and time-dependent inhibitors in human and rat preclinical studies. Drug Metab. Dispos..

[230] Bickers D.R., Pathak M.A. (1984). Psoralen pharmacology: Studies on metabolism and enzyme induction. Natl. Cancer Inst. Monogr..

[231] Wagstaff D.J. (1991). Dietary exposure to furocoumarins. Regul. Toxicol. Pharm..

[232] Gorgus E., Lohr C., Raquet N., Guth S., Schrenk D. (2010). Limettin and furocoumarins in beverages containing citrus juices or extracts. Food Chem. Toxicol..

[233] Ellis C.R., Elston D.M. (2021). Psoralen-induced phytophotodermatitis. Dermatitis.

[234] Berkley S.F. (1986). Dermatitis in grocery workers associated with high natural concentrations of furanocoumarins in celery. Ann. Intern. Med..

[235] Ljunggren B. (1990). Severe phototoxic burn following celery ingestion. Arch. Derm..

[236] Archier E., Devaux S., Castela E., Gallini A., Aubin F., Le Maître M., Aractingi S., Bachelez H., Cribier B., Joly P. (2012). Carcinogenic risks of psoralen UV-A therapy and narrowband UV-B therapy in chronic plaque psoriasis: A systematic literature review. J. Eur. Acad. Derm. Venereol..

[237] Stern R.S., Liebman E.J., Väkevä L. (1998). Oral psoralen and ultraviolet-A light (PUVA) treatment of psoriasis and persistent risk of nonmelanoma skin cancer. PUVA Follow-up Study. J. Natl. Cancer Inst..

[238] Marley A.R., Li M., Champion V.L., Song Y., Han J., Li X. (2021). The association between citrus consumption and melanoma risk in the UK Biobank. Br. J. Derm..

[239] Moreira R., Pereira D.M., Valentão P., Andrade P.B. (2018). Pyrrolizidine alkaloids: Chemistry, pharmacology, toxicology and food safety. Int. J. Mol. Sci..

[240] JECFA, Joint FAO/WHO Expert Committee on Food Additives (2020). Safety Evaluation of Certain Food Additives and Contaminants: Prepared by the Eightieth Meeting of the Joint FAO/WHO Expert Committee on Food Additives (JECFA).

[241] Robertson J., Stevens K. (2017). Pyrrolizidine alkaloids: Occurrence, biology, and chemical synthesis. Nat. Prod. Rep..

[242] EFSA CONTAM Panel, European Food Safety Authority, Panel on Contaminants in the Food Chain (2011). Scientific Opinion on pyrrolizidine alkaloids in food and feed. EFSA J..

[243] Robertson J., Stevens K. (2014). Pyrrolizidine alkaloids. Nat. Prod. Rep..

[244] Kopp T., Abdel-Tawab M., Mizaikoff B. (2020). Extracting and analyzing pyrrolizidine alkaloids in medicinal plants: A review. Toxins.

[245] Fu P.P., Xia Q., Lin G., Chou M.W. (2004). Pyrrolizidine alkaloids—Genotoxicity, metabolism enzymes, metabolic activation, and mechanisms. Drug Metab. Rev..

[246] Dusemund B., Nowak N., Sommerfeld C., Lindtner O., Schäfer B., Lampen A. (2018). Risk assessment of pyrrolizidine alkaloids in food of plant and animal origin. Food Chem. Toxicol..

[247] IARC, International Agency for Research on Cancer (2002). Some Traditional Herbal Medicines, Some Mycotoxins, Naphthalene and Styrene. IARC Monographs on the Evaluation of Carcinogenic Risks to Humans.

[248] JECFA, Joint FAO/WHO Expert Committee on Food Additives (2016). Evaluation of Certain Food Additives and Contaminants.

[249] EMA, European Medicines Agency (2021). Public Statement on the Use of Herbal Medicinal Products Containing Toxic, Unsaturated Pyrrolizidine Alkaloids (PAs) Including Recommendations Regarding Contamination of Herbal Medicinal Products with Pas.

[250] Fu P.P. (2017). Pyrrolizidine alkaloids: Metabolic activation pathways leading to liver tumor initiation. Chem. Res. Toxicol..

[251] NTP, National Toxicology Program (1978). Bioassay of Lasiocarpine for Possible Carcinogenicity.

[252] NTP, National Toxicology Program (2003). Toxicology and Carcinogenesis Studies of Riddelliine (CAS No. 23246-96-0) in F344/N Rats and B6C3F1 Mice (Gavage Studies).

[253] Chen T., Mei N., Fu P.P. (2010). Genotoxicity of pyrrolizidine alkaloids. J. Appl. Toxicol..

[254] Allemang A., Mahony C., Lester C., Pfuhler S. (2018). Relative potency of fifteen pyrrolizidine alkaloids to induce DNA damage as measured by micronucleus induction in HepaRG human liver cells. Food Chem. Toxicol..

[255] Williams G.M., Mori H., Hirono I., Nagao M. (1980). Genotoxicity of pyrrolizidine alkaloids in the hepatocyte primary culture/DNA-repair test. Mutat. Res..

[256] Mori H., Sugie S., Yoshimi N., Asada Y., Furuya T., Williams G.M. (1985). Genotoxicity of a variety of pyrrolizidine alkaloids in the hepatocyte primary culture-DNA repair test using rat, mouse, and hamster hepatocytes. Cancer Res..

[257] Chou M.W., Yan J., Nichols J., Xia Q., Beland F.A., Chan P.C., Fu P.P. (2004). Correlation of DNA adduct formation and riddelliine-induced liver tumorigenesis in F344 rats and B6C3F1 mice. Cancer Lett..

[258] Wang Y.P., Yan J., Beger R.D., Fu P.P., Chou M.W. (2005). Metabolic activation of the tumorigenic pyrrolizidine alkaloid, monocrotaline, leading to DNA adduct formation in vivo. Cancer Lett..

[259] Xia Q., Zhao Y., Von Tungeln L.S., Doerge D.R., Lin G., Cai L., Fu P.P. (2013). Pyrrolizidine alkaloid-derived DNA adducts as a common biological biomarker of pyrrolizidine alkaloid-induced tumorigenicity. Chem. Res. Toxicol..

[260] Ebmeyer J., Braeuning A., Glatt H., These A., Hessel-Pras S., Lampen A. (2019). Human CYP3A4-mediated toxification of the pyrrolizidine alkaloid lasiocarpine. Food Chem. Toxicol..

[261] Schoch T.K., Gardner D.R., Stegelmeier B.L. (2000). GC/MS/MS detection of pyrrolic metabolites in animals poisoned with the pyrrolizidine alkaloid riddelliine. J. Nat. Toxins.

[262] Yang Y.C., Yan J., Doerge D.R., Chan P.C., Fu P.P., Chou M.W. (2001). Metabolic activation of the tumorigenic pyrrolizidine alkaloid, riddelliine, leading to DNA adduct formation in vivo. Chem. Res. Toxicol..

[263] Wang Y.P., Yan J., Fu P.P., Chou M.W. (2005). Human liver microsomal reduction of pyrrolizidine alkaloid N-oxides to form the corresponding carcinogenic parent alkaloid. Toxicol Lett..

[264] Geburek I., Rutz L., Gao L., Küpper J.H., These A., Schrenk D. (2021). Metabolic pattern of hepatotoxic pyrrolizidine alkaloids in liver cells. Chem. Res. Toxicol..

[265] Fashe M.M., Juvonen R.O., Petsalo A., Räsänen J., Pasanen M. (2015). Species-specific differences in the in vitro metabolism of lasiocarpine. Chem. Res. Toxicol..

[266] EFSA, European Food Safety Authority (2016). Dietary exposure assessment to pyrrolizidine alkaloids in the European population. EFSA J..

[267] Rasenack R., Müller C., Kleinschmidt M., Rasenack J., Wiedenfeld H. (2003). Veno-occlusive disease in a fetus caused by pyrrolizidine alkaloids of food origin. Fetal Diagn. Ther..

[268] Neuman M.G., Cohen L., Opris M., Nanau R.M., Hyunjin J. (2015). Hepatotoxicity of pyrrolizidine alkaloids. J. Pharm. Pharm. Sci..

[269] Habs M., Binder K., Krauss S., Müller K., Ernst B., Valentini L., Koller M. (2017). A balanced risk-benefit analysis to determine human risks associated with pyrrolizidine alkaloids (PA)-The case of tea and herbal infusions. Nutrients.

[270] EFSA CONTAM Panel, European Food Safety Authority, Panel on Contaminants in the Food Chain (2017). Statement on the risks for human health related to the presence of pyrrolizidine alkaloids in honey, tea, herbal infusions and food supplements. EFSA J..

[271] Chen L., Mulder P.P.J., Louisse J., Peijnenburg A., Wesseling S., Rietjens I. (2017). Risk assessment for pyrrolizidine alkaloids detected in (herbal) teas and plant food supplements. Regul. Toxicol. Pharm..

[272] Bullerman L.B., Bianchini A. (2007). Stability of mycotoxins during food processing. Int. J. Food Microbiol..

[273] Marin S., Ramos A.J., Cano-Sancho G., Sanchis V. (2013). Mycotoxins: Occurrence, toxicology, and exposure assessment. Food Chem. Toxicol..

[274] Bryden W.L. (2007). Mycotoxins in the food chain: Human health implications. Asia Pac. J. Clin. Nutr..

[275] IARC, International Agency for Research on Cancer (2012). Chemical Agents and Related Occupations. Review of Human Carcinogens. IARC Monographs on the Evaluation of Carcinogenic Risks to Humans.

[276] JECFA, Joint FAO/WHO Expert Committee on Food Additives (2017). Evaluation of Certain Contaminants in Food.

[277] Wogan G.N., Hecht S.S., Felton J.S., Conney A.H., Loeb L.A. (2004). Environmental and chemical carcinogenesis. Semin. Cancer Biol..

[278] Rushing B.R., Selim M.I. (2019). Aflatoxin B1: A review on metabolism, toxicity, occurrence in food, occupational exposure, and detoxification methods. Food Chem. Toxicol..

[279] Ramsdell H.S., Eaton D.L. (1990). Species susceptibility to aflatoxin B1 carcinogenesis: Comparative kinetics of microsomal biotransformation. Cancer Res..

[280] Wogan G.N., Edwards G.S., Newberne P.M. (1971). Structure-activity relationships in toxicity and carcinogenicity of aflatoxins and analogs. Cancer Res..

[281] EFSA CONTAM Panel, European Food Safety Authority, Panel on Contaminants in the Food Chain (2020). Scientific Opinion on risk assessment of aflatoxins in food. EFSA J..

[282] Robens J.F., Richard J.L. (1992). Aflatoxins in animal and human health. Rev. Environ. Contam. Toxicol..

[283] Choy W.N. (1993). A review of the dose-response induction of DNA adducts by aflatoxin B1 and its implications to quantitative cancer-risk assessment. Mutat. Res..

[284] Williams G.M., Duan J.-D., Brunnemann K.D., Iatropoulos M.J., Vock E., Deschl U. (2014). Chicken fetal liver DNA damage and adduct formation by activation-dependent DNA-reactive carcinogens and related compounds of several structural classes. Toxicol. Sci..

[285] Zhang Y.J., Chen C.J., Haghighi B., Yang G.Y., Hsieh L.L., Wang L.W., Santella R.M. (1991). Quantitation of aflatoxin B1-DNA adducts in woodchuck hepatocytes and rat liver tissue by indirect immunofluorescence analysis. Cancer Res..

[286] Coskun E., Jaruga P., Vartanian V., Erdem O., Egner P.A., Groopman J.D., Lloyd R.S., Dizdaroglu M. (2019). Aflatoxin-guanine DNA adducts and oxidatively induced DNA damage in aflatoxin-treated mice in vivo as measured by Liquid Chromatography-Tandem Mass Spectrometry with Isotope Dilution. Chem. Res. Toxicol..

[287] Bedard L.L., Massey T.E. (2006). Aflatoxin B1-induced DNA damage and its repair. Cancer Lett..

[288] Wang J.S., Groopman J.D. (1999). DNA damage by mycotoxins. Mutat. Res..

[289] Eaton D.L., Gallagher E.P. (1994). Mechanisms of aflatoxin carcinogenesis. Annu. Rev. Pharm. Toxicol..

[290] McQueen C.A., Way B.M., Williams G.M. (1988). Genotoxicity of carcinogens in human hepatocytes: Application in hazard assessment. Toxicol. Appl. Pharmacol..

[291] Theumer M.G., Henneb Y., Khoury L., Snini S.P., Tadrist S., Canlet C., Puel O., Oswald I.P., Audebert M. (2018). Genotoxicity of aflatoxins and their precursors in human cells. Toxicol. Lett..

[292] Marchese S., Polo A., Ariano A., Velotto S., Costantini S., Severino L. (2018). Aflatoxin B1 and M1: Biological properties and their involvement in cancer development. Toxins.

[293] Garner R.C., Martin C.N., Smith J.R., Coles B.F., Tolson M.R. (1979). Comparison of aflatoxin B1 and aflatoxin G1 binding to cellular macromolecules in vitro, in vivo and after peracid oxidation; characterisation of the major nucleic acid adducts. Chem. Biol. Interact..

[294] Guengerich F.P., Johnson W.W., Shimada T., Ueng Y.F., Yamazaki H., Langouët S. (1998). Activation and detoxication of aflatoxin B1. Mutat. Res..

[295] Roebuck B.D., Siegel W.G., Wogan G.N. (1978). In vitro metabolism of aflatoxin B2 by animal and human liver. Cancer Res..

[296] Roebuck B.D., Wogan G.N. (1977). Species comparison of in vitro metabolism of aflatoxin B1. Cancer Res..

[297] Benkerroum N. (2020). Chronic and acute toxicities of aflatoxins: Mechanisms of action. Int. J. Environ. Res. Public Health.

[298] Groopman J.D., Wild C.P., Hasler J., Junshi C., Wogan G.N., Kensler T.W. (1993). Molecular epidemiology of aflatoxin exposures: Validation of aflatoxin-N7-guanine levels in urine as a biomarker in experimental rat models and humans. Environ. Health Perspect..

[299] Wogan G.N., Kensler T.W., Groopman J.D. (2012). Present and future directions of translational research on aflatoxin and hepatocellular carcinoma. A review. Food Addit. Contam. Part A Chem. Anal. Control. Expo. Risk Assess..

[300] Kew M.C. (2003). Synergistic interaction between aflatoxin B1 and hepatitis B virus in hepatocarcinogenesis. Liver Int..

[301] Ross R.K., Yuan J.M., Yu M.C., Wogan G.N., Qian G.S., Tu J.T., Groopman J.D., Gao Y.T., Henderson B.E. (1992). Urinary aflatoxin biomarkers and risk of hepatocellular carcinoma. Lancet.

[302] Fang L., Zhao B., Zhang R., Wu P., Zhao D., Chen J., Pan X., Wang J., Wu X., Zhang H. (2022). Occurrence and exposure assessment of aflatoxins in Zhejiang province, China. Environ. Toxicol. Pharmacol..

[303] FDA, Food and Drug Administration (2021). Compliance Policy Guide Sec. 555.400. Aflatoxins in Human Food.

[304] FDA, Food and Drug Administration (2005). Compliance Policy Guide (CPG) Sec 527.400 Whole Milk, Lowfat Milk, Skim Milk-Aflatoxin M1.

[305] IARC, International Agency for Research on Cancer (1993). Some Naturally Occurring Substances: Food Items and Constituents, Heterocyclic Aromatic Amines and Mycotoxins. IARC Monographs on the Evaluation of Carcinogenic Risk to Humans.

[306] Malir F., Ostry V., Pfohl-Leszkowicz A., Malir J., Toman J. (2016). Ochratoxin A: 50 years of research. Toxins.

[307] EFSA CONTAM Panel, European Food Safety Authority, Panel on Contaminants in the Food Chain (2020). Scientific Opinion on the risk assessment of ochratoxin A in food. EFSA J..

[308] NTP, National Toxicology Program (1989). Toxicology and Carcinogenesis Studies of Ochratoxin A (CAS No. 303-47-9) in F344/N Rats (Gavage Studies).

[309] Mantle P., Kulinskaya E., Nestler S. (2005). Renal tumourigenesis in male rats in response to chronic dietary ochratoxin A. Food Addit. Contam..

[310] Mally A. (2012). Ochratoxin A and mitotic disruption: Mode of action analysis of renal tumor formation by Ochratoxin A. Toxicol. Sci..

[311] Pfohl-Leszkowicz A., Manderville R.A. (2007). Ochratoxin A: An overview on toxicity and carcinogenicity in animals and humans. Mol. Nutr. Food Res..

[312] Pfohl-Leszkowicz A., Manderville R.A. (2012). An update on direct genotoxicity as a molecular mechanism of ochratoxin a carcinogenicity. Chem. Res. Toxicol..

[313] Mally A., Dekant W. (2005). DNA adduct formation by ochratoxin A: Review of the available evidence. Food Addit. Contam..

[314] Tozlovanu M., Faucet-Marquis V., Pfohl-Leszkowicz A., Manderville R.A. (2006). Genotoxicity of the hydroquinone metabolite of ochratoxin A:  structure-activity relationships for covalent DNA adduction. Chem. Res. Toxicol..

[315] Mantle P.G., Faucet-Marquis V., Manderville R.A., Squillaci B., Pfohl-Leszkowicz A. (2010). Structures of covalent adducts between DNA and ochratoxin a: A new factor in debate about genotoxicity and human risk assessment. Chem. Res. Toxicol..

[316] Kamp H.G., Eisenbrand G., Janzowski C., Kiossev J., Latendresse J.R., Schlatter J., Turesky R.J. (2005). Ochratoxin A induces oxidative DNA damage in liver and kidney after oral dosing to rats. Mol. Nutr. Food Res..

[317] Arbillaga L., Azqueta A., van Delft J.H.M., López de Cerain A. (2007). In vitro gene expression data supporting a DNA non-reactive genotoxic mechanism for ochratoxin A. Toxicol. Appl. Pharmacol..

[318] Czakai K., Müller K., Mosesso P., Pepe G., Schulze M., Gohla A., Patnaik D., Dekant W., Higgins J.M.G., Mally A. (2011). Perturbation of mitosis through inhibition of histone acetyltransferases: The key to Ochratoxin A toxicity and carcinogenicity?. Toxicol. Sci..

[319] Rásonyi T., Schlatter J., Dietrich D.R. (1999). The role of α2u-globulin in ochratoxin A induced renal toxicity and tumors in F344 rats. Toxicol. Lett..

[320] Pfohl-Leszkowicz A., Pinelli E., Bartsch H., Mohr U., Castegnaro M. (1998). Sex- and strain-specific expression of cytochrome P450s in Ochratoxin A-induced genotoxicity and carcinogenicity in rats. Mol. Carcinog..

[321] Heussner A.H., Bingle L.E. (2015). Comparative ochratoxin toxicity: A review of the available data. Toxins.

[322] JECFA, Joint FAO/WHO Expert Committee on Food Additives (2007). Evaluation of Certain Food Additives and Contaminants.

[323] McNeal T.P., Nyman P.J., Diachenko G.W., Hollifield H.C. (1993). Survey of benzene in foods by using headspace concentration techniques and capillary gas chromatography. J. AOAC Int..

[324] IARC, International Agency for Research on Cancer (2018). Benzene. IARC Monographs on the Evaluation of Carcinogenic Risks to Humans.

[325] Salviano Dos Santos V.P., Medeiros Salgado A., Guedes Torres A., Signori Pereira K. (2015). Benzene as a chemical hazard in processed foods. Int. J. Food Sci..

[326] Smith B., Cadby P., DiNovi M., Setzer R.W. (2010). Application of the Margin of Exposure (MoE) approach to substances in food that are genotoxic and carcinogenic: Example: Benzene, CAS: 71-43-2. Food Chem. Toxicol..

[327] Gardner L.K., Lawrence G.D. (1993). Benzene production from decarboxylation of benzoic acid in the presence of ascorbic acid and a transition-metal catalyst. J. Agric. Food Chem..

[328] Jickells S.M., Crews C., Castle L., Gilbert J. (1990). Headspace analysis of benzene in food contact materials and its migration into foods from plastics cookware. Food Addit. Contam..

[329] Meadows M. (2006). Benzene in beverages. FDA Consum..

[330] Nyman P.J., Diachenko G.W., Perfetti G.A., McNeal T.P., Hiatt M.H., Morehouse K.M. (2008). Survey results of benzene in soft drinks and other beverages by headspace gas chromatography/mass spectrometry. J. Agric. Food Chem..

[331] Snyder R., Witz G., Goldstein B.D. (1993). The toxicology of benzene. Environ. Health Perspect..

[332] Maltoni C., Cotti G., Valgimigli L., Mandrioli A. (1982). Zymbal gland carcinomas in rats following exposure to benzene by inhalation. Am. J. Ind. Med..

[333] NTP, National Toxicology Program (1986). Toxicology and Carcinogenesis Studies of Benzene (CAS No. 71-43-2) in F344/N Rats and B6C3F1 Mice (Gavage Studies).

[334] Whysner J., Reddy M.V., Ross P.M., Mohan M., Lax E.A. (2004). Genotoxicity of benzene and its metabolites. Mutat. Res..

[335] Wetmore B.A., Struve M.F., Gao P., Sharma S., Allison N., Roberts K.C., Letinski D.J., Nicolich M.J., Bird M.G., Dorman D.C. (2008). Genotoxicity of intermittent co-exposure to benzene and toluene in male CD-1 mice. Chem. Biol. Interact..

[336] Tuo J., Loft S., Thomsen M.S., Poulsen H.E. (1996). Benzene-induced genotoxicity in mice in vivo detected by the alkaline comet assay: Reduction by CYP2E1 inhibition. Mutat. Res..

[337] Provost G.S., Mirsalis J.C., Rogers B.J., Short J.M. (1996). Mutagenic response to benzene and tris(2,3-dibromopropyl)-phosphate in the lambda lacI transgenic mouse mutation assay: A standardized approach to in vivo mutation analysis. Environ. Mol. Mutagen..

[338] Salem E., El-Garawani I., Allam H., El-Aal B.A., Hegazy M. (2018). Genotoxic effects of occupational exposure to benzene in gasoline station workers. Ind. Health.

[339] Kitamoto S., Matsuyama R., Uematsu Y., Ogata K., Ota M., Yamada T., Miyata K., Kimura J., Funabashi H., Saito K. (2015). Genotoxicity evaluation of benzene, di(2-ethylhexyl) phthalate, and trisodium ethylenediamine tetraacetic acid monohydrate using a combined rat comet/micronucleus assays. Mutat. Res. Genet. Toxicol. Environ. Mutagen..

[340] Grigoryan H., Edmands W.M.B., Lan Q., Carlsson H., Vermeulen R., Zhang L., Yin S.N., Li G.L., Smith M.T., Rothman N. (2018). Adductomic signatures of benzene exposure provide insights into cancer induction. Carcinogenesis.

[341] Li G., Wang C., Xin W., Yin S. (1996). Tissue distribution of DNA adducts and their persistence in blood of mice exposed to benzene. Environ. Health Perspect..

[342] Bodell W.J., Pathak D.N., Lévay G., Ye Q., Pongracz K. (1996). Investigation of the DNA adducts formed in B6C3F1 mice treated with benzene: Implications for molecular dosimetry. Environ. Health Perspect..

[343] Reddy M.V., Blackburn G.R., Schreiner C.A., Mehlman M.A., Mackerer C.R. (1989). 32P analysis of DNA adducts in tissues of benzene-treated rats. Environ. Health Perspect..

[344] Gaskell M., McLuckie K.I.E., Farmer P.B. (2005). Genotoxicity of the benzene metabolites para-benzoquinone and hydroquinone. Chem. Biol. Interact..

[345] Williams G.M., Iatropoulos M.J., Jeffrey A.M., Duan J.-D. (2007). Inhibition by dietary hydroquinone of acetylaminofluorene induction of initiation of rat liver carcinogenesis. Food Chem. Toxicol..

[346] English J.C., Hill T., O’Donoghue J.L., Reddy M.V. (1994). Measurement of nuclear DNA Modification by 32P-postlabeling in the kidneys of male and female Fischer 344 rats after multiple gavage doses of hydroquinone. Toxicol. Sci..

[347] Gaskell M., McLuckie K.I., Farmer P.B. (2004). Comparison of the mutagenic activity of the benzene metabolites, hydroquinone and para-benzoquinone in the supF forward mutation assay: A role for minor DNA adducts formed from hydroquinone in benzene mutagenicity. Mutat. Res..

[348] Linhart I., Mikes P., Frantík E., Mráz J. (2011). DNA adducts formed from p-benzoquinone, an electrophilic metabolite of benzene, are extensively metabolized in vivo. Chem. Res. Toxicol..

[349] Xie Z., Zhang Y., Guliaev A.B., Shen H., Hang B., Singer B., Wang Z. (2005). The p-benzoquinone DNA adducts derived from benzene are highly mutagenic. DNA Repair..

[350] Kolachana P., Subrahmanyam V.V., Meyer K.B., Zhang L., Smith M.T. (1993). Benzene and its phenolic metabolites produce oxidative DNA damage in HL60 cells in vitro and in the bone marrow in vivo. Cancer Res..

[351] Snyder R. (2012). Leukemia and benzene. Int. J. Environ. Res. Public Health.

[352] Wallace L. (1996). Environmental exposure to benzene: An update. Environ. Health Perspect..

[353] Weisel C.P. (2010). Benzene exposure: An overview of monitoring methods and their findings. Chem. Biol. Interact..

[354] Kim S.R., Halden R.U., Buckley T.J. (2007). Volatile organic compounds in human milk:  Methods and measurements. Environ. Sci. Technol..

[355] Medeiros Vinci R., Jacxsens L., Van Loco J., Matsiko E., Lachat C., de Schaetzen T., Canfyn M., Van Overmeire I., Kolsteren P., De Meulenaer B. (2012). Assessment of human exposure to benzene through foods from the Belgian market. Chemosphere.

[356] Cheasley R., Keller C.P., Setton E. (2017). Lifetime excess cancer risk due to carcinogens in food and beverages: Urban versus rural differences in Canada. Can. J. Public Health.

[357] Galbraith D., Gross S.A., Paustenbach D. (2010). Benzene and human health: A historical review and appraisal of associations with various diseases. Crit. Rev. Toxicol..

[358] JECFA, Joint FAO/WHO Expert Committee on Food Additives (1980). Evaluation of Certain Food Additives Prepared by the Twenty-third Meeting of the Joint FAO/WHO Expert Committee on Food Additives (JECFA).

[359] Lachenmeier D.W., Reusch H., Sproll C., Schoeberl K., Kuballa T. (2008). Occurrence of benzene as a heat-induced contaminant of carrot juice for babies in a general survey of beverages. Food Addit. Contam. Part A Chem. Anal. Control Expo. Risk Assess..

[360] Hamlet C.G., Sadd P.A., Crews C., Velíšek J., Baxter D.E. (2002). Occurrence of 3-chloro-propane-1,2-diol (3-MCPD) and related compounds in foods: A review. Food Addit. Contam..

[361] Andres S., Appel K.E., Lampen A. (2013). Toxicology, occurrence and risk characterisation of the chloropropanols in food: 2-monochloro-1,3-propanediol, 1,3-dichloro-2-propanol and 2,3-dichloro-1-propanol. Food Chem. Toxicol..

[362] EFSA CONTAM Panel, European Food Safety Authority, Panel on Contaminants in the Food Chain (2018). Update of the risk assessment on 3-monochloropropanediol and its fatty acid esters. EFSA J..

[363] Crews C., Hasnip S., Chapman S., Hough P., Potter N., Todd J., Brereton P., Matthews W. (2003). Survey of chloropropanols in soy sauces and related products purchased in the UK in 2000 and 2002. Food Addit. Contam..

[364] Nyman P.J., Diachenko G.W., Perfetti G.A. (2003). Survey of chloropropanols in soy sauces and related products. Food Addit. Contam..

[365] JECFA, Joint FAO/WHO Expert Committee on Food Additives (2002). Safety Evaluation of Certain Food Additives and Contaminants.

[366] Korte R., Schulz S., Brauer B. (2021). Chloropropanols (3-MCPD, 1,3-DCP) from food contact materials: GC-MS method improvement, market survey and investigations on the effect of hot water extraction. Food Addit. Contam. Part A Chem. Anal. Control Expo. Risk Assess..

[367] Becalski A., Zhao T., Breton F., Kuhlmann J. (2016). 2- and 3-Monochloropropanediols in paper products and their transfer to foods. Food Addit. Contam. Part A Chem. Anal. Control Expo. Risk Assess..

[368] NTP, National Toxicology Program (2005). 1,3-Dichloro-2-Propanol [CAS No. 96-23-1]: Review of Toxicological Literature.

[369] JECFA, Joint FAO/WHO Expert Committee on Food Additives (2007). 1,3-Dichloro-2-propanol (addendum). Safety Evaluation of Certain Food Additives and Contaminants.

[370] El Ramy R., Ould Elhkim M., Lezmi S., Poul J.M. (2007). Evaluation of the genotoxic potential of 3-monochloropropane-1,2-diol (3-MCPD) and its metabolites, glycidol and β-chlorolactic acid, using the single cell gel/comet assay. Food Chem. Toxicol..

[371] Aasa J., Törnqvist M., Abramsson-Zetterberg L. (2017). Measurement of micronuclei and internal dose in mice demonstrates that 3-monochloropropane-1,2-diol (3-MCPD) has no genotoxic potency in vivo. Food Chem. Toxicol..

[372] Lynch B.S., Bryant D.W., Hook G.J., Nestmann E.R., Munro I.C. (1998). Carcinogenicity of monochloro-1,2-propanediol (α-chlorohydrin, 3-MCPD). Int. J. Toxicol..

[373] Buhrke T., Voss L., Briese A., Stephanowitz H., Krause E., Braeuning A., Lampen A. (2018). Oxidative inactivation of the endogenous antioxidant protein DJ-1 by the food contaminants 3-MCPD and 2-MCPD. Arch. Toxicol..

[374] Abt E., Incorvati V., Robin L.P., Redan B.W. (2021). Occurrence of ethyl carbamate in foods and beverages: Review of the formation mechanisms, advances in analytical methods, and mitigation strategies. J. Food Prot..

[375] Ough C.S., Crowell E.A., Gutlove B.R. (1988). Carbamyl compound reactions with ethanol. Am. J. Enol. Vitic..

[376] Gowd V., Su H., Karlovsky P., Chen W. (2018). Ethyl carbamate: An emerging food and environmental toxicant. Food Chem..

[377] IARC, International Agency for Research on Cancer (2010). Alcohol Consumption and Ethyl Carbamate. IARC Monographs on the Evaluation of Carcinogenic Risks to Humans.

[378] JECFA, Joint FAO/WHO Expert Committee on Food Additives (2006). Safety Evaluation of Certain Food Contaminants.

[379] EFSA CONTAM Panel, European Food Safety Authority, Panel on Contaminants in the Food Chain (2007). Opinion of the Scientific Panel on Contaminants in the Food chain on a request from the European Commission on ethyl carbamate and hydrocyanic acid in food and beverages. EFSA J..

[380] Choi B., Koh E. (2021). Effect of fruit thermal processing on ethyl carbamate content in maesil (Prunus mume) liqueur. Food Sci. Biotechnol..

[381] NTP, National Toxicology Program (2004). Toxicology and Carcinogenesis Studies of Urethane, Ethanol and Urethane/Ethanol (Urethane, CAS No. 51-79-6; Ethanol CAS No. 64-17-5) in B6C3F1 Mice (Drinking Water Studies).

[382] Chan P.C. (1996). NTP Technical Report on Toxicity Studies of Urethane in Drinking Water and Urethane in 5% Ethanol Administered to F344/N Rats and B6C3F1 Mice.

[383] Hübner P., Groux P.M., Weibel B., Sengstag C., Horlbeck J., Leong-Morgenthaler P.M., Lüthy J. (1997). Genotoxicity of ethyl carbamate (urethane) in Salmonella, yeast and human lymphoblastoid cells. Mutat. Res..

[384] Allen J.W., Stoner G.D., Pereira M.A., Backer L.C., Sharief Y., Hatch G.G., Campbell J.A., Stead A.G., Nesnow S. (1986). Tumorigenesis and genotoxicity of ethyl carbamate and vinyl carbamate in rodent cells. Cancer Res..

[385] Sotomayor R.E., Washington M.C. (1996). Formation of etheno and oxoethyl adducts in liver DNA from rats exposed subchronically to urethane in drinking water and ethanol. Cancer Lett..

[386] Fernando R.C., Nair J., Barbin A., Miller J.A., Bartsch H. (1996). Detection of 1,N6-ethenodeoxyadenosine and 3,N4-ethenodeoxycytidine by immunoaffinity/32P-postlabelling in liver and lung DNA of mice treated with ethyl carbamate (urethane) or its metabolites. Carcinogenesis.

[387] Barbin A. (1998). Formation of DNA etheno adducts in rodents and humans and their role in carcinogenesis. Acta Biochim. Pol..

[388] Forkert P.G. (2010). Mechanisms of lung tumorigenesis by ethyl carbamate and vinyl carbamate. Drug Metab. Rev..

[389] Park K.K., Liem A., Stewart B.C., Miller J.A. (1993). Vinyl carbamate epoxide, a major strong electrophilic, mutagenic and carcinogenic metabolite of vinyl carbamate and ethyl carbamate (urethane). Carcinogenesis.

[390] Hoffler U., El-Masri H.A., Ghanayem B.I. (2003). Cytochrome P450 2E1 (CYP2E1) is the principal enzyme responsible for urethane metabolism: Comparative studies using CYP2E1-null and wild-type mice. J. Pharm. Exp..

[391] Guengerich F.P., Kim D.H. (1991). Enzymatic oxidation of ethyl carbamate to vinyl carbamate and its role as an intermediate in the formation of 1,N6-ethenoadenosine. Chem. Res. Toxicol..

[392] Forkert P.G., Lee R.P., Reid K. (2001). Involvement of CYP2E1 and carboxylesterase enzymes in vinyl carbamate metabolism in human lung microsomes. Drug Metab. Dispos..

[393] Sozio F., Schioppa T., Sozzani S., Del Prete A. (2021). Urethane-induced lung carcinogenesis. Methods Cell Biol..

[394] Zimmerli B., Schlatter J. (1991). Ethyl carbamate: Analytical methodology, occurrence, formation, biological activity and risk assessment. Mutat. Res..

[395] Schlatter J., Lutz W.K. (1990). The carcinogenic potential of ethyl carbamate (urethane): Risk assessment at human dietary exposure levels. Food Chem. Toxicol..

[396] Jägerstad M., Skog K. (2005). Genotoxicity of heat-processed foods. Mutat. Res./Fundam. Mol. Mech. Mutagen..

[397] Mottram D.S., Wedzicha B.L., Dodson A.T. (2002). Acrylamide is formed in the Maillard reaction. Nature.

[398] Stadler R.H., Blank I., Varga N., Robert F., Hau J., Guy P.A., Robert M.-C., Riediker S. (2002). Acrylamide from Maillard reaction products. Nature.

[399] Friedman M. (2003). Chemistry, biochemistry, and safety of acrylamide. A review. J. Agric. Food Chem..

[400] Tritscher A. (2004). Human health risk assessment of processing-related compounds in food. Toxicol. Lett..

[401] Dybing E., Farmer P.B., Andersen M., Fennell T.R., Lalljie S.P.D., Müller D.J.G., Olin S., Petersen B.J., Schlatter J., Scholz G. (2005). Human exposure and internal dose assessments of acrylamide in food. Food Chem. Toxicol..

[402] IARC, International Agency for Research on Cancer (1994). Some Industrial Chemicals. IARC Monographs on the Evaluation of Carcinogenic Risks to Humans.

[403] JECFA, Joint FAO/WHO Expert Committee on Food Additives (2011). Safety Evaluation of Certain Contaminants in Food.

[404] Commission Regulation (EU) (2017). Commission Regulation (EU) 2017/2158 of 20 November 2017 Establishing Mitigation Measures and Benchmark Levels for the Reduction of the Presence of Acrylamide in Food.

[405] Xu F., Oruna-Concha M.J., Elmore J.S. (2016). The use of asparaginase to reduce acrylamide levels in cooked food. Food Chem..

[406] Rice J.M. (2005). The carcinogenicity of acrylamide. Mutat. Res./Genet. Toxicol. Environ. Mutagen..

[407] Klaunig J.E. (2008). Acrylamide carcinogenicity. J. Agric. Food Chem..

[408] NTP, National Toxicology Program (2011). Toxicology and Carcinogenesis Studies of Acrylamide (CAS No. 79-06-1) in F344/N Rats and B6C3F1 Mice (Feed and Drinking Water Studies).

[409] Besaratinia A., Pfeifer G.P. (2007). A review of mechanisms of acrylamide carcinogenicity. Carcinogenesis.

[410] Watzek N., Böhm N., Feld J., Scherbl D., Berger F., Merz K.H., Lampen A., Reemtsma T., Tannenbaum S.R., Skipper P.L. (2012). N7-glycidamide-guanine DNA adduct formation by orally ingested acrylamide in rats: A dose-response study encompassing human diet-related exposure levels. Chem. Res. Toxicol..

[411] Klaunig J.E., Kamendulis L.M. (2005). Mechanisms of acrylamide induced rodent carcinogenesis. Adv. Exp. Med. Biol..

[412] Hagio S., Tsuji N., Furukawa S., Takeuchi K., Hayashi S., Kuroda Y., Honma M., Masumura K. (2021). Effect of sampling time on somatic and germ cell mutations induced by acrylamide in gpt delta mice. Genes Environ..

[413] Shimamura Y., Iio M., Urahira T., Masuda S. (2017). Inhibitory effects of Japanese horseradish (Wasabia japonica) on the formation and genotoxicity of a potent carcinogen, acrylamide. J. Sci. Food Agric..

[414] Katen A.L., Stanger S.J., Anderson A.L., Nixon B., Roman S.D. (2016). Chronic acrylamide exposure in male mice induces DNA damage to spermatozoa; Potential for amelioration by resveratrol. Reprod. Toxicol..

[415] Dobrovolsky V.N., Pacheco-Martinez M.M., McDaniel L.P., Pearce M.G., Ding W. (2016). In vivo genotoxicity assessment of acrylamide and glycidyl methacrylate. Food Chem. Toxicol..

[416] Calleman C.J. (1996). The metabolism and pharmacokinetics of acrylamide: Implications for mechanisms of toxicity and human risk estimation. Drug Metab. Rev..

[417] Exon J.H. (2006). A review of the toxicology of acrylamide. J. Toxicol. Environ. Health Part B.

[418] Gamboa da Costa G., Churchwell M.I., Hamilton L.P., Von Tungeln L.S., Beland F.A., Marques M.M., Doerge D.R. (2003). DNA adduct formation from acrylamide via conversion To glycidamide in adult and neonatal mice. Chem. Res. Toxicol..

[419] Dearfield K.L., Douglas G.R., Ehling U.H., Moore M.M., Sega G.A., Brusick D.J. (1995). Acrylamide: A review of its genotoxicity and an assessment of heritable genetic risk. Mutat. Res./Fundam. Mol. Mech. Mutagen..

[420] Besaratinia A., Pfeifer G.P. (2004). Genotoxicity of acrylamide and glycidamide. J. Natl. Cancer Inst..

[421] Jones D.J.L., Singh R., Emms V., Farmer P.B., Grant D., Quinn P., Maxwell C., Mina A., Ng L.L., Schumacher S. (2022). Determination of N7-glycidamide guanine adducts in human blood DNA following exposure to dietary acrylamide using liquid chromatography/tandem mass spectrometry. Rapid Commun. Mass Spectrom..

[422] Fennell T.R., Friedman M.A. (2005). Comparison of acrylamide metabolism in humans and rodents. Adv. Exp. Med. Biol..

[423] Nowak A., Zakłos-Szyda M., Żyżelewicz D., Koszucka A., Motyl I. (2020). Acrylamide decreases cell viability, and provides oxidative stress, DNA damage, and apoptosis in human colon adenocarcinoma cell line Caco-2. Molecules.

[424] De Conti A., Tryndyak V., VonTungeln L.S., Churchwell M.I., Beland F.A., Antunes A.M.M., Pogribny I.P. (2019). Genotoxic and epigenotoxic alterations in the lung and liver of mice induced by acrylamide: A 28 day drinking water study. Chem. Res. Toxicol..

[425] (2015). EFSA CONTAM Panel, European Food Safety Authority, Panel on Contaminants in the Food Chain. Scientific Opinion on acrylamide in food. EFSA J..

[426] Mucci L.A., Wilson K.M. (2008). Acrylamide intake through diet and human cancer risk. J. Agric. Food Chem..

[427] Pelucchi C., Bosetti C., Galeone C., La Vecchia C. (2015). Dietary acrylamide and cancer risk: An updated meta-analysis. Int. J. Cancer.

[428] Duale N., Bjellaas T., Alexander J., Becher G., Haugen M., Paulsen J.E., Frandsen H., Olesen P.T., Brunborg G. (2009). Biomarkers of human exposure to acrylamide and relation to polymorphisms in metabolizing genes. Toxicol. Sci..

[429] Hogervorst J.G., Schouten L.J., Konings E.J., Goldbohm R.A., van den Brandt P.A. (2007). A prospective study of dietary acrylamide intake and the risk of endometrial, ovarian, and breast cancer. Cancer Epidemiol. Biomark. Prev..

[430] Bonneck S. (2008). Acrylamide Risk Governance in Germany. Global Risk Governance.

[431] Baum M., Fauth E., Fritzen S., Herrmann A., Mertes P., Merz K., Rudolphi M., Zankl H., Eisenbrand G. (2005). Acrylamide and glycidamide: Genotoxic effects in V79-cells and human blood. Mutat. Res..

[432] Sugimura T., Wakabayashi K., Nakagama H., Nagao M. (2004). Heterocyclic amines: Mutagens/carcinogens produced during cooking of meat and fish. Cancer Sci..

[433] Nagao M., Ushijima T., Watanabe N., Okochi E., Ochiai M., Nakagama H., Sugimura T. (2002). Studies on mammary carcinogenesis induced by a heterocyclic amine, 2-amino-1-methyl-6-phenylimidazo[4,5-b]pyridine, in mice and rats. Environ. Mol. Mutagen..

[434] Weisburger J.H., Rivenson A., Kingston D.G., Wilkins T.D., Van Tassell R.L., Nagao M., Sugimura T., Hara Y. (1995). Dietary modulation of the carcinogenicity of the heterocyclic amines. Princess Takamatsu Symp..

[435] Sasaki Y.F., Saga A., Akasaka M., Nishidate E., Watanabe-Akanuma M., Ohta T., Matsusaka N., Tsuda S. (1997). In vivo genotoxicity of heterocyclic amines detected by a modified alkaline single cell gel electrophoresis assay in a multiple organ study in the mouse. Mutat. Res..

[436] Bellamri M., Walmsley S.J., Turesky R.J. (2021). Metabolism and biomarkers of heterocyclic aromatic amines in humans. Genes Environ..

[437] Fuccelli R., Rosignoli P., Servili M., Veneziani G., Taticchi A., Fabiani R. (2018). Genotoxicity of heterocyclic amines (HCAs) on freshly isolated human peripheral blood mononuclear cells (PBMC) and prevention by phenolic extracts derived from olive, olive oil and olive leaves. Food Chem. Toxicol..

[438] Bellamri M., Le Hegarat L., Vernhet L., Baffet G., Turesky R.J., Langouët S. (2016). Human T lymphocytes bioactivate heterocyclic aromatic amines by forming DNA adducts. Environ. Mol. Mutagen..

[439] Dingley K.H., Curtis K.D., Nowell S., Felton J.S., Lang N.P., Turteltaub K.W. (1999). DNA and protein adduct formation in the colon and blood of humans after exposure to a dietary-relevant dose of 2-amino-1-methyl-6-phenylimidazo[4,5-b]pyridine. Cancer Epidemiol. Biomark. Prev..

[440] Reistad R., Rossland O.J., Latva-Kala K.J., Rasmussen T., Vikse R., Becher G., Alexander J. (1997). Heterocyclic aromatic amines in human urine following a fried meat meal. Food Chem. Toxicol..

[441] Friesen M.D., Kaderlik K., Lin D., Garren L., Bartsch H., Lang N.P., Kadlubar F.F. (1994). Analysis of DNA adducts of 2-amino-1-methyl-6-phenylimidazo[4,5- b]pyridine in rat and human tissues by alkaline hydrolysis and gas chromatography/electron capture mass spectrometry: Validation by comparison with 32P-postlabeling. Chem. Res. Toxicol..

[442] Totsuka Y., Fukutome K., Takahashi M., Takahashi S., Tada A., Sugimura T., Wakabayashi K. (1996). Presence of N2-(deoxyguanosin-8-yl)-2-amino-3,8-dimethylimidazo[4,5-f]quinoxaline (dG-C8-MeIQx) in human tissues. Carcinogenesis.

[443] Pathak K.V., Chiu T.L., Amin E.A., Turesky R.J. (2016). Methemoglobin formation and characterization of hemoglobin adducts of carcinogenic aromatic amines and heterocyclic aromatic amines. Chem. Res. Toxicol..

[444] Kim D., Guengerich F.P. (2005). Cytochrome P450 activation of arylamines and heterocyclic amines. Annu. Rev. Pharm. Toxicol..

[445] Chen J., Stampfer M.J., Hough H.L., Garcia-Closas M., Willett W.C., Hennekens C.H., Kelsey K.T., Hunter D.J. (1998). A prospective study of N-acetyltransferase genotype, red meat intake, and risk of colorectal cancer. Cancer Res..

[446] Gooderham N.J., Murray S., Lynch A.M., Yadollahi-Farsani M., Zhao K., Boobis A.R., Davies D.S. (2001). Food-derived heterocyclic amine mutagens: Variable metabolism and significance to humans. Drug Metab. Dispos..

[447] McQueen C.A., Maslansky C.J., Williams G.M. (1983). Role of the acetylation polymorphism in determining susceptibility of cultured rabbit hepatocytes to DNA damage by aromatic amines. Cancer Res..

[448] Layton D.W., Bogen K.T., Knize M.G., Hatch F.T., Johnson V.M., Felton J.S. (1995). Cancer risk of heterocyclic amines in cooked foods: An analysis and implications for research. Carcinogenesis.

[449] Augustsson K., Skog K., Jägerstad M., Dickman P.W., Steineck G. (1999). Dietary heterocyclic amines and cancer of the colon, rectum, bladder, and kidney: A population-based study. Lancet.

[450] Bogen K.T., Keating G.A. (2001). U.S. dietary exposures to heterocyclic amines. J. Expo. Anal. Environ. Epidemiol..

[451] Snyderwine E.G. (1994). Some perspectives on the nutritional aspects of breast cancer research. Food-derived heterocyclic amines as etiologic agents in human mammary cancer. Cancer.

[452] Ward M.H., Sinha R., Heineman E.F., Rothman N., Markin R., Weisenburger D.D., Correa P., Zahm S.H. (1997). Risk of adenocarcinoma of the stomach and esophagus with meat cooking method and doneness preference. Int. J. Cancer.

[453] Sinha R., Kulldorff M., Swanson C.A., Curtin J., Brownson R.C., Alavanja M.C. (2000). Dietary heterocyclic amines and the risk of lung cancer among Missouri women. Cancer Res..

[454] Zhu J., Chang P., Bondy M.L., Sahin A.A., Singletary S.E., Takahashi S., Shirai T., Li D. (2003). Detection of 2-amino-1-methyl-6-phenylimidazo[4,5-b]-pyridine-DNA adducts in normal breast tissues and risk of breast cancer. Cancer Epidemiol. Biomark. Prev..

[455] Tang D., Kryvenko O.N., Wang Y., Trudeau S., Rundle A., Takahashi S., Shirai T., Rybicki B.A. (2013). 2-Amino-1-methyl-6-phenylimidazo[4,5-b]pyridine (PhIP)-DNA adducts in benign prostate and subsequent risk for prostate cancer. Int. J. Cancer.

[456] Jakszyn P., Agudo A., Ibáñez R., García-Closas R., Pera G., Amiano P., González C.A. (2004). Development of a food database of nitrosamines, heterocyclic amines, and polycyclic aromatic hydrocarbons. J. Nutr..

[457] Zelinkova Z., Wenzl T. (2015). The occurrence of 16 EPA PAHs in food—A review. Polycycl. Aromat. Compd..

[458] Lijinsky W. (1991). The formation and occurrence of polynuclear aromatic hydrocarbons associated with food. Mutat. Res..

[459] Park K.C., Pyo H., Kim W., Yoon K.S. (2017). Effects of cooking methods and tea marinades on the formation of benzo[a]pyrene in grilled pork belly (Samgyeopsal). Meat Sci..

[460] Kazerouni N., Sinha R., Hsu C.H., Greenberg A., Rothman N. (2001). Analysis of 200 food items for benzo[a]pyrene and estimation of its intake in an epidemiologic study. Food Chem. Toxicol..

[461] IARC, International Agency for Research on Cancer (2010). Some non-heterocyclic polycyclic aromatic hydrocarbons and some related exposures. IARC Monographs on the Evaluation of Carcinogenic Risks to Humans.

[462] Kroese E.D., Muller J.J.A., Mohn G.R., Dortant P.M., Wester P.W. (2001). Tumorigenic Effects in Wistar Rats Orally Administered benzo[a]pyrene for Two Years (Gavage Studies): Implications for Human Cancer Risks Associated with Oral Exposure to Polycyclic Aromatic Hydrocarbons.

[463] IARC, International Agency for Research on Cancer (2013). Bitumens and Bitumen Emissions, and some N- and S-Heterocyclic Polycyclic Aromatic Hydrocarbons. IARC Monographs on the Evaluation of Carcinogenic Risks to Humans.

[464] Moorthy B., Chu C., Carlin D.J. (2015). Polycyclic aromatic hydrocarbons: From metabolism to lung cancer. Toxicol. Sci..

[465] Gelboin H.V. (1980). Benzo[alpha]pyrene metabolism, activation and carcinogenesis: Role and regulation of mixed-function oxidases and related enzymes. Physiol. Rev..

[466] Jerina D.M., Sayer J.M., Agarwal S.K., Yagi H., Levin W., Wood A.W., Conney A.H., Pruess-Schwartz D., Baird W.M., Pigott M.A. (1986). Reactivity and tumorigenicity of bay-region diol epoxides derived from polycyclic aromatic hydrocarbons. Adv. Exp. Med. Biol..

[467] Kim K.B., Lee B.M. (1997). Oxidative stress to DNA, protein, and antioxidant enzymes (superoxide dismutase and catalase) in rats treated with benzo(a)pyrene. Cancer Lett..

[468] Kawabata T.T., White K.L. (1987). Suppression of the vitro humoral immune response of mouse splenocytes by benzo(a)pyrene metabolites and inhibition of benzo(a)pyrene-induced immunosuppression by alpha-naphthoflavone. Cancer Res..

[469] Myers J.N., Harris K.L., Rekhadevi P.V., Pratap S., Ramesh A. (2021). Benzo(a)pyrene-induced cytotoxicity, cell proliferation, DNA damage, and altered gene expression profiles in HT-29 human colon cancer cells. Cell Biol. Toxicol..

[470] Damiani L.A., Yingling C.M., Leng S., Romo P.E., Nakamura J., Belinsky S.A. (2008). Carcinogen-induced gene promoter hypermethylation is mediated by DNMT1 and causal for transformation of immortalized bronchial epithelial cells. Cancer Res..

[471] Commission Regulation (EU) (2011). Commission Regulation (EU) No 835/2011 of 19 August 2011 Amending Regulation (EC) No 1881/2006 as Regards Maximum Levels for Polycyclic Aromatic Hydrocarbons in Foodstuffs.

[472] Commission Regulation (EU) (2020). Commission Regulation (EU) No 2020/1255 of 7 September 2020 Amending Regulation (EC) No 1881/2006 as Regards Maximum Levels of Polycyclic Aromatic Hydrocarbons (PAHs) in Traditionally Smoked Meat and Smoked Meat Products and Traditionally Smoked Fish and Smoked Fishery Products and Establishing a Maximum Level of PAHs in Powders of Food of Plant Origin Used for the Preparation of Beverages.

[473] Sinha R., Kulldorff M., Gunter M.J., Strickland P., Rothman N. (2005). Dietary benzo[a]pyrene intake and risk of colorectal adenoma. Cancer Epidemiol. Biomark. Prev..

[474] Yoon E., Park K., Lee H., Yang J.-H., Lee C. (2007). Estimation of excess cancer risk on time-weighted lifetime average daily intake of PAHs from food ingestion. Hum. Ecol. Risk Assess. Int. J..

[475] Bononi M., Quaglia G., Tateo F. (2014). Identification of ethylene oxide in herbs, spices and other dried vegetables imported into Italy. Food Addit. Contam. Part A Chem. Anal. Control Expo. Risk Assess..

[476] Heuser S.G., Scudamore K.A. (1968). Fumigant residues in wheat and flour: Solvent extraction and gas-chromatographic determination of free methyl bromide and ethylene oxide. Analyst.

[477] Jensen K.G. (1988). Determination of ethylene oxide residues in processed food products by gas-liquid chromatography after derivatization. Z. Lebensm. Unters..

[478] Tthe European Parliament and the Council of the European Union (2009). Regulation (EC) No 1107/2009 of the European Parliament and of the Council of 21 October 2009 Concerning the Placing of Plant Protection Products on the Market and Repealing Council Directives 79/117/EEC and 91/414/EEC.

[479] The European Parliament (2005). Regulation (EC) No 396/2005 of the European Parliament and of the Council of 23 February 2005 on Maximum Residue Levels of Pesticides in or on Food and Feed of Plant and Animal Origin and Amending Council Directive 91/414/EEC.

[480] Kowalska A., Manning L. (2022). Food Safety Governance and Guardianship: The role of the private sector in addressing the EU ehylene oxide incident. Foods.

[481] Bessaire T., Stroheker T., Eriksen B., Mujahid C., Hammel Y.A., Varela J., Delatour T., Panchaud A., Mottier P., Stadler R.H. (2021). Analysis of ethylene oxide in ice creams manufactured with contaminated carob bean gum (E410). Food Addit. Contam. Part A Chem. Anal. Control Expo. Risk Assess..

[482] Kirman C.R., Li A.A., Sheehan P.J., Bus J.S., Lewis R.C., Hays S.M. (2021). Ethylene oxide review: Characterization of total exposure via endogenous and exogenous pathways and their implications to risk assessment and risk management. J. Toxicol. Environ. Health B Crit. Rev..

[483] Bolt H.M. (1996). Quantification of endogenous carcinogens. The ethylene oxide paradox. Biochem. Pharm..

[484] Törnqvist M., Gustafsson B., Kautiainen A., Harms-Ringdahl M., Granath F., Ehrenberg L. (1989). Unsaturated lipids and intestinal bacteria as sources of endogenous production of ethene and ethylene oxide. Carcinogenesis.

[485] Kirman C.R., Hays S.M. (2017). Derivation of endogenous equivalent values to support risk assessment and risk management decisions for an endogenous carcinogen: Ethylene oxide. Regul. Toxicol. Pharm..

[486] NTP, National Toxicology Program (1987). Toxicology and carcinogenesis studies of ethylene oxide (CAS No. 75-21-8) in B6C3F1 mice (inhalation studies). Natl. Toxicol. Program. Tech. Rep. Ser..

[487] Bolt H.M. (2000). Carcinogenicity and genotoxicity of ethylene oxide: New aspects and recent advances. Crit. Rev. Toxicol..

[488] ATSDR, Agency for Toxic Substances and Disease Registry (2020). Toxicological Profile for Ethylene Oxide.

[489] Lynch H., Kozal J.S., Russell A.J., Thompson W.J., Divis H.R., Freid R.D., Calabrese E.J., Mundt K.A. (2022). Systematic review of the scientific evidence on ethylene oxide as a human carcinogen. Chem. Biol. Interact..

[490] Dunkelberg H. (1982). Carcinogenicity of ethylene oxide and 1,2-propylene oxide upon intragastric administration to rats. Br. J. Cancer.

[491] Natarajan A.T., Preston R.J., Dellarco V., Ehrenberg L., Generoso W., Lewis S., Tates A.D. (1995). Ethylene oxide: Evaluation of genotoxicity data and an exploratory assessment of genetic risk. Mutat. Res..

[492] Farooqi Z., Törnqvist M., Ehrenberg L., Natarajan A.T. (1993). Genotoxic effects of ethylene oxide and propylene oxide in mouse bone marrow cells. Mutat. Res..

[493] Ghosh M., Godderis L. (2016). Genotoxicity of ethylene oxide: A review of micronucleus assay results in human population. Mutat. Res. Rev. Mutat. Res..

[494] Kolman A., Chovanec M., Osterman-Golkar S. (2002). Genotoxic effects of ethylene oxide, propylene oxide and epichlorohydrin in humans: Update review (1990–2001). Mutat. Res..

[495] Recio L., Donner M., Abernethy D., Pluta L., Steen A.M., Wong B.A., James A., Preston R.J. (2004). In vivo mutagenicity and mutation spectrum in the bone marrow and testes of B6C3F1 lacI transgenic mice following inhalation exposure to ethylene oxide. Mutagenesis.

[496] Bolt H.M., Peter H., Föst U. (1988). Analysis of macromolecular ethylene oxide adducts. Int. Arch. Occup. Environ. Health.

[497] Rusyn I., Asakura S., Li Y., Kosyk O., Koc H., Nakamura J., Upton P.B., Swenberg J.A. (2005). Effects of ethylene oxide and ethylene inhalation on DNA adducts, apurinic/apyrimidinic sites and expression of base excision DNA repair genes in rat brain, spleen, and liver. DNA Repair..

[498] Marsden D.A., Jones D.J., Lamb J.H., Tompkins E.M., Farmer P.B., Brown K. (2007). Determination of endogenous and exogenously derived N7-(2-hydroxyethyl)guanine adducts in ethylene oxide-treated rats. Chem. Res. Toxicol..

[499] Walker V.E., Fennell T.R., Upton P.B., Skopek T.R., Prevost V., Shuker D.E., Swenberg J.A. (1992). Molecular dosimetry of ethylene oxide: Formation and persistence of 7-(2-hydroxyethyl)guanine in DNA following repeated exposures of rats and mice. Cancer Res..

[500] Fennell T.R., Brown C.D. (2001). A physiologically based pharmacokinetic model for ethylene oxide in mouse, rat, and human. Toxicol. Appl. Pharm..

[501] Müller M., Krämer A., Angerer J., Hallier E. (1998). Ethylene oxide-protein adduct formation in humans: Influence of glutathione-S-transferase polymorphisms. Int. Arch. Occup. Environ. Health.

[502] Yong L.C., Schulte P.A., Wiencke J.K., Boeniger M.F., Connally L.B., Walker J.T., Whelan E.A., Ward E.M. (2001). Hemoglobin adducts and sister chromatid exchanges in hospital workers exposed to ethylene oxide: Effects of glutathione S-transferase T1 and M1 genotypes. Cancer Epidemiol. Biomark. Prev..

[503] Fennell T.R., MacNeela J.P., Morris R.W., Watson M., Thompson C.L., Bell D.A. (2000). Hemoglobin adducts from acrylonitrile and ethylene oxide in cigarette smokers: Effects of glutathione S-transferase T1-null and M1-null genotypes. Cancer Epidemiol. Biomark. Prev..

[504] Haufroid V., Merz B., Hofmann A., Tschopp A., Lison D., Hotz P. (2007). Exposure to ethylene oxide in hospitals: Biological monitoring and influence of glutathione S-transferase and epoxide hydrolase polymorphisms. Cancer Epidemiol. Biomark. Prev..

[505] Philippin G., Cadet J., Gasparutto D., Mazon G., Fuchs R.P. (2014). Ethylene oxide and propylene oxide derived N7-alkylguanine adducts are bypassed accurately in vivo. DNA Repairr..

[506] Tompkins E.M., McLuckie K.I., Jones D.J., Farmer P.B., Brown K. (2009). Mutagenicity of DNA adducts derived from ethylene oxide exposure in the pSP189 shuttle vector replicated in human Ad293 cells. Mutat. Res..

[507] Pottenger L.H., Boysen G., Brown K., Cadet J., Fuchs R.P., Johnson G.E., Swenberg J.A. (2019). Understanding the importance of low-molecular weight (ethylene oxide- and propylene oxide-induced) DNA adducts and mutations in risk assessment: Insights from 15 years of research and collaborative discussions. Environ. Mol. Mutagen..

[508] Gollapudi B.B., Su S., Li A.A., Johnson G.E., Reiss R., Albertini R.J. (2020). Genotoxicity as a toxicologically relevant endpoint to inform risk assessment: A case study with ethylene oxide. Environ. Mol. Mutagen..

[509] van Sittert N.J., Boogaard P.J., Natarajan A.T., Tates A.D., Ehrenberg L.G., Törnqvist M.A. (2000). Formation of DNA adducts and induction of mutagenic effects in rats following 4 weeks inhalation exposure to ethylene oxide as a basis for cancer risk assessment. Mutat. Res..

[510] SCF, Scientific Committee on Food (2002). Opinion of the Scientific Committee on Food on Impurities of Ethylene Oxide in Food Additives.

[511] Fowles J., Mitchell J., McGrath H. (2001). Assessment of cancer risk from ethylene oxide residues in spices imported into New Zealand. Food Chem. Toxicol..

[512] Bisanti L., Maggini M., Raschetti R., Alegiani S.S., Ippolito F.M., Caffari B., Segnan N., Ponti A. (1993). Cancer mortality in ethylene oxide workers. Br. J. Ind. Med..

[513] Steenland K., Whelan E., Deddens J., Stayner L., Ward E. (2003). Ethylene oxide and breast cancer incidence in a cohort study of 7576 women (United States). Cancer Causes Control.

[514] Jinot J., Fritz J.M., Vulimiri S.V., Keshava N. (2018). Carcinogenicity of ethylene oxide: Key findings and scientific issues. Toxicol. Mech. Methods.

[515] Marsh G.M., Keeton K.A., Riordan A.S., Best E.A., Benson S.M. (2019). Ethylene oxide and risk of lympho-hematopoietic cancer and breast cancer: A systematic literature review and meta-analysis. Int. Arch. Occup. Environ. Health.

[516] Vincent M.J., Kozal J.S., Thompson W.J., Maier A., Dotson G.S., Best E.A., Mundt K.A. (2019). Ethylene oxide: Cancer evidence integration and dose-response implications. Dose Response.

[517] EPA, US Environmental Protection Agency (2016). Evaluation of the Inhalation Carcinogenicity of Ethylene Oxide (CASRN 75-21-8). Support of Summary Information on the Integrated Risk Information System (IRIS); EPA/635/R-16/350Fa.

[518] German Federal Institute for Risk Assessment, Bundesinstitut für Risikobewertung (BfR) (2021). Updated BfR Opinion on Health Risk Assessment of Ethylene Oxide Residues in Sesame Seeds. Opinion no 024/2021 Issued 1 September 2021.

[519] Lijinsky W. (1999). N-Nitroso compounds in the diet. Mutat. Res..

[520] Loeppky R.N., Yu H. (2004). Amidine nitrosation. J. Org. Chem..

[521] EMA, European Medicines Agency (2020). Assessment Report. Procedure under Article 5(3) of Regulation EC (No) 726/2004. Nitrosamine Impurities in Human Medicinal Products. Procedure Number: EMEA/H/A-5(3)/1490.

[522] Leaf C.D., Wishnok J.S., Tannenbaum S.R. (1989). Mechanisms of endogenous nitrosation. Cancer Surv..

[523] Bartsch H. (1991). N-nitroso compounds and human cancer: Where do we stand?. IARC Sci. Publ..

[524] Bartsch H., Ohshima H., Pignatelli B., Calmels S. (1992). Endogenously formed N-nitroso compounds and nitrosating agents in human cancer etiology. Pharmacogenetics.

[525] Griesenbeck J.S., Steck M.D., Huber J.C., Sharkey J.R., Rene A.A., Brender J.D. (2009). Development of estimates of dietary nitrates, nitrites, and nitrosamines for use with the Short Willet Food Frequency Questionnaire. Nutr. J..

[526] Haorah J., Zhou L., Wang X., Xu G., Mirvish S.S. (2001). Determination of total N-nitroso compounds and their precursors in frankfurters, fresh meat, dried salted fish, sauces, tobacco, and tobacco smoke particulates. J. Agric. Food Chem..

[527] Li L., Wang P., Xu X., Zhou G. (2012). Influence of various cooking methods on the concentrations of volatile N-nitrosamines and biogenic amines in dry-cured sausages. J. Food Sci..

[528] Havery D.C., Kline D.A., Miletta E.M., Joe F.L., Fazio T. (1976). Survey of food products for volatile N-nitrosamines. J. Assoc. Off. Anal. Chem..

[529] IARC, International Agency for Research on Cancer (2000). Some industrial chemicals. IARC Monographs on the Evaluation of Carcinogenic Risks to Humans.

[530] IARC, International Agency for Research on Cancer (2010). Ingested nitrate and nitrite, and cyanobacterial peptide toxins. IARC Monographs on the Evaluation of Carcinogenic Risks to Humans.

[531] Jeffrey A.M., Iatropoulos M.J., Williams G.M. (2006). Nasal cytotoxic and carcinogenic activities of systemically distributed organic chemicals. Toxicol. Pathol..

[532] Pour P., Gingell R., Langenbach R., Nagel D., Grandjean C., Lawson T., Salmasi S. (1980). Carcinogenicity of N-nitrosomethyl(2-oxopropyl)amine in Syrian hamsters. Cancer Res..

[533] Thresher A., Foster R., Ponting D.J., Stalford S.A., Tennant R.E., Thomas R. (2020). Are all nitrosamines concerning? A review of mutagenicity and carcinogenicity data. Regul. Toxicol. Pharm..

[534] Lijinsky W. (1986). Mutagenesis, carcinogenesis and alkylating properties of nitrosamines and related compounds. Prog. Clin. Biol. Res..

[535] Tsuda S., Matsusaka N., Madarame H., Miyamae Y., Ishida K., Satoh M., Sekihashi K., Sasaki Y.F. (2000). The alkaline single cell electrophoresis assay with eight mouse organs: Results with 22 mono-functional alkylating agents (including 9 dialkyl N-nitrosoamines) and 10 DNA crosslinkers. Mutat. Res..

[536] Verna L. (1996). N-Nitrosodiethylamine mechanistic data and risk assessment: Bioactivation, DNA-adduct formation, mutagenicity, and tumor initiation. Pharmacol. Ther..

[537] Arimoto-Kobayashi S., Kaji K., Sweetman G.M., Hayatsu H. (1997). Mutation and formation of methyl- and hydroxylguanine adducts in DNA caused by N-nitrosodimethylamine and N-nitrosodiethylamine with UVA irradiation. Carcinogenesis.

[538] Otteneder M., Lutz W.K. (1999). Correlation of DNA adduct levels with tumor incidence: Carcinogenic potency of DNA adducts. Mutat. Res..

[539] Loeppky R.N., Ye Q., Goelzer P., Chen Y. (2002). DNA adducts from N-nitrosodiethanolamine and related beta-oxidized nitrosamines in vivo: (32)P-postlabeling methods for glyoxal- and O(6)-hydroxyethyldeoxyguanosine adducts. Chem. Res. Toxicol..

[540] Rajewsky M.F., Engelbergs J., Thomale J., Schweer T. (1998). Relevance of DNA repair to carcinogenesis and cancer therapy. Recent Results Cancer Res..

[541] Kamataki T., Fujita K.-i., Nakayama K., Yamazaki Y., Miyamoto M., Ariyoshi N. (2002). Role of human cytochrome P450 (CYP) in the metabolic activation of nitrosamine derivatives: Application of genetically engineered Salmonella expressing human CYP. Drug Metab. Rev..

[542] Yang C.S., Smith T., Ishizaki H., Hong J.Y. (1991). Enzyme mechanisms in the metabolism of nitrosamines. IARC Sci. Publ..

[543] Johnson G.E., Dobo K., Gollapudi B., Harvey J., Kenny J., Kenyon M., Lynch A., Minocherhomji S., Nicolette J., Thybaud V. (2021). Permitted daily exposure limits for noteworthy N-nitrosamines. Environ. Mol. Mutagen..

[544] Gushgari A.J., Halden R.U. (2018). Critical review of major sources of human exposure to N-nitrosamines. Chemosphere.

[545] Poirier S., Hubert A., de-Thé G., Ohshima H., Bourgade M.C., Bartsch H. (1987). Occurrence of volatile nitrosamines in food samples collected in three high-risk areas for nasopharyngeal carcinoma. IARC Sci. Publ..

[546] O’Brien P.J., Siraki A.G., Shangari N. (2005). Aldehyde sources, metabolism, molecular toxicity mechanisms, and possible effects on human health. Crit. Rev. Toxicol..

[547] Sousa B.C., Pitt A.R., Spickett C.M. (2017). Chemistry and analysis of HNE and other prominent carbonyl-containing lipid oxidation compounds. Free Radic. Biol. Med..

[548] Adams T.B., Gavin C.L., Taylor S.V., Waddell W.J., Cohen S.M., Feron V.J., Goodman J., Rietjens I.M., Marnett L.J., Portoghese P.S. (2008). The FEMA GRAS assessment of alpha,beta-unsaturated aldehydes and related substances used as flavor ingredients. Food Chem. Toxicol..

[549] JECFA, Joint FAO/WHO Expert Committee on Food Additives (2004). Evaluation of Certain Food Additives and Contaminants.

[550] Gasc N., Taché S., Rathahao E., Bertrand-Michel J., Roques V., Guéraud F. (2007). 4-Hydroxynonenal in foodstuffs: Heme concentration, fatty acid composition and freeze-drying are determining factors. Redox Rep..

[551] Feron V.J., Til H.P., de Vrijer F., Woutersen R.A., Cassee F.R., van Bladeren P.J. (1991). Aldehydes: Occurrence, carcinogenic potential, mechanism of action and risk assessment. Mutat. Res./Genet. Toxicol..

[552] IARC, International Agency for Research on Cancer (2021). Acrolein, crotonaldehyde, and arecoline. IARC Monographs on the Evaluation of Carcinogenic Risks to Humans.

[553] EFSA CONTAM Panel, European Food Safety Authority, Panel on Contaminants in the Food Chain (2018). Scientific Opinion on Flavouring Group Evaluation 200, Revision 1 (FGE.200 Rev.1): 74 a,b-unsaturated aliphatic aldehydes and precursors from chemical subgroup 1.1.1 of FGE.19. EFSA J..

[554] Chung F.L., Tanaka T., Hecht S.S. (1986). Induction of liver tumors in F344 rats by crotonaldehyde. Cancer Res..

[555] NTP, National Toxicology Program (1988). Toxicology and Carcinogenesis Studies of Malonaldehyde, Sodium Salt (3-hydroxy-2-propenal, Sodium Salt) (CAS No. 24382-04-5) in F344/N Rats and B6C3F1 Mice (Gavage Studies).

[556] NTP, National Toxicology Program (2003). Toxicology and Carcinogensis Studies of 2,4-Hexadienal (89% trans,trans Isomer, CAS No. 142-83-6; 11% cis, trans Isomer) (Gavage Studies).

[557] EFSA CONTAM Panel, European Food Safety Authority, Panel on Contaminants in the Food Chain (2018). Scientific Opinion on Flavouring Group Evaluation 203, Revision 2 (FGE.203Rev2): a,b-unsaturated aliphatic aldehydes and precursors from chemical subgroup 1.1.4 of FGE.19 with two or more conjugated double-bonds and with or without additional non-conjugated double-bonds. EFSA J..

[558] IARC, International Agency for Research on Cancer (1999). Re-evaluation of some organic chemicals, hydrazine and hydrogen Peroxide. IARC Monographs on the Evaluation of Carcinogenic Risks to Humans.

[559] Von Tungeln L.S., Yi P., Bucci T.J., Samokyszyn V.M., Chou M.W., Kadlubar F.F., Fu P.P. (2002). Tumorigenicity of chloral hydrate, trichloroacetic acid, trichloroethanol, malondialdehyde, 4-hydroxy-2-nonenal, crotonaldehyde, and acrolein in the B6C3F(1) neonatal mouse. Cancer Lett..

[560] Eder E., Deininger C., Neudecker T., Deininger D. (1992). Mutagenicity of β-alkyl substituted acrolein congeners in the Salmonella typhimurium strain TA100 and genotoxicity testing in the SOS chromotest. Environ. Mol. Mutagen..

[561] Eder E., Scheckenbach S., Deininger C., Hoffman C. (1993). The possible role of alpha, beta-unsaturated carbonyl compounds in mutagenesis and carcinogenesis. Toxicol Lett..

[562] Brambilla G., Sciabà L., Faggin P., Maura A., Marinari U.M., Ferro M., Esterbauer H. (1986). Cytotoxicity, DNA fragmentation and sister-chromatid exchange in Chinese hamster ovary cells exposed to the lipid peroxidation product 4-hydroxynonenal and homologous aldehydes. Mutat. Res..

[563] Esterbauer H. (1993). Cytotoxicity and genotoxicity of lipid-oxidation products. Am. J. Clin. Nutr..

[564] Zhang S., Villalta P.W., Wang M., Hecht S.S. (2006). Analysis of crotonaldehyde- and acetaldehyde-derived 1,n(2)-propanodeoxyguanosine adducts in DNA from human tissues using liquid chromatography electrospray ionization tandem mass spectrometry. Chem. Res. Toxicol..

[565] Guéraud F. (2017). 4-Hydroxynonenal metabolites and adducts in pre-carcinogenic conditions and cancer. Free Radic. Biol. Med..

[566] Hu W., Feng Z., Eveleigh J., Iyer G., Pan J., Amin S., Chung F.L., Tang M.S. (2002). The major lipid peroxidation product, trans-4-hydroxy-2-nonenal, preferentially forms DNA adducts at codon 249 of human p53 gene, a unique mutational hotspot in hepatocellular carcinoma. Carcinogenesis.

[567] Singh S.P., Chen T., Chen L., Mei N., McLain E., Samokyszyn V., Thaden J.J., Moore M.M., Zimniak P. (2005). Mutagenic effects of 4-hydroxynonenal triacetate, a chemically protected form of the lipid peroxidation product 4-hydroxynonenal, as assayed in L5178Y/Tk+/– mouse lymphoma cells. J. Pharmacol. Exp. Ther..

[568] Gutteridge J.M.C. (1986). Aspects to consider when detecting and measuring lipid peroxidation. Free Radic. Res. Commun..

[569] Tsikas D. (2017). Assessment of lipid peroxidation by measuring malondialdehyde (MDA) and relatives in biological samples: Analytical and biological challenges. Anal. Biochem..

[570] Nielsen N.S., Timm-Heinrich M., Jacobsen C. (2003). Comparison of wet-chemical methods for determination of lipid hydroperoxides. J. Food Lipids.

[571] Calingasan N.Y., Uchida K., Gibson G.E. (1999). Protein-bound acrolein. J. Neurochem..

[572] Yuan J.M., Gao Y.T., Wang R., Chen M., Carmella S.G., Hecht S.S. (2012). Urinary levels of volatile organic carcinogen and toxicant biomarkers in relation to lung cancer development in smokers. Carcinogenesis.

[573] Yuan J.M., Butler L.M., Gao Y.T., Murphy S.E., Carmella S.G., Wang R., Nelson H.H., Hecht S.S. (2014). Urinary metabolites of a polycyclic aromatic hydrocarbon and volatile organic compounds in relation to lung cancer development in lifelong never smokers in the Shanghai Cohort Study. Carcinogenesis.

[574] Grigoryan H., Schiffman C., Gunter M.J., Naccarati A., Polidoro S., Dagnino S., Dudoit S., Vineis P., Rappaport S.M. (2019). Cys34 adductomics links colorectal cancer with the gut microbiota and redox biology. Cancer Res..

[575] Eder E., Schuler D. (1999). Cancer risk assessment for crotonaldehyde and 2-hexenal: An approach. IARC Sci. Publ..

[576] Kunishima M., Yamauchi Y., Mizutani M., Kuse M., Takikawa H., Sugimoto Y. (2016). Identification of (Z)-3:(E)-2-hexenal isomerases essential to the production of the leaf aldehyde in plants. J. Biol Chem..

[577] Jordán M.J., Tandon K., Shaw P.E., Goodner K.L. (2001). Aromatic profile of aqueous banana essence and banana fruit by Gas Chromatography−Mass Spectrometry (GC-MS) and Gas Chromatography−Olfactometry (GC-O). J. Agric. Food Chem..

[578] EFSA CONTAM Panel, European Food Safety Authority, Panel on Contaminants in the Food Chain (2020). Scientific Opinion on Flavouring Group Evaluation 71 Revision 1 (FGE.71Rev1): Consideration of aliphatic, linear, a,b-unsaturated alcohols, aldehydes, carboxylic acids, and related esters evaluated by JECFA (63rd and 69th meeting) structurally related to flavouring substances evaluated in FGE.05Rev3. EFSA J..

[579] Nádasi E., Varjas T., Pajor L., Ember I. (2005). Carcinogenic potential of trans-2-hexenal is based on epigenetic effect. In Vivo.

[580] Dittberner U., Eisenbrand G., Zankl H. (1995). Genotoxic effects of the α, β-unsaturated aldehydes 2-trans-butenal,2-trans-hexenal and 2-trans, 6-cis-rmnonadienal. Mutat. Res./Environ. Mutagen. Relat. Subj..

[581] Dittberner U., Schmetzer B., Gölzer P., Eisenbrand G., Zankl H. (1997). Genotoxic effects of 2-trans-hexenal in human buccal mucosa cells in vivo. Mutat. Res..

[582] Eder E., Schuler D. (2000). An approach to cancer risk assessment for the food constituent 2-hexenal on the basis of 1,N2-propanodeoxyguanosine adducts of 2-hexenal in vivo. Arch. Toxicol..

[583] Schuler D., Eder E. (1999). Detection of 1,N2-propanodeoxyguanosine adducts of 2-hexenal in organs of Fischer 344 rats by a 32P-post-labeling technique. Carcinogenesis.

[584] Gölzer P., Janzowski C., Pool-Zobel B.L., Eisenbrand G. (1996). (E)-2-hexenal-induced DNA damage and formation of cyclic 1,N2-(1,3-propano)-2’-deoxyguanosine adducts in mammalian cells. Chem. Res. Toxicol..

[585] Stout M.D., Jeong Y.C., Boysen G., Li Y., Sangaiah R., Ball L.M., Gold A., Swenberg J.A. (2006). LC/MS/MS method for the quantitation of trans-2-hexenal-derived exocyclic 1,N(2)-propanodeoxyguanosine in DNA. Chem. Res. Toxicol..

[586] Stout M.D., Bodes E., Schoonhoven R., Upton P.B., Travlos G.S., Swenberg J.A. (2008). Toxicity, DNA binding, and cell proliferation in male F344 rats following short-term gavage exposures to trans-2-hexenal. Toxicol. Pathol..

[587] Kiwamoto R., Rietjens I.M., Punt A. (2012). A physiologically based in silico model for trans-2-hexenal detoxification and DNA adduct formation in rat. Chem. Res. Toxicol..

[588] Janzowski C., Glaab V., Mueller C., Straesser U., Kamp H.G., Eisenbrand G. (2003). Alpha,beta-unsaturated carbonyl compounds: Induction of oxidative DNA damage in mammalian cells. Mutagenesis.

[589] Palmer S. (1985). Diet, nutrition, and cancer. Prog. Food Nutr. Sci..

[590] Weisburger J.H. (1991). Carcinogenesis in our food and cancer prevention. Adv. Exp. Med. Biol..

[591] Yoo J.Y., Cho H.J., Moon S., Choi J., Lee S., Ahn C., Yoo K.Y., Kim I., Ko K.P., Lee J.E. (2020). Pickled vegetable and salted fish intake and the risk of gastric cancer: Two prospective cohort studies and a meta-analysis. Cancers.

[592] Gallicchio L., Matanoski G., Tao X., Chen L., Lam T.K., Boyd K., Robinson K.A., Balick L., Mickelson S., Caulfield L.E. (2006). Adulthood consumption of preserved and nonpreserved vegetables and the risk of nasopharyngeal carcinoma: A systematic review. Int. J. Cancer.

[593] IARC, International Agency for Research on Cancer (2015). Red meat and processed meat. IARC Monographs on the Evaluation of Carcinogenic Risks to Humans.

[594] Farvid M.S., Sidahmed E., Spence N.D., Mante Angua K., Rosner B.A., Barnett J.B. (2021). Consumption of red meat and processed meat and cancer incidence: A systematic review and meta-analysis of prospective studies. Eur. J. Epidemiol..

[595] Turner N.D., Lloyd S.K. (2017). Association between red meat consumption and colon cancer: A systematic review of experimental results. Exp. Biol. Med..

[596] Anderson J.J., Darwis N.D.M., Mackay D.F., Celis-Morales C.A., Lyall D.M., Sattar N., Gill J.M.R., Pell J.P. (2018). Red and processed meat consumption and breast cancer: UK Biobank cohort study and meta-analysis. Eur. J. Cancer.

[597] Turesky R.J. (2018). Mechanistic evidence for red meat and processed meat intake and cancer risk: A follow-up on the International Agency for Research on Cancer evaluation of 2015. Chimia.

[598] Tannenbaum S.R., Bishop W., Yu M.C., Henderson B.E. (1985). Attempts to isolate N-nitroso compounds from Chinese-style salted fish. Natl. Cancer Inst. Monogr..

[599] IARC, International Agency for Research on Cancer (2012). Review of human carcinogens. Personal habits and indoor combustions. IARC Monographs on the Evaluation of Carcinogenic Risks to Humans.

[600] Qiu Y., Chen J.H., Yu W., Wang P., Rong M., Deng H. (2017). Contamination of Chinese salted fish with volatile N-nitrosamines as determined by QuEChERS and gas chromatography-tandem mass spectrometry. Food Chem..

[601] Poirier S., Bouvier G., Malaveille C., Ohshima H., Shao Y.M., Hubert A., Zeng Y., de Thé G., Bartsch H. (1989). Volatile nitrosamine levels and genotoxicity of food samples from high-risk areas for nasopharyngeal carcinoma before and after nitrosation. Int. J. Cancer.

[602] Zheng X., Luo Y., Christensson B., Drettner B. (1994). Induction of nasal and nasopharyngeal tumours in Sprague-Dawley rats fed with Chinese salted fish. Acta Otolaryngol..

[603] Yu M.C., Nichols P.W., Zou X.N., Estes J., Henderson B.E. (1989). Induction of malignant nasal cavity tumours in Wistar rats fed Chinese salted fish. Br. J. Cancer.

[604] Huang D.P., Ho J.H., Saw D., Teoh T.B. (1978). Carcinoma of the nasal and paranasal regions in rats fed Cantonese salted marine fish. IARC Sci. Publ..

[605] Weisburger J.H., Marquardt H., Hirota N., Mori H., Williams G.M. (1980). Induction of cancer of the glandular stomach in rats by an extract of nitrite-treated fish. J. Natl. Cancer Inst..

[606] Widlak P., Zheng X., Osterdahl B.G., Drettner B., Christensson B., Kumar R., Hemminki K. (1995). N-nitrosodimethylamine and 7-methylguanine DNA adducts in tissues of rats fed Chinese salted fish. Cancer Lett..

[607] Yuan J.M., Wang X.L., Xiang Y.B., Gao Y.T., Ross R.K., Yu M.C. (2000). Preserved foods in relation to risk of nasopharyngeal carcinoma in Shanghai, China. Int. J. Cancer.

[608] Lian M. (2022). Salted fish and processed foods intake and nasopharyngeal carcinoma risk: A dose-response meta-analysis of observational studies. Eur. Arch. Otorhinolaryngol..

[609] Hara N., Sakata K., Nagai M., Fujita Y., Hashimoto T., Yanagawa H. (1984). Statistical analyses on the pattern of food consumption and digestive-tract cancers in Japan. Nutr. Cancer.

[610] FDA, Food and Drug Administration (2018). Food Additive Regulations. Synthetic Flavoring Agents and Adjuvants. A Rule by the Food and Drug Administration on 10/09/2018 83 FR 50490. 21 CFR 172. 21 CFR 177. Document Number: 2018-21807. Final Rule; Notif. Part Denial Petition.

[611] IARC, International Agency for Research on Cancer (2019). Some chemicals that cause tumours of the urinary tract in rodents. IARC Monographs on the Evaluation of Carcinogenic Risks to Humans.

[612] Felter S.P., Llewelyn C., Navarro L., Zhang X. (2020). How the 62-year old Delaney Clause continues to thwart science: Case study of the flavor substance β-myrcene. Regul. Toxicol. Pharm..

[613] Api A.M., Belmonte F., Belsito D., Biserta S., Botelho D., Bruze M., Burton G.A., Buschmann J., Cancellieri M.A., Dagli M.L. (2020). RIFM fragrance ingredient safety assessment, myrcene, CAS Registry Number 123-35-3. Food Chem. Toxicol..

[614] NTP, National Toxicology Program (2010). Technical report on the toxicology and carcinogenesis studies of beta-myrcene (CAS No. 123-35-3) in F344/N rats and B6C3F1 mice (gavage studies). Natl. Toxicol. Program Tech. Rep. Ser..

[615] JECFA, Joint FAO/WHO Expert Committee on Food Additives (2015). Safety Evaluation of Certain Food Additives.

[616] EFSA CEF Panel, European Food Safety Authority, Panel on Food Contact Materials, Enzymes, Flavourings and Processing Aids (2015). Scientific Opinion on Flavouring Group Evaluation 78, Revision 2 (FGE.78Rev2): Consideration of aliphatic and alicyclic and aromatic hydrocarbons evaluated by JECFA (63rd meeting) structurally related to aliphatic hydrocarbonsevaluated by EFSA in FGE.25Rev3. EFSA J..

[617] Kauderer B., Zamith H., Paumgartten F.J., Speit G. (1991). Evaluation of the mutagenicity of beta-myrcene in mammalian cells in vitro. Environ. Mol. Mutagen..

[618] Zamith H.P., Vidal M.N., Speit G., Paumgartten F.J. (1993). Absence of genotoxic activity of beta-myrcene in the in vivo cytogenetic bone marrow assay. Braz. J. Med. Biol. Res..

[619] Cohen S.M., Eisenbrand G., Fukushima S., Gooderham N.J., Guengerich F.P., Hecht S.S., Rietjens I., Bastaki M., Davidsen J.M., Harman C.L. (2019). FEMA GRAS assessment of natural flavor complexes: Citrus-derived flavoring ingredients. Food Chem. Toxicol..

[620] Madyastha K.M., Srivatsan V. (1987). Metabolism of beta-myrcene in vivo and in vitro: Its effects on rat-liver microsomal enzymes. Xenobiotica.

[621] Ishida T., Asakawa Y., Takemoto T., Aratani T. (1981). Terpenoids biotransformation in mammals III: Biotransformation of alpha-pinene, beta-pinene, pinane, 3-carene, carane, myrcene, and p-cymene in rabbits. J. Pharm. Sci..

[622] Adams T.B., Gavin C.L., McGowen M.M., Waddell W.J., Cohen S.M., Feron V.J., Marnett L.J., Munro I.C., Portoghese P.S., Rietjens I.M. (2011). The FEMA GRAS assessment of aliphatic and aromatic terpene hydrocarbons used as flavor ingredients. Food Chem. Toxicol..

[623] De-Oliveira A.C., Ribeiro-Pinto L.F., Paumgartten J.R. (1997). In vitro inhibition of CYP2B1 monooxygenase by beta-myrcene and other monoterpenoid compounds. Toxicol. Lett..

[624] De-Oliveira A.C., Ribeiro-Pinto L.F., Otto S.S., Gonçalves A., Paumgartten F.J. (1997). Induction of liver monooxygenases by beta-myrcene. Toxicology.

[625] Cesta M.F., Hard G.C., Boyce J.T., Ryan M.J., Chan P.C., Sills R.C. (2013). Complex histopathologic response in rat kidney to oral β-myrcene: An unusual dose-related nephrosis and low-dose alpha2u-globulin nephropathy. Toxicol. Pathol..

[626] JECFA, Joint FAO/WHO Expert Committee on Food Additives (2005). Evaluation of Certain Food Additives.

[627] Mog S.R., Zang Y.J. (2019). Safety assessment of food additives: Case example with myrcene, a synthetic flavoring agent. Toxicol. Pathol..

[628] IARC, International Agency for Research on Cancer (2016). Some drugs and herbal products. IARC Monographs on the Evaluation of Carcinogenic Risks to Humans.

[629] JECFA, Joint FAO/WHO Expert Committee on Food Additives (2001). Evaluation of Certain Food Additives and Contaminants.

[630] EFSA AFC Panel, European Food Safety Authority, Panel on Food Additives, Flavourings, Processing Aids and Materials in contact with Food (2005). Opinion of the Scientific Panel on Food Additives, Flavourings, Processing Aids and Materials in contact with Foods on a request from the Commission on Pulegone and Menthofuran in flavourings and other food ingredients with flavouring properties. Question number EFSA-Q-2003-119. EFSA J..

[631] EMA, European Medicines Agency (2016). Public Statement on the Use of Herbal Medicinal Products Containing Pulegone and Menthofuran.

[632] NTP, National Toxicology Program (2011). Toxicology and carcinogenesis studies of pulegone (CAS No. 89-82-7) in F344/N rats and B6C3F1 mice (gavage studies). Natl. Toxicol. Program. Tech. Rep. Ser..

[633] Api A.M., Belsito D., Biserta S., Botelho D., Bruze M., Burton G.A., Buschmann J., Cancellieri M.A., Dagli M.L., Date M. (2021). RIFM fragrance ingredient safety assessment, pulegone, CAS Registry Number 89-82-7. Food Chem. Toxicol..

[634] Khojasteh S.C., Hartley D.P., Ford K.A., Uppal H., Oishi S., Nelson S.D. (2012). Characterization of rat liver proteins adducted by reactive metabolites of menthofuran. Chem. Res. Toxicol..

[635] Chen L.J., Lebetkin E.H., Burka L.T. (2001). Metabolism of (R)-(+)-pulegone in F344 rats. Drug Metab. Dispos..

[636] Chen L.J., Lebetkin E.H., Burka L.T. (2003). Comparative disposition of (R)-(+)-pulegone in B6C3F1 mice and F344 rats. Drug Metab. Dispos..

[637] Khojasteh-Bakht S.C., Chen W., Koenigs L.L., Peter R.M., Nelson S.D. (1999). Metabolism of (R)-(+)-pulegone and (R)-(+)-menthofuran by human liver cytochrome P-450s: Evidence for formation of a furan epoxide. Drug Metab. Dispos..

[638] Gordon W.P., Huitric A.C., Seth C.L., McClanahan R.H., Nelson S.D. (1987). The metabolism of the abortifacient terpene, (R)-(+)-pulegone, to a proximate toxin, menthofuran. Drug Metab. Dispos..

[639] Nelson S.D., McClanahan R.H., Thomassen D., Gordon W.P., Knebel N. (1992). Investigations of mechanisms of reactive metabolite formation from (R)-(+)-pulegone. Xenobiotica.

[640] Engel W. (2003). In vivo studies on the metabolism of the monoterpene pulegone in humans using the metabolism of ingestion-correlated amounts (MICA) approach: Explanation for the toxicity differences between (S)-(-)- and (R)-(+)-pulegone. J. Agric. Food Chem..

[641] McClanahan R.H., Thomassen D., Slattery J.T., Nelson S.D. (1989). Metabolic activation of (R)-(+)-pulegone to a reactive enonal that covalently binds to mouse liver proteins. Chem. Res. Toxicol..

[642] Da Rocha M.S., Dodmane P.R., Arnold L.L., Pennington K.L., Anwar M.M., Adams B.R., Taylor S.V., Wermes C., Adams T.B., Cohen S.M. (2012). Mode of action of pulegone on the urinary bladder of F344 rats. Toxicol. Sci..

[643] Alshannaq A., Yu J.H. (2017). Occurrence, toxicity, and analysis of major mycotoxins in food. Int. J. Environ. Res. Public Health.

[644] Riley R.T., Merrill A.H. (2019). Ceramide synthase inhibition by fumonisins: A perfect storm of perturbed sphingolipid metabolism, signaling, and disease. J. Lipid Res..

[645] Schroeder J.J., Crane H.M., Xia J., Liotta D.C., Merrill A.H. (1994). Disruption of sphingolipid metabolism and stimulation of DNA synthesis by fumonisin B1. A molecular mechanism for carcinogenesis associated with Fusarium moniliforme. J. Biol. Chem..

[646] NTP, National Toxicology Program (2001). Toxicology and carcinogenesis studies of fumonisin B1 (cas no. 116355-83-0) in F344/N rats and B6C3F1 mice (feed studies). Natl. Toxicol. Program. Tech. Rep. Ser..

[647] JECFA, Joint FAO/WHO Expert Committee on Food Additives (2001). Safety Evaluation of Certain Mycotoxins in Food.

[648] Gelderblom W.C.A., Marasas W.F.O., Lebepe-Mazur S., Swanevelder S., Abel S. (2008). Cancer initiating properties of fumonisin B1 in a short-term rat liver carcinogenesis assay. Toxicology.

[649] Gelderblom W.C.A., Snyman S.D., Lebepe-Mazur S., van der Westhuizen L., Kriek N.P.J., Marasas W.F.O. (1996). The cancer-promoting potential of fumonisin B1 in rat liver using diethylnitrosamine as a cancer initiator. Cancer Lett..

[650] Sakai A., Suzuki C., Masui Y., Kuramashi A., Takatori K., Tanaka N. (2007). The activities of mycotoxins derived from Fusarium and related substances in a short-term transformation assay using v-Ha-ras-transfected BALB/3T3 cells (Bhas 42 cells). Mutat. Res..

[651] Carlson D.B., Williams D.E., Spitsbergen J.M., Ross P.F., Bacon C.W., Meredith F.I., Riley R.T. (2001). Fumonisin B1 promotes aflatoxin B1 and N-methyl-N’-nitro-nitrosoguanidine-initiated liver tumors in rainbow trout. Toxicol. Appl. Pharm..

[652] Mobio T.A., Tavan E., Baudrimont I., Anane R., Carratú M.R., Sanni A., Gbeassor M.F., Shier T.W., Narbonne J.F., Creppy E.E. (2003). Comparative study of the toxic effects of fumonisin B1 in rat C6 glioma cells and p53-null mouse embryo fibroblasts. Toxicology.

[653] Galvano F., Russo A., Cardile V., Galvano G., Vanella A., Renis M. (2002). DNA damage in human fibroblasts exposed to fumonisin B(1). Food Chem. Toxicol..

[654] Domijan A.M., Zeljezić D., Milić M., Peraica M. (2007). Fumonisin B(1): Oxidative status and DNA damage in rats. Toxicology.

[655] Hassan A.M., Abdel-Aziem S.H., El-Nekeety A.A., Abdel-Wahhab M.A. (2015). Panax ginseng extract modulates oxidative stress, DNA fragmentation and up-regulate gene expression in rats sub chronically treated with aflatoxin B1 and fumonisin B 1. Cytotechnology.

[656] Ehrlich V., Darroudi F., Uhl M., Steinkellner H., Zsivkovits M., Knasmueller S. (2002). Fumonisin B(1) is genotoxic in human derived hepatoma (HepG2) cells. Mutagenesis.

[657] Aranda M., Pérez-Alzola L.P., Ellahueñe M.F., Sepúlveda C. (2000). Assessment of in vitro mutagenicity in Salmonella and in vivo genotoxicity in mice of the mycotoxin fumonisin B(1). Mutagenesis.

[658] Mary V.S., Valdehita A., Navas J.M., Rubinstein H.R., Fernández-Cruz M.L. (2015). Effects of aflatoxin B₁, fumonisin B₁ and their mixture on the aryl hydrocarbon receptor and cytochrome P450 1A induction. Food Chem. Toxicol..

[659] Spotti M., Maas R.F., de Nijs C.M., Fink-Gremmels J. (2000). Effect of fumonisin B(1) on rat hepatic P450 system. Environ. Toxicol. Pharm..

[660] Müller S., Dekant W., Mally A. (2012). Fumonisin B1 and the kidney: Modes of action for renal tumor formation by fumonisin B1 in rodents. Food Chem. Toxicol..

[661] Harrer H., Humpf H.U., Voss K.A. (2015). In vivo formation of N-acyl-fumonisin B1. Mycotoxin Res..

[662] Wang E., Norred W.P., Bacon C.W., Riley R.T., Merrill A.H. (1991). Inhibition of sphingolipid biosynthesis by fumonisins. Implications for diseases associated with Fusarium moniliforme. J. Biol. Chem..

[663] Voss K.A., Howard P.C., Riley R.T., Sharma R.P., Bucci T.J., Lorentzen R.J. (2002). Carcinogenicity and mechanism of action of fumonisin B1: A mycotoxin produced by Fusarium moniliforme (F. verticillioides). Cancer Detect. Prev..

[664] Riley R.T., Enongene E., Voss K.A., Norred W.P., Meredith F.I., Sharma R.P., Spitsbergen J., Williams D.E., Carlson D.B., Merrill A.H. (2001). Sphingolipid perturbations as mechanisms for fumonisin carcinogenesis. Environ. Health Perspect..

[665] Merrill A.H., Sullards M.C., Wang E., Voss K.A., Riley R.T. (2001). Sphingolipid metabolism: Roles in signal transduction and disruption by fumonisins. Environ. Health Perspect..

[666] Gelderblom W.C., Abel S., Smuts C.M., Marnewick J., Marasas W.F., Lemmer E.R., Ramljak D. (2001). Fumonisin-induced hepatocarcinogenesis: Mechanisms related to cancer initiation and promotion. Environ. Health Perspect..

[667] Rumora L., Domijan A.M., Grubisić T.Z., Peraica M. (2007). Mycotoxin fumonisin B1 alters cellular redox balance and signalling pathways in rat liver and kidney. Toxicology.

[668] Yin J.J., Smith M.J., Eppley R.M., Page S.W., Sphon J.A. (1998). Effects of fumonisin B1 on lipid peroxidation in membranes. Biochim. Biophys. Acta.

[669] Sun G., Wang S., Hu X., Su J., Huang T., Yu J., Tang L., Gao W., Wang J.S. (2007). Fumonisin B1 contamination of home-grown corn in high-risk areas for esophageal and liver cancer in China. Food Addit. Contam..

[670] Wang H., Wei H., Ma J., Luo X. (2000). The fumonisin B1 content in corn from North China, a high-risk area of esophageal cancer. J. Environ. Pathol. Toxicol. Oncol..

[671] Isaacson C. (2005). The change of the staple diet of black South Africans from sorghum to maize (corn) is the cause of the epidemic of squamous carcinoma of the oesophagus. Med. Hypotheses.

[672] van der Westhuizen L., Shephard G.S., Scussel V.M., Costa L.L., Vismer H.F., Rheeder J.P., Marasas W.F. (2003). Fumonisin contamination and fusarium incidence in corn from Santa Catarina, Brazil. J. Agric. Food Chem..

[673] Claeys L., Romano C., De Ruyck K., Wilson H., Fervers B., Korenjak M., Zavadil J., Gunter M.J., De Saeger S., De Boevre M. (2020). Mycotoxin exposure and human cancer risk: A systematic review of epidemiological studies. Compr. Rev. Food Sci. Food Saf..

[674] Persson E.C., Sewram V., Evans A.A., London W.T., Volkwyn Y., Shen Y.J., Van Zyl J.A., Chen G., Lin W., Shephard G.S. (2012). Fumonisin B1 and risk of hepatocellular carcinoma in two Chinese cohorts. Food Chem. Toxicol..

[675] Cantalejo M.J., Torondel P., Amate L., Carrasco J.M., Hernández E. (1999). Detection of fusarin C and trichothecenes in Fusarium strains from Spain. J. Basic Microbiol..

[676] Kleigrewe K., Söhnel A.C., Humpf H.U. (2011). A new high-performance liquid chromatography-tandem mass spectrometry method based on dispersive solid phase extraction for the determination of the mycotoxin fusarin C in corn ears and processed corn samples. J. Agric. Food Chem..

[677] Han Z., Tangni E.K., Huybrechts B., Munaut F., Scauflaire J., Wu A., Callebaut A. (2014). Screening survey of co-production of fusaric acid, fusarin C, and fumonisins B₁, B₂ and B₃ by Fusarium strains grown in maize grains. Mycotoxin Res..

[678] Zhu B., Jeffrey A.M. (1992). Stability of Fusarin C: Effects of the normal cooking procedure used in china and ph. Nutr. Cancer.

[679] Gelderblom W.C., Thiel P.G., Jaskiewicz K., Marasas W.F. (1986). Investigations on the carcinogenicity of fusarin C--a mutagenic metabolite of Fusarium moniliforme. Carcinogenesis.

[680] Cheng S.J., Jiang Y.Z., Li M.H., Lo H.Z. (1985). A mutagenic metabolite produced by Fusarium moniliforme isolated from Linxian county, China. Carcinogenesis.

[681] Gelderblom W.C., Snyman S.D. (1991). Mutagenicity of potentially carcinogenic mycotoxins produced by Fusarium moniliforme. Mycotoxin Res..

[682] Norred W.P., Plattner R.D., Vesonder R.F., Bacon C.W., Voss K.A. (1992). Effects of selected secondary metabolites of Fusarium moniliforme on unscheduled synthesis of DNA by rat primary hepatocytes. Food Chem. Toxicol..

[683] Bever R.J., Couch L.H., Sutherland J.B., Williams A.J., Beger R.D., Churchwell M.I., Doerge D.R., Howard P.C. (2000). DNA adduct formation by Fusarium culture extracts: Lack of role of fusarin C. Chem. Biol. Interact..

[684] Lu S.J., Ronai Z.A., Li M.H., Jeffrey A.M. (1988). Fusarium moniliforme metabolites: Genotoxicity of culture extracts. Carcinogenesis.

[685] Gelderblom W.C., Swart P., Kramer P.S. (1988). Investigations on the spectral interactions of fusarin C with rat liver microsomal cytochrome P-450. Xenobiotica.

[686] Lu S.J., Li M.H., Park S.S., Gelboin H.V., Jeffrey A.M. (1989). Metabolism of fusarin C by rat liver microsomes. Role of esterase and cytochrome P-450 enzymes with respect to the mutagenicity of fusarin C in Salmonella typhimurium. Biochem. Pharm..

[687] Zhu B., Jeffrey A.M. (1993). Fusarin C: Isolation and identification of two microsomal metabolites. Chem. Res. Toxicol..

[688] Sondergaard T.E., Hansen F.T., Purup S., Nielsen A.K., Bonefeld-Jørgensen E.C., Giese H., Sørensen J.L. (2011). Fusarin C acts like an estrogenic agonist and stimulates breast cancer cells in vitro. Toxicol. Lett..

[689] Marasas W.F., Jaskiewicz K., Venter F.S., Van Schalkwyk D.J. (1988). Fusarium moniliforme contamination of maize in oesophageal cancer areas in Transkei. S. Afr. Med. J..

[690] IARC, International Agency for Research on Cancer (2016). Polychlorinated biphenyls and polybrominted biphenyls. IARC Monographs on the Evaluation of Carcinogenic Risks to Humans.

[691] IARC, International Agency for Research on Cancer (2018). DDT, lindane, and 2,4-D. IARC Monographs on the Evaluation of Carcinogenic Risks to Humans.

[692] Walker K. (2000). Cost-comparison of DDT and alternative insecticides for malaria control. Med. Vet. Entomol..

[693] Pedercini M., Movilla Blanco S., Kopainsky B. (2011). Application of the malaria management model to the analysis of costs and benefits of DDT versus non-DDT malaria control. PLoS ONE.

[694] ATSDR, Agency for Toxic Substances and Disease Registry (2022). Toxicological Profile for DDT, DDE, and DDD.

[695] WHO, World Health Organization (2011). The Use of DDT in Malaria Vector Control.

[696] Smith D. (1999). Worldwide trends in DDT levels in human breast milk. Int. J. Epidemiol..

[697] van den Berg M., Kypke K., Kotz A., Tritscher A., Lee S.Y., Magulova K., Fiedler H., Malisch R. (2017). WHO/UNEP global surveys of PCDDs, PCDFs, PCBs and DDTs in human milk and benefit-risk evaluation of breastfeeding. Arch. Toxicol..

[698] NCI, National Cancer Institute (1978). Bioassay of DDT, TDE, and p,p′-DDE for Possible Carcinogenicity.

[699] IARC, International Agency for Research on Cancer (1991). DDT and associated compounds. Occupational exposures in insecticide application, and some pesticides. IARC Monographs on the Evaluation of Carcinogenic Risks to Humans.

[700] EFSA CONTAM Panel, European Food Safety Authority, Panel on Contaminants in the Food Chain (2006). Opinion of the Scientific Panel on contaminants in the food chain [CONTAM] related to DDT as an undesirable substance in animal feed. EFSA J..

[701] Tebourbi O., Driss M.R., Sakly M., Rhouma K.B. (2006). Metabolism of DDT in different tissues of young rats. J. Environ. Sci. Health B.

[702] Morgan D.P., Roan C.C. (1971). Absorption, storage, and metabolic conversion of ingested DDT and DDT metabolites in man. Arch. Environ. Health.

[703] Nims R.W., Lubet R.A., Fox S.D., Jones C.R., Thomas P.E., Reddy A.B., Kocarek T.A. (1998). Comparative pharmacodynamics of CYP2B induction by DDT, DDE, and DDD in male rat liver and cultured rat hepatocytes. J. Toxicol. Environ. Health A.

[704] Lund B.O., Bergman A., Brandt I. (1988). Metabolic activation and toxicity of a DDT-metabolite, 3-methylsulphonyl-DDE, in the adrenal zona fasciculata in mice. Chem. Biol. Interact..

[705] Williams G.M., Numoto S. (1984). Promotion of mouse liver neoplasms by the organochlorine pesticides chlordane and heptachlor in comparison to dichlorodiphenyltrichlorpethane. Carcinogenesis.

[706] Williams G.M., Telang S., Tong C. (1981). Inhibition of intercellular communication between liver cells by the liver tumor promoter 1,1,1-trichloro-2,2-bis(p-chlorophenyl)ethane. Cancer Lett..

[707] López-Cervantes M., Torres-Sánchez L., Tobías A., López-Carrillo L. (2003). Dichlorodiphenyldichloroethane burden and breast cancer risk: A meta-analysis of the epidemiologic evidence. Environ. Health Perspect..

[708] Gatto N.M., Longnecker M.P., Press M.F., Sullivan-Halley J., McKean-Cowdin R., Bernstein L. (2007). Serum organochlorines and breast cancer: A case–control study among African-American women. Cancer Causes Control.

[709] Mouly T.A., Toms L.L. (2016). Breast cancer and persistent organic pollutants (excluding DDT): A systematic literature review. Environ. Sci. Pollut. Res. Int..

[710] VoPham T., Bertrand K.A., Hart J.E., Laden F., Brooks M.M., Yuan J.M., Talbott E.O., Ruddell D., Chang C.H., Weissfeld J.L. (2017). Pesticide exposure and liver cancer: A review. Cancer Causes Control.

[711] Baris D., Zahm S.H., Cantor K.P., Blair A. (1998). Agricultural use of DDT and risk of non-Hodgkin’s lymphoma: Pooled analysis of three case-control studies in the United States. Occup. Environ. Med..

[712] Kulkarni P.S., Crespo J.G., Afonso C.A. (2008). Dioxins sources and current remediation technologies—A review. Environ. Int..

[713] Institute of Medicine, (US) Committee on the Implications of Dioxin in the Food Supply (2003). Dioxins and Dioxin-like Compounds in the Food Supply: Strategies to Decrease Exposure.

[714] Van den Berg M., Birnbaum L., Bosveld A.T., Brunström B., Cook P., Feeley M., Giesy J.P., Hanberg A., Hasegawa R., Kennedy S.W. (1998). Toxic equivalency factors (TEFs) for PCBs, PCDDs, PCDFs for humans and wildlife. Environ. Health Perspect..

[715] Van den Berg M., Birnbaum L.S., Denison M., De Vito M., Farland W., Feeley M., Fiedler H., Hakansson H., Hanberg A., Haws L. (2006). The 2005 World Health Organization reevaluation of human and Mammalian toxic equivalency factors for dioxins and dioxin-like compounds. Toxicol. Sci..

[716] Schecter A., Birnbaum L., Ryan J.J., Constable J.D. (2006). Dioxins: An overview. Environ. Res..

[717] Charnley G., Kimbrough R.D. (2006). Overview of exposure, toxicity, and risks to children from current levels of 2,3,7,8-tetrachlorodibenzo-p-dioxin and related compounds in the USA. Food Chem. Toxicol..

[718] Scialli A.R., Watkins D.K., Ginevan M.E. (2015). Agent Orange eposure and 2,3,7,8-tetrachlorodibenzo-p-dioxin (TCDD) in human milk. Birth Defects Res. B Dev. Reprod. Toxicol..

[719] Ulaszewska M.M., Zuccato E., Davoli E. (2011). PCDD/Fs and dioxin-like PCBs in human milk and estimation of infants’ daily intake: A review. Chemosphere.

[720] Arisawa K. (2018). Recent decreasing trends of exposure to PCDDs/PCDFs/dioxin-like PCBs in general populations, and associations with diabetes, metabolic syndrome, and gout/hyperuricemia. J. Med. Investig..

[721] NTP, National Toxicology Program (2006). Toxicology and carcinogenesis studies of a mixture of 2,3,7,8-tetrachlorodibenzo-p-dioxin (TCDD) (Cas No. 1746-01-6), 2,3,4,7,8-pentachlorodibenzofuran (PeCDF) (Cas No. 57117-31-4), and 3,3′,4,4′,5-pentachlorobiphenyl (PCB 126) (Cas No. 57465-28-8) in female Harlan Sprague-Dawley rats (gavage studies). Natl. Toxicol. Program. Tech. Rep. Ser..

[722] NTP, National Toxicology Program (2006). Toxicology and carcinogenesis studies of 3,3′,4,4′,5-pentachlorobiphenyl (PCB 126) (CAS No. 57465-28-8) in female Harlan Sprague-Dawley rats (Gavage Studies). Natl. Toxicol. Program. Tech. Rep. Ser..

[723] (2006). NTP, National Toxicology Program. Technical report on the toxicology and carcinogenesis studies of 2,3,7,8-tetrachlorodibenzo-p-dioxin (TCDD) (CAS No. 1746-01-6) in female Harlan Sprague-Dawley rats (Gavage Studies). Natl. Toxicol. Program. Tech. Rep. Ser..

[724] Knerr S., Schrenk D. (2006). Carcinogenicity of “non-dioxinlike” polychlorinated biphenyls. Crit. Rev. Toxicol..

[725] Huff J.E., Salmon A.G., Hooper N.K., Zeise L. (1991). Long-term carcinogenesis studies on 2,3,7,8-tetrachlorodibenzo-p-dioxin and hexachlorodibenzo-p-dioxins. Cell Biol. Toxicol..

[726] Turteltaub K.W., Felton J.S., Gledhill B.L., Vogel J.S., Southon J.R., Caffee M.W., Finkel R.C., Nelson D.E., Proctor I.D., Davis J.C. (1990). Accelerator mass spectrometry in biomedical dosimetry: Relationship between low-level exposure and covalent binding of heterocyclic amine carcinogens to DNA. Proc. Natl. Acad. Sci. USA.

[727] Randerath K., Putman K.L., Randerath E., Mason G., Kelley M., Safe S. (1988). Organ-specific effects of long term feeding of 2,3,7,8-tetrachlorodibenzo-p-dioxin and 1,2,3,7,8-pentachlorodibenzo-p-dioxin on I-compounds in hepatic and renal DNA of female Sprague-Dawley rats. Carcinogenesis.

[728] Dragan Y.P., Schrenk D. (2000). Animal studies addressing the carcinogenicity of TCDD (or related compounds) with an emphasis on tumour promotion. Food Addit. Contam..

[729] Whysner J., Montandon F., McClain R.M., Downing J., Verna L.K., Steward R.E., Williams G.M. (1998). Absence of DNA adduct formation by phenobarbital, polychlorinated biphenyls, and chlordane in mouse liver using the 32P-postlabeling assay. Toxicol. Appl. Pharmacol..

[730] Schilderman P.A., Maas L.M., Pachen D.M., de Kok T.M., Kleinjans J.C., van Schooten F.J. (2000). Induction of DNA adducts by several polychlorinated biphenyls. Environ. Mol. Mutagen..

[731] Pohjanvirta R., Vartiainen T., Uusi-Rauva A., Mönkkönen J., Tuomisto J. (1990). Tissue distribution, metabolism, and excretion of 14C-TCDD in a TCDD-susceptible and a TCDD-resistant rat strain. Pharm. Toxicol..

[732] Olson J.R. (1986). Metabolism and disposition of 2,3,7,8-tetrachlorodibenzo-p-dioxin in guinea pigs. Toxicol. Appl. Pharm..

[733] Kahn P.C., Gochfeld M., Nygren M., Hansson M., Rappe C., Velez H., Ghent-Guenther T., Wilson W.P. (1988). Dioxins and dibenzofurans in blood and adipose tissue of Agent Orange-exposed Vietnam veterans and matched controls. JAMA.

[734] Diliberto J.J., DeVito M.J., Ross D.G., Birnbaum L.S. (2001). Subchronic exposure of [3H]- 2,3,7,8-tetrachlorodibenzo-p-dioxin (TCDD) in female B6C3F1 mice: Relationship of steady-state levels to disposition and metabolism. Toxicol. Sci..

[735] Gasiewicz T.A., Geiger L.E., Rucci G., Neal R.A. (1983). Distribution, excretion, and metabolism of 2,3,7,8-tetrachlorodibenzo-p-dioxin in C57BL/6J, DBA/2J, and B6D2F1/J mice. Drug Metab. Dispos..

[736] Inui H., Itoh T., Yamamoto K., Ikushiro S., Sakaki T. (2014). Mammalian cytochrome P450-dependent metabolism of polychlorinated dibenzo-p-dioxins and coplanar polychlorinated biphenyls. Int. J. Mol. Sci..

[737] Wroblewski V.J., Olson J.R. (1985). Hepatic metabolism of 2,3,7,8-tetrachlorodibenzo-p-dioxin (TCDD) in the rat and guinea pig. Toxicol. Appl. Pharm..

[738] Whysner J., Williams G.M. (1996). 2,3,7,8-Tetrachlorodibenzo-p-dioxin mechanistic data and risk assessment: Gene regulation, cytotoxicity, enhanced cell proliferation, and tumor promotion. Pharmacol. Ther..

[739] Patrizi B., Siciliani de Cumis M. (2018). TCDD toxicity mediated by epigenetic mechanisms. Int. J. Mol. Sci..

[740] Knerr S., Schrenk D. (2006). Carcinogenicity of 2,3,7,8-tetrachlorodibenzo-p-dioxin in experimental models. Mol. Nutr. Food Res..

[741] Lucier G., Clark G., Hiermath C., Tritscher A., Sewall C., Huff J. (1993). Carcinogenicity of TCDD in laboratory animals: Implications for risk assessment. Toxicol. Ind. Health.

[742] Schecter A., Startin J., Wright C., Kelly M., Päpke O., Lis A., Ball M., Olson J. (1994). Dioxins in U.S. food and estimated daily intake. Chemosphere.

[743] González N., Domingo J.L. (2021). Polychlorinated dibenzo-p-dioxins and dibenzofurans (PCDD/Fs) in food and human dietary intake: An update of the scientific literature. Food Chem. Toxicol..

[744] Danjou A.M., Fervers B., Boutron-Ruault M.C., Philip T., Clavel-Chapelon F., Dossus L. (2015). Estimated dietary dioxin exposure and breast cancer risk among women from the French E3N prospective cohort. Breast Cancer Res..

[745] Boffetta P., Mundt K.A., Adami H.O., Cole P., Mandel J.S. (2011). TCDD and cancer: A critical review of epidemiologic studies. Crit Rev. Toxicol..

[746] Cole P., Trichopoulos D., Pastides H., Starr T., Mandel J.S. (2003). Dioxin and cancer: A critical review. Regul. Toxicol. Pharm..

[747] WHO, World Health Organization (1998). Assessment of the Health Risk of Dioxins: Re-Evaluation of the Tolerable Daily Intake (TDI).

[748] van Leeuwen F.X., Feeley M., Schrenk D., Larsen J.C., Farland W., Younes M. (2000). Dioxins: WHO’s tolerable daily intake (TDI) revisited. Chemosphere.

[749] SCF, Scientific Committee on Food (2001). Opinion of the Scientific Committee on Food on the Risk Assessment of Dioxins and Dioxin-like PCBs in Food.

[750] EPA, US Environmental Protection Agency (2001). Dioxin Reassessment—An SAB Review of the Office of Research and Development’s Reassessment of Dioxin: Review of the Revised Sections (Dose Response Modeling, Integrated Summary, Risk Characterization, and Toxicity Equivalence Factors) of the EPA’s Reassessment of Dioxin by the Dioxin Reassessment Review Subcommittee of the EPA Science Advisory Board (SAB).

[751] Starr T.B. (2001). Significant shortcomings of the U.S. Environmental Protection Agency’s latest draft risk characterization for dioxin-like compounds. Toxicol. Sci..

[752] Paustenbach D.J. (2002). The U.S. EPA Science Advisory Board Evaluation (2001) of the EPA dioxin reassessment. Regul. Toxicol. Pharm..

[753] Popp J.A., Crouch E., McConnell E.E. (2006). A Weight-of-evidence analysis of the cancer dose-response characteristics of 2,3,7,8-tetrachlorodibenzodioxin (TCDD). Toxicol. Sci..

[754] NRC, National Research Council (2006). Health Risks from Dioxin and Related Compounds.

[755] EFSA CEP Panel, European Food Safety Authority, Panel on Food Contact Materials, Enzymes and Processing Aids (2017). Scientific Opinion on safety of benzophenone to be used as flavouring. EFSA J..

[756] Adams T.B., McGowen M.M., Williams M.C., Cohen S.M., Feron V.J., Goodman J.I., Marnett L.J., Munro I.C., Portoghese P.S., Smith R.L. (2007). The FEMA GRAS assessment of aromatic substituted secondary alcohols, ketones, and related esters used as flavor ingredients. Food Chem. Toxicol..

[757] Hu L., Tian M., Feng W., He H., Wang Y., Yang L. (2019). Sensitive detection of benzophenone-type ultraviolet filters in plastic food packaging materials by sheathless capillary electrophoresis-electrospray ionization-tandem mass spectrometry. J. Chromatogr. A.

[758] Anderson W.A., Castle L. (2003). Benzophenone in cartonboard packaging materials and the factors that influence its migration into food. Food Addit. Contam..

[759] Castle L., Damant A.P., Honeybone C.A., Johns S.M., Jickells S.M., Sharman M., Gilbert J. (1997). Migration studies from paper and board food packaging materials. Part 2. Survey for residues of dialkylamino benzophenone UV-cure ink photoinitiators. Food Addit. Contam..

[760] Jung T., Simat T.J., Altkofer W., Fügel D. (2013). Survey on the occurrence of photo-initiators and amine synergists in cartonboard packaging on the German market and their migration into the packaged foodstuffs. Food Addit. Contam. Part A Chem. Anal. Control Expo. Risk Assess..

[761] Rhodes M.C., Bucher J.R., Peckham J.C., Kissling G.E., Hejtmancik M.R., Chhabra R.S. (2007). Carcinogenesis studies of benzophenone in rats and mice. Food Chem. Toxicol..

[762] NTP, National Toxicology Program (2000). Technical Report on the Toxicity Studies of Benzophenone (CAS No. 119-61-9) Administered in Feed to F344/N Rats and B6C3F1 Mice 0888-8051 (Print).

[763] NTP, National Toxicology Program (2006). Toxicology and carcinogenesis studies of benzophenone (CAS No. 119-61-9) in F344/N rats and B6C3F1 mice (feed studies). Natl. Toxicol. Program. Tech. Rep. Ser..

[764] JECFA, Joint FAO/WHO Expert Committee on Food Additives (2011). Safety Evaluation of Certain Food Additives and Contaminants.

[765] Abramsson-Zetterberg L., Svensson K. (2011). 4-Methylbenzophenone and benzophenone are inactive in the micronucleus assay. Toxicol. Lett..

[766] Takemoto K., Yamazaki H., Nakajima M., Yokoi T. (2002). Genotoxic activation of benzophenone and its two metabolites by human cytochrome P450s in SOS/umu assay. Mutat. Res..

[767] Cuquerella M.C., Lhiaubet-Vallet V., Cadet J., Miranda M.A. (2012). Benzophenone photosensitized DNA damage. Acc. Chem. Res..

[768] Dumont E., Wibowo M., Roca-Sanjuán D., Garavelli M., Assfeld X., Monari A. (2015). Resolving the benzophenone DNA-photosensitization mechanism at QM/MM level. J. Phys. Chem. Lett..

[769] Nakagawa Y., Suzuki T., Tayama S. (2000). Metabolism and toxicity of benzophenone in isolated rat hepatocytes and estrogenic activity of its metabolites in MCF-7 cells. Toxicology.

[770] Jeon H.K., Sarma S.N., Kim Y.J., Ryu J.C. (2008). Toxicokinetics and metabolisms of benzophenone-type UV filters in rats. Toxicology.

[771] Nakagawa Y., Tayama K. (2002). Benzophenone-induced estrogenic potency in ovariectomized rats. Arch. Toxicol..

[772] Nakagawa Y., Tayama K. (2001). Estrogenic potency of benzophenone and its metabolites in juvenile female rats. Arch. Toxicol..

[773] Suzuki T., Kitamura S., Khota R., Sugihara K., Fujimoto N., Ohta S. (2005). Estrogenic and antiandrogenic activities of 17 benzophenone derivatives used as UV stabilizers and sunscreens. Toxicol. Appl. Pharm..

[774] Chen M.L., Chen C.H., Huang Y.F., Chen H.C., Chang J.W. (2022). Cumulative dietary risk assessment of benzophenone-type photoinitiators from packaged foodstuffs. Foods.

[775] Erythropel H.C., Maric M., Nicell J.A., Leask R.L., Yargeau V. (2014). Leaching of the plasticizer di(2-ethylhexyl)phthalate (DEHP) from plastic containers and the question of human exposure. Appl. Microbiol. Biotechnol..

[776] Serrano S.E., Braun J., Trasande L., Dills R., Sathyanarayana S. (2014). Phthalates and diet: A review of the food monitoring and epidemiology data. Environ. Health.

[777] ATSDR, Agency for Toxic Substances and Disease Registry (2022). Toxicological Profile for di(2-Ethylhexyl)Phthalate (DEHP).

[778] Fierens T., Servaes K., Van Holderbeke M., Geerts L., De Henauw S., Sioen I., Vanermen G. (2012). Analysis of phthalates in food products and packaging materials sold on the Belgian market. Food Chem. Toxicol..

[779] Sharman M., Read W.A., Castle L., Gilbert J. (1994). Levels of di-(2-ethylhexyl)phthalate and total phthalate esters in milk, cream, butter and cheese. Food Addit. Contam..

[780] EFSA CEP Panel, European Food Safety Authority, Panel on Food Contact Materials, Enzymes and Processing Aids (2019). Scientific Opinion on the update of the risk assessment of di-butylphthalate(DBP), butyl-benzyl-phthalate (BBP), bis(2-ethylhexyl)phthalate (DEHP), di-isononylphthalate (DINP) anddi-isodecylphthalate (DIDP) for use in food contact materials. EFSA J..

[781] Bosgra S., Bos P.M., Vermeire T.G., Luit R.J., Slob W. (2005). Probabilistic risk characterization: An example with di(2-ethylhexyl) phthalate. Regul. Toxicol. Pharm..

[782] Khedr A. (2013). Optimized extraction method for LC-MS determination of bisphenol A, melamine and di(2-ethylhexyl) phthalate in selected soft drinks, syringes, and milk powder. J. Chromatogr. B Anal. Technol. Biomed. Life Sci..

[783] NTP, National Toxicology Program (1982). Carcinogenesis bioassay of di(2-ethylhexyl)phthalate (CAS No. 117-81-7) in F344 rats and B6C3F1 mice (feed studies). Natl. Toxicol. Program. Tech. Rep. Ser..

[784] NTP, National Toxicology Program (2021). Toxicology and carcinogenesis studies of di(2-ethylhexyl) phthalate administered in feed to Sprague Dawley (Hsd:Sprague Dawley SD) rats. Natl. Toxicol. Program. Tech. Rep. Ser..

[785] Voss C., Zerban H., Bannasch P., Berger M.R. (2005). Lifelong exposure to di-(2-ethylhexyl)-phthalate induces tumors in liver and testes of Sprague-Dawley rats. Toxicology.

[786] Ito Y., Yamanoshita O., Asaeda N., Tagawa Y., Lee C.H., Aoyama T., Ichihara G., Furuhashi K., Kamijima M., Gonzalez F.J. (2007). Di(2-ethylhexyl)phthalate induces hepatic tumorigenesis through a peroxisome proliferator-activated receptor alpha-independent pathway. J. Occup. Health.

[787] Doull J., Cattley R., Elcombe C., Lake B.G., Swenberg J., Wilkinson C., Williams G., van Gemert M. (1999). A cancer risk assessment of di(2-ethylhexyl)phthalate: Application of the new U.S. EPA Risk Assessment Guidelines. Regul. Toxicol. Pharm..

[788] Ward J.M., Ohshima M., Lynch P., Riggs C. (1984). Di(2-ethylhexyl)phthalate but not phenobarbital promotes N-nitrosodiethylamine-initiated hepatocellular proliferative lesions after short-term exposure in male B6C3F1 mice. Cancer Lett..

[789] Diwan B.A., Ward J.M., Rice J.M., Colburn N.H., Spangler E.F. (1985). Tumor-promoting effects of di(2-ethylhexyl)phthalate in JB6 mouse epidermal cells and mouse skin. Carcinogenesis.

[790] Butterworth B.E., Bermudez E., Smith-Oliver T., Earle L., Cattley R., Martin J., Popp J.A., Strom S., Jirtle R., Michalopoulos G. (1984). Lack of genotoxic activity of di(2-ethylhexyl)phthalate (DEHP) in rat and human hepatocytes. Carcinogenesis.

[791] Caldwell J.C. (2012). DEHP: Genotoxicity and potential carcinogenic mechanisms-a review. Mutat. Res..

[792] Erkekoglu P., Kocer-Gumusel B. (2014). Genotoxicity of phthalates. Toxicol. Mech. Methods.

[793] Karabulut G., Barlas N. (2018). Genotoxic, histologic, immunohistochemical, morphometric and hormonal effects of di-(2-ethylhexyl)-phthalate (DEHP) on reproductive systems in pre-pubertal male rats. Toxicol. Res..

[794] Wang X., Jiang L., Ge L., Chen M., Yang G., Ji F., Zhong L., Guan Y., Liu X. (2015). Oxidative DNA damage induced by di-(2-ethylhexyl) phthalate in HEK-293 cell line. Environ. Toxicol. Pharm..

[795] Erkekoglu P., Rachidi W., Yuzugullu O.G., Giray B., Favier A., Ozturk M., Hincal F. (2010). Evaluation of cytotoxicity and oxidative DNA damaging effects of di(2-ethylhexyl)-phthalate (DEHP) and mono(2-ethylhexyl)-phthalate (MEHP) on MA-10 Leydig cells and protection by selenium. Toxicol. Appl. Pharm..

[796] She Y., Jiang L., Zheng L., Zuo H., Chen M., Sun X., Li Q., Geng C., Yang G., Jiang L. (2017). The role of oxidative stress in DNA damage in pancreatic β cells induced by di-(2-ethylhexyl) phthalate. Chem. Biol. Interact..

[797] Takagi A., Sai K., Umemura T., Hasegawa R., Kurokawa Y. (1990). Significant increase of 8-hydroxydeoxyguanosine in liver DNA of rats following short-term exposure to the peroxisome proliferators di(2-ethylhexyl)phthalate and di(2-ethylhexyl)adipate. Jpn. J. Cancer Res..

[798] Takagi A., Sai K., Umemura T., Hasegawa R., Kurokawa Y. (1990). Relationship between hepatic peroxisome proliferation and 8-hydroxydeoxyguanosine formation in liver DNA of rats following long-term exposure to three peroxisome proliferators; di(2-ethylhexyl) phthalate, aluminium clofibrate and simfibrate. Cancer Lett..

[799] von Däniken A., Lutz W.K., Jäckh R., Schlatter C. (1984). Investigation of the potential for binding of Di(2-ethylhexyl) phthalate (DEHP) and Di(2-ethylhexyl) adipate (DEHA) to liver DNA in vivo. Toxicol. Appl. Pharm..

[800] Lutz W.K. (1986). Investigation of the potential for binding of di(2-ethylhexyl) phthalate (DEHP) to rat liver DNA in vivo. Environ. Health Perspect..

[801] Gupta R.C., Goel S.K., Earley K., Singh B., Reddy J.K. (1985). 32P-postlabeling analysis of peroxisome proliferator-DNA adduct formation in rat liver in vivo and hepatocytes in vitro. Carcinogenesis.

[802] Koch H.M., Preuss R., Angerer J. (2006). Di(2-ethylhexyl)phthalate (DEHP): Human metabolism and internal exposure—An update and latest results. Int. J..

[803] Silva M.J., Samandar E., Preau J.L., Needham L.L., Calafat A.M. (2006). Urinary oxidative metabolites of di(2-ethylhexyl) phthalate in humans. Toxicology.

[804] Frederiksen H., Skakkebaek N.E., Andersson A.M. (2007). Metabolism of phthalates in humans. Mol. Nutr. Food Res..

[805] Ito Y., Yokota H., Wang R., Yamanoshita O., Ichihara G., Wang H., Kurata Y., Takagi K., Nakajima T. (2005). Species differences in the metabolism of di(2-ethylhexyl) phthalate (DEHP) in several organs of mice, rats, and marmosets. Arch. Toxicol..

[806] Ito Y., Kamijima M., Hasegawa C., Tagawa M., Kawai T., Miyake M., Hayashi Y., Naito H., Nakajima T. (2014). Species and inter-individual differences in metabolic capacity of di(2-ethylhexyl)phthalate (DEHP) between human and mouse livers. Environ. Health Prev. Med..

[807] Ito Y., Kamijima M., Nakajima T. (2019). Di(2-ethylhexyl) phthalate-induced toxicity and peroxisome proliferator-activated receptor alpha: A review. Environ. Health Prev. Med..

[808] Melnick R.L. (2001). Is peroxisome proliferation an obligatory precursor step in the carcinogenicity of di(2-ethylhexyl)phthalate (DEHP)?. Environ. Health Perspect..

[809] Corton J.C., Peters J.M., Klaunig J.E. (2018). The PPARα-dependent rodent liver tumor response is not relevant to humans: Addressing misconceptions. Arch. Toxicol..

[810] Rusyn I., Corton J.C. (2012). Mechanistic considerations for human relevance of cancer hazard of di(2-ethylhexyl) phthalate. Mutat. Res..

[811] Shelby M.D. (2006). NTP-CERHR monograph on the potential human reproductive and developmental effects of di (2-ethylhexyl) phthalate (DEHP). Ntp. Cerhr. Mon..

[812] Schecter A., Lorber M., Guo Y., Wu Q., Yun S.H., Kannan K., Hommel M., Imran N., Hynan L.S., Cheng D. (2013). Phthalate concentrations and dietary exposure from food purchased in New York State. Environ. Health Perspect..

[813] Qu J., Xia W., Qian X., Wu Y., Li J., Wen S., Xu S. (2022). Geographic distribution and time trend of human exposure of Di(2-ethylhexyl) phthalate among different age groups based on global biomonitoring data. Chemosphere.

[814] Morgan M., Deoraj A., Felty Q., Roy D. (2017). Environmental estrogen-like endocrine disrupting chemicals and breast cancer. Mol. Cell Endocrinol..

[815] Stickney J.A., Sager S.L., Clarkson J.R., Smith L.A., Locey B.J., Bock M.J., Hartung R., Olp S.F. (2003). An updated evaluation of the carcinogenic potential of 1,4-dioxane. Regul. Toxicol. Pharm..

[816] ATSDR, Agency for Toxic Substances and Disease Registry (2012). Toxicological Profile for 1,4-Dioxane.

[817] SCF, Scientific Committee on Food (2002). Opinion of the Scientific Committee on Food on Impurities of 1,4-Dioxane, 2-Chloroethanol and Mono- and Diethylene Glycol in Currently Permitted Food Additives and in Proposed Use of Ethyl Hydroxyethyl Cellulose in Gluten-Free Bread.

[818] Nishimura T., Iizuka S., Kibune N., Ando M. (2004). Study of 1,4-dioxane intake in the total diet using the market-basket method. J. Health Sci..

[819] NCI, National Cancer Institute (1978). Bioassay of 1,4-dioxane for possible carcinogenicity. Natl. Cancer Inst. Carcinog. Tech. Rep. Ser..

[820] Kano H., Umeda Y., Kasai T., Sasaki T., Matsumoto M., Yamazaki K., Nagano K., Arito H., Fukushima S. (2009). Carcinogenicity studies of 1,4-dioxane administered in drinking-water to rats and mice for 2 years. Food Chem. Toxicol..

[821] Kano H., Umeda Y., Saito M., Senoh H., Ohbayashi H., Aiso S., Yamazaki K., Nagano K., Fukushima S. (2008). Thirteen-week oral toxicity of 1,4-dioxane in rats and mice. J. Toxicol. Sci..

[822] Lundberg I., Högberg J., Kronevi T., Holmberg B. (1987). Three industrial solvents investigated for tumor promoting activity in the rat liver. Cancer Lett..

[823] Rosenkranz H.S., Klopman G. (1992). 1,4-Dioxane: Prediction of in vivo clastogenicity. Mutat. Res..

[824] Mirkova E.T. (1994). Activity of the rodent carcinogen 1,4-dioxane in the mouse bone marrow micronucleus assay. Mutat. Res..

[825] Kitchin K.T., Brown J.L. (1990). Is 1,4-dioxane a genotoxic carcinogen?. Cancer Lett..

[826] Morita T., Hayashi M. (1998). 1,4-Dioxane is not mutagenic in five in vitro assays and mouse peripheral blood micronucleus assay, but is in mouse liver micronucleus assay. Environ. Mol. Mutagen..

[827] Itoh S., Hattori C. (2019). In vivo genotoxicity of 1,4-dioxane evaluated by liver and bone marrow micronucleus tests and Pig-a assay in rats. Mutat. Res. Genet. Toxicol. Environ. Mutagen..

[828] Gi M., Fujioka M., Kakehashi A., Okuno T., Masumura K., Nohmi T., Matsumoto M., Omori M., Wanibuchi H., Fukushima S. (2018). In vivo positive mutagenicity of 1,4-dioxane and quantitative analysis of its mutagenicity and carcinogenicity in rats. Arch. Toxicol..

[829] Charkoftaki G., Golla J.P., Santos-Neto A., Orlicky D.J., Garcia-Milian R., Chen Y., Rattray N.J.W., Cai Y., Wang Y., Shearn C.T. (2021). Identification of dose-dependent DNA damage and repair responses from subchronic exposure to 1,4-dioxane in mice using a systems analysis approach. Toxicol. Sci..

[830] Roy S.K., Thilagar A.K., Eastmond D.A. (2005). Chromosome breakage is primarily responsible for the micronuclei induced by 1,4-dioxane in the bone marrow and liver of young CD-1 mice. Mutat. Res..

[831] Totsuka Y., Maesako Y., Ono H., Nagai M., Kato M., Gi M., Wanibuchi H., Fukushima S., Shiizaki K., Nakagama H. (2020). Comprehensive analysis of DNA adducts (DNA adductome analysis) in the liver of rats treated with 1,4-dioxane. Proc. Jpn. Acad. Ser. B.

[832] Göen T., von Helden F., Eckert E., Knecht U., Drexler H., Walter D. (2016). Metabolism and toxicokinetics of 1,4-dioxane in humans after inhalational exposure at rest and under physical stress. Arch. Toxicol..

[833] Nannelli A., De Rubertis A., Longo V., Gervasi P.G. (2005). Effects of dioxane on cytochrome P450 enzymes in liver, kidney, lung and nasal mucosa of rat. Arch. Toxicol..

[834] Lafranconi M., Budinsky R., Corey L., Klapacz J., Crissman J., LeBaron M., Golden R., Pleus R. (2021). A 90-day drinking water study in mice to characterize early events in the cancer mode of action of 1,4-dioxane. Regul. Toxicol. Pharm..

[835] Dourson M.L., Higginbotham J., Crum J., Burleigh-Flayer H., Nance P., Forsberg N.D., Lafranconi M., Reichard J. (2017). Update: Mode of action (MOA) for liver tumors induced by oral exposure to 1,4-dioxane. Regul Toxicol Pharm..

[836] Dourson M., Reichard J., Nance P., Burleigh-Flayer H., Parker A., Vincent M., McConnell E.E. (2014). Mode of action analysis for liver tumors from oral 1,4-dioxane exposures and evidence-based dose response assessment. Regul. Toxicol. Pharm..

[837] Chappell G.A., Heintz M.M., Haws L.C. (2021). Transcriptomic analyses of livers from mice exposed to 1,4-dioxane for up to 90 days to assess potential mode(s) of action underlying liver tumor development. Curr. Res. Toxicol..

[838] Chen Y., Wang Y., Charkoftaki G., Orlicky D.J., Davidson E., Wan F., Ginsberg G., Thompson D.C., Vasiliou V. (2022). Oxidative stress and genotoxicity in 1,4-dioxane liver toxicity as evidenced in a mouse model of glutathione deficiency. Sci. Total Environ..

[839] Goldsworthy T.L., Monticello T.M., Morgan K.T., Bermudez E., Wilson D.M., Jäckh R., Butterworth B.E. (1991). Examination of potential mechanisms of carcinogenicity of 1,4-dioxane in rat nasal epithelial cells and hepatocytes. Arch. Toxicol..

[840] FDA, Food and Drug Administration (1998). Indirect Food Additives: Adhesives and Components of Coatings.

[841] Nishimura T., Iizuka S., Kibune N., Ando M., Magara Y. (2005). Study of 1,4-dioxane intake in the total diet. J. Health Sci..

[842] EFSA CONTAM Panel, European Food Safety Authority, Panel on Contaminants in the Food Chain (2012). Scientific Opinion on the evaluation of the substances currently on the list in the annex to Commission Directive 96/3/EC as acceptable previous cargoes for edible fats and oils—Part II of III. EFSA J..

[843] Johnson W. (2004). Safety assessment of MIBK (methyl isobutyl ketone). Int. J. Toxicol..

[844] Api A.M., Belsito D., Botelho D., Bruze M., Burton G.A., Buschmann J., Dagli M.L., Date M., Dekant W., Deodhar C. (2019). RIFM fragrance ingredient safety assessment, 4-methyl-2-pentanone, CAS Registry Number 108-10-1. Food Chem. Toxicol..

[845] Stout M.D., Herbert R.A., Kissling G.E., Suarez F., Roycroft J.H., Chhabra R.S., Bucher J.R. (2008). Toxicity and carcinogenicity of methyl isobutyl ketone in F344N rats and B6C3F1 mice following 2-year inhalation exposure. Toxicology.

[846] NTP, National Toxicology Program (2007). Toxicology and carcinogenesis studies of methyl isobutyl ketone (Cas No. 108-10-1) in F344/N rats and B6C3F1 mice (inhalation studies). Natl. Toxicol. Program. Tech. Rep. Ser..

[847] Brooks T.M., Meyer A.L., Hutson D.H. (1988). The genetic toxicology of some hydrocarbon and oxygenated solvents. Mutagenesis.

[848] O’Donoghue J.L., Haworth S.R., Curren R.D., Kirby P.E., Lawlor T., Moran E.J., Phillips R.D., Putnam D.L., Rogers-Back A.M., Slesinski R.S. (1988). Mutagenicity studies on ketone solvents: Methyl ethyl ketone, methyl isobutyl ketone, and isophorone. Mutat. Res..

[849] JECFA, Joint FAO/WHO Expert Committee on Food Additives (1999). Safety Evaluation of Certain Food Additives.

[850] Granvil C.P., Sharkawi M., Plaa G.L. (1994). Metabolic fate of methyl n-butyl ketone, methyl isobutyl ketone and their metabolites in mice. Toxicol. Lett..

[851] Duguay A.B., Plaa G.L. (1993). Plasma concentrations in methyl isobutyl ketone-potentiated experimental cholestasis after inhalation or oral administration. Fundam. Appl. Toxicol..

[852] Duguay A.B., Plaa G.L. (1995). Tissue concentrations of methyl isobutyl ketone, methyl n-butyl ketone and their metabolites after oral or inhalation exposure. Toxicol. Lett..

[853] Vézina M., Kobusch A.B., du Souich P., Greselin E., Plaa G.L. (1990). Potentiation of chloroform-induced hepatotoxicity by methyl isobutyl ketone and two metabolites. Can. J. Physiol. Pharm..

[854] Gingell R., Régnier J.F., Wilson D.M., Guillaumat P.O., Appelqvist T. (2003). Comparative metabolism of methyl isobutyl carbinol and methyl isobutyl ketone in male rats. Toxicol. Lett..

[855] Raymond P., Plaa G.L. (1995). Ketone potentiation of haloalkane-induced hepato- and nephrotoxicity. I. Dose-response relationships. J. Toxicol. Environ. Health.

[856] Raymond P., Plaa G.L. (1995). Ketone potentiation of haloalkane-induced hepato- and nephrotoxicity. II. Implication of monooxygenases. J. Toxicol. Environ. Health.

[857] Borghoff S.J., Poet T.S., Green S., Davis J., Hughes B., Mensing T., Sarang S.S., Lynch A.M., Hard G.C. (2015). Methyl isobutyl ketone exposure-related increases in specific measures of α2u-globulin (α2u) nephropathy in male rats along with in vitro evidence of reversible protein binding. Toxicology.

[858] Borghoff S.J., Hard G.C., Berdasco N.M., Gingell R., Green S.M., Gulledge W. (2009). Methyl isobutyl ketone (MIBK) induction of alpha2u-globulin nephropathy in male, but not female rats. Toxicology.

[859] Vézina M., Plaa G.L. (1987). Potentiation by methyl isobutyl ketone of the cholestasis induced in rats by a manganese-bilirubin combination or manganese alone. Toxicol. Appl. Pharm..

[860] Joseph L.D., Yousef I.M., Plaa G.L., Sharkawi M. (1992). Potentiation of lithocholic-acid-induced cholestasis by methyl isobutyl ketone. Toxicol. Lett..

[861] Hughes B.J., Thomas J., Lynch A.M., Borghoff S.J., Green S., Mensing T., Sarang S.S., LeBaron M.J. (2016). Methyl isobutyl ketone-induced hepatocellular carcinogenesis in B6C3F(1) mice: A constitutive androstane receptor (CAR)-mediated mode of action. Regul. Toxicol. Pharm..

[862] Grober E., Schaumburg H.H. (2000). Occupational exposure to methyl isobutyl ketone causes lasting impairment in working memory. Neurology.

[863] Wong J.M., Bernhard R.A. (1988). Effect of nitrogen source on pyrazine formation. J. Agric. Food Chem..

[864] Wu X., Huang M., Kong F., Yu S. (2015). Short communication: Study on the formation of 2-methylimidazole and 4-methylimidazole in the Maillard reaction. J. Dairy Sci..

[865] Chan P.C. (2004). NTP technical report on the toxicity studies of 2- and 4-Methylimidazole (CAS No. 693-98-1 and 822-36-6) administered in feed to F344/N rats and B6C3F1 mice. Toxic Rep. Ser..

[866] Schlee C., Markova M., Schrank J., Laplagne F., Schneider R., Lachenmeier D.W. (2013). Determination of 2-methylimidazole, 4-methylimidazole and 2-acetyl-4-(1,2,3,4-tetrahydroxybutyl)imidazole in caramel colours and cola using LC/MS/MS. J. Chromatogr. B Anal. Technol. Biomed. Life Sci..

[867] Jacobs G., Voorspoels S., Vloemans P., Fierens T., Van Holderbeke M., Cornelis C., Sioen I., De Maeyer M., Vinkx C., Vanermen G. (2018). Caramel colour and process by-products in foods and beverages: Part I—Development of a UPLC-MS/MS isotope dilution method for determination of 2-acetyl-4-(1,2,3,4-tetrahydroxybutyl)imidazole (THI), 4-methylimidazole (4-MEI) and 2-methylimidazol (2-MEI). Food Chem..

[868] Choi S.J., Jung M.Y. (2017). Simple and fast sample preparation followed by Gas Chromatography-Tandem Mass Spectrometry (GC-MS/MS) for the analysis of 2- and 4-methylimidazole in cola and dark beer. J. Food Sci..

[869] JECFA, Joint FAO/WHO Expert Committee on Food Additives (1985). Toxicological Evaluation of Certain Food Additives and Contaminants.

[870] EFSA ANS Panel, European Food Safety Authority, Panel on Food Additives and Nutrient Sources added to Food (2011). Scientific Opinion on the re-evaluation of caramel colours (E 150a,b,c,d) as food additives. EFSA J..

[871] Vollmuth T.A. (2018). Caramel color safety—An update. Food Chem. Toxicol..

[872] Morgan S.E., Edwards W.C. (1986). Pilot studies in cattle and mice to determine the presence of 4-methylimidazole in milk after oral ingestion. Vet. Hum. Toxicol..

[873] Müller L., Sivertsen T., Langseth W. (1998). Ammoniated forage poisoning: Concentrations of alkylimidazoles in ammoniated forage and in milk, plasma and urine in sheep and cow. Acta Vet. Scand..

[874] Mottier P., Mujahid C., Tarres A., Bessaire T., Stadler R.H. (2017). Process-induced formation of imidazoles in selected foods. Food Chem..

[875] NTP, National Toxicology Program (2007). Toxicology and carcinogenesis studies of 4-methylimidazole (Cas No. 822-36-6) in F344/N rats and B6C3F1 mice (feed studies). Natl. Toxicol. Program. Tech. Rep. Ser..

[876] Chan P.C., Sills R.C., Kissling G.E., Nyska A., Richter W. (2008). Induction of thyroid and liver tumors by chronic exposure to 2-methylimidazole in F344/N rats and B6C3F1 mice. Arch. Toxicol..

[877] Chan P.C., Hill G.D., Kissling G.E., Nyska A. (2008). Toxicity and carcinogenicity studies of 4-methylimidazole in F344/N rats and B6C3F1 mice. Arch. Toxicol..

[878] NTP, National Toxicology Program (2004). Toxicology and carcinogensis studies of 2-methylimidazole (Cas No. 693-98-1) in B6C3F1 mice (feed studies). Natl. Toxicol. Program. Tech. Rep. Ser..

[879] Chan P., Mahler J., Travlos G., Nyska A., Wenk M. (2006). Induction of thyroid lesions in 14-week toxicity studies of 2 and 4-methylimidazole in Fischer 344/N rats and B6C3F1 mice. Arch. Toxicol..

[880] Beevers C., Adamson R.H. (2016). Evaluation of 4-methylimidazole, in the Ames/Salmonella test using induced rodent liver and lung S9. Environ. Mol. Mutagen..

[881] Morita T., Uneyama C. (2016). Genotoxicity assessment of 4-methylimidazole: Regulatory perspectives. Genes Environ..

[882] Celik R., Topaktas M. (2018). Genotoxic effects of 4-methylimidazole on human peripheral lymphocytes in vitro. Drug Chem. Toxicol..

[883] Johnson J.D., Reichelderfer D., Zutshi A., Graves S., Walters D., Smith C. (2002). Toxicokinetics of 2-methylimidazole in male and female F344 rats. J. Toxicol. Environ. Health Part A.

[884] Fennell T.R., Watson S.L., Dhungana S., Snyder R.W. (2019). Metabolism of 4-methylimidazole in Fischer 344 rats and B6C3F1 mice. Food Chem. Toxicol.

[885] Sanders J.M., Griffin R.J., Burka L.T., Matthews H.B. (1998). Disposition of 2-methylimidazole in rats. J. Toxicol. Environ. Health A.

[886] Dalvie D.K., Kalgutkar A.S., Khojasteh-Bakht S.C., Obach R.S., O’Donnell J.P. (2002). Biotransformation reactions of five-membered aromatic heterocyclic rings. Chem. Res. Toxicol..

[887] Ohta K., Fukasawa Y., Yamaguchi J., Kohno Y., Fukushima K., Suwa T., Awazu S. (1998). Retention mechanism of imidazoles in connective tissue. IV. Identification of a nucleophilic imidazolone metabolite in rats. Biol. Pharm. Bull..

[888] Capen C.C. (1994). Mechanisms of chemical injury of thyroid gland. Prog. Clin. Biol Res..

[889] Williams G.M., Iatropoulos M.J. (2002). Alteration of liver cell function and proliferation: Differentiation between adaptation and toxicity. Toxicol. Pathol..

[890] Borghoff S.J., Fitch S.E., Black M.B., McMullen P.D., Andersen M.E., Chappell G.A. (2021). A systematic approach to evaluate plausible modes of actions for mouse lung tumors in mice exposed to 4-methylimidozole. Regul. Toxicol. Pharm..

[891] Brusick D., Aardema M.J., Allaben W.T., Kirkland D.J., Williams G., Llewellyn G.C., Parker J.M., Rihner M.O. (2020). A weight of evidence assessment of the genotoxic potential of 4-methylimidazole as a possible mode of action for the formation of lung tumors in exposed mice. Food Chem. Toxicol..

[892] Folmer D.E., Doell D.L., Lee H.S., Noonan G.O., Carberry S.E. (2018). A U.S. population dietary exposure assessment for 4-methylimidazole (4-MEI) from foods containing caramel colour and from formation of 4-MEI through the thermal treatment of food. Food Addit. Contam. Part A Chem. Anal. Control Expo. Risk Assess..

[893] Fierens T., Van Holderbeke M., Cornelis C., Jacobs G., Sioen I., De Maeyer M., Vinkx C., Vanermen G. (2018). Caramel colour and process contaminants in foods and beverages: Part II—Occurrence data and exposure assessment of 2-acetyl-4-(1,2,3,4-tetrahydroxybutyl)imidazole (THI) and 4-methylimidazole (4-MEI) in Belgium. Food Chem..

[894] JECFA, Joint FAO/WHO Expert Committee on Food Additives (2011). Combined Compendium of Food Additive Specification.

[895] Yaylayan V.A. (2006). Precursors, formation and determination of furan in food. J. Verbrauch. Lebensm..

[896] EFSA CONTAM Panel, European Food Safety Authority, Panel on Contaminants in the Food Chain (2017). Scientific opinion on the risks for publichealth related to the presence of furan and methylfurans in food. EFSA J..

[897] Moro S., Chipman J.K., Wegener J.W., Hamberger C., Dekant W., Mally A. (2012). Furan in heat-treated foods: Formation, exposure, toxicity, and aspects of risk assessment. Mol. Nutr. Food Res..

[898] FDA, Food and Drug Administration (2009). Exploratory Data on Furan in Food: Individual Food Products.

[899] Van Lancker F., Adams A., Owczarek A., De Meulenaer B., De Kimpe N. (2009). Impact of various food ingredients on the retention of furan in foods. Mol. Nutr. Food Res..

[900] NTP, National Toxicology Program (1993). Toxicology and Carcinogenesis Studies of Furan (CAS No. 110-00-9) in F344 Rats and B6C3F1 Mice(Gavage Studies).

[901] IARC, International Agency for Research on Cancer (1995). Dry cleaning, some chlorinated solvents and other industrial chemicals. IARC Monographs on the Evaluation of Carcinogenic Risk to Humans.

[902] Von Tungeln L.S., Walker N.J., Olson G.R., Mendoza M.C., Felton R.P., Thorn B.T., Marques M.M., Pogribny I.P., Doerge D.R., Beland F.A. (2017). Low dose assessment of the carcinogenicity of furan in male F344/N Nctr rats in a 2-year gavage study. Food Chem. Toxicol..

[903] Moser G.J., Foley J., Burnett M., Goldsworthy T.L., Maronpot R. (2009). Furan-induced dose-response relationships for liver cytotoxicity, cell proliferation, and tumorigenicity (furan-induced liver tumorigenicity). Exp. Toxicol. Pathol..

[904] Durling L., Svensson K., Abramssonzetterberg L. (2007). Furan is not genotoxic in the micronucleus assay in vivo or in vitro. Toxicol. Lett..

[905] Wilson D.M., Goldsworthy T.L., Popp J.A., Butterworth B.E. (1992). Evaluation of genotoxicity, pathological lesions, and cell proliferation in livers of rats and mice treated with furan. Environ. Mol. Mutagen..

[906] McDaniel L.P., Ding W., Dobrovolsky V.N., Shaddock J.G., Mittelstaedt R.A., Doerge D.R., Heflich R.H. (2012). Genotoxicity of furan in Big Blue rats. Mutat. Res..

[907] Ding W., Petibone D.M., Latendresse J.R., Pearce M.G., Muskhelishvili L., White G.A., Chang C.W., Mittelstaedt R.A., Shaddock J.G., McDaniel L.P. (2012). In vivo genotoxicity of furan in F344 rats at cancer bioassay doses. Toxicol. Appl. Pharm..

[908] Jeffrey A.M., Brunnemann K.D., Duan J.D., Schlatter J., Williams G.M. (2012). Furan induction of DNA cross-linking and strand breaks in turkey fetal liver in comparison to 1,3-propanediol. Food Chem. Toxicol..

[909] Peterson L.A., Naruko K.C., Predecki D.P. (2000). A reactive metabolite of furan, cis-2-butene-1,4-dial, is mutagenic in the Ames assay. Chem. Res. Toxicol..

[910] Byrns M.C., Vu C.C., Neidigh J.W., Abad J.-L., Jones R.A., Peterson L.A. (2006). Detection of DNA adducts derived from the reactive metabolite of furan, cis-2-butene-1,4-dial. Chem. Res. Toxicol..

[911] Churchwell M.I., Scheri R.C., Von Tungeln L.S., Gamboa da Costa G., Beland F.A., Doerge D.R. (2015). Evaluation of serum and liver toxicokinetics for furan and liver DNA adduct formation in male Fischer 344 rats. Food Chem. Toxicol..

[912] Neuwirth C., Mosesso P., Pepe G., Fiore M., Malfatti M., Turteltaub K., Dekant W., Mally A. (2012). Furan carcinogenicity: DNA binding and genotoxicity of furan in rats in vivo. Mol. Nutr. Food Res..

[913] Kedderis G.L., Carfagna M.A., Held S.D., Batra R., Murphy J.E., Gargas M.L. (1993). Kinetic analysis of furan biotransformation by F-344 rats in vivo and in vitro. Toxicol. Appl. Pharm..

[914] Chen L.J., Hecht S.S., Peterson L.A. (1995). Identification of cis-2-butene-1,4-dial as a microsomal metabolite of furan. Chem. Res. Toxicol..

[915] Byrns M.C., Predecki D.P., Peterson L.A. (2002). Characterization of nucleoside adducts of cis-2-butene-1,4-dial, a reactive metabolite of furan. Chem. Res. Toxicol..

[916] Russo M.T., De Luca G., Palma N., Leopardi P., Degan P., Cinelli S., Pepe G., Mosesso P., Di Carlo E., Sorrentino C. (2021). Oxidative stress, mutations and chromosomal aberrations induced by in vitro and in vivo exposure to furan. Int. J. Mol. Sci..

[917] De Conti A., Kobets T., Escudero-Lourdes C., Montgomery B., Tryndyak V., Beland F.A., Doerge D.R., Pogribny I.P. (2014). Dose- and time-dependent epigenetic changes in the livers of Fisher 344 rats exposed to furan. Toxicol. Sci..

[918] de Conti A., Kobets T., Tryndyak V., Burnett S.D., Han T., Fuscoe J.C., Beland F.A., Doerge D.R., Pogribny I.P. (2015). Persistence of furan-induced epigenetic aberrations in the livers of F344 rats. Toxicol. Sci..

[919] FDA, Food and Drug Administration (2007). An Updated Exposure Assessment for Furan from the Consumption of Adult and Baby Foods.

[920] Lachenmeier D.W., Maser E., Kuballa T., Reusch H., Kersting M., Alexy U. (2012). Detailed exposure assessment of dietary furan for infants consuming commercially jarred complementary food based on data from the DONALD study. Matern Child. Nutr..

[921] IFIC, International Food Information Council, FDA, US Food and Drug Administration (2010). Overview of Food Ingredients, Additives & Colors.

[922] Krishan M., Navarro L., Beck B., Carvajal R., Dourson M. (2021). A regulatory relic: After 60 years of research on cancer risk, the Delaney Clause continues to keep us in the past. Toxicol. Appl. Pharm..

[923] Williams G.M., Karbe E., Fenner-Crisp P., Iatropoulos M.J., Weisburger J.H. (1996). Risk assessment of carcinogens in food with special consideration of non-genotoxic carcinogens. Exp. Toxicol. Pathol..

[924] Felter S.P., Zhang X., Thompson C. (2021). Butylated hydroxyanisole: Carcinogenic food additive to be avoided or harmless antioxidant important to protect food supply?. Regul. Toxicol. Pharm..

[925] Xu X., Liu A., Hu S., Ares I., Martínez-Larrañaga M.R., Wang X., Martínez M., Anadón A., Martínez M.A. (2021). Synthetic phenolic antioxidants: Metabolism, hazards and mechanism of action. Food Chem..

[926] EFSA ANS Panel, European Food Safety Authority, Panel on Food Additives and Nutrient Sources added to Food (2011). Scientific Opinion on the reevaluation of butylated hydroxyanisole-BHA (E 320) as a food additive. EFSA J..

[927] EFSA ANS Panel, European Food Safety Authority, Panel on Food Additives and Nutrient Sources added to Food (2012). Scientific Opinion on the reevaluation of butylated hydroxytoluene BHT (E 321) as a food additive. EFSA J..

[928] EFSA ANS Panel, European Food Safety Authority, Panel on Food Additives and Nutrient Sources added to Food (2016). Statement on the refined exposure assessment of tertiary-butyl hydroquinone (E 319). EFSA J..

[929] Deisinger P.J. (1996). Human exposure to naturally occurring hydroquinone. J. Toxicol. Environ. Health.

[930] Whysner J. (1996). Butylated hydroxyanisole mechanistic data and risk assessment: Conditional species-specific cytotoxicity, enhanced cell proliferation, and tumor promotion. Pharmacol. Ther..

[931] Williams G.M., Iatropoulos M.J., Whysner J. (1999). Safety assessment of butylated hydroxyanisole and butylated hydroxytoluene as antioxidant food additives. Food Chem. Toxicol..

[932] Hirose M., Fukushima S., Sakata T., Inui M., Ito N. (1983). Effect of quercetin on two-stage carcinogenesis of the rat urinary bladder. Cancer Lett..

[933] Gharavi N., Haggarty S., El-Kadi A.S. (2007). Chemoprotective and carcinogenic effects of tert-butylhydroquinone and its metabolites. Curr. Drug Metab..

[934] NTP, National Toxicology Program (1989). Toxicology and carcinogenesis studies of hydroquinone (CAS No. 123-31-9) in F344/N rats and B6C3F1 mice (gavage studies). Natl. Toxicol. Program. Tech. Rep. Ser..

[935] Lanigan R.S., Yamarik T.A. (2002). Final report on the safety assessment of BHT(1). Int. J. Toxicol..

[936] Williams G.M., McQueen C.A., Tong C. (1990). Toxicity studies of butylated hydroxyanisole and butylated hydroxytoluene. I. Genetic and cellular effects. Food Chem. Toxicol..

[937] van Esch G.J. (1986). Toxicology of tert-butylhydroquinone (TBHQ). Food Chem. Toxicol..

[938] Eskandani M., Hamishehkar H., Ezzati Nazhad Dolatabadi J. (2014). Cytotoxicity and DNA damage properties of tert-butylhydroquinone (TBHQ) food additive. Food Chem..

[939] Saito K., Nakagawa S., Yoshitake A., Miyamoto J., Hirose M., Ito N. (1989). DNA-adduct formation in the forestomach of rats treated with 3-tert-butyl-4-hydroxyanisole and its metabolites as assessed by an enzymatic 32P-postlabeling method. Cancer Lett..

[940] Jagetia G.C., Menon K.S.L., Jain V. (2001). Genotoxic effect of hydroquinone on the cultured mouse spleenocytes. Toxicol. Lett..

[941] Peters M.M., Lau S.S., Dulik D., Murphy D., van Ommen B., van Bladeren P.J., Monks T.J. (1996). Metabolism of tert-butylhydroquinone to S-substituted conjugates in the male Fischer 344 rat. Chem. Res. Toxicol..

[942] Malkinson A., Radcliffe R., Bauer A., Dwyer-Nield L., Kleeberger S. (2002). Quantitative trait loci that regulate susceptibility to both butylated hydroxytoluene-induced pulmonary inflammation and lung tumor promotion in CXB recombinant inbred mice. Chest.

[943] Whysner J., Wang C.X., Zang E., Iatropoulos M.J., Williams G.M. (1994). Dose response of promotion by butylated hydroxyanisole in chemically initiated tumours of the rat forestomach. Food Chem. Toxicol..

[944] Bauer A.K., Dwyer-Nield L.D., Keil K., Koski K., Malkinson A.M. (2001). Butylated hydroxytoluene (BHT) induction of pulmonary inflammation: A role in tumor promotion. Exp. Lung Res..

[945] Malkinson A.M. (2005). Role of inflammation in mouse lung tumorigenesis: A review. Exp. Lung Res..

[946] English J.C., Perry L.G., Vlaovic M., Moyer C., O’Donoghue J.L. (1994). Measurement of cell proliferation in the kidneys of Fischer 344 and Sprague-Dawley rats after gavage administration of hydroquinone. Fundam. Appl. Toxicol..

[947] Whysner J., Verna L., English J.C., Williams G.M. (1995). Analysis of studies related to tumorigenicity induced by hydroquinone. Regul. Toxicol. Pharmacol..

[948] Peters M. (1997). Cytotoxicity and cell-proliferation induced by the nephrocarcinogen hydroquinone and its nephrotoxic metabolite 2,3,5-(tris-glutathion-S- yl)hydroquinone. Carcinogenesis.

[949] Hard G.C., Whysner J., English J.C., Zang E., Williams G.M. (1997). Relationship of hydroquinone-associated rat renal tumors with spontaneous chronic progressive nephropathy. Toxicol. Pathol..

[950] Botterweck A.A., Verhagen H., Goldbohm R.A., Kleinjans J., van den Brandt P.A. (2000). Intake of butylated hydroxyanisole and butylated hydroxytoluene and stomach cancer risk: Results from analyses in the Netherlands Cohort Study. Food Chem. Toxicol..

[951] IARC, International Agency for Research on Cancer (2004). Cruciferous Vegetables, Isothiocyanates and Indoles.

[952] Williams G.M., Iatropoulos M.J., Jeffrey A.M. (2002). Anticarcinogenicity of monocyclic phenolic compounds. Eur. J. Cancer Prev..

[953] O’Donoghue J.L. (2006). Hydroquinone and its analogues in dermatology—A risk-benefit viewpoint. J. Cosmet. Dermatol..

[954] Suh H.J., Chung M.S., Cho Y.H., Kim J.W., Kim D.H., Han K.W., Kim C.J. (2005). Estimated daily intakes of butylated hydroxyanisole (BHA), butylated hydroxytoluene (BHT) andtert-butyl hydroquinone (TBHQ) antioxidants in Korea. Food Addit. Contam..

[955] Haque A., Brazeau D., Amin A.R. (2021). Perspectives on natural compounds in chemoprevention and treatment of cancer: An update with new promising compounds. Eur. J. Cancer.

[956] Langner E., Rzeski W. (2012). Dietary derived compounds in cancer chemoprevention. Contemp. Oncol..

[957] Murakami A., Ohigashi H., Koshimizu K. (1996). Anti-tumor promotion with food phytochemicals: A strategy for cancer chemoprevention. Biosci. Biotechnol. Biochem..

[958] Ranjan A., Ramachandran S., Gupta N., Kaushik I., Wright S., Srivastava S., Das H., Srivastava S., Prasad S., Srivastava S.K. (2019). Role of phytochemicals in cancer prevention. Int. J. Mol. Sci..

[959] Wattenberg L.W. (1990). Inhibition of carcinogenesis by naturally-occurring and synthetic compounds. Basic Life Sci..

[960] Newman D.J., Cragg G.M. (2020). Natural products as sources of new drugs over the nearly four decades from 01/1981 to 09/2019. J. Nat. Prod..

[961] Gescher A.J., Sharma R.A., Steward W.P. (2001). Cancer chemoprevention by dietary constituents: A tale of failure and promise. Lancet Oncol..

[962] Potter J.D. (2014). The failure of cancer chemoprevention. Carcinogenesis.

[963] Steward W.P., Brown K. (2013). Cancer chemoprevention: A rapidly evolving field. Br. J. Cancer.

[964] Patterson S.L., Colbert Maresso K., Hawk E. (2013). Cancer chemoprevention: Successes and failures. Clin. Chem..

[965] Johnson I.T. (2007). Phytochemicals and cancer. Proc. Nutr. Soc..

[966] Kleiner H.E. (2001). Oral administration of naturally occurring coumarins leads to altered phase I and II enzyme activities and reduced DNA adduct formation by polycyclic aromatic hydrocarbons in various tissues of SENCAR mice. Carcinogenesis.

[967] Prince M., Campbell C.T., Robertson T.A., Wells A.J., Kleiner H.E. (2005). Naturally occurring coumarins inhibit 7,12-dimethylbenz[a]anthracene DNA adduct formation in mouse mammary gland. Carcinogenesis.

[968] Huber W.W., McDaniel L.P., Kaderlik K.R., Teitel C.H., Lang N.P., Kadlubar F.F. (1997). Chemoprotection against the formation of colon DNA adducts from the food-borne carcinogen 2-amino-1-methyl-6-phenylimidazo[4,5-b]pyridine (PhIP) in the rat. Mutat. Res./Fundam. Mol. Mech. Mutagen..

[969] Cavin C. (1998). The coffee-specific diterpenes cafestol and kahweol protect against aflatoxin B1-induced genotoxicity through a dual mechanism. Carcinogenesis.

[970] Miller E.G., McWhorter K., Rivera-Hidalgo F., Wright J.M., Hirsbrunner P., Sunahara G.I. (1991). Kahweol and cafestol: Inhibitors of hamster buccal pouch carcinogenesis. Nutr. Cancer.

[971] Alhusainy W., Williams G.M., Jeffrey A.M., Iatropoulos M.J., Taylor S., Adams T.B., Rietjens I.M.C.M. (2014). The natural basil flavonoid nevadensin protects against a methyleugenol-induced marker of hepatocarcinogenicity in male F344 rat. Food Chem. Toxicol..

[972] Azuine M.A., Bhide S.V. (1992). Chemopreventive effect of turmeric against stomach and skin tumors induced by chemical carcinogens in Swiss mice. Nutr. Cancer.

[973] Ikezaki S., Nishikawa A., Furukawa F., Kudo K., Nakamura H., Tamura K., Mori H. (2001). Chemopreventive effects of curcumin on glandular stomach carcinogenesis induced by N-methyl-N’-nitro-N-nitrosoguanidine and sodium chloride in rats. Anticancer Res..

[974] Singletary K.W., Nelshoppen J.M., Scardefield S., Wallig M. (1992). Inhibition by butylated hydroxytoluene and its oxidative metabolites of DMBA-induced mammary tumorigenesis and of mammary DMBA-DNA adduct formation in vivo in the female rat. Food Chem. Toxicol..

[975] Williams G.M., Tanaka T., Maeura Y. (1986). Dose-related inhibition of aflatoxin B1 induced hepatocarcinogenesis by the phenolic antioxidants, butylated hydroxyanisole and butylated hydroxytoluene. Carcinogenesis.

[976] Williams G.M., Iatropoulos M.J. (1996). Inhibition of the hepatocarcinogenicity of aflatoxin B1 in rats by low levels of the phenolic antioxidants butylated hydroxyanisole and butylated hydroxytoluene. Cancer Lett..

[977] Simonich M.T., Egner P.A., Roebuck B.D., Orner G.A., Jubert C., Pereira C., Groopman J.D., Kensler T.W., Dashwood R.H., Williams D.E. (2007). Natural chlorophyll inhibits aflatoxin B1-induced multi-organ carcinogenesis in the rat. Carcinogenesis.

[978] Kensler T.W., Egner P.A., Dolan P.M., Groopman J.D., Roebuck B.D. (1987). Mechanism of protection against aflatoxin tumorigenicity in rats fed 5-(2-pyrazinyl)-4-methyl-1,2-dithiol-3-thione (oltipraz) and related 1,2-dithiol-3-thiones and 1,2-dithiol-3-ones. Cancer Res..

[979] Wattenberg L.W., Loub W.D. (1978). Inhibition of polycyclic aromatic hydrocarbon-induced neoplasia by naturally occurring indoles. Cancer Res..

[980] Rauscher R., Edenharder R., Platt K.L. (1998). In vitro antimutagenic and in vivo anticlastogenic effects of carotenoids and solvent extracts from fruits and vegetables rich in carotenoids. Mutat. Res./Genet. Toxicol. Environ. Mutagen..

[981] Tanaka T., Shnimizu M., Moriwaki H. (2012). Cancer chemoprevention by carotenoids. Molecules.

[982] Hecht S.S. (1999). Chemoprevention of cancer by isothiocyanates, modifiers of carcinogen metabolism. J. Nutr..

[983] Wattenberg L.W. (1981). Inhibition of carcinogen-induced neoplasia by sodium cyanate, tert-butyl isocyanate, and benzyl isothiocyanate administered subsequent to carcinogen exposure. Cancer Res..

[984] Zhang Y., Kensler T.W., Cho C.G., Posner G.H., Talalay P. (1994). Anticarcinogenic activities of sulforaphane and structurally related synthetic norbornyl isothiocyanates. Proc. Natl. Acad. Sci. USA.

[985] Walters D.G. (2004). Cruciferous vegetable consumption alters the metabolism of the dietary carcinogen 2-amino-1-methyl-6-phenylimidazo[4,5-b]pyridine (PhIP) in humans. Carcinogenesis.

[986] Bayat Mokhtari R., Baluch N., Homayouni T.S., Morgatskaya E., Kumar S., Kazemi P., Yeger H. (2018). The role of Sulforaphane in cancer chemoprevention and health benefits: A mini-review. J. Cell Commun. Signal..

[987] Cömert E.D., Gökmen V. (2018). Evolution of food antioxidants as a core topic of food science for a century. Food Res. Int..

[988] Liu R.H. (2004). Potential synergy of phytochemicals in cancer prevention: Mechanism of action. J. Nutr..

[989] Racchi M.L. (2013). Antioxidant defenses in plants with attention to Prunus and *Citrus spp.*. Antioxidants.

[990] Bishayee A. (2009). Cancer prevention and treatment with resveratrol: From rodent studies to clinical trials. Cancer Prev. Res..

[991] Heidor R., de Conti A., Ortega J.F., Furtado K.S., Silva R.C., Tavares P.E.L.M., Purgatto E., Ract J.N.R., de Paiva S.A.R., Gioielli L.A. (2015). The chemopreventive activity of butyrate-containing structured lipids in experimental rat hepatocarcinogenesis. Mol. Nutr. Food Res..

[992] Guariento A.H., Furtado K.S., de Conti A., Campos A., Purgatto E., Carrilho J., Shinohara E.M.G., Tryndyak V., Han T., Fuscoe J.C. (2014). Transcriptomic responses provide a new mechanistic basis for the chemopreventive effects of folic acid and tributyrin in rat liver carcinogenesis. Int. J. Cancer.

[993] Ulrich S., Wolter F., Stein J.M. (2005). Molecular mechanisms of the chemopreventive effects of resveratrol and its analogs in carcinogenesis. Mol. Nutr. Food Res..

[994] Dashwood R.H. (1998). Indole-3-carbinol: Anticarcinogen or tumor promoter in brassica vegetables?. Chem. Biol. Interact..

[995] Lake B.G. (1999). Coumarin metabolism, toxicity and carcinogenicity: Relevance for human risk assessment. Food Chem. Toxicol..

